# Supplementary material for: Exploring bidirectional causality between religion and mental health: A longitudinal study using data from the parent generation of a UK birth cohort
Source: PLoS One. 2025 Mar 18;20(3):e0319796. doi: 10.1371/journal.pone.0319796 (PMC11918439; doi:10.1371/journal.pone.0319796)
Supplement: S1 File — This supporting information file contains additional information regarding differences from the pre-registered analysis plan (Section S1) and information regarding the multiple imputation sensitivity analysis (Section S2), in addition to all supporting tables (Tables S1-S34) and figures (Figs S1-S48). (PDF) [file pone.0319796.s001.pdf]

Supporting Material for ‘Exploring bidirectional causality between religion and mental health: A longitudinal study using data from the parent generation of a UK birth cohort’

Section S1: Differences from Pre-Registered Analysis Plan (https://osf.io/qtdze/)

The pre-registered analysis plan was followed as specified, with no substantial changes to the research questions or analysis methods. Some minor changes were made, though, as detailed below:

- In the pre-registered analysis plan, we stated that we would use “binary markers to indicate possible depression (EPDS scores of 13 or more; (Matthey et al. 2006)) and anxiety (CCEI-A scores of 9 or more; (Heron et al. 2004)), respectively”. However, these cut-offs correspond to markers of possible depression and anxiety in women, but not in men, for which these thresholds differ. In the updated methods section we have therefore specified different cut-offs for ALSPAC mothers (using the cut-offs described above) and partners (using an EPDS score of 10 or more to indicate probable depression, and a CCEI-A score of 6 or more to indicate probable anxiety).
- In the pre-registered analysis plan, we did not specify a formal test to assess potential gender differences between mothers and partners. A formal test for interactions has now been included in the full paper.
- In the pre-registered analysis plan, we said that we would make the analysis code openly-available, but did not mention data availability (as raw ALSPAC data cannot be made openly-available). However, to facilitate open-science practices and aid reproducibility, in the updated paper we have made synthetic ALSPAC datasets openly-available. These synthesised datasets are modelled on the original ALSPAC data, thus maintaining variable distributions and relations among variables (albeit not perfectly), while at the same time preserving participant anonymity and confidentiality, and can be used to follow and test our analysis code.
- In the pre-registered analysis plan regarding E-value sensitivity analyses for unmeasured confounding, we also stated that “Note that such E-value sensitivity analyses cannot be conducted on categorical outcomes via multinomial models, so when RSBB is the outcome they will only be performed when the RSBB outcome is binary”. This was an error on our part, as such E-value analysis *can* be conducted on multinomial models, and have been used in our paper where appropriate.
- In our original pre-registration document we discussed and justified why we focussed on complete-case analyses, despite missing data in many of our variables. This is because we believed that our complete-case results, although perhaps somewhat inefficient, would be largely unbiased and hence methods such as multiple imputation would not be necessary to remove any selection bias (because any bias would be minimal). Nonetheless, as an additional sensitivity analysis suggested during the review process, we have now performed multiple imputation. Methods and results are discussed in more detail in Section S2 of this Supporting Information, but overall the multiple imputation and complete-case results were broadly consistent with one another.

Section S2: Multiple imputation sensitivity analyses

As an additional sensitivity analysis, we repeated all analyses using multiple imputation to impute missing data.

For each of the four analyses (mental health 6 years post-delivery as outcome and RSBB 5 years post-delivery as outcome, for both mothers and partners) we imputed up to the total sample with data on any of the following: baseline mental health or RSBB data from pregnancy, exposure variables, and outcome variables. For instance, for the analysis of RSBB 5 post-delivery as the exposure and mental health 6 years post-delivery as the outcome in mothers, any mothers with any of the following variables were included in the imputation model: religious belief in pregnancy, religious identity in pregnancy, religious attendance in pregnancy, depression scores in pregnancy, anxiety scores in pregnancy, religious belief at age 5, religious identity at age 5, religious attendance at age 5, depression scores at age 6 and anxiety scores at age 6. We included participants with baseline mental health and RSBB data in these imputation models, even if missing exposure or outcome data, as data in these baseline time-points would be correlated with later mental health and RSBB, and hence provide information for imputing missing data in the later exposure and outcomes. Sample sizes for imputation models were: mental health 6 years post-delivery as outcome for mothers n = 13,085 (95.7% of full ALSPAC sample); mental health 6 years post-delivery as outcome for partners n = 9,745 (73.3% of full ALSPAC sample); RSBB 5 years post-delivery as outcome for mothers n = 13,150 (96.1% of full ALSPAC sample); RSBB 5 years post-delivery as outcome for partners n = 9,887 (74.4% of full ALSPAC sample).

All other confounders in Table S2 were included in the imputation models. As described in the main text, we did not include any additional auxiliary variables because, beyond the included confounder variables, no additional variables were relevant to predicting missing values of our exposures or outcomes. Conditional on all these variables, we therefore assumed that our imputation models were 'Missing-At-Random', and hence would provide unbiased results; although we believe the 'Missing-At-Random' assumption is plausible, we cannot rule out data being 'Missing-Not-At-Random' (e.g., if participants with poorer mental health and/or lower religiosity were less likely to provide baseline data in pregnancy, although levels of missing data in these baseline variables are low, and hence at lower risk of selection bias). To perform imputations we used the R package 'mice' [1], creating 50 imputed datasets with 10 iterations per imputation for burn-in (this was checked to ensure that imputations had converged).

As discussed in main text, because we assumed the complete-case analyses were relatively unbiased we did not expect the multiple imputation results to differ substantially, other than perhaps improving precision of the estimates. Consistent with these expectations, the results of the multiple imputation analyses were broadly comparable to those of the complete-case analyses, with no substantial changes in interpretation. Numeric results for the multiple imputation results are in Tables S23-S34, with plots comparing adjusted estimates between complete-case and multiple imputation analyses in Figures S21-S48.

Table S1: The ALSPAC religious/spiritual beliefs and behaviour variables used in the present study.

**Question**	**Original variable coding**	**Dichotomised variable coding**	**Variable name** (mothers; *partners*)	
			**Pregnancy**	**5 years after delivery**
Do you believe in God or in some divine power?	Unordered category (Yes vs Not sure vs No)	Yes vs No (No = Not sure or No)	d810; *pb150*	k6240; *ph6240*
What sort of religious faith would you say you have?	Unordered category (Christian vs Other religion vs None)	Religious vs None (Religious = Christian or Other religion)	d813; *pb153*	k6243; *ph6243*
Do you go to a place of worship?	Ordered category (At least once a week vs At least once a month vs At least once a year vs Not at all) [a]	Regular attendance vs Occasional/non-attendance (Regular = At least once a week or at least once a month; Occasional/non-attendance = At least once a year or not at all)	d816; *pb155*	k6247; *ph6247*

[a] At the 5 year time-point a small number of participants answered ‘occasionally’ (11 mothers and < 5 partners), which we have combined with ‘At least once a year’ here.

Table S2: The baseline ALSPAC variables measured in pregnancy used as confounders in the present study (excluding religious/spiritual beliefs and behaviours and mental health, detailed in the main text).

**Variable**	**ALSPAC cohort (variable name)**	**Variable coding**
Age at delivery	Mothers (*mz028b*); Partners (*partner_age*)	Continuous (years)
Ethnicity	Mothers (*c800*); Partners (*pb440*)	Binary (White vs other than White)
Marital status	Mothers (*a525*); Partners (*pa065*)	Unordered category (never married vs currently married vs widowed/divorced/ separated)
Maternal parity	Mothers and partners (b032)	Ordered category (0 vs 1 vs 2 or more)
Urban/Rural location	Mothers and partners (*jan1993ur01ind_M*)	Binary (town/village/hamlet vs urban)
Highest educational qualification	Mother (*c645a*); Partner (*pb325a*)	Ordered category (CSE/none vs vocational vs O-level vs A-level vs degree) [a]
Occupational social class	Mother (*c755*); Partner (*pb_sc_p*)	Binary (low [III manual/IV/V] vs high [I/II/III non-manual]) [b]
Index of multiple deprivation	Mothers and partners (*jan1993imd2010q5_M*)	Ordered category (1st quintile [least deprived] vs 2nd quintile vs 3rd quintile vs 4th quintile vs 5th quintile [most deprived])
Recent financial difficulties	Mothers (*b594*); Partners (*pb184*)	Binary (Yes vs No)
Housing status	Mothers and partners (*a006*)	Unordered category (owned/mortgaged vs renting vs council/housing association vs other)
Household access to car	Mothers and partners (*a053*)	Binary (Yes vs No)
Employment status	Mothers (*c710; c711; c712; c713*); Partners (*pb380; pb381; pb382; pb383*)	Binary (Yes vs No)
Adverse childhood experiences	Mothers (*c433*); Partners (*pb482*)	Continuous (0-31; number of experiences)
Locus of control	Mothers (*d842*); Partners (*pa782*)	Continuous (0-12; higher scores indicate a more external locus of control; [2])
Interpersonal sensitivity	Mothers (*b921*); Partners (*pb551*)	Continuous (36-144; higher scores indicate greater interpersonal sensitivity; [3])
Subjective health status	Mothers (*b040*); Partners (*a524*)	Ordered category (Always well vs Usually well vs Sometimes/Often/Always unwell)
Body Mass Index (BMI; pre-pregnancy for mothers)	Mothers (*dw042*); Partners (*paw002; paw010*)	Continuous (Weight [kg] / Height [m] ^{2})
Self-reported physical activity, relative to others	Mothers (*b633*); Partners (*pb012*)	Ordered category (Much more active vs Somewhat more active vs About the same vs Somewhat less active Vs Much less active)
Smoking status	Mothers (*b650; b659*); Partners (*pb071; pb074*)	Ordered category (Currently smokes vs Formerly smoked vs Never smoked)
Alcohol intake (prior to pregnancy)	Mothers (*b720*); Partners (*pb099*)	Ordered category (Never vs < 1 glass per week vs 1 or more glasses per week vs 1-2 glasses per day vs 3 or more glasses per day)

Social networks	Mothers (*d780*); Partners (*pb120*)	Continuous (1-29; higher scores indicate a larger and more supportive social network)
Social support	Mothers (*d800*); Partners (*pb140*)	Continuous (0-30; higher scores indicate greater perceptions of social support)
Maternal history of depression/anxiety	Mothers (*d536a*); Partners (*pa536a*)	Binary (Yes vs No)
Paternal history of depression/anxiety	Mothers (*d586a*); Partners (*pa586a*)	Binary (Yes vs No)

[a] CSE = Certificate of Secondary Education qualification (examinations sat at the end of secondary school at approx. age 16; compulsory from the early 1970s, unless completing O-level qualifications instead; replaced in 1986 by GCSEs); O-level = Ordinary level qualifications (examinations sat at the end of secondary school, often for more academically-able pupils at approx. age 16; replaced in 1986 by GCSEs); A-level = Advanced level qualification (non-compulsory examinations sat at the end of college or sixth form at approx. age 18).
[b] For more information on these occupational social classes, see: https://sru.soc.surrey.ac.uk/SRU9.html.

Table S3: Summary of models where religious/spiritual beliefs and behaviours (RSBB) 5 years post-delivery are the exposures and mental health 6 years post-delivery are the outcomes. For all models, we compared unadjusted and adjusted estimates, where adjusted models control for all baseline confounders in table S2, plus baseline RSBB and mental health. All models were repeated for both mothers and partners.

**Model**	**RSBB Exposure**	**Mental Health Outcome**	**Regression Model**
1a	Religious belief (categorical)	Depression (continuous)	Linear
1b	Religious affiliation (categorical)	Depression (continuous)	Linear
1c	Religious attendance (categorical)	Depression (continuous)	Linear
2a	Religious belief (categorical)	Anxiety (continuous)	Linear
2b	Religious affiliation (categorical)	Anxiety (continuous)	Linear
2c	Religious attendance (categorical)	Anxiety (continuous)	Linear
3a	Religious belief (binary)	Depression (continuous)	Linear
3b	Religious affiliation (binary)	Depression (continuous)	Linear
3c	Religious attendance (binary)	Depression (continuous)	Linear
4a	Religious belief (binary)	Anxiety (continuous)	Linear
4b	Religious affiliation (binary)	Anxiety (continuous)	Linear
4c	Religious attendance (binary)	Anxiety (continuous)	Linear
5a	Religious belief (categorical)	Depression (binary)	Logistic
5b	Religious affiliation (categorical)	Depression (binary)	Logistic
5c	Religious attendance (categorical)	Depression (binary)	Logistic
6a	Religious belief (categorical)	Anxiety (binary)	Logistic
6b	Religious affiliation (categorical)	Anxiety (binary)	Logistic
6c	Religious attendance (categorical)	Anxiety (binary)	Logistic
7a	Religious belief (binary)	Depression (binary)	Logistic
7b	Religious affiliation (binary)	Depression (binary)	Logistic
7c	Religious attendance (binary)	Depression (binary)	Logistic
8a	Religious belief (binary)	Anxiety (binary)	Logistic
8b	Religious affiliation (binary)	Anxiety (binary)	Logistic
8c	Religious attendance (binary)	Anxiety (binary)	Logistic

Table S4: Summary of models where mental health 2 years post-delivery are the exposures and religious/spiritual beliefs and behaviours (RSBB) 5 years post-delivery are the outcomes. For all models, we compared unadjusted and adjusted estimates, where adjusted models control for all baseline confounders in table S2, plus baseline RSBB and mental health. All models were repeated for both mothers and partners.

Model	Mental Health Exposure	RSBB Outcome	Regression Model
1a	Depression (continuous)	Religious belief (categorical)	Multinomial
1b	Anxiety (continuous)	Religious belief (categorical)	Multinomial
2a	Depression (continuous)	Religious affiliation (categorical)	Multinomial
2b	Anxiety (continuous)	Religious affiliation (categorical)	Multinomial
3a	Depression (continuous)	Religious attendance (categorical)	Multinomial
3b	Anxiety (continuous)	Religious attendance (categorical)	Multinomial
4a	Depression (binary)	Religious belief (categorical)	Multinomial
4b	Anxiety (binary)	Religious belief (categorical)	Multinomial
5a	Depression (binary)	Religious affiliation (categorical)	Multinomial
5b	Anxiety (binary)	Religious affiliation (categorical)	Multinomial
6a	Depression (binary)	Religious attendance (categorical)	Multinomial
6b	Anxiety (binary)	Religious attendance (categorical)	Multinomial
7a	Depression (continuous)	Religious belief (binary)	Logistic
7b	Anxiety (continuous)	Religious belief (binary)	Logistic
8a	Depression (continuous)	Religious affiliation (binary)	Logistic
8b	Anxiety (continuous)	Religious affiliation (binary)	Logistic
9a	Depression (continuous)	Religious attendance (binary)	Logistic
9b	Anxiety (continuous)	Religious attendance (binary)	Logistic
10a	Depression (binary)	Religious belief (binary)	Logistic
10b	Anxiety (binary)	Religious belief (binary)	Logistic
11a	Depression (binary)	Religious affiliation (binary)	Logistic
11b	Anxiety (binary)	Religious affiliation (binary)	Logistic
12a	Depression (binary)	Religious attendance (binary)	Logistic
12b	Anxiety (binary)	Religious attendance (binary)	Logistic

Table S5: Descriptive statistics for all variables in the ALSPAC mothers. Results are presented for the full ALSPAC dataset (*n* = 13,678), the complete-case data with mental health 6 years post-delivery as the outcome (*n* = 3,856), and the complete-case data with RSBB 5 years post-delivery as the outcome (*n* = 4,025). Depending on whether variables are numeric or categorical, cells represent either means (standard deviations in brackets) or counts (percentages in brackets).

**Variable**	**Full cohort**	**Complete-case data for mental health at age 6 as outcome**	**Complete-case data for RSBB at age 5 as outcome**
Depression score (pregnancy)			
	6.99 (4.85)	6.28 (4.55)	6.27 (4.51)
Missing	1,811 (13.2%)	-	-
Anxiety score (pregnancy)			
	4,92 (3.55)	4,52 (3.35)	4.53 (3.36)
Missing	1,855 (13.8%)	-	-
Religious belief (pregnancy)			
No	1,789 (14.8%)	433 (11.2%)	450 (11.2%)
Not sure	4,262 (35.3%)	1,345 (34.9%)	1,411 (35.1%)
Yes	6,024 (49.9%)	2,078 (53.9%)	2,164 (53.8%)
Missing	1,603 (11.7%)	-	-
Religious identity (pregnancy)			
None	1,821 (15.3%)	488 (12.7%)	510 (12.7%)
Christian	9,600 (80.5%)	3,250 (84.3%)	3,388 (84.2%)
Other religion	508 (4.3%)	118 (3.06%)	127 (3.2%)
Missing	1,749 (12.8%)	-	-
Religious service attendance (pregnancy)			
Not at all	6,663 (56.4%)	1,945 (50.4%)	2,041 (50.7%)
Min. once a year	3,459 (29.3%)	1,300 (33.7%)	1,352 (33.6%)
Min. once a month	809 (6.9%)	310 (8.0%)	319 (7.9%)
Min. once a week	874 (7.4%)	301 (7.8%)	313 (7.8%)
Missing	1,873 (13.7%)	-	-
Depression score (2 years post-delivery)			
	5.73 (4.79)	-	5.39 (4.60)
Missing	3,551 (26.0%)	-	-
Probable depression diagnosis (2 years post-delivery)			
No	9,124 (90.1%)	-	3,682 (91.5%)
Yes	1,003 (9.9%)	-	343 (8.5%)
Missing	3,551 (26.0%)	-	-
Anxiety score (2 years post-delivery)			
	3.78 (3.33)	-	3.61 (3.19)

Variable	Full cohort	Complete-case data for mental health at age 6 as outcome	Complete-case data for RSBB at age 5 as outcome
Missing	3,550 (26.0%)	-	-
Probable anxiety diagnosis (2 years post-delivery)			
No	9,097 (89.8%)	-	3,677 (91.4%)
Yes	1,031 (10.2%)	-	348 (8.6%)
Missing	3,550 (26.0%)	-	-
Religious belief (5 years post-delivery)			
No	1,730 (19.6%)	639 (16.6%)	673 (16.7%)
Not sure	2,985 (33.9%)	1,330 (34.5%)	1,387 (34.5%)
Yes	4,093 (46.5%)	1,887 (48.9%)	1,965 (48.8%)
Missing	4,870 (35.6%)	-	-
Religious identity (5 years post-delivery)			
None	1,341 (15.5%)	507 (13.2%)	535 (13.3%)
Christian	7,051 (81.4%)	3,256 (84.4%)	3,394 (84.3%)
Other religion	271 (3.1%)	93 (2.4%)	96 (2.4%)
Missing	5,025 (36.7%)	-	-
Religious service attendance (5 years post-delivery)			
Not at all	4,553 (53.4%)	1,956 (50.7%)	2,050 (50.9%)
Min. once a year	2,269 (26.6%)	1,074 (27.9%)	1,119 (27.8%)
Min. once a month	835 (9.8%)	413 (10.7%)	431 (10.7%)
Min. once a week	873 (10.2%)	413 (10.7%)	425 (10.6%)
Missing	5,148 (37.6%)	-	-
Depression score (6 years post-delivery)			
	6.36 (5.15)	6.01 (4.92)	-
Missing	5,263 (38.5%)	-	-
Probable depression diagnosis (6 years post-delivery)			
No	7,279 (86.5%)	3,411 (88.5%)	-
Yes	1,136 (13.5%)	445 (11.5%)	-
Missing	5,263 (38.5%)	-	-
Anxiety score (6 years post-delivery)			
	5.14 (3.64)	4.94 (3.48)	-
Missing	5,272 (38.5%)	-	-
Probable anxiety diagnosis (6 years post-delivery)			
No	6,919 (82.3%)	3,257 (84.5%)	-
Yes	1,487 (17.7%)	599 (15.5%)	-
Missing	5,272 (38.5%)	-	-

Variable	Full cohort	Complete-case data for mental health at age 6 as outcome	Complete-case data for RSBB at age 5 as outcome
Age at delivery			
	27.99 (4.97)	29.08 (4.24)	29.05 (4.27)
Missing	0 (0.0%)	-	-
Ethnicity			
White	11,730 (97.4%)	3,811 (98.8%)	3,977 (98.8%)
Other than White	318 (2.6%)	45 (1.2%)	48 (1.2%)
Missing	1,630 (11.9%)	-	-
Marital status			
Married	9,599 (74.9%)	3,265 (84.7%)	3,403 (84.6%)
Never married	2,439 (19.0%)	415 (10.8%)	435 (10.8%)
Separated/Widowed/ Divorced	772 (6.0%)	176 (4.6%)	187 (4.6%)
Missing	868 (6.4%)	-	-
Maternal parity			
0	5,651 (44.6%)	1,871 (48.5%)	1,955 (48.6%)
1	4,445 (35.1%)	1,409 (36.5%)	1,454 (36.1%)
2 or more	2,573 (20.3%)	576 (15.9%)	616 (15.3%)
Missing	1,009 (7.4%)	-	-
Urban/Rural location			
Urban	11,372 (90.2%)	3,423 (88.8%)	3,567 (88.6%)
Rural	1,234 (9.8%)	433 (11.2%)	458 (11.4%)
Missing	1,072 (7.8%)	-	-
Highest educational qualification			
CSE/None	2,444 (20.1%)	392 (10.2%)	420 (10.4%)
Vocational	1,200 (9.9%)	312 (8.1%)	311 (7.7%)
O-level	4,208 (34.7%)	1,469 (38.1%)	1,545 (38.4%)
A-level	2,722 (22.4%)	1,050 (27.2%)	1,099 (27.3%)
Degree	1,564 (12.9%)	633 (16.4%)	650 (16.3%)
Missing	1,540 (11.3%)	-	-
Occupational social class			
Low	1,946 (19.8%)	602 (15.6%)	635 (15.8%)
High	7,885 (80.2%)	3,254 (84.4%)	3,390 (84.2%)
Missing	3,847 (28.1%)	-	-
Index of multiple deprivation			
Quintile 1 (Least deprived)	3,135 (24.9%)	1,246 (32.3%)	1,283 (31.9%)
Quintile 2	2,687 (21.4%)	981 (25.4%)	1,016 (25.2%)
Quintile 3	2,191 (17.4%)	688 (17.8%)	729 (18.1%)

Variable	Full cohort	Complete-case data for mental health at age 6 as outcome	Complete-case data for RSBB at age 5 as outcome
Quintile 4	2,453 (19.5%)	614 (15.9%)	651 (16.2%)
Quintile 5 (Most deprived)	2,108 (16.7%)	327 (8.5%)	346 (8.6%)
Missing	1,104 (8.1%)	-	-
Recent financial difficulties			
No	10,083 (86.4%)	3,433 (89.3%)	3,592 (89.2%)
Yes	1,582 (13.6%)	413 (10.7%)	433 (10.8%)
Missing	2,013 (14.7%)	-	-
Housing status			
Owned/Mortgaged	9,359 (73.3%)	3,338 (86.6%)	3,480 (86.5%)
Rented	914 (7.2%)	164 (4.3%)	171 (4.2%)
Council/Housing Association	2,043 (16.0%)	275 (7.1%)	290 (7.2%)
Other	450 (3.5%)	79 (2.0%)	84 (2.1%)
Missing	912 (6.7%)	-	-
Household access to car			
No	1,381 (10.8%)	143 (3.7%)	153 (3.8%)
Yes	11,393 (89.2%)	3,713 (96.3%)	3,872 (96.2%)
Missing	904 (6.6%)	-	-
Employed			
No	7,492 (61.3%)	1,946 (50.5%)	2,038 (50.6%)
Yes	4,721 (38.7%)	1,910 (49.5%)	1,987 (49.4%)
Missing	1,465 (10.7%)	-	-
Adverse childhood experiences			
	3.80 (3.00)	3.66 (2.73)	3.68 (2.76)
Missing	1,588 (11.6%)	-	-
Locus of control			
	4.36 (2.17)	3.88 (2.02)	3.89 (2.02)
Missing	3,345 (24.5%)	-	-
Interpersonal sensitivity			
	89.70 (16.33)	90.42 (15.25)	90.39 (15.27)
Missing	981 (7.2%)	-	-
Subjective health status			
Always well	3,735 (31.8%)	1,399 (36.3%)	1,438 (35.7%)
Usually well	7.072 (60.2%)	2,251 (58.4%)	2,373 (59.0%)
Often unwell	937 (8.0%)	206 (5.3%)	214 (5.3%)
Missing	1,934 (14.4%)	-	-

Variable	Full cohort	Complete-case data for mental health at age 6 as outcome	Complete-case data for RSBB at age 5 as outcome
Body Mass Index (BMI)			
	22.93 (3.84)	22.89 (3.58)	22.87 (3.58)
Missing	2,392 (17.5%)	-	-
Self-reported physical activity (relate to similar others)			
Much less	210 (1.8%)	44 (1.1%)	46 (1.1%)
Somewhat less	1,272 (10.9%)	392 (10.2%)	401 (10.0%)
About the same	7,257 (62.0%)	2,370 (61.5%)	2,472 (61.4%)
Somewhat more	2,318 (19.8%)	839 (21.8%)	889 (22.1%)
Much more	653 (5.6%)	211 (5.5%)	217 (5.4%)
Missing	1,968 (14.4%)	-	-
Smoking status			
Never	6,296 (49.5%)	2,207 (57.2%)	2,280 (56.7%)
Former	3,772 (29.7%)	1,120 (29.1%)	1,181 (29.3%)
Current	2,643 (20.8%)	529 (13.7%)	564 (14.0%)
Missing	967 (7.1%)	-	-
Alcohol intake			
Never	1,069 (8.4%)	204 (5.3%)	209 (5.2%)
<1 per week	4,803 (37.6%)	1,407 (36.5%)	1,475 (36.6%)
1+ per week	5,466 (42.8%)	1,792 (46.5%)	1,866 (46.4%)
1+ per day	1,217 (9.5%)	404 (10.5%)	426 (10.6%)
3+ per day	211 (1.7%)	49 (1.3%)	49 (1.2%)
Missing	912 (6.7%)	-	-
Social networks			
	23.27 (3.91)	23.72 (3.49)	23.72 (3.50)
Missing	1,842 (13.5%)	-	-
Social support			
	19.59 (5.08)	20.07 (4.74)	20.11 (4.78)
Missing	2,484 (18.2%)	-	-
Maternal history of depression/anxiety			
No	9,738 (79.9%)	3,129 (81.2%)	3,263 (81.1%)
Yes	2,449 (20.1%)	727 (18.8%)	762 (18.9%)
Missing	1,491 (10.9%)	-	-
Paternal history of depression/anxiety			
No	11,304 (92.8%)	3,589 (93.1%)	3,737 (92.8%)
Yes	883 (7.2%)	267 (6.9%)	288 (7.2%)
Missing	1,491 (10.9%)	-	-

Table S6: Descriptive statistics for all variables in the ALSPAC partners. Results are presented for the full ALSPAC dataset (*n* = 13,296), the complete-case data with mental health 6 years post-delivery as the outcome (*n* = 1,940), and the complete-case data with RSBB 5 years post-delivery as the outcome (*n* = 2,120). Depending on whether variables are numeric or categorical, cells represent either means (standard deviations in brackets) or counts (percentages in brackets).

**Variable**	**Full cohort**	**Complete-case data for mental health at age 6 as outcome**	**Complete-case data for RSBB at age 5 as outcome**
Depression score (pregnancy)			
	4.21 (3.91)	3.58 (3.46)	3.63 (3.48)
Missing	3,990 (30.0%)	-	-
Anxiety score (pregnancy)			
	2.97 (2.78)	2.74 (2.52)	2,80 (2.57)
Missing	4,005 (30.1%)	-	-
Religious belief (pregnancy)			
No	2,614 (28.3%)	498 (25.7%)	538 (25.4%)
Not sure	3,194 (34.6%)	679 (35.0%)	749 (35.3%)
Yes	3,420 (37.1%)	763 (39.3%)	833 (39.3%)
Missing	4,068 (30.6%)	-	-
Religious identity (pregnancy)			
None	2,340 (25.8%)	482 (24.9%)	529 (25.0%)
Christian	6,262 (69.0%)	1,361 (70.1%)	1,497 (70.6%)
Other religion	478 (5.3%)	97 (5.0%)	94 (4.4%)
Missing	4,216 (31.7%)	-	-
Religious service attendance (pregnancy)			
Not at all	5,711 (63.2%)	1,077 (55.5%)	1,181 (55.7%)
Min. once a year	2,375 (26.3%)	598 (30.8%)	666 (31.4%)
Min. once a month	395 (4.4%)	110 (5.7%)	120 (5.7%)
Min. once a week	559 (6.2%)	155 (8.0%)	153 (7.2%)
Missing	4,256 (32.0%)	-	-
Depression score (2 years post-delivery)			
	3.64 (3.79)	-	3.25 (3.42)
Missing	7,405 (55.7%)	-	-
Probable depression diagnosis (2 years post-delivery)			
No	5,411 (91.9%)	-	1,988 (93.8%)
Yes	480 (8.2%)	-	132 (6.2%)
Missing	7,405 (55.7%)	-	-
Anxiety score (2 years post-delivery)			

Variable	Full cohort	Complete-case data for mental health at age 6 as outcome	Complete-case data for RSBB at age 5 as outcome
	2.57 (2.56)	-	2.41 (2.35)
Missing	7,434 (55.9%)	-	-
Probable anxiety diagnosis (2 years post-delivery)			
No	5.170 (88.2%)	-	1,898 (89.5%)
Yes	692 (11.8%)	-	222 (10.5%)
Missing	7,434 (55.9%)	-	-
Religious belief (5 years post-delivery)			
No	1,349 (31.2%)	541 (27.9%)	608 (28.7%)
Not sure	1,522 (35.2%)	715 (36.9%)	782 (36.9%)
Yes	1,454 (33.6%)	684 (35.3%)	730 (34.4%)
Missing	8,971 (67.5%)	-	-
Religious identity (5 years post-delivery)			
None	1,007 (23.9%)	429 (22.1%)	470 (22.2%)
Christian	3,081 (73.2%)	1,469 (75.7%)	1,608 (75.8%)
Other religion	123 (2.9%)	42 (2.2%)	42 (2.0%)
Missing	9,085 (68.3%)	-	-
Religious service attendance (5 years post-delivery)			
Not at all	2,601 (62.1%)	1,153 (59.4%)	1,281 (60.4%)
Min. once a year	958 (22.9%)	462 (23.8%)	506 (23.9%)
Min. once a month	278 (6.6%)	143 (7.4%)	154 (7.3%)
Min. once a week	349 (8.3%)	182 (9.4%)	179 (8.4%)
Missing	9,110 (68.5%)	-	-
Depression score (6 years post-delivery)			
	4.43 (4.35)	4.27 (4.27)	-
Missing	9,022 (67.9%)	-	-
Probable depression diagnosis (6 years post-delivery)			
No	3,687 (86.3%)	1,682 (86.7%)	-
Yes	587 (13.7%)	258 (13.3%)	-
Missing	9,022 (67.9%)	-	-
Anxiety score (6 years post-delivery)			
	3.79 (2.89)	3.67 (2.79)	-
Missing	9,028 (67.9%)	-	-
Probable anxiety diagnosis (6 years post-delivery)			
No	3,295 (77.2%)	1,524 (78.6%)	-
Yes	973 (22.8%)	416 (21.4%)	-
Missing	9,028 (67.9%)	-	-

Variable	Full cohort	Complete-case data for mental health at age 6 as outcome	Complete-case data for RSBB at age 5 as outcome
Age at delivery			
	30.69 (5.79)	31.74 (5.10)	31.69 (5.10)
Missing	2,388 (18.0%)	-	-
Ethnicity			
White	8.945 (97.1%)	1,920 (99.0%)	2,101 (99.1%)
Other than White	272 (2.9%)	20 (1.0%)	19 (0.9%)
Missing	4,079 (30.7%)	-	-
Marital status			
Married	6,633 (82.5%)	1,759 (90.7%)	1,921 (90.6%)
Never married	1,067 (13.3%)	125 (6.4%)	144 (6.8%)
Separated/Widowed/ Divorced	344 (4.3%)	56 (2.9%)	55 (2.6%)
Missing	5,252 (39.5%)	-	-
Maternal parity			
0	5,465 (44.4%)	958 (49.4%)	1,058 (49.9%)
1	4,336 (35.2%)	680 (35.0%)	722 (34.1%)
2 or more	2,503 (20.3%)	302 (15.6%)	340 (16.0%)
Missing	992 (7.5%)	-	-
Urban/Rural location			
Urban	11,036 (90.1%)	1,697 (87.5%)	1,853 (87.4%)
Rural	1,209 (9.9%)	243 (12.5%)	267 (12.6%)
Missing	1,051 (7.9%)	-	-
Highest educational qualification			
CSE/None	1,987 (21.4%)	235 (12.1%)	247 (11.6%)
Vocational	766 (8.3%)	109 (5.6%)	116 (5.5%)
O-level	2,066 (22.3%)	421 (21.7%)	483 (22.8%)
A-level	2,593 (28.0%)	581 (30.0%)	653 (30.8%)
Degree	1,856 (20.0%)	594 (30.6%)	621 (29.3%)
Missing	4,028 (30.3%)	-	-
Occupational social class			
Low	4,105 (46.2%)	659 (34.0%)	729 (34.4%)
High	4,785 (53.8%)	1,281 (66.0%)	1,391 (65.6%)
Missing	4,406 (33.1%)	-	-
Index of multiple deprivation			
Quintile 1 (Least deprived)	3,068 (25.1%)	716 (36.9%)	766 (36.1%)
Quintile 2	2,616 (21.4%)	488 (25.2%)	535 (25.2%)

Variable	Full cohort	Complete-case data for mental health at age 6 as outcome	Complete-case data for RSBB at age 5 as outcome
Quintile 3	2,141 (17.5%)	348 (17.9%)	382 (18.0%)
Quintile 4	2,362 (19.3%)	259 (13.3%)	300 (14.2%)
Quintile 5 (Most deprived)	2,027 (16.6%)	129 (6.7%)	137 (6.5%)
Missing	1,082 (8.1%)	-	-
Recent financial difficulties			
No	7,709 (84.1%)	1,743 (89.9%)	1,895 (89.4%)
Yes	1,462 (15.9%)	197 (10.1%)	225 (10.6%)
Missing	4,124 (31.0%)	-	-
Housing status			
Owned/Mortgaged	9,130 (73.7%)	1,736 (89.5%)	1,894 (89.3%)
Rented	881 (7.1%)	66 (3.4%)	72 (3.4%)
Council/Housing Association	1,952 (15.7%)	97 (5.0%)	109 (5.1%)
Other	432 (3.5%)	41 (2.1%)	45 (2.1%)
Missing	901 (6.8%)	-	-
Household access to car			
No	1,310 (10.6%)	47 (2.4%)	52 (2.5%)
Yes	11,096 (89.4%)	1,893 (97.6%)	2,068 (97.5%)
Missing	890 (6.7%)	-	-
Employed			
No	1,188 (12.6%)	86 (4.4%)	96 (4.5%)
Yes	8,218 (87.4%)	1,854 (95.6%)	2,024 (95.5%)
Missing	3,890 (29.3%)	-	-
Adverse childhood experiences			
	4.55 (3.20)	4.58 (2.91)	4.57 (2.87)
Missing	3,890 (29.3%)	-	-
Locus of control			
	3.76 (2.30)	3.17 (2.11)	3,18 (2.11)
Missing	6,333 (47.6%)	-	-
Interpersonal sensitivity			
	82.2 (16.53)	83.78 (14.60)	83.78 (14.59)
Missing	4,193 (31.5%)	-	-
Subjective health status			
Always well	5,656 (46.8%)	977 (50.4%)	1,049 (49.5%)
Usually well	5,922 (49.0%)	916 (47.2%)	1,023 (48.2%)
Often unwell	515 (4.3%)	47 (2.4%)	48 (2.3%)

**Variable**	**Full cohort**	**Complete-case data for mental health at age 6 as outcome**	**Complete-case data for RSBB at age 5 as outcome**
Missing	1,203 (9.1%)	-	-
Body Mass Index (BMI)			
	25.18 (3.30)	25.04 (3.14)	25.08 (3.12)
Missing	5,415 (40.7%)	-	-
Self-reported physical activity (relate to similar others)			
Much less	60 (0.6%)	8 (0.4%)	10 (0.5%)
Somewhat less	686 (7.4%)	163 (8.4%)	171 (8.1%)
About the same	4,561 (48.9%)	926 (47.7%)	1,030 (48.6%)
Somewhat more	2,891 (31.0%)	620 (32.0%)	667 (31.5%)
Much more	1,127 (12.1%)	223 (11.5%)	242 (11.4%)
Missing	3,971 (29.9%)	-	-
Smoking status			
Never	4,206 (45.7%)	1,075 (55.4%)	1,167 (55.0%)
Former	2,037 (22.1%)	440 (22.7%)	487 (23.0%)
Current	2,971 (32.2%)	425 (21.9%)	466 (22.0%)
Missing	4,082 (30.7%)	-	-
Alcohol intake			
Never	348 (3.7%)	49 (2.5%)	51 (2.4%)
<1 per week	1,990 (21.4%)	368 (19.0%)	407 (19.2%)
1+ per week	4,812 (51.6%)	1,068 (55.1%)	1,165 (54.9%)
1+ per day	1,601 (17.2%)	358 (18.5%)	393 (18.5%)
3+ per day	567 (6.1%)	97 (5.0%)	104 (4.9%)
Missing	3,978 (29.9%)	-	-
Social networks			
	22.33 (4.03)	22.76 (3.77)	22.76 (3.74)
Missing	4,271 (32.1%)	-	-
Social support			
	17.77 (4.90)	18.49 (4.70)	18.47 (4.74)
Missing	4,557 (34.3%)	-	-
Maternal history of depression/anxiety			
No	6,669 (81.7%)	1,596 (82.3%)	1,744 (82.3%)
Yes	1,492 (18.3%)	344 (17.7%)	376 (17.7%)
Missing	5,135 (38.6%)	-	-
Paternal history of depression/anxiety			
No	7,649 (93.7%)	1,818 (93.7%)	1,982 (93.5%)
Yes	512 (6.3%)	122 (6.3%)	138 (6.5%)

Variable	Full cohort	Complete-case data for mental health at age 6 as outcome	Complete-case data for RSBB at age 5 as outcome
Missing	5,135 (38.6%)	-	-

Table S7: Descriptive statistics for religious/spiritual beliefs and behaviour (RSBB) exposures 5 years post-delivery and mental health outcomes 6 years post-delivery for mothers (*n* = 3,856). EPDS = Edinburgh Postnatal Depression Scale; CCEI-A = Crown-Crisp Experiential Inventory – Anxiety subscale; SD = Standard deviation.

	**EPDS depression scores (Mean = 6.01 [SD = 4.92])**	**Probable depression diagnosis (*n* = 445 [11.5%])**	**CCEI-A anxiety scores (Mean = 4.94 [SD = 3.48])**	**Probable anxiety diagnosis (*n* = 599 [15.5%])**
	Mean (SD)	*n (%)*	*Mean (SD)*	*n (%)*
**Religious Belief (categorical)**				
No (n = 639 [16.6%])	5.82 (5.13)	80 (12.5%)	4.91 (3.76)	115 (18.0%)
Not Sure (n = 1,330 [34.5%])	6.28 (5.00)	172 (12.9%)	5.02 (3.47)	217 (16.3%)
Yes (n = 1,887 [48.9%])	5.89 (4.79)	193 (10.2%)	4.90 (3.38)	267 (14.2%)
**Religious Belief (binary)**				
No/Not Sure (n = 1,969 [51.1%])	6.13 (5.04)	252 (12.8%)	4.98 (3.57)	332 (16.9%)
Yes (n = 1,887 [48.9%])	5.89 (4.79)	193 (10.2%)	4.90 (3.38)	267 (14.2%)
**Religious Affiliation (categorical)**				
None (n = 507 [13.2%])	6.21 (5.19)	70 (13.8%)	5.14 (3.67)	95 (18.7%)
Christian (n = 3,256 [84.4%])	5.97 (4.88)	363 (11.2%)	4.90 (3.44)	489 (15.0%)
Other religion (n = 93 [2.4%])	6.32 (4.89)	12 (12.9%)	5.42 (3.58)	15 (16.1%)
**Religious Affiliation (binary)**				
None (n = 507 [13.2%])	6.21 (5.19)	70 (13.8%)	5.14 (3.67)	95 (18.7%)
Religious (n = 3,349 [86.8%])	5.98 (4.88)	375 (11.2%)	4.91 (3.44)	504 (15.1%)
**Religious Attendance (categorical)**				
Not at all (n = 1,956 [50.7%])	6.15 (5.09)	248 (12.7%)	5.05 (3.66)	342 (17.5%)
Min. 1/year (n = 1,074 [27.9%])	5.84 (4.75)	114 (10.6%)	4.82 (3.22)	143 (13.3%)
Min. 1/month (n = 413 [10.7%])	6.03 (4.97)	46 (11.1%)	4.93 (3.40)	62 (15.0%)
Min. 1/week (n = 413 [10.7%])	5.77 (4.46)	37 (9.0%)	4.76 (3.28)	52 (12.6%)
**Religious Attendance (binary)**				
Occasional/Never (n = 3,030 [78.6%])	6.04 (4.97)	362 (12.0%)	4.97 (3.51)	485 (16.0%)
Regular (n = 826 [21.4%])	5.90 (4.72)	83 (10.0%)	4.84 (3.34)	114 (13.8%)

Table S8: Results of the mothers analyses with religious/spiritual belief and behaviour (RSBB) exposures and depression and anxiety as outcomes (*n* = 3,856). Est = Estimate (either mean difference from a linear model, or odds ratio from a logistic model); LCI = Lower 95% confidence interval; UCI = Upper 95% confidence interval; *p* = *p*-value; no adj = unadjusted model; adj = Adjusted model; std = model using standardised depression or anxiety scores.

**Model num.**	**RSBB expo-sure**	**RSBB expo-sure level**	**Out-come**	**Est (no adj)**	**LCI (no adj)**	**UCI (no adj)**	**Est (no adj; std)**	**LCI (no adj; std)**	**UCI (no adj; std)**	***p* (no adj)**	**Est (adj)**	**LCI (adj)**	**UCI (adj)**	**Est (adj; std)**	**LCI (adj; std)**	**UCI (adj; std)**	***p* (adj)**
1a	Belief (cat)	Not sure	Dep (cont)	0.464	0.000	0.928	0.094	0.000	0.189	0.0500	0.090	-0.387	0.568	0.018	-0.079	0.115	0.7113
1a	Belief (cat)	Yes	Dep (cont)	0.067	-0.375	0.508	0.014	-0.076	0.103	0.7675	-0.245	-0.792	0.302	-0.050	-0.161	0.061	0.3796
1b	Identity (cat)	Christian	Dep (cont)	-0.234	-0.694	0.227	-0.047	-0.141	0.046	0.3203	-0.422	-0.951	0.107	-0.086	-0.193	0.022	0.1178
1b	Identity (cat)	Other	Dep (cont)	0.115	-0.973	1.204	0.023	-0.198	0.245	0.8352	-1.010	-2.041	0.020	-0.205	-0.415	0.004	0.0547
1c	Attend (cat)	1/yr	Dep (cont)	-0.312	-0.678	0.055	-0.063	-0.138	0.011	0.0953	-0.234	-0.607	0.139	-0.048	-0.123	0.028	0.2188
1c	Attend (cat)	1/mth	Dep (cont)	-0.128	-0.650	0.395	-0.026	-0.132	0.080	0.6316	-0.531	-1.071	0.009	-0.108	-0.218	0.002	0.0537
1c	Attend (cat)	1/wk	Dep (cont)	-0.384	-0.907	0.138	-0.078	-0.184	0.028	0.1491	-0.462	-1.097	0.174	-0.094	-0.223	0.035	0.1543
2a	Belief (cat)	Not sure	Anx (cont)	0.116	-0.212	0.444	0.033	-0.061	0.128	0.4893	-0.114	-0.447	0.218	-0.033	-0.129	0.063	0.5010
2a	Belief (cat)	Yes	Anx (cont)	-0.010	-0.322	0.302	-0.003	-0.093	0.087	0.9500	-0.187	-0.568	0.194	-0.054	-0.163	0.056	0.3364
2b	Identity (cat)	Christian	Anx (cont)	-0.244	-0.569	0.082	-0.070	-0.164	0.024	0.1422	-0.181	-0.549	0.187	-0.052	-0.158	0.054	0.3355
2b	Identity (cat)	Other	Anx (cont)	0.279	-0.489	1.048	0.080	-0.141	0.301	0.4762	-0.214	-0.931	0.504	-0.061	-0.268	0.145	0.5596
2c	Attend (cat)	1/yr	Anx (cont)	-0.233	-0.491	0.026	-0.067	-0.141	0.008	0.0781	-0.145	-0.405	0.114	-0.042	-0.117	0.033	0.2726
2c	Attend (cat)	1/mth	Anx (cont)	-0.120	-0.489	0.249	-0.035	-0.141	0.072	0.5226	-0.344	-0.720	0.032	-0.099	-0.207	0.009	0.0729
2c	Attend (cat)	1/wk	Anx (cont)	-0.292	-0.661	0.077	-0.084	-0.190	0.022	0.1205	-0.373	-0.816	0.069	-0.107	-0.235	0.020	0.0985
3a	Belief (bin)	Yes	Dep (cont)	-0.247	-0.558	0.064	-0.050	-0.113	0.013	0.1192	-0.32	-0.696	0.056	-0.065	-0.142	0.011	0.0950

Model num.	RSBB expo-sure	RSBB expo-sure level	Out-come	Est (no adj)	LCI (no adj)	UCI (no adj)	Est (no adj; std)	LCI (no adj; std)	UCI (no adj; std)	*p* (no adj)	Est (adj)	LCI (adj)	UCI (adj)	Est (adj; std)	LCI (adj; std)	UCI (adj; std)	*p* (adj)
3b	Identity (bin)	Religious	Dep (cont)	-0.224	-0.684	0.236	-0.045	-0.139	0.048	0.3399	-0.474	-0.995	0.048	-0.096	-0.202	0.010	0.0751
3c	Attend (bin)	Regular	Dep (cont)	-0.146	-0.524	0.233	-0.03	-0.107	0.047	0.4510	-0.371	-0.801	0.059	-0.075	-0.163	0.012	0.0908
4a	Belief (bin)	Yes	Anx (cont)	-0.088	-0.308	0.131	-0.025	-0.089	0.038	0.4313	-0.092	-0.354	0.170	-0.026	-0.102	0.049	0.4917
4b	Identity (bin)	Religious	Anx (cont)	-0.229	-0.554	0.096	-0.066	-0.159	0.028	0.1668	-0.184	-0.547	0.180	-0.053	-0.157	0.052	0.3213
4c	Attend (bin)	Regular	Anx (cont)	-0.124	-0.391	0.144	-0.036	-0.113	0.041	0.3641	-0.271	-0.571	0.028	-0.078	-0.164	0.008	0.0761
5a	Belief (cat)	Not sure	Dep (bin)	1.038	0.784	1.384	-	-	-	0.7974	0.824	0.583	1.172	-	-	-	0.2772
5a	Belief (cat)	Yes	Dep (bin)	0.796	0.605	1.055	-	-	-	0.1074	0.651	0.435	0.978	-	-	-	0.0375
5b	Identity (cat)	Christian	Dep (bin)	0.783	0.599	1.038	-	-	-	0.0817	0.673	0.464	0.984	-	-	-	0.0388
5b	Identity (cat)	Other	Dep (bin)	0.925	0.459	1.726	-	-	-	0.8157	0.527	0.234	1.112	-	-	-	0.1052
5c	Attend (cat)	1/yr	Dep (bin)	0.818	0.644	1.032	-	-	-	0.0941	0.894	0.671	1.188	-	-	-	0.4430
5c	Attend (cat)	1/mth	Dep (bin)	0.863	0.611	1.195	-	-	-	0.3884	0.804	0.528	1.208	-	-	-	0.3020
5c	Attend (cat)	1/wk	Dep (bin)	0.678	0.465	0.962	-	-	-	0.0357	0.776	0.466	1.267	-	-	-	0.3198
6a	Belief (cat)	Not sure	Anx (bin)	0.888	0.694	1.142	-	-	-	0.3511	0.714	0.521	0.981	-	-	-	0.0368
6a	Belief (cat)	Yes	Anx (bin)	0.751	0.592	0.957	-	-	-	0.0192	0.650	0.450	0.940	-	-	-	0.0214
6b	Identity (cat)	Christian	Anx (bin)	0.766	0.604	0.982	-	-	-	0.0318	0.760	0.541	1.074	-	-	-	0.1167
6b	Identity (cat)	Other	Anx (bin)	0.834	0.445	1.476	-	-	-	0.5505	0.557	0.264	1.118	-	-	-	0.1109
6c	Attend (cat)	1/yr	Anx (bin)	0.725	0.586	0.893	-	-	-	0.0028	0.851	0.655	1.103	-	-	-	0.2256

**Model num.**	**RSBB expo-sure**	**RSBB expo-sure level**	**Out-come**	**Est (no adj)**	**LCI (no adj)**	**UCI (no adj)**	**Est (no adj; std)**	**LCI (no adj; std)**	**UCI (no adj; std)**	***p* (no adj)**	**Est (adj)**	**LCI (adj)**	**UCI (adj)**	**Est (adj; std)**	**LCI (adj; std)**	**UCI (adj; std)**	***p* (adj)**
6c	Attend (cat)	1/mth	Anx (bin)	0.834	0.617	1.111	-	-	-	0.2252	0.828	0.565	1.202	-	-	-	0.327
6c	Attend (cat)	1/wk	Anx (bin)	0.680	0.492	0.922	-	-	-	0.0157	0.713	0.448	1.119	-	-	-	0.1470
7a	Belief (bin)	Yes	Dep (bin)	0.776	0.636	0.947	-	-	-	0.0127	0.764	0.575	1.015	-	-	-	0.0636
7b	Identity (bin)	Religious	Dep (bin)	0.787	0.602	1.042	-	-	-	0.0872	0.660	0.458	0.96	-	-	-	0.0279
7c	Attend (bin)	Regular	Dep (bin)	0.823	0.636	1.054	-	-	-	0.1305	0.845	0.602	1.173	-	-	-	0.3209
8a	Belief (bin)	Yes	Anx (bin)	0.813	0.682	0.968	-	-	-	0.0203	0.856	0.658	1.113	-	-	-	0.2457
8b	Identity (bin)	Religious	Anx (bin)	0.768	0.605	0.983	-	-	-	0.0330	0.742	0.530	1.042	-	-	-	0.0825
8c	Attend (bin)	Regular	Anx (bin)	0.840	0.672	1.044	-	-	-	0.1213	0.855	0.627	1.159	-	-	-	0.3186

Table S9: Predicted probabilities of the mothers logistic analyses with religious/spiritual belief and behaviour (RSBB) exposures and binary probable depression and anxiety as outcomes (n = 3,856). These results indicate the difference in the predicted probability of a probable depression or anxiety diagnosis, based on the associated logistic regression model (see table S8). LCI = Lower 95% confidence interval; UCI = Upper 95% confidence interval; no adj = unadjusted model; adj = Adjusted model.

**Model num.**	**RSBB expo-sure**	**RSBB expo-sure level**	**Out-come**	**Diff in pred prob (no adj)**	**LCI (no adj)**	**UCI (no adj)**	**Diff in pred prob (adj)**	**LCI (adj)**	**UCI (adj)**
5a	Belief (cat)	Not sure	Dep (bin)	0.41	-2.72	3.55	-1.95	-5.56	1.67
5a	Belief (cat)	Yes	Dep (bin)	-2.29	-5.20	0.62	-4.03	-8.04	-0.01
5b	Identity (cat)	Christian	Dep (bin)	-2.66	-5.85	0.53	-3.92	-7.96	0.12
5b	Identity (cat)	Other	Dep (bin)	-0.90	-8.35	6.54	-5.90	-12.22	0.41
5c	Attend (cat)	1/yr	Dep (bin)	-2.06	-4.42	0.30	-1.02	-3.62	1.57
5c	Attend (cat)	1/mth	Dep (bin)	-1.54	-4.91	1.83	-1.93	-5.45	1.60
5c	Attend (cat)	1/wk	Dep (bin)	-3.72	-6.84	-0.60	-2.22	-6.37	1.93
6a	Belief (cat)	Not sure	Anx (bin)	-1.68	-5.26	1.90	-3.99	-7.88	-0.09
6a	Belief (cat)	Yes	Anx (bin)	-3.85	-7.22	-0.48	-4.99	-9.42	-0.56
6b	Identity (cat)	Christian	Anx (bin)	-3.72	-7.33	-0.11	-3.16	-7.31	0.99
6b	Identity (cat)	Other	Anx (bin)	-2.61	-10.82	5.60	-6.23	-13.13	0.68
6c	Attend (cat)	1/yr	Anx (bin)	-4.17	-6.81	-1.53	-1.78	-4.63	1.07
6c	Attend (cat)	1/mth	Anx (bin)	-2.47	-6.31	1.36	-2.07	-6.08	1.95
6c	Attend (cat)	1/wk	Anx (bin)	-4.89	-8.51	-1.28	-3.56	-8.09	0.97
7a	Belief (bin)	Yes	Dep (bin)	-2.57	-4.58	-0.56	-2.42	-4.97	0.13
7b	Identity (bin)	Religious	Dep (bin)	-2.61	-5.80	0.58	-4.12	-8.14	-0.10
7c	Attend (bin)	Regular	Dep (bin)	-1.90	-4.25	0.45	-1.48	-4.30	1.35
8a	Belief (bin)	Yes	Anx (bin)	-2.71	-4.99	-0.43	-1.70	-4.55	1.16
8b	Identity (bin)	Religious	Anx (bin)	-3.69	-7.29	-0.08	-3.46	-7.58	0.66
8c	Attend (bin)	Regular	Anx (bin)	-2.21	-4.90	0.49	-1.67	-4.86	1.53

Table S10: Descriptive statistics for religious/spiritual beliefs and behaviour (RSBB) exposures 5 years post-delivery and mental health outcomes 6 years post-delivery for partners (*n* = 1,940). EPDS = Edinburgh Postnatal Depression Scale; CCEI-A = Crown-Crisp Experiential Inventory – Anxiety subscale; SD = Standard deviation.

	**EPDS depression scores (Mean = 4.27 [SD = 4.27])**	**Probable depression diagnosis (*n* = 258 [13.3%])**	**CCEI-A anxiety scores (Mean = 3.67 [SD = 2.79])**	**Probable anxiety diagnosis (*n* = 416 [21.4%])**
	Mean (SD)	*n (%)*	*Mean (SD)*	*n (%)*
**Religious Belief (categorical)**				
No (n = 541 [27.9%])	3.94 (4.21)	65 (12.0%)	3.41 (2.69)	92 (17.0%)
Not Sure (n = 715 [36.9%])	4.18 (4.23)	84 (11.8%)	3.59 (2.76)	151 (21.1%)
Yes (n = 684 [35.3%])	4.63 (4.35)	109 (12.9%)	3.96 (2.86)	173 (25.3%)
**Religious Belief (binary)**				
No/Not Sure (n = 1,256 [64.7%])	4.07 (4.22)	149 (11.9%)	3.52 (2.73)	243 (19.4%)
Yes (n = 684 [35.3%])	4.63 (4.35)	109 (15.9%)	3.96 (2.86)	173 (25.3%)
**Religious Affiliation (categorical)**				
None (n = 429 [22.1%])	3.88 (4.08)	46 (10.7%)	3.48 (2.63)	79 (18.4%)
Christian (n = 1,469 [75.7%])	4.34 (4.29)	204 (13.9%)	3.69 (2.78)	320 (21.8%)
Other religion (n = 42 [2.2%])	5.83 (5.09)	8 (19.1%)	5.24 (3.88)	17 (40.5%)
**Religious Affiliation (binary)**				
None (n = 429 [22.1%])	3.88 (4.08)	46 (10.7%)	3.48 (2.63)	79 (18.4%)
Religious (n = 1,511 [77.9%])	4.38 (4.32)	212 (14.0%)	3.73 (2.83)	337 (22.3%)
**Religious Attendance (categorical)**				
Not at all (n = 1,153 [59.4%])	4.24 (4.34)	155 (13.4%)	3.64 (2.81)	242 (21.0%)
Min. 1/year (n = 462 [23.8%])	4.11 (4.22)	59 (12.8%)	3.69 (2.82)	103 (22.3%)
Min. 1/month (n = 143 [7.4%])	4.90 (4.37)	20 (14.0%)	3.81 (2.61)	33 (23.1%)
Min. 1/week (n = 182 [9.4%])	4.41 (3.84)	24 (13.2%)	3.74 (2.72)	38 (20.9%)
**Religious Attendance (binary)**				
Occasional/Never (n = 1,615 [83.3%])	4.20 (4.31)	214 (13.3%)	3.65 (2.81)	345 (21.4%)
Regular (n = 325 [16.7%])	4.62 (4.08)	44 (13.5%)	3.77 (2.67)	71 (21.9%)

Table S11: Results of the partners analyses with religious/spiritual belief and behaviour (RSBB) exposures and depression and anxiety as outcomes (n = 1,940). Est = Estimate (either mean difference from a linear model, or odds ratio from a logistic model); LCI = Lower 95% confidence interval; UCI = Upper 95% confidence interval; p = p-value; no adj = unadjusted model; adj = Adjusted model; std = model using standardised depression or anxiety scores.

Model num.	RSBB expo-sure	RSBB expo-sure level	Out-come	Est (no adj)	LCI (no adj)	UCI (no adj)	Est (no adj; std)	LCI (no adj; std)	UCI (no adj; std)	*p* (no adj)	Est (adj)	LCI (adj)	UCI (adj)	Est (adj; std)	LCI (adj; std)	UCI (adj; std)	*p* (adj)
1a	Belief (cat)	Not sure	Dep (cont)	0.244	-0.233	0.720	0.057	-0.055	0.169	0.3160	0.025	-0.495	0.546	0.006	-0.116	0.128	0.9236
1a	Belief (cat)	Yes	Dep (cont)	0.695	0.214	1.176	0.163	0.050	0.275	0.0047	0.188	-0.463	0.840	0.044	-0.108	0.197	0.5713
1b	Identity (cat)	Christian	Dep (cont)	0.461	0.002	0.920	0.108	0.000	0.215	0.0490	0.184	-0.348	0.717	0.043	-0.082	0.168	0.4973
1b	Identity (cat)	Other	Dep (cont)	1.955	0.603	3.306	0.458	0.141	0.774	0.0046	0.959	-0.321	2.238	0.224	-0.075	0.524	0.1419
1c	Attend (cat)	1/yr	Dep (cont)	-0.130	-0.591	0.331	-0.030	-0.138	0.078	0.5809	-0.453	-0.903	-0.003	-0.106	-0.211	-0.001	0.0487
1c	Attend (cat)	1/mth	Dep (cont)	0.659	-0.083	1.402	0.154	-0.020	0.328	0.0818	0.006	-0.735	0.747	0.001	-0.172	0.175	0.9877
1c	Attend (cat)	1/wk	Dep (cont)	0.176	-0.492	0.844	0.041	-0.115	0.198	0.6051	-0.098	-1.071	0.875	-0.023	-0.251	0.205	0.8439
2a	Belief (cat)	Not sure	Anx (cont)	0.182	-0.128	0.493	0.065	-0.046	0.177	0.2500	0.126	-0.207	0.459	0.045	-0.074	0.165	0.4589
2a	Belief (cat)	Yes	Anx (cont)	0.551	0.238	0.865	0.198	0.085	0.310	0.0006	0.413	-0.005	0.830	0.148	-0.002	0.298	0.0526
2b	Identity (cat)	Christian	Anx (cont)	0.211	-0.088	0.510	0.076	-0.031	0.183	0.1657	0.203	-0.138	0.545	0.073	-0.050	0.195	0.2433
2b	Identity (cat)	Other	Anx (cont)	1.763	0.882	2.643	0.633	0.317	0.949	0.0001	0.925	0.105	1.744	0.332	0.038	0.626	0.0270
2c	Attend (cat)	1/yr	Anx (cont)	0.045	-0.256	0.346	0.016	-0.092	0.124	0.7684	-0.212	-0.501	0.077	-0.076	-0.180	0.028	0.1505
2c	Attend (cat)	1/mth	Anx (cont)	0.170	-0.315	0.655	0.061	-0.113	0.235	0.4911	-0.165	-0.640	0.311	-0.059	-0.230	0.112	0.4971
2c	Attend (cat)	1/wk	Anx (cont)	0.101	-0.335	0.537	0.036	-0.120	0.193	0.6504	-0.067	-0.691	0.557	-0.024	-0.248	0.200	0.8331
3a	Belief (bin)	Yes	Dep (cont)	0.556	0.159	0.954	0.130	0.037	0.223	0.0061	0.167	-0.320	0.654	0.039	-0.075	0.153	0.5013

Model num.	RSBB expo-sure	RSBB expo-sure level	Out-come	Est (no adj)	LCI (no adj)	UCI (no adj)	Est (no adj; std)	LCI (no adj; std)	UCI (no adj; std)	*p* (no adj)	Est (adj)	LCI (adj)	UCI (adj)	Est (adj; std)	LCI (adj; std)	UCI (adj; std)	*p* (adj)
3b	Identity (bin)	Religious	Dep (cont)	0.502	0.045	0.960	0.118	0.010	0.225	0.0315	0.237	-0.289	0.763	0.055	-0.068	0.179	0.3775
3c	Attend (bin)	Regular	Dep (cont)	0.426	-0.083	0.935	0.100	-0.019	0.219	0.1011	0.209	-0.426	0.845	0.049	-0.100	0.198	0.5184
4a	Belief (bin)	Yes	Anx (cont)	0.448	0.189	0.707	0.161	0.068	0.254	0.0007	0.308	-0.004	0.620	0.110	-0.001	0.222	0.0529
4b	Identity (bin)	Religious	Anx (cont)	0.254	-0.044	0.553	0.091	-0.016	0.199	0.0951	0.252	-0.085	0.589	0.090	-0.031	0.211	0.1430
4c	Attend (bin)	Regular	Anx (cont)	0.118	-0.214	0.451	0.043	-0.077	0.162	0.4846	-0.025	-0.433	0.382	-0.009	-0.155	0.137	0.9029
5a	Belief (cat)	Not sure	Dep (bin)	0.975	0.691	1.380	-	-	-	0.8850	0.774	0.471	1.275	-	-	-	0.3123
5a	Belief (cat)	Yes	Dep (bin)	1.388	1.001	1.939	-	-	-	0.0516	0.985	0.548	1.780	-	-	-	0.9586
5b	Identity (cat)	Christian	Dep (bin)	1.343	0.964	1.905	-	-	-	0.0891	1.019	0.619	1.689	-	-	-	0.9422
5b	Identity (cat)	Other	Dep (bin)	1.959	0.803	4.302	-	-	-	0.1117	0.905	0.296	2.520	-	-	-	0.8538
5c	Attend (cat)	1/yr	Dep (bin)	0.943	0.679	1.293	-	-	-	0.7186	0.766	0.508	1.145	-	-	-	0.1984
5c	Attend (cat)	1/mth	Dep (bin)	1.047	0.617	1.694	-	-	-	0.8578	0.738	0.383	1.370	-	-	-	0.3479
5c	Attend (cat)	1/wk	Dep (bin)	0.978	0.604	1.525	-	-	-	0.9248	0.881	0.366	2.010	-	-	-	0.7697
6a	Belief (cat)	Not sure	Anx (bin)	1.307	0.982	1.746	-	-	-	0.0681	1.570	1.018	2.437	-	-	-	0.0425
6a	Belief (cat)	Yes	Anx (bin)	1.652	1.248	2.199	-	-	-	0.0005	1.926	1.142	3.272	-	-	-	0.0146
6b	Identity (cat)	Christian	Anx (bin)	1.234	0.943	1.631	-	-	-	0.1324	1.415	0.919	2.192	-	-	-	0.1170
6b	Identity (cat)	Other	Anx (bin)	3.013	1.531	5.812	-	-	-	0.0011	2.173	0.912	5.108	-	-	-	0.0764
6c	Attend (cat)	1/yr	Anx (bin)	1.080	0.830	1.399	-	-	-	0.5630	0.839	0.589	1.189	-	-	-	0.3265

Model num.	RSBB expo-sure	RSBB expo-sure level	Out-come	Est (no adj)	LCI (no adj)	UCI (no adj)	Est (no adj; std)	LCI (no adj; std)	UCI (no adj; std)	*p* (no adj)	Est (adj)	LCI (adj)	UCI (adj)	Est (adj; std)	LCI (adj; std)	UCI (adj; std)	*p* (adj)
6c	Attend (cat)	1/mth	Anx (bin)	1.129	0.737	1.691	-	-	-	0.5648	0.783	0.446	1.350	-	-	-	0.3856
6c	Attend (cat)	1/wk	Anx (bin)	0.993	0.669	1.445	-	-	-	0.9731	0.677	0.322	1.381	-	-	-	0.2922
7a	Belief (bin)	Yes	Dep (bin)	1.408	1.077	1.837	-	-	-	0.0119	1.224	0.809	1.850	-	-	-	0.3381
7b	Identity (bin)	Religious	Dep (bin)	1.359	0.977	1.926	-	-	-	0.0759	1.009	0.617	1.662	-	-	-	0.9713
7c	Attend (bin)	Regular	Dep (bin)	1.025	0.716	1.440	-	-	-	0.8892	0.894	0.516	1.509	-	-	-	0.6816
8a	Belief (bin)	Yes	Anx (bin)	1.411	1.129	1.761	-	-	-	0.0024	1.307	0.907	1.885	-	-	-	0.1507
8b	Identity (bin)	Religious	Anx (bin)	1.272	0.973	1.679	-	-	-	0.0838	1.474	0.964	2.267	-	-	-	0.0750
8c	Attend (bin)	Regular	Anx (bin)	1.029	0.767	1.367	-	-	-	0.8462	0.821	0.510	1.305	-	-	-	0.4114

Table S12: Predicted probabilities of the partners logistic analyses with religious/spiritual belief and behaviour (RSBB) exposures and binary probable depression and anxiety as outcomes (*n* = 1,940). These results indicate the difference in the predicted probability of a probable depression or anxiety diagnosis, based on the associated logistic regression model (see table S11). LCI = Lower 95% confidence interval; UCI = Upper 95% confidence interval; no adj = unadjusted model; adj = Adjusted model.

**Model num.**	**RSBB expo-sure**	**RSBB expo-sure level**	**Out-come**	**Diff in pred prob (no adj)**	**LCI (no adj)**	**UCI (no adj)**	**Diff in pred prob (adj)**	**LCI (adj)**	**UCI (adj)**
5a	Belief (cat)	Not sure	Dep (bin)	-0.27	-3.88	3.35	-2.33	-6.97	2.31
5a	Belief (cat)	Yes	Dep (bin)	3.92	0.04	7.80	-0.15	-5.87	5.57
5b	Identity (cat)	Christian	Dep (bin)	3.16	-0.26	6.58	0.17	-4.45	4.79
5b	Identity (cat)	Other	Dep (bin)	8.33	-3.91	20.56	-0.90	-10.22	8.43
5c	Attend (cat)	1/yr	Dep (bin)	-0.67	-4.30	2.95	-2.43	-6.04	1.19
5c	Attend (cat)	1/mth	Dep (bin)	0.54	-5.47	6.56	-2.74	-8.16	2.67
5c	Attend (cat)	1/wk	Dep (bin)	-0.26	-5.55	5.04	-1.20	-9.04	6.64
6a	Belief (cat)	Not sure	Anx (bin)	4.11	-0.24	8.47	5.20	0.34	10.05
6a	Belief (cat)	Yes	Anx (bin)	8.29	3.74	12.83	7.88	1.70	14.06
6b	Identity (cat)	Christian	Anx (bin)	3.37	-0.86	7.60	4.13	-0.83	9.09
6b	Identity (cat)	Other	Anx (bin)	22.06	6.77	37.35	10.04	-2.25	22.34
6c	Attend (cat)	1/yr	Anx (bin)	1.31	-3.16	5.77	-2.19	-6.50	2.13
6c	Attend (cat)	1/mth	Anx (bin)	2.09	-5.21	9.38	-3.01	-9.59	3.57
6c	Attend (cat)	1/wk	Anx (bin)	-0.11	-6.47	6.25	-4.66	-12.85	3.53
7a	Belief (bin)	Yes	Dep (bin)	4.07	0.80	7.35	1.89	-2.04	5.83
7b	Identity (bin)	Religious	Dep (bin)	3.31	-0.10	6.72	0.08	-4.48	4.65
7c	Attend (bin)	Regular	Dep (bin)	0.29	-3.78	4.36	-1.02	-5.77	3.74
8a	Belief (bin)	Yes	Anx (bin)	5.95	2.02	9.87	3.37	-1.29	8.03
8b	Identity (bin)	Religious	Anx (bin)	3.89	-0.34	8.11	4.61	-0.24	9.46
8c	Attend (bin)	Regular	Anx (bin)	0.48	-4.43	5.40	-2.38	-7.91	3.15

Table S13: Results of the interaction analyses assessing whether the adjusted mother and partner results differ, with religious/spiritual belief and behaviour (RSBB) exposures and standardised depression and anxiety scores as outcomes. SE = Standard error; LCI = Lower 95% confidence interval; UCI = Upper 95% confidence interval; *p* = *p*-value.

Model num.	Exposure	Exposure level	Outcome	Mother coef.	Mother SE	Partner coef.	Partner SE	Mother vs partner diff.	Diff. SE	Diff. LCI	Diff. UCI	Diff. *p*
1a	Belief (cat)	Not sure	Dep (cont)	0.018	0.049	0.006	0.062	0.012	0.080	-0.144	0.168	0.8801
1a	Belief (cat)	Yes	Dep (cont)	-0.050	0.057	0.044	0.078	-0.094	0.096	-0.283	0.095	0.3287
1b	Identity (cat)	Christian	Dep (cont)	-0.086	0.055	0.043	0.064	-0.129	0.084	-0.294	0.036	0.1251
1b	Identity (cat)	Other	Dep (cont)	-0.205	0.107	0.224	0.153	-0.429	0.186	-0.795	-0.063	0.0214
1c	Attend (cat)	1/yr	Dep (cont)	-0.048	0.039	-0.106	0.054	0.058	0.066	-0.071	0.187	0.3794
1c	Attend (cat)	1/mth	Dep (cont)	-0.108	0.056	0.001	0.089	-0.109	0.105	-0.314	0.096	0.2984
1c	Attend (cat)	1/wk	Dep (cont)	-0.094	0.066	-0.023	0.116	-0.071	0.134	-0.333	0.191	0.5953
2a	Belief (cat)	Not sure	Anx (cont)	-0.033	0.049	0.045	0.061	-0.078	0.078	-0.231	0.075	0.3186
2a	Belief (cat)	Yes	Anx (cont)	-0.054	0.056	0.148	0.077	-0.202	0.095	-0.388	-0.016	0.0330
2b	Identity (cat)	Christian	Anx (cont)	-0.052	0.054	0.073	0.063	-0.125	0.083	-0.287	0.037	0.1304
2b	Identity (cat)	Other	Anx (cont)	-0.061	0.105	0.332	0.150	-0.393	0.183	-0.752	-0.034	0.0320
2c	Attend (cat)	1/yr	Anx (cont)	-0.042	0.038	-0.076	0.053	0.034	0.065	-0.094	0.162	0.6033
2c	Attend (cat)	1/mth	Anx (cont)	-0.099	0.055	-0.059	0.087	-0.040	0.103	-0.242	0.162	0.6983
2c	Attend (cat)	1/wk	Anx (cont)	-0.107	0.065	-0.024	0.114	-0.083	0.132	-0.341	0.175	0.5279
3a	Belief (bin)	Yes	Dep (cont)	-0.065	0.039	0.039	0.058	-0.104	0.070	-0.241	0.033	0.1376
3b	Identity (bin)	Religious	Dep (cont)	-0.096	0.054	0.055	0.063	-0.151	0.083	-0.314	0.012	0.0690
3c	Attend (bin)	Regular	Dep (cont)	-0.075	0.045	0.049	0.076	-0.124	0.088	-0.297	0.049	0.1596
4a	Belief (bin)	Yes	Anx (cont)	-0.026	0.039	0.110	0.057	-0.136	0.069	-0.271	-0.001	0.0478
4b	Identity (bin)	Religious	Anx (cont)	-0.053	0.053	0.090	0.062	-0.143	0.082	-0.303	0.017	0.0796
4c	Attend (bin)	Regular	Anx (cont)	-0.078	0.044	-0.009	0.074	-0.069	0.086	-0.238	0.100	0.4248

Table S14: Results of the interaction analyses assessing whether the adjusted mother and partner results differ, with religious/spiritual belief and behaviour (RSBB) exposures and probable depression and anxiety diagnoses as outcomes. OR = Odds ratio; SE = Standard error; LCI = Lower 95% confidence interval; UCI = Upper 95% confidence interval; *p* = *p*-value.

**Model num.**	**Exposure**	**Expo-sure level**	**Outcome**	**Mother OR**	**Mother log-odds**	**Mother log-odds SE**	**Partner OR**	**Partner log-odds**	**Partner log-odds SE**	**Log-odds diff.**	**Diff. log-odds SE**	**Diff. OR**	**Diff. OR LCI**	**Diff. OR UCI**	**Diff. *p***
5a	Belief (cat)	Not sure	Dep (bin)	0.824	-0.194	0.178	0.774	-0.256	0.254	0.063	0.310	1.065	0.580	1.956	0.8401
5a	Belief (cat)	Yes	Dep (bin)	0.651	-0.429	0.207	0.985	-0.015	0.301	-0.414	0.365	0.661	0.323	1.351	0.2562
5b	Identity (cat)	Christian	Dep (bin)	0.673	-0.396	0.192	1.019	0.019	0.256	-0.415	0.320	0.660	0.353	1.236	0.1947
5b	Identity (cat)	Other	Dep (bin)	0.527	-0.641	0.398	0.905	-0.100	0.546	-0.541	0.676	0.582	0.155	2.189	0.4236
5c	Attend (cat)	1/yr	Dep (bin)	0.894	-0.112	0.146	0.766	-0.267	0.207	0.155	0.253	1.167	0.710	1.918	0.5420
5c	Attend (cat)	1/mth	Dep (bin)	0.804	-0.218	0.211	0.738	-0.304	0.325	0.086	0.388	1.089	0.510	2.329	0.8251
5c	Attend (cat)	1/wk	Dep (bin)	0.776	-0.254	0.255	0.881	-0.127	0.435	-0.127	0.504	0.881	0.328	2.365	0.8012
6a	Belief (cat)	Not sure	Anx (bin)	0.714	-0.337	0.161	1.570	0.451	0.223	-0.788	0.275	0.455	0.265	0.780	0.0042
6a	Belief (cat)	Yes	Anx (bin)	0.650	-0.431	0.188	1.926	0.655	0.269	-1.086	0.328	0.337	0.178	0.642	0.0009
6b	Identity (cat)	Christian	Anx (bin)	0.760	-0.274	0.175	1.415	0.347	0.222	-0.622	0.282	0.537	0.309	0.934	0.0278
6b	Identity (cat)	Other	Anx (bin)	0.557	-0.585	0.368	2.173	0.776	0.440	-1.361	0.573	0.256	0.083	0.789	0.0176
6c	Attend (cat)	1/yr	Anx (bin)	0.851	-0.161	0.133	0.839	-0.176	0.179	0.014	0.223	1.014	0.655	1.571	0.9493
6c	Attend (cat)	1/mth	Anx (bin)	0.828	-0.189	0.193	0.783	-0.245	0.283	0.056	0.342	1.057	0.541	2.067	0.8702
2c	Attend (cat)	1/wk	Anx (bin)	0.713	-0.338	0.234	0.677	-0.390	0.371	0.052	0.439	1.053	0.446	2.489	0.9060
7a	Belief (bin)	Yes	Dep (bin)	0.764	-0.269	0.145	1.224	0.202	0.211	-0.471	0.256	0.624	0.378	1.031	0.0656
7b	Identity (bin)	Religious	Dep (bin)	0.660	-0.416	0.189	1.009	0.009	0.253	-0.424	0.316	0.654	0.352	1.214	0.1785
7c	Attend (bin)	Regular	Dep (bin)	0.845	-0.168	0.170	0.894	-0.112	0.274	-0.056	0.322	0.945	0.503	1.778	0.8612
8a	Belief (bin)	Yes	Anx (bin)	0.856	-0.155	0.134	1.307	0.268	0.187	-0.423	0.230	0.655	0.417	1.028	0.0655
8b	Identity (bin)	Religious	Anx (bin)	0.742	-0.298	0.172	1.474	0.388	0.218	-0.686	0.278	0.503	0.292	0.868	0.0136
8c	Attend (bin)	Regular	Anx (bin)	0.855	-0.157	0.157	0.821	-0.197	0.240	0.041	0.286	1.041	0.594	1.826	0.8873

Table S15: Descriptive statistics for mental health exposures 2 years post-delivery and religious/spiritual beliefs and behaviour (RSBB) outcomes 5 years post-delivery for mothers (*n* = 4,025). EPDS = Edinburgh Postnatal Depression Scale; CCEI-A = Crown-Crisp Experiential Inventory – Anxiety subscale; SD = Standard deviation.

	**Religious Belief (categorical)**			**Religious Belief (binary)**		**Religious Affiliation (categorical)**			**Religious Affiliation (binary)**		**Religious Attendance (categorical)**				**Religious Attendance (binary)**	
	No (n = *673 [16.7%])*	*Not Sure* (n *= 1,387 [34.5%])*	*Yes* (n = *1,965 [48.8%])*	*No/Not Sure* (n *= 2,060 [51.2%])*	*Yes* (n = *1,965 [48.8%])*	*None* (n *= 535 [13.3%])*	*Christian* (n = *3,394 [84.3%])*	*Other* (n = *96 [2.4%])*	*None* (n *= 535 [13.3%])*	*Religious* (n = *3,490 [86.7%])*	*Not at all* (n = *2,050 [50.9%])*	*Min. 1/ yr* (n = *1,119 [27.8%])*	*Min. 1/ mth* (n = *431 [10.7%])*	*Min. 1/ wk* (n = *425 [10.6%])*	*Occ./ Never* (n = *3,169 [78.7%])*	*Regular* (n = *856 [21.3%])*
**EPDS depression scores (Mean = 5.39 [SD = 4.60])**																
Mean (SD)	5.56 (4.88)	5.47 (4.63)	5.27 (4.48)	5.50 (4.71)	5.27 (4.48)	5.68 (5.00)	5.32 (4.53)	6.09 (4.69)	5.68 (5.00)	5.34 (4.54)	5.62 (4.79)	4.89 (4.21)	5.40 (4.52)	5.56 (4.69)	5.36 (4.60)	5.48 (4.60)
**Probable depression diagnosis (*n* = 343 [8.5%])**																
No	603 (16.4%)	1,263 (34.3%)	1,816 (49.3%)	1,866 (50.7%)	1,816 (49.3%)	472 (12.8%)	3,125 (84.9%)	85 (2.3%)	472 (12.8%)	3,210 (87.2%)	1,844 (50.1%)	1,059 (28.8%)	393 (10.7%)	386 (10.5%)	2,903 (78.8%)	779 (21.2%)
Yes	70 (20.4%)	124 (36.2%)	149 (43.4%)	194 (56.6%)	149 (43.4%)	63 (18.4%)	269 (78.4%)	11 (3.2%)	63 (18.4%)	280 (81.6%)	206 (60.1%)	60 (17.5%)	38 (11.1%)	39 (11.4%)	266 (77.6%)	77 (22.4%)
**CCEI-A anxiety scores (Mean = 3.61 [SD = 3.19])**																
Mean (SD)	3.71 (3.40)	3.64 (3.19)	3.55 (3.12)	3.66 (3.26)	3.55 (3.12)	3.97 (3.42)	3.54 (3.15)	4.07 (3.24)	3.97 (3.42)	3.55 (3.15)	3,72 (3.30)	3.38 (2.89)	3.63 (3.19)	3.68 (3.38)	3.60 (3.16)	3.65 (3.28)
**Probable anxiety diagnosis (*n* = 348 [8.7%])**																
No	608 (16.5%)	1,266 (34.4%)	1,803 (49.0%)	1,874 (51.0%)	1,803 (49.0%)	476 (13.0%)	3,115 (84.7%)	86 (2.3%)	476 (13.0%)	3,201 (87.0%)	1,857 (50.5%)	1,042 (28.3%)	395 (10.7%)	383 (10.4%)	2,899 (78.8%)	778 (21.2%)
Yes	65 (18.7%)	121 (34.8%)	162 (46.5%)	186 (53.5%)	162 (46.5%)	59 (16.9%)	279 (80.2%)	10 (2.9%)	59 (16.9%)	289 (83.1%)	193 (55.5%)	77 (22.1%)	36 (10.3%)	42 (12.1%)	270 (77.6%)	78 (22.4%)

Table S16: Results of the mothers analyses with depression and anxiety exposures and religious/spiritual belief and behaviour (RSBB) as outcomes (n = 4,025). Est = Estimate (either relative risk ratio from a multinomial model, or odds ratio from a logistic model); LCI = Lower 95% confidence interval; UCI = Upper 95% confidence interval; p = p-value; no adj = unadjusted model; adj = Adjusted model; std = model using standardised depression or anxiety scores.

**Model num.**	**Mental health expo-sure**	**RSBB out-come**	**RSBB out-come level**	**Est (no adj)**	**LCI (no adj)**	**UCI (no adj)**	**Est (no adj; std)**	**LCI (no adj; std)**	**UCI (no adj; std)**	***p* (no adj)**	**Est (adj)**	**LCI (adj)**	**UCI (adj)**	**Est (adj; std)**	**LCI (adj; std)**	**UCI (adj; std)**	***p* (adj)**
1a	Dep (cont)	Belief (cat)	Not sure	0.995	0.976	1.015	0.979	0.895	1.072	0.6521	0.979	0.953	1.006	0.908	0.801	1.030	0.1342
1a	Dep (cont)	Belief (cat)	Yes	0.986	0.968	1.005	0.938	0.860	1.023	0.1460	0.974	0.944	1.005	0.886	0.766	1.025	0.1023
1b	Anx (cont)	Belief (cat)	Not sure	0.994	0.966	1.023	0.981	0.896	1.075	0.6860	0.974	0.936	1.014	0.920	0.809	1.045	0.1979
1b	Anx (cont)	Belief (cat)	Yes	0.985	0.959	1.012	0.954	0.875	1.040	0.2837	0.979	0.935	1.026	0.935	0.807	1.084	0.3735
2a	Dep (cont)	Identity (cat)	Christian	0.984	0.965	1.003	0.927	0.848	1.013	0.0949	0.982	0.952	1.012	0.919	0.798	1.058	0.2399
2a	Dep (cont)	Identity (cat)	Other	1.018	0.974	1.063	1.084	0.886	1.327	0.4326	0.972	0.914	1.035	0.879	0.660	1.171	0.3783
2b	Anx (cont)	Identity (cat)	Christian	0.960	0.935	0.987	0.879	0.806	0.958	0.0034	0.966	0.925	1.010	0.896	0.779	1.032	0.1288
2b	Anx (cont)	Identity (cat)	Other	1.008	0.947	1.074	1.027	0.841	1.254	0.7942	0.975	0.890	1.069	0.924	0.690	1.235	0.5924
3a	Dep (cont)	Attend (cat)	1/yr	0.965	0.949	0.981	0.848	0.786	0.915	<0.0001	0.964	0.942	0.986	0.845	0.760	0.939	0.0018
3a	Dep (cont)	Attend (cat)	1/mth	0.990	0.968	1.012	0.954	0.860	1.058	0.3749	0.978	0.946	1.012	0.903	0.774	1.052	0.1995
3a	Dep (cont)	Attend (cat)	1/wk	0.997	0.975	1.020	0.987	0.891	1.094	0.8025	1.029	0.990	1.069	1.134	0.950	1.355	0.1530
3b	Anx (cont)	Attend (cat)	1/yr	0.966	0.943	0.989	0.895	0.830	0.965	0.0037	0.964	0.933	0.997	0.890	0.801	0.990	0.0309
3b	Anx (cont)	Attend (cat)	1/mth	0.991	0.959	1.024	0.972	0.876	1.077	0.5848	0.975	0.929	1.024	0.923	0.790	1.077	0.3153
3b	Anx (cont)	Attend (cat)	1/wk	0.996	0.964	1.029	0.987	0.891	1.094	0.8048	1.042	0.985	1.103	1.139	0.950	1.365	0.1526
4a	Dep (bin)	Belief (cat)	Not sure	0.846	0.621	1.152	-	-	-	0.2877	0.893	0.600	1.329	-	-	-	0.5777

Model num.	Mental health expo-sure	RSBB out-come	RSBB out-come level	Est (no adj)	LCI (no adj)	UCI (no adj)	Est (no adj; std)	LCI (no adj; std)	UCI (no adj; std)	*p* (no adj)	Est (adj)	LCI (adj)	UCI (adj)	Est (adj; std)	LCI (adj; std)	UCI (adj; std)	*p* (adj)
4a	Dep (bin)	Belief (cat)	Yes	0.707	0.524	0.953	-	-	-	0.0228	0.853	0.535	1.358	-	-	-	0.5022
4b	Anx (bin)	Belief (cat)	Not sure	0.894	0.651	1.227	-	-	-	0.4879	0.754	0.502	1.132	-	-	-	0.1735
4b	Anx (bin)	Belief (cat)	Yes	0.840	0.621	1.137	-	-	-	0.2594	0.816	0.512	1.302	-	-	-	0.3943
5a	Dep (bin)	Identity (cat)	Christian	0.645	0.482	0.863	-	-	-	0.0031	0.752	0.490	1.153	-	-	-	0.1915
5a	Dep (bin)	Identity (cat)	Other	0.970	0.491	1.915	-	-	-	0.9291	0.542	0.223	1.316	-	-	-	0.1761
5b	Anx (bin)	Identity (cat)	Christian	0.723	0.537	0.972	-	-	-	0.0320	0.684	0.441	1.059	-	-	-	0.0888
5b	Anx (bin)	Identity (cat)	Other	0.938	0.462	1.905	-	-	-	0.8597	0.637	0.255	1.594	-	-	-	0.3351
6a	Dep (bin)	Attend (cat)	1/yr	0.507	0.377	0.683	-	-	-	<0.0001	0.564	0.394	0.810	-	-	-	0.0019
6a	Dep (bin)	Attend (cat)	1/mth	0.866	0.602	1.244	-	-	-	0.4353	0.931	0.579	1.499	-	-	-	0.7698
6a	Dep (bin)	Attend (cat)	1/wk	0.904	0.631	1.296	-	-	-	0.5839	1.364	0.779	2.388	-	-	-	0.2768
6b	Anx (bin)	Attend (cat)	1/yr	0.711	0.540	0.936	-	-	-	0.0150	0.760	0.540	1.070	-	-	-	0.1162
6b	Anx (bin)	Attend (cat)	1/mth	0.877	0.604	1.272	-	-	-	0.4890	0.853	0.526	1.385	-	-	-	0.5208
6b	Anx (bin)	Attend (cat)	1/wk	1.055	0.743	1.499	-	-	-	0.7647	1.488	0.854	2.593	-	-	-	0.1612
7a	Dep (cont)	Belief (bin)	Yes	0.989	0.976	1.003	0.951	0.894	1.012	0.1118	0.991	0.969	1.013	0.959	0.865	1.062	0.4209
7b	Anx (cont)	Belief (bin)	Yes	0.989	0.970	1.009	0.966	0.908	1.027	0.2702	1.001	0.968	1.034	1.002	0.902	1.112	0.9752
8a	Dep (cont)	Identity (bin)	Religious	0.985	0.966	1.004	0.932	0.853	1.019	0.1162	0.983	0.954	1.013	0.924	0.806	1.063	0.2662
8b	Anx (cont)	Identity (bin)	Religious	0.962	0.937	0.988	0.883	0.811	0.964	0.0047	0.970	0.928	1.013	0.906	0.789	1.043	0.1659

**Model num.**	**Mental health expo-sure**	**RSBB out-come**	**RSBB out-come level**	**Est (no adj)**	**LCI (no adj)**	**UCI (no adj)**	**Est (no adj; std)**	**LCI (no adj; std)**	**UCI (no adj; std)**	***p* (no adj)**	**Est (adj)**	**LCI (adj)**	**UCI (adj)**	**Est (adj; std)**	**LCI (adj; std)**	**UCI (adj; std)**	***p* (adj)**
9a	Dep (cont)	Attend (bin)	Regular	1.005	0.989	1.022	1.025	0.950	1.104	0.5186	1.016	0.989	1.043	1.074	0.951	1.212	0.2469
9b	Anx (cont)	Attend (bin)	Regular	1.005	0.982	1.029	1.017	0.942	1.095	0.6683	1.017	0.979	1.057	1.057	0.934	1.195	0.3799
10a	Dep (bin)	Belief (bin)	Yes	0.789	0.631	0.986	-	-	-	0.0375	0.938	0.671	1.312	-	-	-	0.7101
10b	Anx (bin)	Belief (bin)	Yes	0.905	0.726	1.128	-	-	-	0.3760	1.034	0.743	1.439	-	-	-	0.8436
11a	Dep (bin)	Identity (bin)	Religious	0.654	0.492	0.880	-	-	-	0.0040	0.750	0.494	1.148	-	-	-	0.1800
11b	Anx (bin)	Identity (bin)	Religious	0.728	0.546	0.987	-	-	-	0.0359	0.693	0.451	1.076	-	-	-	0.0976
12a	Dep (bin)	Attend (bin)	Regular	1.079	0.822	1.400	-	-	-	0.5760	1.418	0.964	2.066	-	-	-	0.0724
12b	Anx (bin)	Attend (bin)	Regular	1.076	0.822	1.395	-	-	-	0.5845	1.203	0.817	1.754	-	-	-	0.3430

Table S17: Predicted probabilities of the mothers multinomial and logistic regression analyses with depression and anxiety exposures and religious/spiritual belief and behaviour (RSBB) outcomes (*n* = 4,025). These results indicate the change in the predicted probability of the RSBB outcome, based on either a standardised one-unit increase in depression or anxiety score (if exposure continuous) or the difference if a probable depression or anxiety diagnosis (if exposure binary). For the associated multinomial or logistic regression models, see table S16. LCI = Lower 95% confidence interval; UCI = Upper 95% confidence interval; no adj = unadjusted model; adj = Adjusted model.

**Model num.**	**Mental health exposure**	**RSBB outcome**	**RSBB outcome level**	**Est (no adj)**	**LCI (no adj)**	**UCI (no adj)**	**Est (no adj; std)**	**LCI (no adj; std)**	**UCI (no adj; std)**	**Est (adj)**	**LCI (adj)**	**UCI (adj)**	**Est (adj; std)**	**LCI (adj; std)**	**UCI (adj; std)**
1a	Dep (cont)	Belief (cat)	No	0.14	-0.11	0.39	0.64	-0.49	1.78	0.19	-0.04	0.42	0.88	-0.17	1.93
1a	Dep (cont)	Belief (cat)	Not sure	0.13	-0.19	0.45	0.61	-0.85	2.07	-0.07	-0.41	0.26	-0.32	-1.87	1.22
1a	Dep (cont)	Belief (cat)	Yes	-0.27	-0.61	0.06	-1.25	-2.80	0.29	-0.12	-0.42	0.18	-0.56	-1.94	0.82
1b	Anx (cont)	Belief (cat)	No	0.15	-0.20	0.51	0.49	-0.64	1.63	0.21	-0.12	0.55	0.68	-0.38	1.74
1b	Anx (cont)	Belief (cat)	Not sure	0.12	-0.34	0.58	0.37	-1.09	1.84	-0.22	-0.71	0.27	-0.71	-2.28	0.86
1b	Anx (cont)	Belief (cat)	Yes	-0.27	-0.76	0.21	-0.87	-2.41	0.68	0.01	-0.43	0.45	0.03	-1.38	1.43
2a	Dep (cont)	Identity (cat)	None	0.18	-0.04	0.40	0.82	-0.20	1.84	0.13	-0.07	0.33	0.60	-0.34	1.54
2a	Dep (cont)	Identity (cat)	Christian	-0.25	-0.49	-0.02	-1.16	-2.25	-0.07	-0.10	-0.32	0.12	-0.47	-1.46	0.53
2a	Dep (cont)	Identity (cat)	Other	0.07	-0.02	0.17	0.34	-0.10	0.78	-0.03	-0.14	0.08	-0.13	-0.64	0.37
2b	Anx (cont)	Identity (cat)	None	0.45	0.14	0.76	1.43	0.44	2.42	0.23	-0.07	0.52	0.72	-0.22	1.67
2b	Anx (cont)	Identity (cat)	Christian	-0.55	-0.88	-0.22	-1.75	-2.81	-0.69	-0.22	-0.54	0.09	-0.72	-1.72	0.28
2b	Anx (cont)	Identity (cat)	Other	0.10	-0.04	0.24	0.32	-0.12	0.76	0.00	-0.16	0.16	-0.01	-0.52	0.51
3a	Dep (cont)	Attend (cat)	Never	0.57	0.24	0.91	2.64	1.10	4.19	0.41	0.09	0.73	1.90	0.42	3.38
3a	Dep (cont)	Attend (cat)	1/yr	-0.68	-0.99	-0.36	-3.11	-4.55	-1.68	-0.58	-0.93	-0.23	-2.66	-4.25	-1.06
3a	Dep (cont)	Attend (cat)	1/mth	0.01	-0.19	0.22	0.06	-0.90	1.01	-0.09	-0.33	0.15	-0.43	-1.54	0.67
3a	Dep (cont)	Attend (cat)	1/wk	0.09	-0.11	0.29	0.41	-0.52	1.34	0.26	0.07	0.45	1.19	0.31	2.07
3b	Anx (cont)	Attend (cat)	Never	0.56	0.08	1.05	1.79	0.25	3.34	0.40	-0.07	0.87	1.27	-0.22	2.76
3b	Anx (cont)	Attend (cat)	1/yr	-0.66	-1.11	-0.21	-2.10	-3.53	-0.67	-0.60	-1.10	-0.09	-1.89	-3.49	-0.28
3b	Anx (cont)	Attend (cat)	1/mth	0.02	-0.28	0.32	0.07	-0.88	1.02	-0.14	-0.49	0.21	-0.46	-1.58	0.66
3b	Anx (cont)	Attend (cat)	1/wk	0.07	-0.22	0.37	0.24	-0.70	1.17	0.34	0.06	0.62	1.07	0.17	1.97
4a	Dep (bin)	Belief (cat)	No	4.03	-0.40	8.46	-	-	-	1.10	-2.36	4.55	-	-	-
4a	Dep (bin)	Belief (cat)	Not sure	1.85	-3.46	7.16	-	-	-	-0.20	-5.19	4.78	-	-	-

Model num.	Mental health exposure	RSBB outcome	RSBB outcome level	Est (no adj)	LCI (no adj)	UCI (no adj)	Est (no adj; std)	LCI (no adj; std)	UCI (no adj; std)	Est (adj)	LCI (adj)	UCI (adj)	Est (adj; std)	LCI (adj; std)	UCI (adj; std)
4a	Dep (bin)	Belief (cat)	Yes	-5.88	-11.37	-0.39	-	-	-	-0.89	-5.37	3.59	-	-	-
4b	Anx (bin)	Belief (cat)	No	2.14	-2.12	6.41	-	-	-	2.30	-1.32	5.93	-	-	-
4b	Anx (bin)	Belief (cat)	Not sure	0.34	-4.89	5.57	-	-	-	-2.66	-7.57	2.26	-	-	-
4b	Anx (bin)	Belief (cat)	Yes	-2.48	-7.97	3.00	-	-	-	0.35	-4.02	4.73	-	-	-
5a	Dep (bin)	Identity (cat)	None	5.55	1.31	9.79	-	-	-	2.19	-0.93	5.31	-	-	-
5a	Dep (bin)	Identity (cat)	Christian	-6.45	-10.95	-1.94	-	-	-	-1.50	-4.72	1.73	-	-	-
5a	Dep (bin)	Identity (cat)	Other	0.90	-1.03	2.83	-	-	-	-0.69	-1.94	0.55	-	-	-
5b	Anx (bin)	Identity (cat)	None	4.01	-0.08	8.10	-	-	-	2.77	-0.52	6.07	-	-	-
5b	Anx (bin)	Identity (cat)	Christian	-4.54	-8.89	-0.20	-	-	-	-2.44	-5.84	0.96	-	-	-
5b	Anx (bin)	Identity (cat)	Other	0.53	-1.29	2.36	-	-	-	-0.34	-1.79	1.12	-	-	-
6a	Dep (bin)	Attend (cat)	Never	9.98	4.55	15.41	-	-	-	5.27	0.60	9.93	-	-	-
6a	Dep (bin)	Attend (cat)	1/yr	-11.27	-15.55	-6.99	-	-	-	-9.09	-13.68	-4.51	-	-	-
6a	Dep (bin)	Attend (cat)	1/mth	0.41	-3.06	3.87	-	-	-	0.71	-2.89	4.32	-	-	-
6a	Dep (bin)	Attend (cat)	1/wk	0.89	-2.62	4.39	-	-	-	3.11	-0.30	6.53	-	-	-
6b	Anx (bin)	Attend (cat)	Never	4.96	-0.51	10.42	-	-	-	2.51	-2.15	7.16	-	-	-
6b	Anx (bin)	Attend (cat)	1/yr	-6.21	-10.81	-1.61	-	-	-	-4.67	-9.46	0.11	-	-	-
6b	Anx (bin)	Attend (cat)	1/mth	-0.40	-3.75	2.96	-	-	-	-1.08	-4.38	2.22	-	-	-
6b	Anx (bin)	Attend (cat)	1/wk	1.65	-1.91	5.22	-	-	-	3.24	-0.12	6.61	-	-	-
7a	Dep (cont)	Belief (bin)	Yes	-0.27	-0.61	0.06	-1.25	-2.80	0.29	-0.12	-0.42	0.18	-0.56	-1.94	0.81
7b	Anx (cont)	Belief (bin)	Yes	-0.27	-0.76	0.21	-0.87	-2.41	0.68	0.01	-0.43	0.45	0.02	-1.38	1.42
8a	Dep (cont)	Identity (bin)	Religious	-0.18	-0.40	0.04	-0.82	-1.84	0.20	-0.12	-0.32	0.09	-0.53	-1.47	0.41
8b	Anx (cont)	Identity (bin)	Religious	-0.45	-0.76	-0.14	-1.43	-2.42	-0.44	-0.21	-0.51	0.09	-0.67	-1.61	0.28
9a	Dep (cont)	Attend (bin)	Regular	0.09	-0.18	0.36	0.41	-0.84	1.67	0.15	-0.11	0.41	0.71	-0.49	1.90
9b	Anx (cont)	Attend (bin)	Regular	0.09	-0.31	0.48	0.27	-0.98	1.53	0.17	-0.21	0.55	0.54	-0.67	1.76
10a	Dep (bin)	Belief (bin)	Yes	-5.88	-11.37	-0.39	-	-	-	-0.85	-5.34	3.64	-	-	-
10b	Anx (bin)	Belief (bin)	Yes	-2.48	-7.97	3.00	-	-	-	0.44	-3.97	4.86	-	-	-

Model num.	Mental health exposure	RSBB outcome	RSBB outcome level	Est (no adj)	LCI (no adj)	UCI (no adj)	Est (no adj; std)	LCI (no adj; std)	UCI (no adj; std)	Est (adj)	LCI (adj)	UCI (adj)	Est (adj; std)	LCI (adj; std)	UCI (adj; std)
11a	Dep (bin)	Identity (bin)	Religious	-5.55	-9.79	-1.31	-	-	-	-2.04	-5.17	1.08	-	-	-
11b	Anx (bin)	Identity (bin)	Religious	-4.01	-8.10	0.08	-	-	-	-2.63	-5.93	0.66	-	-	-
12a	Dep (bin)	Attend (bin)	Regular	1.29	-3.32	5.90	-	-	-	3.63	-0.53	7.78	-	-	-
12b	Anx (bin)	Attend (bin)	Regular	1.26	-3.32	5.83	-	-	-	1.87	-2.10	5.84	-	-	-

Table S18: Descriptive statistics for mental health exposures 2 years post-delivery and religious/spiritual beliefs and behaviour (RSBB) outcomes 5 years post-delivery for partners (*n* = 2,120). EPDS = Edinburgh Postnatal Depression Scale; CCEI-A = Crown-Crisp Experiential Inventory – Anxiety subscale; SD = Standard deviation. Note that some cells with counts < 5 have been altered to preserve participant anonymity.

	**Religious Belief (categorical)**			**Religious Belief (binary)**		**Religious Affiliation (categorical)**			**Religious Affiliation (binary)**		**Religious Attendance (categorical)**				**Religious Attendance (binary)**	
	No (n = 608 [26.7%])	*Not Sure (n = 782 [36.9%])*	*Yes (n = 730 [34.4%])*	*No/Not Sure (n = 1,390 [65.6%])*	*Yes (n = 782 [34.4%])*	*None (n = 470 [22.2%])*	*Christian (n = 1,608 [75.8%])*	*Other (n = 42 [2.0%])*	*None (n = 470 [22.2%])*	*Religious (n = 1,650 [77.8%])*	*Not at all (n = 1,281 [60.4%])*	*Min. 1/ yr (n = 506 [23.9%])*	*Min. 1/ mth (n = 154 [7.3%])*	*Min. 1/ wk (n = 179 [8.4%])*	*Occ./ Never (n = 1,787 [84.3%])*	*Regular (n = 333 [15.7%])*
**EPDS depression scores (Mean = 3.25 [SD = 3.42])**																
Mean (SD)	2.85 (3.18)	3.30 (3.48)	3.53 (3.51)	3.10 (3.36)	3.53 (3.51)	2.78 (3.02)	3.38 (3.51)	3.60 (3.74)	2.78 (3.02)	3.38 (3.51)	3.16 (3.33)	3.23 (3.50)	3.68 (3.83)	3.55 (3.39)	3.18 (3.38)	3.61 (3.60)
**Probable depression diagnosis (*n* = 132 [6.2%])**																
No	580 (29.2%)	734 (36.9%)	674 (33.9%)	1,314 (66.1%)	674 (33.9%)	~450 (~23%)	~1,500 (~75%)	>37 (<2%)	~450 (~23%)	~1540 (~77%)	1,207 (60.7%)	474 (23.8%)	140 (7.0%)	167 (8.4%)	1,681 (84.6%)	307 (15.4%)
Yes	28 (21.2%)	48 (36.4%)	56 (42.4%)	76 (57.6%)	56 (42.4%)	~20 (~15%)	~110 (~82%)	<5 (<3%)	~20 (~15%)	~110 (~85%)	74 (56.1%)	32 (24.2%)	14 (10.6%)	12 (9.1%)	106 (80.3%)	26 (19.7%)
**CCEI-A anxiety scores (Mean = 2.41 [SD = 2.35])**																
Mean (SD)	2.22 (2.31)	2.42 (2.37)	2.56 (2.36)	2.34 (2.35)	2.56 (2.36)	2.25 (2.19)	2.44 (2.37)	3.26 (3.09)	2.25 (2.19)	2.46 (2.40)	2.38 (2.33)	2.34 (2.32)	2.79 (2.52)	2.54 (2.45)	2.37 (2.33)	2.66 (2.48)
**Probable anxiety diagnosis (*n* = 222 [10.5%])**																
No	553 (29.1%)	700 (36.9%)	645 (34.0%)	1,253 (66.0%)	645 (34.0%)	431 (22.7%)	1,432 (75.5%)	35 (1.8%)	431 (22.7%)	1,467 (77.3%)	1,149 (60.5%)	456 (24.0%)	134 (7.1%)	159 (8.4%)	1,605 (84.6%)	293 (15.4%)
Yes	55 (24.8%)	82 (36.9%)	85 (38.3%)	137 (61.7%)	85 (38.3%)	39 (17.6%)	176 (79.3%)	7 (3.2%)	39 (17.6%)	183 (82.4%)	132 (59.5%)	50 (22.5%)	20 (9.0%)	20 (9.0%)	182 (82.0%)	40 (18.0%)

Table S19: Results of the partners analyses with depression and anxiety exposures and religious/spiritual belief and behaviour (RSBB) as outcomes (*n* = 2,120). Est = Estimate (either relative risk ratio from a multinomial model, or odds ratio from a logistic model); LCI = Lower 95% confidence interval; UCI = Upper 95% confidence interval; *p* = *p*-value; no adj = unadjusted model; adj = Adjusted model; std = model using standardised depression or anxiety scores.

Model num.	Mental health expo-sure	RSBB out-come	RSBB out-come level	Est (no adj)	LCI (no adj)	UCI (no adj)	Est (no adj; std)	LCI (no adj; std)	UCI (no adj; std)	*p* (no adj)	Est (adj)	LCI (adj)	UCI (adj)	Est (adj; std)	LCI (adj; std)	UCI (adj; std)	*p* (adj)
1a	Dep (cont)	Belief (cat)	Not sure	1.043	1.009	1.077	1.153	1.030	1.290	0.0130	1.015	0.966	1.067	1.053	0.889	1.247	0.5530
1a	Dep (cont)	Belief (cat)	Yes	1.062	1.028	1.097	1.228	1.098	1.374	0.0003	1.025	0.965	1.089	1.089	0.884	1.340	0.4237
1b	Anx (cont)	Belief (cat)	Not sure	1.040	0.992	1.090	1.096	0.982	1.223	0.1028	0.995	0.924	1.071	0.988	0.831	1.175	0.8928
1b	Anx (cont)	Belief (cat)	Yes	1.065	1.016	1.116	1.160	1.039	1.294	0.0084	1.004	0.917	1.098	1.009	0.817	1.246	0.9362
2a	Dep (cont)	Identity (cat)	Christian	1.058	1.023	1.093	1.210	1.081	1.355	0.0009	1.038	0.985	1.094	1.137	0.950	1.361	0.1638
2a	Dep (cont)	Identity (cat)	Other	1.076	0.985	1.175	1.283	0.949	1.733	0.1044	1.008	0.879	1.156	1.031	0.645	1.647	0.9108
2b	Anx (cont)	Identity (cat)	Christian	1.036	0.990	1.085	1.088	0.977	1.211	0.1241	1.038	0.961	1.122	1.093	0.912	1.311	0.3373
2b	Anx (cont)	Identity (cat)	Other	1.168	1.043	1.307	1.440	1.105	1.877	0.0070	1.151	0.955	1.386	1.390	0.897	2.153	0.1400
3a	Dep (cont)	Attend (cat)	1/yr	1.006	0.976	1.037	1.021	0.921	1.133	0.6882	0.992	0.950	1.035	0.972	0.839	1.126	0.7013
3a	Dep (cont)	Attend (cat)	1/mth	1.042	0.996	1.091	1.151	0.985	1.345	0.0769	1.003	0.932	1.078	1.011	0.789	1.295	0.9435
3a	Dep (cont)	Attend (cat)	1/wk	1.033	0.989	1.079	1.116	0.962	1.295	0.1486	1.050	0.953	1.156	1.184	0.852	1.644	0.3227
3b	Anx (cont)	Attend (cat)	1/yr	0.991	0.948	1.037	0.980	0.882	1.089	0.7067	0.937	0.877	1.001	0.858	0.735	1.003	0.0546
3b	Anx (cont)	Attend (cat)	1/mth	1.070	1.002	1.142	1.172	1.006	1.366	0.0421	1.035	0.932	1.150	1.083	0.846	1.387	0.5217
3b	Anx (cont)	Attend (cat)	1/wk	1.029	0.964	1.097	1.069	0.918	1.244	0.3913	0.979	0.849	1.128	0.950	0.681	1.326	0.7665
4a	Dep (bin)	Belief (cat)	Not sure	1.355	0.839	2.186	-	-	-	0.2139	1.158	0.602	2.228	-	-	-	0.6596

Model num.	Mental health expo-sure	RSBB out-come	RSBB out-come level	Est (no adj)	LCI (no adj)	UCI (no adj)	Est (no adj; std)	LCI (no adj; std)	UCI (no adj; std)	*p* (no adj)	Est (adj)	LCI (adj)	UCI (adj)	Est (adj; std)	LCI (adj; std)	UCI (adj; std)	*p* (adj)
4a	Dep (bin)	Belief (cat)	Yes	1.721	1.079	2.746	-	-	-	0.0227	1.318	0.614	2.831	-	-	-	0.4784
4b	Anx (bin)	Belief (cat)	Not sure	1.178	0.822	1.687	-	-	-	0.3720	1.100	0.661	1.831	-	-	-	0.7141
4b	Anx (bin)	Belief (cat)	Yes	1.325	0.927	1.895	-	-	-	0.1231	1.136	0.617	2.093	-	-	-	0.6814
5a	Dep (bin)	Identity (cat)	Christian	1.621	0.994	2.642	-	-	-	0.0528	1.425	0.715	2.838	-	-	-	0.3141
5a	Dep (bin)	Identity (cat)	Other	2.370	0.771	7.290	-	-	-	0.1323	1.322	0.268	6.517	-	-	-	0.7313
5b	Anx (bin)	Identity (cat)	Christian	1.358	0.945	1.953	-	-	-	0.0985	1.385	0.808	2.373	-	-	-	0.2362
5b	Anx (bin)	Identity (cat)	Other	2.211	0.922	5.305	-	-	-	0.0755	2.520	0.744	8.536	-	-	-	0.1375
6a	Dep (bin)	Attend (cat)	1/yr	1.098	0.716	1.685	-	-	-	0.6672	1.130	0.664	1.925	-	-	-	0.6525
6a	Dep (bin)	Attend (cat)	1/mth	1.634	0.899	2.968	-	-	-	0.1072	1.484	0.646	3.407	-	-	-	0.3524
6a	Dep (bin)	Attend (cat)	1/wk	1.166	0.620	2.194	-	-	-	0.6341	1.452	0.461	4.572	-	-	-	0.5236
6b	Anx (bin)	Attend (cat)	1/yr	0.954	0.677	1.345	-	-	-	0.7899	0.845	0.546	1.308	-	-	-	0.4506
6b	Anx (bin)	Attend (cat)	1/mth	1.299	0.786	2.149	-	-	-	0.3079	1.267	0.632	2.540	-	-	-	0.5043
6b	Anx (bin)	Attend (cat)	1/wk	1.095	0.665	1.803	-	-	-	0.7216	1.154	0.446	2.981	-	-	-	0.7679
7a	Dep (cont)	Belief (bin)	Yes	1.036	1.010	1.063	1.129	1.034	1.233	0.0068	1.012	0.966	1.059	1.041	0.890	1.216	0.6162
7b	Anx (cont)	Belief (bin)	Yes	1.041	1.003	1.081	1.100	1.006	1.201	0.0350	1.006	0.941	1.077	1.015	0.866	1.190	0.8513
8a	Dep (cont)	Identity (bin)	Religious	1.058	1.024	1.094	1.212	1.086	1.360	0.0008	1.037	0.985	1.093	1.133	0.950	1.355	0.1685
8b	Anx (cont)	Identity (bin)	Religious	1.040	0.995	1.089	1.097	0.988	1.223	0.0888	1.042	0.966	1.125	1.101	0.921	1.320	0.2926

**Model num.**	**Mental health expo-sure**	**RSBB out-come**	**RSBB out-come level**	**Est (no adj)**	**LCI (no adj)**	**UCI (no adj)**	**Est (no adj; std)**	**LCI (no adj; std)**	**UCI (no adj; std)**	***p* (no adj)**	**Est (adj)**	**LCI (adj)**	**UCI (adj)**	**Est (adj; std)**	**LCI (adj; std)**	**UCI (adj; std)**	***p* (adj)**
9a	Dep (cont)	Attend (bin)	Regular	1.035	1.002	1.069	1.125	1.006	1.255	0.0366	1.020	0.959	1.083	1.070	0.867	1.313	0.5233
9b	Anx (cont)	Attend (bin)	Regular	1.051	1.001	1.101	1.123	1.004	1.253	0.0401	1.050	0.959	1.148	1.121	0.905	1.382	0.2903
10a	Dep (bin)	Belief (bin)	Yes	1.437	1.001	2.050	-	-	-	0.0470	1.156	0.673	1.987	-	-	-	0.6000
10b	Anx (bin)	Belief (bin)	Yes	1.205	0.902	1.602	-	-	-	0.2020	1.041	0.664	1.632	-	-	-	0.8602
11a	Dep (bin)	Identity (bin)	Religious	1.638	1.030	2.742	-	-	-	0.0470	1.433	0.736	2.881	-	-	-	0.3008
11b	Anx (bin)	Identity (bin)	Religious	1.379	0.970	2.005	-	-	-	0.0821	1.387	0.819	2.386	-	-	-	0.2297
12a	Dep (bin)	Attend (bin)	Regular	1.343	0.844	2.066	-	-	-	0.1948	1.382	0.679	2.707	-	-	-	0.3572
12b	Anx (bin)	Attend (bin)	Regular	1.204	0.827	1.716	-	-	-	0.3179	1.337	0.728	2.404	-	-	-	0.3398

Table S20: Predicted probabilities of the partners multinomial and logistic regression analyses with depression and anxiety exposures and religious/spiritual belief and behaviour (RSBB) outcomes (n = 2,120). These results indicate the change in the predicted probability of the RSBB outcome, based on either a standardised one-unit increase in depression or anxiety score (if exposure continuous) or the difference if a probable depression or anxiety diagnosis (if exposure binary). For the associated multinomial or logistic regression models, see table S19. LCI = Lower 95% confidence interval; UCI = Upper 95% confidence interval; no adj = unadjusted model; adj = Adjusted model.

**Model num.**	**Mental health exposure**	**RSBB outcome**	**RSBB outcome level**	**Est (no adj)**	**LCI (no adj)**	**UCI (no adj)**	**Est (no adj; std)**	**LCI (no adj; std)**	**UCI (no adj; std)**	**Est (adj)**	**LCI (adj)**	**UCI (adj)**	**Est (adj; std)**	**LCI (adj; std)**	**UCI (adj; std)**
1a	Dep (cont)	Belief (cat)	No	-1.03	-1.62	-0.43	-3.51	-5.55	-1.48	-0.18	-0.69	0.33	-0.61	-2.34	1.12
1a	Dep (cont)	Belief (cat)	Not sure	0.20	-0.40	0.80	0.70	-1.35	2.75	0.04	-0.60	0.68	0.13	-2.06	2.32
1a	Dep (cont)	Belief (cat)	Yes	0.82	0.25	1.40	2.81	0.85	4.78	0.14	-0.36	0.65	0.48	-1.25	2.21
1b	Anx (cont)	Belief (cat)	No	-1.03	-1.88	-0.18	-2.42	-4.42	-0.43	0.03	-0.72	0.79	0.08	-1.69	1.85
1b	Anx (cont)	Belief (cat)	Not sure	0.11	-0.77	0.98	0.25	-1.80	2.31	-0.12	-1.08	0.84	-0.28	-2.54	1.97
1b	Anx (cont)	Belief (cat)	Yes	0.92	0.08	1.76	2.17	0.19	4.15	0.09	-0.67	0.84	0.20	-1.57	1.97
2a	Dep (cont)	Identity (cat)	None	-0.97	-1.53	-0.40	-3.30	-5.22	-1.38	-0.34	-0.84	0.16	-1.18	-2.88	0.52
2a	Dep (cont)	Identity (cat)	Christian	0.91	0.34	1.48	3.11	1.15	5.06	0.37	-0.14	0.87	1.26	-0.48	2.99
2a	Dep (cont)	Identity (cat)	Other	0.06	-0.11	0.22	0.20	-0.36	0.75	-0.02	-0.22	0.17	-0.08	-0.75	0.60
2b	Anx (cont)	Identity (cat)	None	-0.67	-1.45	0.11	-1.57	-3.40	0.27	-0.42	-1.15	0.32	-0.98	-2.71	0.74
2b	Anx (cont)	Identity (cat)	Christian	0.42	-0.38	1.22	0.99	-0.89	2.86	0.25	-0.49	0.98	0.58	-1.16	2.32
2b	Anx (cont)	Identity (cat)	Other	0.25	0.03	0.46	0.58	0.07	1.10	0.17	-0.09	0.44	0.40	-0.22	1.03
3a	Dep (cont)	Attend (cat)	Never	-0.43	-1.04	0.17	-1.48	-3.55	0.58	0.05	-0.55	0.66	0.17	-1.89	2.23
3a	Dep (cont)	Attend (cat)	1/yr	-0.02	-0.56	0.51	-0.08	-1.90	1.74	-0.18	-0.78	0.43	-0.60	-2.67	1.47
3a	Dep (cont)	Attend (cat)	1/mth	0.25	-0.05	0.55	0.84	-0.19	1.87	-0.02	-0.39	0.35	-0.06	-1.33	1.21
3a	Dep (cont)	Attend (cat)	1/wk	0.21	-0.12	0.54	0.72	-0.40	1.84	0.14	-0.12	0.40	0.49	-0.39	1.37
3b	Anx (cont)	Attend (cat)	Never	-0.32	-1.20	0.57	-0.74	-2.82	1.33	0.70	-0.21	1.62	1.66	-0.49	3.81
3b	Anx (cont)	Attend (cat)	1/yr	-0.33	-1.11	0.46	-0.77	-2.62	1.07	-1.02	-1.95	-0.10	-2.41	-4.59	-0.23
3b	Anx (cont)	Attend (cat)	1/mth	0.45	0.02	0.88	1.06	0.05	2.08	0.36	-0.17	0.89	0.84	-0.41	2.09
3b	Anx (cont)	Attend (cat)	1/wk	0.19	-0.29	0.68	0.46	-0.69	1.60	-0.04	-0.41	0.34	-0.09	-0.97	0.79
4a	Dep (bin)	Belief (cat)	No	-7.96	-15.22	-0.71	-	-	-	-1.79	-8.26	4.69	-	-	-
4a	Dep (bin)	Belief (cat)	Not sure	-0.56	-9.03	7.92	-	-	-	0.02	-7.98	8.03	-	-	-

Model num.	Mental health exposure	RSBB outcome	RSBB outcome level	Est (no adj)	LCI (no adj)	UCI (no adj)	Est (no adj; std)	LCI (no adj; std)	UCI (no adj; std)	Est (adj)	LCI (adj)	UCI (adj)	Est (adj; std)	LCI (adj; std)	UCI (adj; std)
4a	Dep (bin)	Belief (cat)	Yes	8.52	-0.16	17.21	-	-	-	1.76	-4.34	7.87	-	-	-
4b	Anx (bin)	Belief (cat)	No	-4.36	-10.40	1.67	-	-	-	-1.06	-6.18	4.07	-	-	-
4b	Anx (bin)	Belief (cat)	Not sure	0.06	-6.65	6.77	-	-	-	0.49	-5.99	6.97	-	-	-
4b	Anx (bin)	Belief (cat)	Yes	4.31	-2.43	11.05	-	-	-	0.57	-4.45	5.59	-	-	-
5a	Dep (bin)	Identity (cat)	None	-7.49	-13.88	-1.10	-	-	-	-3.25	-9.37	2.88	-	-	-
5a	Dep (bin)	Identity (cat)	Christian	6.37	-0.47	13.22	-	-	-	3.17	-3.06	9.41	-	-	-
5a	Dep (bin)	Identity (cat)	Other	1.12	-1.87	4.10	-	-	-	0.07	-2.26	2.41	-	-	-
5b	Anx (bin)	Identity (cat)	None	-5.14	-10.49	0.21	-	-	-	-3.41	-8.24	1.41	-	-	-
5b	Anx (bin)	Identity (cat)	Christian	3.83	-1.84	9.50	-	-	-	2.14	-2.84	7.12	-	-	-
5b	Anx (bin)	Identity (cat)	Other	1.31	-1.07	3.69	-	-	-	1.27	-1.18	3.73	-	-	-
6a	Dep (bin)	Attend (cat)	Never	-4.60	-13.33	4.13	-	-	-	-2.69	-10.21	4.83	-	-	-
6a	Dep (bin)	Attend (cat)	1/yr	0.36	-7.18	7.91	-	-	-	0.52	-7.03	8.07	-	-	-
6a	Dep (bin)	Attend (cat)	1/mth	3.59	-1.79	8.96	-	-	-	1.63	-3.33	6.60	-	-	-
6a	Dep (bin)	Attend (cat)	1/wk	0.65	-4.39	5.69	-	-	-	0.54	-2.87	3.95	-	-	-
6b	Anx (bin)	Attend (cat)	Never	-1.08	-7.90	5.74	-	-	-	1.20	-4.72	7.11	-	-	-
6b	Anx (bin)	Attend (cat)	1/yr	-1.50	-7.32	4.32	-	-	-	-3.12	-8.90	2.67	-	-	-
6b	Anx (bin)	Attend (cat)	1/mth	1.95	-1.99	5.89	-	-	-	1.63	-2.39	5.65	-	-	-
6b	Anx (bin)	Attend (cat)	1/wk	0.63	-3.34	4.60	-	-	-	0.29	-2.39	2.97	-	-	-
7a	Dep (cont)	Belief (bin)	Yes	0.80	0.22	1.37	2.73	0.77	4.70	0.13	-0.38	0.64	0.44	-1.29	2.17
7b	Anx (cont)	Belief (bin)	Yes	0.91	0.07	1.75	2.14	0.16	4.12	0.07	-0.68	0.82	0.17	-1.59	1.93
8a	Dep (cont)	Identity (bin)	Religious	0.97	0.41	1.53	3.30	1.38	5.23	0.35	-0.15	0.85	1.20	-0.50	2.90
8b	Anx (cont)	Identity (bin)	Religious	0.68	-0.10	1.46	1.60	-0.24	3.43	0.39	-0.34	1.13	0.93	-0.80	2.65
9a	Dep (cont)	Attend (bin)	Regular	0.46	0.03	0.88	1.56	0.10	3.02	0.12	-0.25	0.50	0.42	-0.87	1.71
9b	Anx (cont)	Attend (bin)	Regular	0.65	0.03	1.28	1.54	0.07	3.00	0.30	-0.26	0.86	0.71	-0.60	2.02
10a	Dep (bin)	Belief (bin)	Yes	8.52	-0.16	17.21	-	-	-	1.61	-4.45	7.67	-	-	-
10b	Anx (bin)	Belief (bin)	Yes	4.31	-2.43	11.05	-	-	-	0.45	-4.54	5.44	-	-	-

Model num.	Mental health exposure	RSBB outcome	RSBB outcome level	Est (no adj)	LCI (no adj)	UCI (no adj)	Est (no adj; std)	LCI (no adj; std)	UCI (no adj; std)	Est (adj)	LCI (adj)	UCI (adj)	Est (adj; std)	LCI (adj; std)	UCI (adj; std)
11a	Dep (bin)	Identity (bin)	Religious	7.48	1.10	13.87	-	-	-	3.34	-2.77	9.45	-	-	-
11b	Anx (bin)	Identity (bin)	Religious	5.14	-0.21	10.49	-	-	-	3.06	-1.79	7.90	-	-	-
12a	Dep (bin)	Attend (bin)	Regular	4.25	-2.71	11.22	-	-	-	2.16	-2.75	7.06	-	-	-
12b	Anx (bin)	Attend (bin)	Regular	2.58	-2.73	7.89	-	-	-	1.90	-2.22	6.03	-	-	-

Table S21: Results of the interaction analyses assessing whether the adjusted mother and partner results differ, with standardised depression and anxiety scores, or binary probable depression and anxiety diagnoses, as exposures and categorical religious/spiritual belief and behaviour (RSBB) as outcomes. RRR = Relative risk ratio; SE = Standard error; LCI = Lower 95% confidence interval; UCI = Upper 95% confidence interval; *p* = *p*-value.

**Model num.**	**Exposure**	**Outcome**	**Out-come level**	**Mother RRR**	**Mother log-RRR**	**Mother log-RRR SE**	**Partner RRR**	**Partner log-RRR**	**Partner log-RRR SE**	**Log-RRR diff.**	**Diff. log-RRR SE**	**Diff. RRR**	**Diff. RRR LCI**	**Diff. RRR UCI**	**Diff. *p***
1a	Dep (cont)	Belief (cat)	Not sure	0.908	-0.097	0.064	1.053	0.052	0.086	-0.148	0.108	0.862	0.698	1.065	0.1683
1a	Dep (cont)	Belief (cat)	Yes	0.886	-0.121	0.074	1.089	0.085	0.106	-0.206	0.130	0.814	0.631	1.049	0.1113
1b	Anx (cont)	Belief (cat)	Not sure	0.920	-0.083	0.065	0.988	-0.012	0.088	-0.071	0.110	0.931	0.751	1.155	0.5163
1b	Anx (cont)	Belief (cat)	Yes	0.935	-0.067	0.075	1.009	0.009	0.108	-0.076	0.131	0.927	0.716	1.199	0.5621
2a	Dep (cont)	Identity (cat)	Christian	0.919	-0.084	0.072	1.137	0.128	0.092	-0.213	0.117	0.808	0.643	1.016	0.0678
2a	Dep (cont)	Identity (cat)	Other	0.879	-0.129	0.146	1.031	0.031	0.239	-0.159	0.280	0.853	0.492	1.477	0.5694
2b	Anx (cont)	Identity (cat)	Christian	0.896	-0.110	0.072	1.093	0.089	0.093	-0.199	0.117	0.820	0.652	1.031	0.0897
2b	Anx (cont)	Identity (cat)	Other	0.924	-0.079	0.149	1.390	0.329	0.223	-0.408	0.268	0.665	0.393	1.125	0.1279
3a	Dep (cont)	Attend (cat)	1/yr	0.845	-0.168	0.054	0.972	-0.028	0.075	-0.140	0.092	0.869	0.725	1.042	0.1298
3a	Dep (cont)	Attend (cat)	1/mth	0.903	-0.102	0.078	1.011	0.011	0.126	-0.113	0.149	0.893	0.667	1.195	0.4474
3a	Dep (cont)	Attend (cat)	1/wk	1.134	0.126	0.091	1.184	0.169	0.168	-0.043	0.191	0.958	0.659	1.392	0.8209
3b	Anx (cont)	Attend (cat)	1/yr	0.890	-0.117	0.054	0.858	-0.153	0.079	0.037	0.096	1.037	0.859	1.252	0.7028
3b	Anx (cont)	Attend (cat)	1/mth	0.923	-0.080	0.079	1.083	0.080	0.126	-0.160	0.149	0.852	0.637	1.141	0.2828
3b	Anx (cont)	Attend (cat)	1/wk	1.139	0.130	0.092	0.950	-0.051	0.170	0.181	0.194	1.199	0.821	1.752	0.3484
4a	Dep (bin)	Belief (cat)	Not sure	0.893	-0.113	0.203	1.158	0.147	0.334	-0.260	0.391	0.771	0.359	1.658	0.5059
4a	Dep (bin)	Belief (cat)	Yes	0.853	-0.159	0.238	1.318	0.276	0.390	-0.435	0.457	0.647	0.264	1.584	0.3406
4b	Anx (bin)	Belief (cat)	Not sure	0.754	-0.282	0.207	1.100	0.095	0.260	-0.378	0.333	0.685	0.357	1.315	0.2561
4b	Anx (bin)	Belief (cat)	Yes	0.816	-0.203	0.238	1.136	0.128	0.312	-0.331	0.392	0.718	0.333	1.549	0.3988
5a	Dep (bin)	Identity (cat)	Christian	0.752	-0.285	0.218	1.425	0.354	0.352	-0.639	0.414	0.528	0.234	1.188	0.1225
5a	Dep (bin)	Identity (cat)	Other	0.542	-0.612	0.453	1.322	0.279	0.814	-0.892	0.932	0.410	0.066	2.545	0.3385
5b	Anx (bin)	Identity (cat)	Christian	0.684	-0.380	0.223	1.385	0.326	0.275	-0.705	0.354	0.494	0.247	0.989	0.0464
5b	Anx (bin)	Identity (cat)	Other	0.637	-0.451	0.468	2.520	0.924	0.622	-1.375	0.778	0.253	0.055	1.163	0.0773
6a	Dep (bin)	Attend (cat)	1/yr	0.564	-0.573	0.184	1.130	0.122	0.272	-0.695	0.328	0.499	0.262	0.949	0.0341
6a	Dep (bin)	Attend (cat)	1/mth	0.931	-0.071	0.243	1.484	0.395	0.424	-0.466	0.489	0.627	0.241	1.635	0.3401
6a	Dep (bin)	Attend (cat)	1/wk	1.364	0.310	0.286	1.452	0.373	0.585	-0.063	0.651	0.939	0.262	3.367	0.9235
6b	Anx (bin)	Attend (cat)	1/yr	0.760	-0.274	0.174	0.845	-0.168	0.223	-0.106	0.283	0.899	0.516	1.566	0.7080

6b	Anx (bin)	Attend (cat)	1/mth	0.853	-0.159	0.247	1.267	0.237	0.355	-0.396	0.432	0.673	0.289	1.571	0.3601
6b	Anx (bin)	Attend (cat)	1/wk	1.488	0.397	0.283	1.154	0.143	0.485	0.254	0.561	1.289	0.429	3.875	0.6507

Table S22: Results of the interaction analyses assessing whether the adjusted mother and partner results differ, with standardised depression and anxiety scores, or binary probable depression and anxiety diagnoses, as exposures and binary religious/spiritual belief and behaviour (RSBB) as outcomes. OR = Odds ratio; SE = Standard error; LCI = Lower 95% confidence interval; UCI = Upper 95% confidence interval; *p* = *p*-value.

**Model num.**	**Exposure**	**Outcome**	**Out-come level**	**Mother OR**	**Mother log-odds**	**Mother log-odds SE**	**Partner OR**	**Partner log-odds**	**Partner log-odds SE**	**Log-odds diff.**	**Diff. log-odds SE**	**Diff. OR**	**Diff. OR LCI**	**Diff. OR UCI**	**Diff. *p***
7a	Dep (cont)	Belief (bin)	Yes	0.959	-0.042	0.052	1.041	0.040	0.080	-0.082	0.095	0.921	0.764	1.110	0.3892
7b	Anx (cont)	Belief (bin)	Yes	1.002	0.002	0.053	1.015	0.015	0.081	-0.013	0.097	0.987	0.816	1.194	0.8944
8a	Dep (cont)	Identity (bin)	Religious	0.924	-0.079	0.071	1.133	0.125	0.091	-0.204	0.115	0.816	0.651	1.021	0.0758
8b	Anx (cont)	Identity (bin)	Religious	0.906	-0.099	0.071	1.101	0.096	0.092	-0.195	0.116	0.823	0.655	1.033	0.0934
9a	Dep (cont)	Attend (bin)	Regular	1.074	0.071	0.062	1.070	0.068	0.106	0.004	0.123	1.004	0.789	1.276	0.9757
9b	Anx (cont)	Attend (bin)	Regular	1.057	0.055	0.063	1.121	0.114	0.108	-0.059	0.125	0.943	0.738	1.205	0.6380
10a	Dep (bin)	Belief (bin)	Yes	0.938	-0.064	0.171	1.156	0.145	0.276	-0.209	0.325	0.811	0.429	1.534	0.5201
10b	Anx (bin)	Belief (bin)	Yes	1.034	0.033	0.169	1.041	0.040	0.229	-0.007	0.285	0.993	0.568	1.735	0.9811
11a	Dep (bin)	Identity (bin)	Religious	0.750	-0.288	0.215	1.433	0.360	0.348	-0.647	0.409	0.523	0.235	1.167	0.1136
11b	Anx (bin)	Identity (bin)	Religious	0.693	-0.367	0.222	1.387	0.327	0.273	-0.694	0.352	0.500	0.251	0.995	0.0484
12a	Dep (bin)	Attend (bin)	Regular	1.418	0.349	0.194	1.382	0.324	0.353	0.026	0.403	1.026	0.466	2.260	0.9491
12b	Anx (bin)	Attend (bin)	Regular	1.203	0.185	0.195	1.337	0.290	0.305	-0.106	0.362	0.900	0.443	1.828	0.7703

Table S23: Results of the mothers analyses with religious/spiritual belief and behaviour (RSBB) exposures and depression and anxiety as outcomes using multiple imputation (*n* = 13,085). Est = Estimate (either mean difference from a linear model, or odds ratio from a logistic model); LCI = Lower 95% confidence interval; UCI = Upper 95% confidence interval; *p* = *p*-value; no adj = unadjusted model; adj = Adjusted model; std = model using standardised depression or anxiety scores.

**Model num.**	**RSBB expo-sure**	**RSBB expo-sure level**	**Out-come**	**Est (no adj)**	**LCI (no adj)**	**UCI (no adj)**	**Est (no adj; std)**	**LCI (no adj; std)**	**UCI (no adj; std)**	***p* (no adj)**	**Est (adj)**	**LCI (adj)**	**UCI (adj)**	**Est (adj; std)**	**LCI (adj; std)**	**UCI (adj; std)**	***p* (adj)**
1a	Belief (cat)	Not sure	Dep (cont)	0.084	-0.225	0.394	0.019	-0.040	0.078	0.5924	0.035	-0.295	0.366	0.007	-0.056	0.078	0.5924
1a	Belief (cat)	Yes	Dep (cont)	0.044	-0.269	0.357	0.012	-0.048	0.072	0.7839	-0.011	-0.408	0.387	-0.002	-0.079	0.074	0.9585
1b	Identity (cat)	Christian	Dep (cont)	-0.138	-0.445	0.169	-0.025	-0.084	0.034	0.3768	-0.061	-0.430	0.307	-0.012	-0.082	0.059	0.7431
1b	Identity (cat)	Other	Dep (cont)	1.052	0.374	1.731	0.200	0.071	0.330	0.0025	-0.284	-0.943	0.376	-0.054	-0.181	0.072	0.3978
1c	Attend (cat)	1/yr	Dep (cont)	-0.156	-0.423	0.112	-0.027	-0.078	0.025	0.2527	0.035	-0.238	0.309	0.007	-0.046	0.059	0.7990
1c	Attend (cat)	1/mth	Dep (cont)	-0.093	-0.486	0.301	-0.013	-0.088	0.062	0.6436	-0.145	-0.539	0.250	-0.027	-0.103	0.048	0.4715
1c	Attend (cat)	1/wk	Dep (cont)	-0.369	-0.737	-0.002	-0.066	-0.136	0.004	0.0489	-0.247	-0.720	0.226	-0.048	-0.138	0.043	0.3047
2a	Belief (cat)	Not sure	Anx (cont)	0.120	-0.103	0.342	0.034	-0.026	0.095	0.2912	-0.036	-0.266	0.193	-0.010	-0.072	0.053	0.7553
2a	Belief (cat)	Yes	Anx (cont)	0.168	-0.053	0.388	0.048	-0.012	0.108	0.1360	-0.050	-0.326	0.225	-0.014	-0.089	0.061	0.7189
2b	Identity (cat)	Christian	Anx (cont)	-0.216	-0.419	-0.013	-0.058	-0.113	-0.003	0.0368	-0.137	-0.390	0.115	-0.037	-0.106	0.032	0.2852
2b	Identity (cat)	Other	Anx (cont)	0.679	0.162	1.195	0.184	0.044	0.323	0.0103	-0.144	-0.649	0.361	-0.039	-0.176	0.098	0.5743
2c	Attend (cat)	1/yr	Anx (cont)	-0.033	-0.229	0.162	-0.007	-0.060	0.046	0.7366	0.039	-0.155	0.234	0.011	-0.042	0.064	0.6905
2c	Attend (cat)	1/mth	Anx (cont)	-0.026	-0.309	0.258	-0.004	-0.081	0.073	0.8593	-0.201	-0.495	0.094	-0.054	-0.134	0.026	0.1812
2c	Attend (cat)	1/wk	Anx (cont)	-0.185	-0.445	0.076	-0.047	-0.118	0.024	0.1651	-0.300	-0.636	0.036	-0.082	-0.173	0.009	0.0797
3a	Belief (bin)	Yes	Dep (cont)	-0.008	-0.244	0.227	0.001	-0.045	0.046	0.9455	-0.039	-0.322	0.245	-0.008	-0.062	0.047	0.7889

Model num.	RSBB expo-sure	RSBB expo-sure level	Out-come	Est (no adj)	LCI (no adj)	UCI (no adj)	Est (no adj; std)	LCI (no adj; std)	UCI (no adj; std)	*p* (no adj)	Est (adj)	LCI (adj)	UCI (adj)	Est (adj; std)	LCI (adj; std)	UCI (adj; std)	*p* (adj)
3b	Identity (bin)	Religious	Dep (cont)	-0.086	-0.392	0.220	-0.015	-0.074	0.043	0.5804	-0.086	-0.446	0.274	-0.016	-0.085	0.053	0.6396
3c	Attend (bin)	Regular	Dep (cont)	-0.189	-0.456	0.078	-0.032	-0.084	0.019	0.1646	-0.202	-0.519	0.115	-0.039	-0.100	0.023	0.2116
4a	Belief (bin)	Yes	Anx (cont)	0.094	-0.069	0.257	0.027	-0.017	0.071	0.2570	-0.021	-0.215	0.173	-0.006	-0.059	0.047	0.8281
4b	Identity (bin)	Religious	Anx (cont)	-0.177	-0.379	0.025	-0.047	-0.102	0.008	0.0865	-0.138	-0.388	0.113	-0.037	-0.106	0.031	0.2793
4c	Attend (bin)	Regular	Anx (cont)	-0.098	-0.290	0.094	-0.024	-0.076	0.028	0.3173	-0.259	-0.490	-0.028	-0.070	-0.133	-0.008	0.0278
5a	Belief (cat)	Not sure	Dep (bin)	0.962	0.815	1.136	-	-	-	0.6503	0.947	0.767	1.170	-	-	-	0.6149
5a	Belief (cat)	Yes	Dep (bin)	0.945	0.801	1.114	-	-	-	0.4970	0.930	0.714	1.211	-	-	-	0.5887
5b	Identity (cat)	Christian	Dep (bin)	0.891	0.756	1.051	-	-	-	0.1698	0.947	0.754	1.189	-	-	-	0.6402
5b	Identity (cat)	Other	Dep (bin)	1.367	1.013	1.845	-	-	-	0.0408	0.842	0.582	1.216	-	-	-	0.3581
5c	Attend (cat)	1/yr	Dep (bin)	0.933	0.809	1.075	-	-	-	0.3371	1.045	0.873	1.252	-	-	-	0.6292
5c	Attend (cat)	1/mth	Dep (bin)	0.959	0.779	1.180	-	-	-	0.6926	0.988	0.762	1.282	-	-	-	0.9289
5c	Attend (cat)	1/wk	Dep (bin)	0.783	0.632	0.970	-	-	-	0.0250	0.924	0.667	1.281	-	-	-	0.6363
6a	Belief (cat)	Not sure	Anx (bin)	0.993	0.855	1.154	-	-	-	0.9301	0.909	0.742	1.115	-	-	-	0.3582
6a	Belief (cat)	Yes	Anx (bin)	1.003	0.863	1.165	-	-	-	0.9687	0.910	0.713	1.161	-	-	-	0.4456
6b	Identity (cat)	Christian	Anx (bin)	0.876	0.764	1.005	-	-	-	0.0597	0.904	0.729	1.121	-	-	-	0.3557
6b	Identity (cat)	Other	Anx (bin)	1.326	0.975	1.804	-	-	-	0.0722	0.880	0.587	1.318	-	-	-	0.5328
6c	Attend (cat)	1/yr	Anx (bin)	0.926	0.809	1.060	-	-	-	0.2629	1.019	0.857	1.212	-	-	-	0.8303

**Model num.**	**RSBB expo-sure**	**RSBB expo-sure level**	**Out-come**	**Est (no adj)**	**LCI (no adj)**	**UCI (no adj)**	**Est (no adj; std)**	**LCI (no adj; std)**	**UCI (no adj; std)**	***p* (no adj)**	**Est (adj)**	**LCI (adj)**	**UCI (adj)**	**Est (adj; std)**	**LCI (adj; std)**	**UCI (adj; std)**	***p* (adj)**
6c	Attend (cat)	1/mth	Anx (bin)	0.930	0.765	1.130	-	-	-	0.4635	0.884	0.682	1.145	-	-	-	0.3490
6c	Attend (cat)	1/wk	Anx (bin)	0.806	0.666	0.976	-	-	-	0.0270	0.781	0.576	1.058	-	-	-	0.1106
7a	Belief (bin)	Yes	Dep (bin)	0.967	0.855	1.093	-	-	-	0.5885	0.970	0.804	1.171	-	-	-	0.7529
7b	Identity (bin)	Religious	Dep (bin)	0.911	0.774	1.072	-	-	-	0.2600	0.934	0.750	1.163	-	-	-	0.5398
7c	Attend (bin)	Regular	Dep (bin)	0.885	0.760	1.030	-	-	-	0.1137	0.942	0.758	1.170	-	-	-	0.5883
8a	Belief (bin)	Yes	Anx (bin)	1.007	0.903	1.123	-	-	-	0.9006	0.981	0.831	1.158	-	-	-	0.8204
8b	Identity (bin)	Religious	Anx (bin)	0.894	0.779	1.026	-	-	-	0.1121	0.901	0.729	1.114	-	-	-	0.3342
8c	Attend (bin)	Regular	Anx (bin)	0.885	0.773	1.013	-	-	-	0.0772	0.836	0.682	1.023	-	-	-	0.0822

Table S24: Predicted probabilities of the mothers logistic analyses with religious/spiritual belief and behaviour (RSBB) exposures and binary probable depression and anxiety as outcomes using multiple imputation (*n* = 13,085). These results indicate the difference in the predicted probability of a probable depression or anxiety diagnosis, based on the associated logistic regression model (see table S8). LCI = Lower 95% confidence interval; UCI = Upper 95% confidence interval; no adj = unadjusted model; adj = Adjusted model.

Model num.	RSBB expo-sure	RSBB expo-sure level	Out-come	Diff in pred prob (no adj)	LCI (no adj)	UCI (no adj)	Diff in pred prob (adj)	LCI (adj)	UCI (adj)
5a	Belief (cat)	Not sure	Dep (bin)	-0.49	-2.60	1.62	-0.58	-2.86	1.69
5a	Belief (cat)	Yes	Dep (bin)	-0.72	-2.81	1.37	-0.78	-3.61	2.05
5b	Identity (cat)	Christian	Dep (bin)	-1.46	-3.59	0.67	-0.59	-3.04	1.87
5b	Identity (cat)	Other	Dep (bin)	4.59	-0.05	9.23	-1.77	-5.48	1.94
5c	Attend (cat)	1/yr	Dep (bin)	-0.87	-2.63	0.89	0.48	-1.46	2.41
5c	Attend (cat)	1/mth	Dep (bin)	-0.52	-3.12	2.08	-0.12	-2.85	2.61
5c	Attend (cat)	1/wk	Dep (bin)	-2.88	-5.24	-0.52	-0.79	-4.10	2.52
6a	Belief (cat)	Not sure	Anx (bin)	-0.11	-2.37	2.16	-1.18	-3.72	1.36
6a	Belief (cat)	Yes	Anx (bin)	0.04	-2.24	2.32	-1.17	-4.21	1.86
6b	Identity (cat)	Christian	Anx (bin)	-2.04	-4.22	0.14	-1.26	-3.97	1.44
6b	Identity (cat)	Other	Anx (bin)	4.95	-0.70	10.59	-1.55	-6.46	3.37
6c	Attend (cat)	1/yr	Anx (bin)	-1.17	-3.21	0.87	0.24	-1.92	2.39
6c	Attend (cat)	1/mth	Anx (bin)	-1.10	-4.01	1.81	-1.47	-4.52	1.58
6c	Attend (cat)	1/wk	Anx (bin)	-3.13	-5.77	-0.49	-2.87	-6.26	0.53
7a	Belief (bin)	Yes	Dep (bin)	-0.42	-1.95	1.11	-0.32	-2.29	1.66
7b	Identity (bin)	Religious	Dep (bin)	-1.20	-3.32	0.92	-0.74	-3.11	1.63
7c	Attend (bin)	Regular	Dep (bin)	-1.48	-3.28	0.31	-0.62	-2.87	1.63
8a	Belief (bin)	Yes	Anx (bin)	0.11	-1.55	1.76	-0.23	-2.26	1.79
8b	Identity (bin)	Religious	Anx (bin)	-1.74	-3.92	0.45	-1.30	-3.97	1.37
8c	Attend (bin)	Regular	Anx (bin)	-1.80	-3.75	0.15	-2.13	-4.46	0.21

Table S25: Results of the partners analyses with religious/spiritual belief and behaviour (RSBB) exposures and depression and anxiety as outcomes using multiple imputation (*n* = 9,745). Est = Estimate (either mean difference from a linear model, or odds ratio from a logistic model); LCI = Lower 95% confidence interval; UCI = Upper 95% confidence interval; *p* = *p*-value; no adj = unadjusted model; adj = Adjusted model; std = model using standardised depression or anxiety scores.

Model num.	RSBB expo-sure	RSBB expo-sure level	Out-come	Est (no adj)	LCI (no adj)	UCI (no adj)	Est (no adj; std)	LCI (no adj; std)	UCI (no adj; std)	*p* (no adj)	Est (adj)	LCI (adj)	UCI (adj)	Est (adj; std)	LCI (adj; std)	UCI (adj; std)	*p* (adj)
1a	Belief (cat)	Not sure	Dep (cont)	0.253	-0.057	0.563	0.059	-0.011	0.128	0.1086	0.16	-0.21	0.53	0.04	-0.05	0.13	0.1086
1a	Belief (cat)	Yes	Dep (cont)	0.807	0.486	1.129	0.184	0.112	0.256	<0.0001	0.51	0.01	1.00	0.12	0.00	0.23	0.0448
1b	Identity (cat)	Christian	Dep (cont)	0.359	0.067	0.651	0.081	0.016	0.147	0.0162	0.17	-0.24	0.59	0.04	-0.05	0.13	0.4115
1b	Identity (cat)	Other	Dep (cont)	2.194	1.276	3.112	0.492	0.287	0.696	<0.0001	1.13	0.29	1.96	0.25	0.07	0.44	0.0085
1c	Attend (cat)	1/yr	Dep (cont)	-0.223	-0.542	0.097	-0.047	-0.119	0.025	0.1710	-0.39	-0.73	-0.04	-0.09	-0.17	-0.01	0.0302
1c	Attend (cat)	1/mth	Dep (cont)	0.084	-0.511	0.678	0.024	-0.109	0.157	0.7816	-0.26	-0.88	0.36	-0.06	-0.20	0.08	0.4010
1c	Attend (cat)	1/wk	Dep (cont)	0.130	-0.334	0.594	0.036	-0.069	0.140	0.5818	0.13	-0.61	0.87	0.03	-0.14	0.20	0.7273
2a	Belief (cat)	Not sure	Anx (cont)	0.119	-0.096	0.334	0.041	-0.031	0.114	0.2746	0.15	-0.10	0.40	0.05	-0.03	0.13	0.2353
2a	Belief (cat)	Yes	Anx (cont)	0.540	0.329	0.750	0.185	0.114	0.256	<0.0001	0.43	0.12	0.73	0.15	0.04	0.25	0.0064
2b	Identity (cat)	Christian	Anx (cont)	0.137	-0.045	0.319	0.047	-0.015	0.108	0.1387	0.25	-0.02	0.51	0.08	-0.01	0.17	0.0721
2b	Identity (cat)	Other	Anx (cont)	1.560	0.971	2.149	0.529	0.330	0.728	<0.0001	0.84	0.24	1.45	0.29	0.08	0.49	0.0070
2c	Attend (cat)	1/yr	Anx (cont)	0.006	-0.226	0.237	0.004	-0.075	0.082	0.9614	-0.14	-0.37	0.09	-0.05	-0.13	0.03	0.2369
2c	Attend (cat)	1/mth	Anx (cont)	-0.033	-0.398	0.333	-0.009	-0.132	0.115	0.8608	-0.20	-0.61	0.21	-0.07	-0.21	0.07	0.3335
2c	Attend (cat)	1/wk	Anx (cont)	0.360	0.031	0.689	0.126	0.014	0.237	0.0321	0.38	-0.07	0.84	0.13	-0.02	0.29	0.0995
3a	Belief (bin)	Yes	Dep (cont)	0.678	0.396	0.959	0.154	0.091	0.218	<0.0001	0.38	-0.01	0.77	0.09	0.00	0.17	0.0532

Model num.	RSBB expo-sure	RSBB expo-sure level	Out-come	Est (no adj)	LCI (no adj)	UCI (no adj)	Est (no adj; std)	LCI (no adj; std)	UCI (no adj; std)	*p* (no adj)	Est (adj)	LCI (adj)	UCI (adj)	Est (adj; std)	LCI (adj; std)	UCI (adj; std)	*p* (adj)
3b	Identity (bin)	Religious	Dep (cont)	0.450	0.152	0.748	0.101	0.035	0.168	0.0033	0.26	-0.15	0.67	0.06	-0.03	0.15	0.2160
3c	Attend (bin)	Regular	Dep (cont)	0.161	-0.228	0.550	0.041	-0.046	0.128	0.4155	0.06	-0.47	0.59	0.01	-0.11	0.13	0.8248
4a	Belief (bin)	Yes	Anx (cont)	0.479	0.299	0.659	0.164	0.103	0.224	<0.0001	0.32	0.08	0.55	0.11	0.03	0.19	0.0097
4b	Identity (bin)	Religious	Anx (cont)	0.207	0.021	0.394	0.071	0.007	0.134	0.0294	0.30	0.03	0.57	0.10	0.01	0.19	0.0305
4c	Attend (bin)	Regular	Anx (cont)	0.185	-0.062	0.432	0.065	-0.018	0.149	0.1415	0.07	-0.25	0.39	0.02	-0.09	0.13	0.6721
5a	Belief (cat)	Not sure	Dep (bin)	1.054	0.877	1.267	-	-	-	0.5729	1.01	0.76	1.34	-	-	-	0.9355
5a	Belief (cat)	Yes	Dep (bin)	1.412	1.171	1.704	-	-	-	0.0004	1.29	0.89	1.85	-	-	-	0.1770
5b	Identity (cat)	Christian	Dep (bin)	1.190	0.993	1.427	-	-	-	0.0591	1.10	0.80	1.50	-	-	-	0.5524
5b	Identity (cat)	Other	Dep (bin)	2.405	1.641	3.526	-	-	-	<0.0001	1.70	1.01	2.86	-	-	-	0.0459
5c	Attend (cat)	1/yr	Dep (bin)	0.863	0.703	1.059	-	-	-	0.1580	0.78	0.60	1.02	-	-	-	0.0654
5c	Attend (cat)	1/mth	Dep (bin)	0.976	0.702	1.358	-	-	-	0.8862	0.80	0.51	1.25	-	-	-	0.3232
5c	Attend (cat)	1/wk	Dep (bin)	1.028	0.774	1.365	-	-	-	0.8493	1.05	0.63	1.74	-	-	-	0.8622
6a	Belief (cat)	Not sure	Anx (bin)	1.100	0.926	1.308	-	-	-	0.2761	1.14	0.89	1.47	-	-	-	0.3039
6a	Belief (cat)	Yes	Anx (bin)	1.474	1.255	1.731	-	-	-	<0.0001	1.43	1.05	1.94	-	-	-	0.0223
6b	Identity (cat)	Christian	Anx (bin)	1.149	0.999	1.323	-	-	-	0.0524	1.27	0.99	1.64	-	-	-	0.0640
6b	Identity (cat)	Other	Anx (bin)	2.461	1.711	3.541	-	-	-	<0.0001	1.85	1.08	3.16	-	-	-	0.0245
6c	Attend (cat)	1/yr	Anx (bin)	1.012	0.835	1.226	-	-	-	0.9028	0.89	0.68	1.16	-	-	-	0.3655

Model num.	RSBB expo-sure	RSBB expo-sure level	Out-come	Est (no adj)	LCI (no adj)	UCI (no adj)	Est (no adj; std)	LCI (no adj; std)	UCI (no adj; std)	*p* (no adj)	Est (adj)	LCI (adj)	UCI (adj)	Est (adj; std)	LCI (adj; std)	UCI (adj; std)	*p* (adj)
6c	Attend (cat)	1/mth	Anx (bin)	1.001	0.744	1.347	-	-	-	0.9947	0.83	0.53	1.29	-	-	-	0.4052
6c	Attend (cat)	1/wk	Anx (bin)	1.294	1.025	1.634	-	-	-	0.0305	1.23	0.78	1.95	-	-	-	0.3721
7a	Belief (bin)	Yes	Dep (bin)	1.374	1.169	1.615	-	-	-	0.0001	1.27	0.97	1.67	-	-	-	0.0788
7b	Identity (bin)	Religious	Dep (bin)	1.243	1.036	1.492	-	-	-	0.0195	1.16	0.85	1.58	-	-	-	0.3643
7c	Attend (bin)	Regular	Dep (bin)	1.039	0.829	1.303	-	-	-	0.7366	1.00	0.70	1.41	-	-	-	0.9809
8a	Belief (bin)	Yes	Anx (bin)	1.402	1.231	1.598	-	-	-	<0.0001	1.29	1.02	1.63	-	-	-	0.0318
8b	Identity (bin)	Religious	Anx (bin)	1.201	1.041	1.386	-	-	-	0.0121	1.32	1.02	1.72	-	-	-	0.0347
8c	Attend (bin)	Regular	Anx (bin)	1.157	0.965	1.387	-	-	-	0.1156	1.02	0.73	1.41	-	-	-	0.9247

Table S26: Predicted probabilities of the partners logistic analyses with religious/spiritual belief and behaviour (RSBB) exposures and binary probable depression and anxiety as outcomes using multiple imputation (*n* = 9,745). These results indicate the difference in the predicted probability of a probable depression or anxiety diagnosis, based on the associated logistic regression model (see table S25). LCI = Lower 95% confidence interval; UCI = Upper 95% confidence interval; no adj = unadjusted model; adj = Adjusted model.

**Model num.**	**RSBB expo-sure**	**RSBB expo-sure level**	**Out-come**	**Diff in pred prob (no adj)**	**LCI (no adj)**	**UCI (no adj)**	**Diff in pred prob (adj)**	**LCI (adj)**	**UCI (adj)**
5a	Belief (cat)	Not sure	Dep (bin)	0.61	-1.52	2.75	0.11	-2.62	2.83
5a	Belief (cat)	Yes	Dep (bin)	4.46	2.06	6.86	2.57	-1.17	6.31
5b	Identity (cat)	Christian	Dep (bin)	2.07	-0.01	4.15	0.91	-2.15	3.97
5b	Identity (cat)	Other	Dep (bin)	13.36	6.56	20.15	5.90	-0.32	12.13
5c	Attend (cat)	1/yr	Dep (bin)	-1.79	-4.21	0.64	-2.44	-4.96	0.08
5c	Attend (cat)	1/mth	Dep (bin)	-0.27	-4.43	3.89	-2.13	-6.32	2.05
5c	Attend (cat)	1/wk	Dep (bin)	0.38	-3.32	4.09	0.54	-4.87	5.95
6a	Belief (cat)	Not sure	Anx (bin)	1.59	-1.27	4.46	1.73	-1.57	5.03
6a	Belief (cat)	Yes	Anx (bin)	7.02	4.15	9.89	4.88	0.72	9.03
6b	Identity (cat)	Christian	Anx (bin)	2.38	0.02	4.74	3.17	-0.12	6.46
6b	Identity (cat)	Other	Anx (bin)	18.52	10.28	26.75	8.84	0.68	17.00
6c	Attend (cat)	1/yr	Anx (bin)	0.22	-3.17	3.62	-1.62	-5.15	1.90
6c	Attend (cat)	1/mth	Anx (bin)	0.06	-5.15	5.26	-2.43	-8.13	3.28
6c	Attend (cat)	1/wk	Anx (bin)	4.87	0.27	9.47	3.02	-3.70	9.74
7a	Belief (bin)	Yes	Dep (bin)	4.15	1.99	6.30	2.49	-0.33	5.30
7b	Identity (bin)	Religious	Dep (bin)	2.63	0.52	4.73	1.40	-1.63	4.42
7c	Attend (bin)	Regular	Dep (bin)	0.51	-2.37	3.39	-0.01	-3.52	3.50
8a	Belief (bin)	Yes	Anx (bin)	6.20	3.75	8.66	3.54	0.28	6.79
8b	Identity (bin)	Religious	Anx (bin)	3.18	0.77	5.59	3.71	0.35	7.06
8c	Attend (bin)	Regular	Anx (bin)	2.68	-0.73	6.10	0.25	-4.26	4.75

Table S27: Results of the interaction analyses assessing whether the adjusted mother and partner results differ, with religious/spiritual belief and behaviour (RSBB) exposures and standardised depression and anxiety scores as outcomes using multiple imputation. SE = Standard error; LCI = Lower 95% confidence interval; UCI = Upper 95% confidence interval; *p* = *p*-value.

Model num.	Exposure	Exposure level	Outcome	Mother coef.	Mother SE	Partner coef.	Partner SE	Mother vs partner diff.	Diff. SE	Diff. LCI	Diff. UCI	Diff. *p*
1a	Belief (cat)	Not sure	Dep (cont)	0.007	0.034	0.036	0.044	-0.029	0.056	-0.139	0.081	0.6047
1a	Belief (cat)	Yes	Dep (cont)	-0.002	0.039	0.115	0.056	-0.117	0.069	-0.251	0.017	0.0880
1b	Identity (cat)	Christian	Dep (cont)	-0.012	0.036	0.039	0.047	-0.051	0.060	-0.168	0.066	0.3917
1b	Identity (cat)	Other	Dep (cont)	-0.054	0.065	0.253	0.095	-0.307	0.115	-0.533	-0.081	0.0077
1c	Attend (cat)	1/yr	Dep (cont)	0.007	0.027	-0.087	0.040	0.094	0.048	0.000	0.188	0.0501
1c	Attend (cat)	1/mth	Dep (cont)	-0.027	0.039	-0.059	0.071	0.032	0.081	-0.126	0.190	0.6917
1c	Attend (cat)	1/wk	Dep (cont)	-0.048	0.046	0.030	0.084	-0.078	0.096	-0.266	0.110	0.4166
2a	Belief (cat)	Not sure	Anx (cont)	-0.010	0.032	0.051	0.043	-0.061	0.053	-0.165	0.043	0.2517
2a	Belief (cat)	Yes	Anx (cont)	-0.014	0.038	0.146	0.053	-0.160	0.065	-0.287	-0.033	0.0138
2b	Identity (cat)	Christian	Anx (cont)	-0.037	0.035	0.083	0.046	-0.120	0.058	-0.234	-0.006	0.0388
2b	Identity (cat)	Other	Anx (cont)	-0.039	0.070	0.286	0.105	-0.325	0.126	-0.572	-0.078	0.0098
2c	Attend (cat)	1/yr	Anx (cont)	0.011	0.027	-0.047	0.040	0.058	0.048	-0.036	0.152	0.2280
2c	Attend (cat)	1/mth	Anx (cont)	-0.054	0.041	-0.068	0.071	0.014	0.082	-0.147	0.175	0.8645
2c	Attend (cat)	1/wk	Anx (cont)	-0.082	0.046	0.131	0.079	-0.213	0.092	-0.393	-0.033	0.0202
3a	Belief (bin)	Yes	Dep (cont)	-0.008	0.028	0.087	0.044	-0.095	0.052	-0.198	0.008	0.0697
3b	Identity (bin)	Religious	Dep (cont)	-0.016	0.035	0.058	0.047	-0.074	0.059	-0.189	0.041	0.2072
3c	Attend (bin)	Regular	Dep (cont)	-0.039	0.031	0.014	0.060	-0.053	0.068	-0.187	0.081	0.4365
4a	Belief (bin)	Yes	Anx (cont)	-0.006	0.027	0.108	0.041	-0.114	0.049	-0.210	-0.018	0.0204
4b	Identity (bin)	Religious	Anx (cont)	-0.037	0.035	0.101	0.047	-0.138	0.058	-0.252	-0.024	0.0180
4c	Attend (bin)	Regular	Anx (cont)	-0.070	0.032	0.024	0.056	-0.094	0.064	-0.220	0.032	0.1439

Table S28: Results of the interaction analyses assessing whether the adjusted mother and partner results differ, with religious/spiritual belief and behaviour (RSBB) exposures and probable depression and anxiety diagnoses as outcomes using multiple imputation. OR = Odds ratio; SE = Standard error; LCI = Lower 95% confidence interval; UCI = Upper 95% confidence interval; *p* = *p*-value.

Model num.	Exposure	Expo-sure level	Outcome	Mother OR	Mother log-odds	Mother log-odds SE	Partner OR	Partner log-odds	Partner log-odds SE	Log-odds diff.	Diff. log-odds SE	Diff. OR	Diff. OR LCI	Diff. OR UCI	Diff. *p*
5a	Belief (cat)	Not sure	Dep (bin)	0.947	-0.054	0.108	1.012	0.012	0.145	-0.066	0.180	0.936	0.657	1.332	0.7127
5a	Belief (cat)	Yes	Dep (bin)	0.930	-0.073	0.135	1.285	0.251	0.186	-0.323	0.230	0.724	0.461	1.135	0.1594
5b	Identity (cat)	Christian	Dep (bin)	0.947	-0.054	0.116	1.099	0.094	0.160	-0.149	0.198	0.862	0.585	1.270	0.4517
5b	Identity (cat)	Other	Dep (bin)	0.842	-0.172	0.188	1.699	0.530	0.265	-0.702	0.325	0.496	0.262	0.938	0.0309
5c	Attend (cat)	1/yr	Dep (bin)	1.045	0.044	0.092	0.777	-0.252	0.136	0.296	0.165	1.345	0.974	1.857	0.0718
5c	Attend (cat)	1/mth	Dep (bin)	0.988	-0.012	0.133	0.800	-0.223	0.226	0.211	0.262	1.235	0.739	2.065	0.4208
5c	Attend (cat)	1/wk	Dep (bin)	0.924	-0.079	0.166	1.046	0.045	0.259	-0.124	0.308	0.883	0.483	1.616	0.6874
6a	Belief (cat)	Not sure	Anx (bin)	0.909	-0.095	0.104	1.142	0.133	0.129	-0.228	0.166	0.796	0.575	1.102	0.1690
6a	Belief (cat)	Yes	Anx (bin)	0.910	-0.094	0.124	1.427	0.356	0.155	-0.450	0.199	0.638	0.432	0.942	0.0238
6b	Identity (cat)	Christian	Anx (bin)	0.904	-0.101	0.110	1.272	0.241	0.130	-0.342	0.170	0.711	0.509	0.992	0.0447
6b	Identity (cat)	Other	Anx (bin)	0.880	-0.128	0.206	1.850	0.615	0.273	-0.743	0.342	0.476	0.243	0.930	0.0299
6c	Attend (cat)	1/yr	Anx (bin)	1.019	0.019	0.088	0.885	-0.122	0.136	0.141	0.162	1.151	0.838	1.582	0.3845
6c	Attend (cat)	1/mth	Anx (bin)	0.884	-0.123	0.132	0.829	-0.188	0.228	0.064	0.263	1.066	0.636	1.787	0.8072
2c	Attend (cat)	1/wk	Anx (bin)	0.781	-0.247	0.155	1.231	0.208	0.234	-0.455	0.281	0.634	0.366	1.100	0.1052
7a	Belief (bin)	Yes	Dep (bin)	0.970	-0.030	0.096	1.273	0.241	0.138	-0.272	0.168	0.762	0.548	1.059	0.1051
7b	Identity (bin)	Religious	Dep (bin)	0.934	-0.068	0.112	1.155	0.144	0.159	-0.212	0.195	0.809	0.552	1.184	0.2754
7c	Attend (bin)	Regular	Dep (bin)	0.942	-0.060	0.111	0.996	-0.004	0.178	-0.056	0.210	0.946	0.627	1.427	0.7907
8a	Belief (bin)	Yes	Anx (bin)	0.981	-0.019	0.085	1.290	0.255	0.118	-0.274	0.146	0.760	0.572	1.011	0.0599
8b	Identity (bin)	Religious	Anx (bin)	0.901	-0.104	0.108	1.324	0.281	0.133	-0.385	0.171	0.681	0.486	0.952	0.0246
8c	Attend (bin)	Regular	Anx (bin)	0.836	-0.179	0.103	1.016	0.016	0.168	-0.195	0.197	0.823	0.559	1.211	0.3228

Table S29: Results of the mothers analyses with depression and anxiety exposures and religious/spiritual belief and behaviour (RSBB) as outcomes using multiple imputation (*n* = 13,150). Est = Estimate (either relative risk ratio from a multinomial model, or odds ratio from a logistic model); LCI = Lower 95% confidence interval; UCI = Upper 95% confidence interval; *p* = *p*-value; no adj = unadjusted model; adj = Adjusted model; std = model using standardised depression or anxiety scores.

**Model num.**	**Mental health expo-sure**	**RSBB out-come**	**RSBB out-come level**	**Est (no adj)**	**LCI (no adj)**	**UCI (no adj)**	**Est (no adj; std)**	**LCI (no adj; std)**	**UCI (no adj; std)**	***p* (no adj)**	**Est (adj)**	**LCI (adj)**	**UCI (adj)**	**Est (adj; std)**	**LCI (adj; std)**	**UCI (adj; std)**	***p* (adj)**
1a	Dep (cont)	Belief (cat)	Not sure	0.992	0.981	1.004	0.965	0.913	1.020	0.1875	0.990	0.972	1.007	0.951	0.874	1.035	0.2486
1a	Dep (cont)	Belief (cat)	Yes	0.989	0.978	0.999	0.948	0.901	0.997	0.0328	0.987	0.968	1.007	0.939	0.855	1.033	0.1910
1b	Anx (cont)	Belief (cat)	Not sure	0.997	0.981	1.013	0.991	0.939	1.047	0.7201	0.986	0.962	1.010	0.953	0.878	1.034	0.2507
1b	Anx (cont)	Belief (cat)	Yes	0.994	0.980	1.009	0.983	0.935	1.032	0.4466	0.978	0.951	1.006	0.928	0.845	1.020	0.1167
2a	Dep (cont)	Identity (cat)	Christian	0.988	0.977	0.999	0.945	0.896	0.997	0.0347	0.994	0.975	1.013	0.969	0.885	1.061	0.5184
2a	Dep (cont)	Identity (cat)	Other	1.036	1.011	1.062	1.184	1.053	1.332	0.0046	0.989	0.954	1.025	0.943	0.795	1.118	0.5314
2b	Anx (cont)	Identity (cat)	Christian	0.968	0.953	0.984	0.899	0.853	0.947	0.0001	0.970	0.946	0.995	0.902	0.829	0.982	0.0192
2b	Anx (cont)	Identity (cat)	Other	1.055	1.021	1.090	1.196	1.072	1.334	0.0014	0.986	0.938	1.038	0.958	0.810	1.134	0.5967
3a	Dep (cont)	Attend (cat)	1/yr	0.976	0.966	0.986	0.891	0.847	0.937	<0.0001	0.984	0.969	0.999	0.925	0.859	0.996	0.0397
3a	Dep (cont)	Attend (cat)	1/mth	0.984	0.969	0.998	0.926	0.862	0.994	0.0281	0.982	0.961	1.004	0.922	0.829	1.024	0.1112
3a	Dep (cont)	Attend (cat)	1/wk	0.985	0.970	1.000	0.932	0.867	1.002	0.0506	0.999	0.971	1.028	1.001	0.874	1.148	0.9430
3b	Anx (cont)	Attend (cat)	1/yr	0.974	0.959	0.989	0.917	0.872	0.964	0.0006	0.979	0.956	1.002	0.933	0.863	1.008	0.0719
3b	Anx (cont)	Attend (cat)	1/mth	0.986	0.965	1.007	0.955	0.890	1.024	0.1760	0.980	0.948	1.012	0.936	0.839	1.043	0.2164
3b	Anx (cont)	Attend (cat)	1/wk	0.986	0.966	1.007	0.956	0.891	1.026	0.1950	1.001	0.958	1.045	1.004	0.869	1.160	0.9809
4a	Dep (bin)	Belief (cat)	Not sure	0.870	0.731	1.035	-	-	-	0.1165	0.891	0.705	1.127	-	-	-	0.3358

Model num.	Mental health expo-sure	RSBB out-come	RSBB out-come level	Est (no adj)	LCI (no adj)	UCI (no adj)	Est (no adj; std)	LCI (no adj; std)	UCI (no adj; std)	*p* (no adj)	Est (adj)	LCI (adj)	UCI (adj)	Est (adj; std)	LCI (adj; std)	UCI (adj; std)	*p* (adj)
4a	Dep (bin)	Belief (cat)	Yes	0.829	0.701	0.981	-	-	-	0.0288	0.869	0.653	1.156	-	-	-	0.3350
4b	Anx (bin)	Belief (cat)	Not sure	0.925	0.777	1.101	-	-	-	0.3790	0.901	0.720	1.127	-	-	-	0.3610
4b	Anx (bin)	Belief (cat)	Yes	0.911	0.774	1.073	-	-	-	0.2659	0.903	0.693	1.177	-	-	-	0.4502
5a	Dep (bin)	Identity (cat)	Christian	0.792	0.667	0.939	-	-	-	0.0074	0.856	0.666	1.100	-	-	-	0.2225
5a	Dep (bin)	Identity (cat)	Other	1.551	1.113	2.162	-	-	-	0.0097	0.904	0.585	1.395	-	-	-	0.6464
5b	Anx (bin)	Identity (cat)	Christian	0.761	0.644	0.899	-	-	-	0.0014	0.798	0.629	1.014	-	-	-	0.0645
5b	Anx (bin)	Identity (cat)	Other	1.633	1.180	2.259	-	-	-	0.0032	0.989	0.641	1.524	-	-	-	0.9588
6a	Dep (bin)	Attend (cat)	1/yr	0.704	0.595	0.834	-	-	-	0.0001	0.816	0.656	1.016	-	-	-	0.0695
6a	Dep (bin)	Attend (cat)	1/mth	0.860	0.680	1.088	-	-	-	0.2078	0.943	0.695	1.281	-	-	-	0.7078
6a	Dep (bin)	Attend (cat)	1/wk	0.779	0.609	0.997	-	-	-	0.0474	0.967	0.652	1.433	-	-	-	0.8672
6b	Anx (bin)	Attend (cat)	1/yr	0.768	0.648	0.909	-	-	-	0.0022	0.867	0.693	1.086	-	-	-	0.2142
6b	Anx (bin)	Attend (cat)	1/mth	0.809	0.641	1.022	-	-	-	0.0748	0.849	0.621	1.161	-	-	-	0.3043
6b	Anx (bin)	Attend (cat)	1/wk	0.870	0.689	1.099	-	-	-	0.2415	1.053	0.707	1.570	-	-	-	0.7977
7a	Dep (cont)	Belief (bin)	Yes	0.993	0.985	1.001	0.969	0.931	1.008	0.1056	0.995	0.981	1.010	0.976	0.910	1.047	0.5015
7b	Anx (cont)	Belief (bin)	Yes	0.996	0.985	1.008	0.988	0.950	1.027	0.5073	0.989	0.969	1.010	0.964	0.900	1.032	0.2919
8a	Dep (cont)	Identity (bin)	Religious	0.990	0.979	1.002	0.955	0.906	1.008	0.0905	0.993	0.975	1.012	0.969	0.887	1.057	0.4753
8b	Anx (cont)	Identity (bin)	Religious	0.973	0.958	0.988	0.912	0.866	0.962	0.0007	0.973	0.949	0.998	0.912	0.838	0.993	0.0341

**Model num.**	**Mental health expo-sure**	**RSBB out-come**	**RSBB out-come level**	**Est (no adj)**	**LCI (no adj)**	**UCI (no adj)**	**Est (no adj; std)**	**LCI (no adj; std)**	**UCI (no adj; std)**	***p* (no adj)**	**Est (adj)**	**LCI (adj)**	**UCI (adj)**	**Est (adj; std)**	**LCI (adj; std)**	**UCI (adj; std)**	***p* (adj)**
9a	Dep (cont)	Attend (bin)	Regular	0.991	0.981	1.002	0.961	0.912	1.012	0.1099	0.998	0.980	1.016	0.990	0.907	1.080	0.8077
9b	Anx (cont)	Attend (bin)	Regular	0.993	0.978	1.009	0.980	0.931	1.031	0.3906	0.998	0.972	1.026	0.995	0.910	1.088	0.9075
10a	Dep (bin)	Belief (bin)	Yes	0.901	0.789	1.029	-	-	-	0.1249	0.950	0.773	1.168	-	-	-	0.6255
10b	Anx (bin)	Belief (bin)	Yes	0.955	0.839	1.088	-	-	-	0.4905	0.980	0.797	1.204	-	-	-	0.8460
11a	Dep (bin)	Identity (bin)	Religious	0.822	0.693	0.975	-	-	-	0.0244	0.871	0.682	1.111	-	-	-	0.2649
11b	Anx (bin)	Identity (bin)	Religious	0.795	0.673	0.940	-	-	-	0.0073	0.833	0.657	1.057	-	-	-	0.1321
12a	Dep (bin)	Attend (bin)	Regular	0.900	0.758	1.069	-	-	-	0.2312	1.051	0.816	1.355	-	-	-	0.6971
12b	Anx (bin)	Attend (bin)	Regular	0.905	0.764	1.072	-	-	-	0.2475	0.988	0.767	1.272	-	-	-	0.9237

Table S30: Predicted probabilities of the mothers multinomial and logistic regression analyses with depression and anxiety exposures and religious/spiritual belief and behaviour (RSBB) outcomes using multiple imputation (*n* = 13,150). These results indicate the change in the predicted probability of the RSBB outcome, based on either a standardised one-unit increase in depression or anxiety score (if exposure continuous) or the difference if a probable depression or anxiety diagnosis (if exposure binary). For the associated multinomial or logistic regression models, see table S29. LCI = Lower 95% confidence interval; UCI = Upper 95% confidence interval; no adj = unadjusted model; adj = Adjusted model.

**Model num.**	**Mental health exposure**	**RSBB outcome**	**RSBB outcome level**	**Est (no adj)**	**LCI (no adj)**	**UCI (no adj)**	**Est (no adj; std)**	**LCI (no adj; std)**	**UCI (no adj; std)**	**Est (adj)**	**LCI (adj)**	**UCI (adj)**	**Est (adj; std)**	**LCI (adj; std)**	**UCI (adj; std)**
1a	Dep (cont)	Belief (cat)	No	0.170	0.000	0.330	0.770	-0.030	1.570	0.110	-0.060	0.280	0.540	-0.270	1.350
1a	Dep (cont)	Belief (cat)	Not sure	0.000	-0.200	0.200	0.020	-0.950	0.980	-0.050	-0.280	0.180	-0.240	-1.340	0.860
1a	Dep (cont)	Belief (cat)	Yes	-0.170	-0.370	0.040	-0.780	-1.770	0.200	-0.060	-0.250	0.130	-0.300	-1.220	0.620
1b	Anx (cont)	Belief (cat)	No	0.080	-0.160	0.310	0.230	-0.550	1.010	0.170	-0.070	0.410	0.550	-0.240	1.350
1b	Anx (cont)	Belief (cat)	Not sure	0.020	-0.270	0.310	0.070	-0.880	1.030	-0.020	-0.340	0.300	-0.080	-1.140	0.990
1b	Anx (cont)	Belief (cat)	Yes	-0.100	-0.380	0.190	-0.300	-1.260	0.660	-0.150	-0.420	0.130	-0.480	-1.390	0.430
2a	Dep (cont)	Identity (cat)	None	0.140	-0.020	0.290	0.650	-0.090	1.380	0.060	-0.100	0.210	0.290	-0.450	1.020
2a	Dep (cont)	Identity (cat)	Christian	-0.300	-0.460	-0.140	-1.410	-2.180	-0.640	-0.040	-0.200	0.130	-0.190	-0.990	0.610
2a	Dep (cont)	Identity (cat)	Other	0.160	0.080	0.240	0.760	0.380	1.140	-0.020	-0.110	0.070	-0.100	-0.530	0.330
2b	Anx (cont)	Identity (cat)	None	0.390	0.170	0.600	1.290	0.570	2.010	0.240	0.030	0.450	0.810	0.100	1.520
2b	Anx (cont)	Identity (cat)	Christian	-0.670	-0.890	-0.440	-2.230	-2.980	-1.470	-0.260	-0.480	-0.040	-0.880	-1.610	-0.150
2b	Anx (cont)	Identity (cat)	Other	0.280	0.170	0.390	0.940	0.580	1.290	0.020	-0.100	0.140	0.070	-0.340	0.470
3a	Dep (cont)	Attend (cat)	Never	0.510	0.300	0.710	2.380	1.380	3.380	0.220	0.010	0.430	1.020	0.020	2.030
3a	Dep (cont)	Attend (cat)	1/yr	-0.380	-0.570	-0.190	-1.810	-2.710	-0.900	-0.190	-0.410	0.020	-0.950	-1.990	0.090
3a	Dep (cont)	Attend (cat)	1/mth	-0.070	-0.180	0.050	-0.310	-0.860	0.240	-0.070	-0.210	0.060	-0.330	-0.980	0.320
3a	Dep (cont)	Attend (cat)	1/wk	-0.060	-0.180	0.070	-0.270	-0.870	0.330	0.050	-0.080	0.170	0.260	-0.340	0.850
3b	Anx (cont)	Attend (cat)	Never	0.520	0.220	0.820	1.690	0.690	2.690	0.270	-0.050	0.600	0.890	-0.190	1.980
3b	Anx (cont)	Attend (cat)	1/yr	-0.420	-0.690	-0.160	-1.400	-2.300	-0.500	-0.270	-0.580	0.050	-0.870	-1.930	0.180
3b	Anx (cont)	Attend (cat)	1/mth	-0.050	-0.210	0.120	-0.150	-0.700	0.410	-0.080	-0.280	0.120	-0.250	-0.910	0.410
3b	Anx (cont)	Attend (cat)	1/wk	-0.050	-0.220	0.130	-0.140	-0.720	0.440	0.070	-0.120	0.250	0.230	-0.380	0.840
4a	Dep (bin)	Belief (cat)	No	2.900	0.140	5.670	-	-	-	1.240	-1.140	3.630	-	-	-

Model num.	Mental health exposure	RSBB outcome	RSBB outcome level	Est (no adj)	LCI (no adj)	UCI (no adj)	Est (no adj; std)	LCI (no adj; std)	UCI (no adj; std)	Est (adj)	LCI (adj)	UCI (adj)	Est (adj; std)	LCI (adj; std)	UCI (adj; std)
4a	Dep (bin)	Belief (cat)	Not sure	-0.340	-3.420	2.730	-	-	-	-0.590	-3.600	2.420	-	-	-
4a	Dep (bin)	Belief (cat)	Yes	-2.560	-5.820	0.700	-	-	-	-0.650	-3.370	2.060	-	-	-
4b	Anx (bin)	Belief (cat)	No	1.480	-1.150	4.100	-	-	-	1.050	-1.160	3.260	-	-	-
4b	Anx (bin)	Belief (cat)	Not sure	-0.350	-3.430	2.720	-	-	-	-0.790	-3.860	2.290	-	-	-
4b	Anx (bin)	Belief (cat)	Yes	-1.120	-4.320	2.070	-	-	-	-0.270	-2.960	2.430	-	-	-
5a	Dep (bin)	Identity (cat)	None	2.840	0.250	5.440	-	-	-	1.290	-0.840	3.420	-	-	-
5a	Dep (bin)	Identity (cat)	Christian	-5.620	-8.410	-2.820	-	-	-	-1.310	-3.580	0.970	-	-	-
5a	Dep (bin)	Identity (cat)	Other	2.770	1.180	4.360	-	-	-	0.010	-1.070	1.090	-	-	-
5b	Anx (bin)	Identity (cat)	None	3.350	0.770	5.930	-	-	-	1.750	-0.340	3.840	-	-	-
5b	Anx (bin)	Identity (cat)	Christian	-6.570	-9.360	-3.780	-	-	-	-2.130	-4.290	0.030	-	-	-
5b	Anx (bin)	Identity (cat)	Other	3.220	1.580	4.860	-	-	-	0.380	-0.740	1.490	-	-	-
6a	Dep (bin)	Attend (cat)	Never	6.800	3.680	9.910	-	-	-	2.280	-0.550	5.100	-	-	-
6a	Dep (bin)	Attend (cat)	1/yr	-5.300	-7.970	-2.630	-	-	-	-2.790	-5.750	0.180	-	-	-
6a	Dep (bin)	Attend (cat)	1/mth	-0.310	-2.110	1.490	-	-	-	0.250	-1.700	2.200	-	-	-
6a	Dep (bin)	Attend (cat)	1/wk	-1.190	-3.060	0.680	-	-	-	0.260	-1.540	2.060	-	-	-
6b	Anx (bin)	Attend (cat)	Never	5.400	2.160	8.640	-	-	-	1.770	-1.250	4.790	-	-	-
6b	Anx (bin)	Attend (cat)	1/yr	-3.970	-6.700	-1.230	-	-	-	-1.750	-4.740	1.240	-	-	-
6b	Anx (bin)	Attend (cat)	1/mth	-1.000	-2.670	0.670	-	-	-	-0.760	-2.560	1.050	-	-	-
6b	Anx (bin)	Attend (cat)	1/wk	-0.430	-2.280	1.410	-	-	-	0.740	-1.060	2.530	-	-	-
7a	Dep (cont)	Belief (bin)	Yes	-0.170	-0.380	0.040	-0.790	-1.780	0.200	-0.070	-0.260	0.130	-0.320	-1.230	0.600
7b	Anx (cont)	Belief (bin)	Yes	-0.100	-0.380	0.190	-0.300	-1.260	0.660	-0.140	-0.410	0.120	-0.490	-1.390	0.410
8a	Dep (cont)	Identity (bin)	Religious	-0.130	-0.290	0.020	-0.630	-1.370	0.110	-0.060	-0.210	0.100	-0.270	-1.010	0.470
8b	Anx (cont)	Identity (bin)	Religious	-0.380	-0.600	-0.160	-1.260	-1.990	-0.540	-0.230	-0.450	-0.020	-0.780	-1.490	-0.060
9a	Dep (cont)	Attend (bin)	Regular	-0.130	-0.290	0.030	-0.590	-1.360	0.170	-0.020	-0.180	0.140	-0.090	-0.850	0.670
9b	Anx (cont)	Attend (bin)	Regular	-0.100	-0.320	0.130	-0.300	-1.050	0.450	-0.010	-0.250	0.220	-0.040	-0.820	0.730
10a	Dep (bin)	Belief (bin)	Yes	-2.560	-5.820	0.700	-	-	-	-0.670	-3.370	2.030	-	-	-

Model num.	Mental health exposure	RSBB outcome	RSBB outcome level	Est (no adj)	LCI (no adj)	UCI (no adj)	Est (no adj; std)	LCI (no adj; std)	UCI (no adj; std)	Est (adj)	LCI (adj)	UCI (adj)	Est (adj; std)	LCI (adj; std)	UCI (adj; std)
10b	Anx (bin)	Belief (bin)	Yes	-1.120	-4.320	2.070	-	-	-	-0.270	-2.970	2.430	-	-	-
11a	Dep (bin)	Identity (bin)	Religious	-2.840	-5.440	-0.250	-	-	-	-1.200	-3.340	0.940	-	-	-
11b	Anx (bin)	Identity (bin)	Religious	-3.350	-5.930	-0.770	-	-	-	-1.590	-3.700	0.520	-	-	-
12a	Dep (bin)	Attend (bin)	Regular	-1.500	-3.910	0.900	-	-	-	0.450	-1.790	2.680	-	-	-
12b	Anx (bin)	Attend (bin)	Regular	-1.430	-3.810	0.940	-	-	-	-0.100	-2.290	2.090	-	-	-

Table S31: Results of the partners analyses with depression and anxiety exposures and religious/spiritual belief and behaviour (RSBB) as outcomes using multiple imputation (*n* = 9,887). Est = Estimate (either relative risk ratio from a multinomial model, or odds ratio from a logistic model); LCI = Lower 95% confidence interval; UCI = Upper 95% confidence interval; *p* = *p*-value; no adj = unadjusted model; adj = Adjusted model; std = model using standardised depression or anxiety scores.

Model num.	Mental health expo-sure	RSBB out-come	RSBB out-come level	Est (no adj)	LCI (no adj)	UCI (no adj)	Est (no adj; std)	LCI (no adj; std)	UCI (no adj; std)	*p* (no adj)	Est (adj)	LCI (adj)	UCI (adj)	Est (adj; std)	LCI (adj; std)	UCI (adj; std)	*p* (adj)
1a	Dep (cont)	Belief (cat)	Not sure	1.008	0.989	1.026	1.031	0.962	1.105	0.4125	1.000	0.970	1.030	0.998	0.888	1.121	0.9793
1a	Dep (cont)	Belief (cat)	Yes	1.037	1.021	1.054	1.153	1.085	1.226	<0.0001	1.027	0.990	1.067	1.108	0.960	1.278	0.1543
1b	Anx (cont)	Belief (cat)	Not sure	1.006	0.980	1.033	1.017	0.951	1.087	0.6442	0.987	0.946	1.029	0.966	0.867	1.077	0.5358
1b	Anx (cont)	Belief (cat)	Yes	1.056	1.031	1.081	1.152	1.084	1.225	<0.0001	1.025	0.969	1.084	1.065	0.922	1.230	0.3828
2a	Dep (cont)	Identity (cat)	Christian	1.022	1.004	1.039	1.085	1.016	1.159	0.0153	1.040	1.008	1.072	1.160	1.031	1.305	0.0135
2a	Dep (cont)	Identity (cat)	Other	1.128	1.092	1.164	1.585	1.402	1.791	<0.0001	1.093	1.025	1.166	1.405	1.099	1.798	0.0069
2b	Anx (cont)	Identity (cat)	Christian	1.007	0.983	1.030	1.017	0.958	1.081	0.5775	1.034	0.986	1.085	1.090	0.963	1.234	0.1680
2b	Anx (cont)	Identity (cat)	Other	1.201	1.146	1.260	1.608	1.421	1.819	<0.0001	1.175	1.073	1.286	1.517	1.201	1.915	0.0006
3a	Dep (cont)	Attend (cat)	1/yr	0.989	0.972	1.007	0.962	0.899	1.030	0.2389	0.981	0.953	1.010	0.930	0.833	1.038	0.1879
3a	Dep (cont)	Attend (cat)	1/mth	1.014	0.987	1.042	1.060	0.955	1.176	0.3157	1.012	0.967	1.059	1.050	0.882	1.249	0.6168
3a	Dep (cont)	Attend (cat)	1/wk	1.007	0.980	1.035	1.030	0.927	1.144	0.6235	1.033	0.977	1.093	1.135	0.916	1.407	0.2492
3b	Anx (cont)	Attend (cat)	1/yr	0.987	0.961	1.013	0.967	0.904	1.035	0.3152	0.945	0.902	0.990	0.866	0.769	0.975	0.0176
3b	Anx (cont)	Attend (cat)	1/mth	1.031	0.989	1.074	1.084	0.974	1.206	0.1536	1.003	0.929	1.081	1.007	0.829	1.223	0.9477
3b	Anx (cont)	Attend (cat)	1/wk	1.020	0.980	1.062	1.055	0.951	1.171	0.3279	0.986	0.898	1.082	0.959	0.752	1.222	0.7651
4a	Dep (bin)	Belief (cat)	Not sure	0.961	0.696	1.327	-	-	-	0.8078	0.947	0.601	1.491	-	-	-	0.8133

Model num.	Mental health expo-sure	RSBB out-come	RSBB out-come level	Est (no adj)	LCI (no adj)	UCI (no adj)	Est (no adj; std)	LCI (no adj; std)	UCI (no adj; std)	*p* (no adj)	Est (adj)	LCI (adj)	UCI (adj)	Est (adj; std)	LCI (adj; std)	UCI (adj; std)	*p* (adj)
4a	Dep (bin)	Belief (cat)	Yes	1.395	1.031	1.887	-	-	-	0.0308	1.215	0.691	2.136	-	-	-	0.4977
4b	Anx (bin)	Belief (cat)	Not sure	0.904	0.638	1.281	-	-	-	0.5698	0.903	0.560	1.456	-	-	-	0.6728
4b	Anx (bin)	Belief (cat)	Yes	1.281	0.939	1.747	-	-	-	0.1181	1.157	0.641	2.088	-	-	-	0.6258
5a	Dep (bin)	Identity (cat)	Christian	1.176	0.821	1.683	-	-	-	0.3750	1.406	0.848	2.329	-	-	-	0.1850
5a	Dep (bin)	Identity (cat)	Other	4.985	3.123	7.956	-	-	-	<0.0001	2.950	1.384	6.287	-	-	-	0.0053
5b	Anx (bin)	Identity (cat)	Christian	0.976	0.717	1.328	-	-	-	0.8751	1.179	0.740	1.879	-	-	-	0.4860
5b	Anx (bin)	Identity (cat)	Other	4.600	2.928	7.227	-	-	-	<0.0001	2.914	1.450	5.857	-	-	-	0.0028
6a	Dep (bin)	Attend (cat)	1/yr	0.782	0.535	1.143	-	-	-	0.2026	0.800	0.502	1.275	-	-	-	0.3461
6a	Dep (bin)	Attend (cat)	1/mth	1.109	0.647	1.902	-	-	-	0.7059	1.130	0.543	2.351	-	-	-	0.7437
6a	Dep (bin)	Attend (cat)	1/wk	0.904	0.501	1.632	-	-	-	0.7374	1.147	0.421	3.127	-	-	-	0.7873
6b	Anx (bin)	Attend (cat)	1/yr	0.760	0.525	1.101	-	-	-	0.1454	0.727	0.453	1.166	-	-	-	0.1849
6b	Anx (bin)	Attend (cat)	1/mth	0.962	0.520	1.779	-	-	-	0.9019	0.904	0.392	2.084	-	-	-	0.8108
6b	Anx (bin)	Attend (cat)	1/wk	0.974	0.573	1.656	-	-	-	0.9220	1.123	0.456	2.766	-	-	-	0.8005
7a	Dep (cont)	Belief (bin)	Yes	1.033	1.020	1.047	1.135	1.079	1.194	<0.0001	1.028	0.998	1.058	1.109	0.993	1.239	0.0634
7b	Anx (cont)	Belief (bin)	Yes	1.053	1.033	1.073	1.142	1.087	1.201	<0.0001	1.035	0.989	1.084	1.094	0.971	1.231	0.1371
8a	Dep (cont)	Identity (bin)	Religious	1.028	1.011	1.045	1.112	1.044	1.185	0.0011	1.047	1.017	1.078	1.191	1.065	1.331	0.0023
8b	Anx (cont)	Identity (bin)	Religious	1.019	0.997	1.042	1.050	0.991	1.113	0.0972	1.052	1.005	1.101	1.140	1.015	1.281	0.0282

**Model num.**	**Mental health expo-sure**	**RSBB out-come**	**RSBB out-come level**	**Est (no adj)**	**LCI (no adj)**	**UCI (no adj)**	**Est (no adj; std)**	**LCI (no adj; std)**	**UCI (no adj; std)**	***p* (no adj)**	**Est (adj)**	**LCI (adj)**	**UCI (adj)**	**Est (adj; std)**	**LCI (adj; std)**	**UCI (adj; std)**	***p* (adj)**
9a	Dep (cont)	Attend (bin)	Regular	1.013	0.993	1.033	1.053	0.976	1.135	0.2132	1.031	0.993	1.069	1.122	0.975	1.290	0.1079
9b	Anx (cont)	Attend (bin)	Regular	1.028	0.999	1.058	1.076	1.000	1.159	0.0585	1.022	0.963	1.086	1.059	0.907	1.236	0.4702
10a	Dep (bin)	Belief (bin)	Yes	1.422	1.101	1.837	-	-	-	0.0071	1.265	0.819	1.955	-	-	-	0.2879
10b	Anx (bin)	Belief (bin)	Yes	1.344	1.047	1.726	-	-	-	0.0205	1.253	0.803	1.955	-	-	-	0.3193
11a	Dep (bin)	Identity (bin)	Religious	1.348	0.958	1.896	-	-	-	0.0863	1.660	1.037	2.658	-	-	-	0.0348
11b	Anx (bin)	Identity (bin)	Religious	1.137	0.850	1.521	-	-	-	0.3857	1.448	0.944	2.222	-	-	-	0.0900
12a	Dep (bin)	Attend (bin)	Regular	1.056	0.708	1.574	-	-	-	0.7896	1.235	0.659	2.317	-	-	-	0.5086
12b	Anx (bin)	Attend (bin)	Regular	1.034	0.695	1.538	-	-	-	0.8676	1.138	0.602	2.153	-	-	-	0.6893

Table S32: Predicted probabilities of the partners multinomial and logistic regression analyses with depression and anxiety exposures and religious/spiritual belief and behaviour (RSBB) outcomes using multiple imputation (*n* = 9,887). These results indicate the change in the predicted probability of the RSBB outcome, based on either a standardised one-unit increase in depression or anxiety score (if exposure continuous) or the difference if a probable depression or anxiety diagnosis (if exposure binary). For the associated multinomial or logistic regression models, see table S31. LCI = Lower 95% confidence interval; UCI = Upper 95% confidence interval; no adj = unadjusted model; adj = Adjusted model.

**Model num.**	**Mental health exposure**	**RSBB outcome**	**RSBB outcome level**	**Est (no adj)**	**LCI (no adj)**	**UCI (no adj)**	**Est (no adj; std)**	**LCI (no adj; std)**	**UCI (no adj; std)**	**Est (adj)**	**LCI (adj)**	**UCI (adj)**	**Est (adj; std)**	**LCI (adj; std)**	**UCI (adj; std)**
1a	Dep (cont)	Belief (cat)	No	-0.48	-0.81	-0.14	-1.87	-3.15	-0.60	-0.07	-0.42	0.28	-0.26	-1.60	1.08
1a	Dep (cont)	Belief (cat)	Not sure	-0.24	-0.58	0.10	-0.90	-2.20	0.40	-0.23	-0.64	0.18	-0.89	-2.46	0.68
1a	Dep (cont)	Belief (cat)	Yes	0.72	0.43	1.00	2.77	1.67	3.87	0.30	-0.02	0.62	1.15	-0.08	2.38
1b	Anx (cont)	Belief (cat)	No	-0.65	-1.14	-0.17	-1.70	-2.95	-0.46	0.05	-0.43	0.54	0.14	-1.11	1.39
1b	Anx (cont)	Belief (cat)	Not sure	-0.47	-0.95	0.01	-1.21	-2.45	0.03	-0.44	-1.04	0.17	-1.13	-2.70	0.44
1b	Anx (cont)	Belief (cat)	Yes	1.12	0.70	1.54	2.91	1.84	3.99	0.38	-0.13	0.90	0.99	-0.33	2.31
2a	Dep (cont)	Identity (cat)	None	-0.48	-0.79	-0.17	-1.86	-3.05	-0.67	-0.45	-0.77	-0.14	-1.73	-2.92	-0.53
2a	Dep (cont)	Identity (cat)	Christian	0.11	-0.22	0.45	0.43	-0.86	1.73	0.30	-0.04	0.64	1.13	-0.16	2.42
2a	Dep (cont)	Identity (cat)	Other	0.37	0.25	0.49	1.43	0.97	1.88	0.16	0.00	0.31	0.60	0.01	1.19
2b	Anx (cont)	Identity (cat)	None	-0.28	-0.71	0.14	-0.74	-1.83	0.36	-0.47	-0.95	0.02	-1.19	-2.45	0.06
2b	Anx (cont)	Identity (cat)	Christian	-0.35	-0.81	0.11	-0.91	-2.10	0.28	0.12	-0.41	0.66	0.31	-1.06	1.68
2b	Anx (cont)	Identity (cat)	Other	0.63	0.45	0.81	1.65	1.18	2.12	0.34	0.12	0.56	0.88	0.31	1.46
3a	Dep (cont)	Attend (cat)	Never	0.06	-0.25	0.37	0.16	-1.03	1.35	0.14	-0.21	0.50	0.52	-0.83	1.86
3a	Dep (cont)	Attend (cat)	1/yr	-0.20	-0.48	0.09	-0.72	-1.80	0.36	-0.30	-0.65	0.05	-1.13	-2.46	0.19
3a	Dep (cont)	Attend (cat)	1/mth	0.08	-0.06	0.23	0.34	-0.21	0.89	0.06	-0.13	0.24	0.23	-0.47	0.93
3a	Dep (cont)	Attend (cat)	1/wk	0.05	-0.12	0.23	0.22	-0.46	0.90	0.10	-0.05	0.26	0.39	-0.20	0.98
3b	Anx (cont)	Attend (cat)	Never	-0.03	-0.47	0.42	-0.10	-1.26	1.05	0.59	0.03	1.15	1.51	0.07	2.95
3b	Anx (cont)	Attend (cat)	1/yr	-0.27	-0.69	0.14	-0.70	-1.78	0.38	-0.72	-1.28	-0.16	-1.83	-3.27	-0.38
3b	Anx (cont)	Attend (cat)	1/mth	0.17	-0.05	0.39	0.44	-0.13	1.00	0.12	-0.19	0.43	0.31	-0.48	1.11
3b	Anx (cont)	Attend (cat)	1/wk	0.14	-0.13	0.40	0.36	-0.32	1.05	0.01	-0.26	0.28	0.01	-0.69	0.70
4a	Dep (bin)	Belief (cat)	No	-3.38	-9.07	2.31	-	-	-	-0.08	-5.23	5.08	-	-	-

Model num.	Mental health exposure	RSBB outcome	RSBB outcome level	Est (no adj)	LCI (no adj)	UCI (no adj)	Est (no adj; std)	LCI (no adj; std)	UCI (no adj; std)	Est (adj)	LCI (adj)	UCI (adj)	Est (adj; std)	LCI (adj; std)	UCI (adj; std)
4a	Dep (bin)	Belief (cat)	Not sure	-4.74	-10.46	0.97	-	-	-	-2.55	-8.53	3.42	-	-	-
4a	Dep (bin)	Belief (cat)	Yes	8.12	1.99	14.25	-	-	-	2.63	-2.35	7.61	-	-	-
4b	Anx (bin)	Belief (cat)	No	-1.77	-8.05	4.50	-	-	-	0.50	-5.01	6.00	-	-	-
4b	Anx (bin)	Belief (cat)	Not sure	-5.00	-10.87	0.87	-	-	-	-2.97	-9.01	3.07	-	-	-
4b	Anx (bin)	Belief (cat)	Yes	6.78	0.86	12.69	-	-	-	2.47	-2.52	7.46	-	-	-
5a	Dep (bin)	Identity (cat)	None	-5.11	-10.52	0.31	-	-	-	-4.26	-8.99	0.46	-	-	-
5a	Dep (bin)	Identity (cat)	Christian	-4.87	-11.83	2.09	-	-	-	1.51	-3.91	6.92	-	-	-
5a	Dep (bin)	Identity (cat)	Other	9.97	5.43	14.52	-	-	-	2.76	-0.16	5.67	-	-	-
5b	Anx (bin)	Identity (cat)	None	-2.29	-7.37	2.79	-	-	-	-2.72	-7.18	1.74	-	-	-
5b	Anx (bin)	Identity (cat)	Christian	-8.30	-15.08	-1.51	-	-	-	-0.45	-5.72	4.82	-	-	-
5b	Anx (bin)	Identity (cat)	Other	10.59	5.67	15.51	-	-	-	3.18	0.24	6.11	-	-	-
6a	Dep (bin)	Attend (cat)	Never	2.96	-3.26	9.18	-	-	-	1.64	-3.90	7.18	-	-	-
6a	Dep (bin)	Attend (cat)	1/yr	-3.63	-8.83	1.56	-	-	-	-3.08	-8.22	2.05	-	-	-
6a	Dep (bin)	Attend (cat)	1/mth	0.95	-2.26	4.16	-	-	-	0.88	-2.37	4.13	-	-	-
6a	Dep (bin)	Attend (cat)	1/wk	-0.27	-3.90	3.35	-	-	-	0.56	-2.39	3.51	-	-	-
6b	Anx (bin)	Attend (cat)	Never	3.54	-2.53	9.60	-	-	-	3.00	-2.48	8.47	-	-	-
6b	Anx (bin)	Attend (cat)	1/yr	-3.98	-8.95	1.00	-	-	-	-3.82	-8.83	1.18	-	-	-
6b	Anx (bin)	Attend (cat)	1/mth	0.18	-3.03	3.38	-	-	-	0.07	-3.30	3.44	-	-	-
6b	Anx (bin)	Attend (cat)	1/wk	0.27	-3.30	3.83	-	-	-	0.76	-1.92	3.44	-	-	-
7a	Dep (cont)	Belief (bin)	Yes	0.71	0.43	1.00	2.77	1.67	3.86	0.30	-0.02	0.62	1.15	-0.08	2.38
7b	Anx (cont)	Belief (bin)	Yes	1.12	0.70	1.54	2.91	1.83	3.98	0.38	-0.12	0.89	0.99	-0.32	2.30
8a	Dep (cont)	Identity (bin)	Religious	0.52	0.21	0.82	1.98	0.81	3.15	0.49	0.18	0.79	1.86	0.69	3.03
8b	Anx (cont)	Identity (bin)	Religious	0.35	-0.06	0.77	0.91	-0.16	1.99	0.54	0.06	1.02	1.40	0.16	2.63
9a	Dep (cont)	Attend (bin)	Regular	0.14	-0.08	0.36	0.56	-0.27	1.40	0.17	-0.04	0.37	0.63	-0.14	1.40
9b	Anx (cont)	Attend (bin)	Regular	0.31	-0.01	0.62	0.81	-0.01	1.62	0.12	-0.21	0.45	0.31	-0.54	1.17
10a	Dep (bin)	Belief (bin)	Yes	8.12	1.99	14.25	-	-	-	2.65	-2.28	7.57	-	-	-

Model num.	Mental health exposure	RSBB outcome	RSBB outcome level	Est (no adj)	LCI (no adj)	UCI (no adj)	Est (no adj; std)	LCI (no adj; std)	UCI (no adj; std)	Est (adj)	LCI (adj)	UCI (adj)	Est (adj; std)	LCI (adj; std)	UCI (adj; std)
10b	Anx (bin)	Belief (bin)	Yes	6.78	0.86	12.69	-	-	-	2.53	-2.49	7.55	-	-	-
11a	Dep (bin)	Identity (bin)	Religious	5.11	-0.31	10.52	-	-	-	5.12	0.61	9.64	-	-	-
11b	Anx (bin)	Identity (bin)	Religious	2.29	-2.79	7.37	-	-	-	3.80	-0.44	8.04	-	-	-
12a	Dep (bin)	Attend (bin)	Regular	0.67	-3.88	5.23	-	-	-	1.28	-2.54	5.11	-	-	-
12b	Anx (bin)	Attend (bin)	Regular	0.44	-4.05	4.93	-	-	-	0.80	-2.93	4.54	-	-	-

Table S33: Results of the interaction analyses assessing whether the adjusted mother and partner results differ, with standardised depression and anxiety scores, or binary probable depression and anxiety diagnoses, as exposures and categorical religious/spiritual belief and behaviour (RSBB) as outcomes using multiple imputation. RRR = Relative risk ratio; SE = Standard error; LCI = Lower 95% confidence interval; UCI = Upper 95% confidence interval; *p* = *p*-value.

**Model num.**	**Exposure**	**Outcome**	**Out-come level**	**Mother RRR**	**Mother log-RRR**	**Mother log-RRR SE**	**Partner RRR**	**Partner log-RRR**	**Partner log-RRR SE**	**Log-RRR diff.**	**Diff. log-RRR SE**	**Diff. RRR**	**Diff. RRR LCI**	**Diff. RRR UCI**	**Diff. *p***
1a	Dep (cont)	Belief (cat)	Not sure	0.951	-0.050	0.043	0.998	-0.002	0.059	-0.048	0.073	0.953	0.825	1.100	0.5113
1a	Dep (cont)	Belief (cat)	Yes	0.939	-0.063	0.048	1.108	0.103	0.073	-0.165	0.087	0.847	0.714	1.006	0.0586
1b	Anx (cont)	Belief (cat)	Not sure	0.953	-0.048	0.042	0.966	-0.035	0.055	-0.014	0.069	0.987	0.861	1.130	0.8450
1b	Anx (cont)	Belief (cat)	Yes	0.928	-0.075	0.048	1.065	0.063	0.074	-0.138	0.088	0.871	0.734	1.035	0.1169
2a	Dep (cont)	Identity (cat)	Christian	0.969	-0.031	0.046	1.160	0.148	0.060	-0.180	0.076	0.835	0.720	0.969	0.0177
2a	Dep (cont)	Identity (cat)	Other	0.943	-0.059	0.087	1.405	0.340	0.126	-0.399	0.153	0.671	0.498	0.905	0.0091
2b	Anx (cont)	Identity (cat)	Christian	0.902	-0.103	0.043	1.090	0.086	0.063	-0.189	0.077	0.828	0.712	0.962	0.0135
2b	Anx (cont)	Identity (cat)	Other	0.958	-0.043	0.086	1.517	0.417	0.119	-0.460	0.147	0.632	0.474	0.842	0.0017
3a	Dep (cont)	Attend (cat)	1/yr	0.925	-0.078	0.038	0.930	-0.073	0.056	-0.005	0.068	0.995	0.871	1.136	0.9365
3a	Dep (cont)	Attend (cat)	1/mth	0.922	-0.081	0.054	1.050	0.049	0.089	-0.130	0.104	0.878	0.716	1.076	0.2106
3a	Dep (cont)	Attend (cat)	1/wk	1.001	0.001	0.070	1.135	0.127	0.109	-0.126	0.130	0.882	0.684	1.137	0.3328
3b	Anx (cont)	Attend (cat)	1/yr	0.933	-0.069	0.040	0.866	-0.144	0.061	0.075	0.072	1.077	0.935	1.242	0.3031
3b	Anx (cont)	Attend (cat)	1/mth	0.936	-0.066	0.056	1.007	0.007	0.099	-0.073	0.114	0.929	0.744	1.161	0.5201
3b	Anx (cont)	Attend (cat)	1/wk	1.004	0.004	0.074	0.959	-0.042	0.124	0.046	0.144	1.047	0.789	1.389	0.7503
4a	Dep (bin)	Belief (cat)	Not sure	0.891	-0.115	0.120	0.947	-0.054	0.232	-0.061	0.261	0.941	0.564	1.569	0.8152
4a	Dep (bin)	Belief (cat)	Yes	0.869	-0.140	0.146	1.215	0.195	0.288	-0.335	0.323	0.715	0.380	1.346	0.2989
4b	Anx (bin)	Belief (cat)	Not sure	0.901	-0.104	0.114	0.903	-0.102	0.244	-0.002	0.269	0.998	0.589	1.691	0.9934
4b	Anx (bin)	Belief (cat)	Yes	0.903	-0.102	0.135	1.157	0.146	0.301	-0.248	0.330	0.780	0.409	1.491	0.4528
5a	Dep (bin)	Identity (cat)	Christian	0.856	-0.155	0.128	1.406	0.341	0.258	-0.496	0.288	0.609	0.346	1.070	0.0846
5a	Dep (bin)	Identity (cat)	Other	0.904	-0.101	0.222	2.950	1.082	0.386	-1.183	0.445	0.306	0.128	0.733	0.0079
5b	Anx (bin)	Identity (cat)	Christian	0.798	-0.226	0.122	1.179	0.165	0.238	-0.390	0.267	0.677	0.401	1.143	0.1439
5b	Anx (bin)	Identity (cat)	Other	0.989	-0.011	0.221	2.914	1.070	0.356	-1.081	0.419	0.339	0.149	0.772	0.0099
6a	Dep (bin)	Attend (cat)	1/yr	0.816	-0.203	0.112	0.800	-0.223	0.238	0.020	0.263	1.020	0.610	1.707	0.9399
6a	Dep (bin)	Attend (cat)	1/mth	0.943	-0.059	0.156	1.130	0.122	0.374	-0.181	0.405	0.835	0.377	1.846	0.6552
6a	Dep (bin)	Attend (cat)	1/wk	0.967	-0.034	0.201	1.147	0.137	0.512	-0.171	0.550	0.843	0.287	2.475	0.7561
6b	Anx (bin)	Attend (cat)	1/yr	0.867	-0.143	0.115	0.727	-0.319	0.241	0.176	0.267	1.193	0.707	2.013	0.5096

6b	Anx (bin)	Attend (cat)	1/mth	0.849	-0.164	0.160	0.904	-0.101	0.426	-0.063	0.455	0.939	0.385	2.292	0.8903
6b	Anx (bin)	Attend (cat)	1/wk	1.053	0.052	0.204	1.123	0.116	0.460	-0.064	0.503	0.938	0.350	2.513	0.8982

Table S34: Results of the interaction analyses assessing whether the adjusted mother and partner results differ, with standardised depression and anxiety scores, or binary probable depression and anxiety diagnoses, as exposures and binary religious/spiritual belief and behaviour (RSBB) as outcomes using multiple imputation. OR = Odds ratio; SE = Standard error; LCI = Lower 95% confidence interval; UCI = Upper 95% confidence interval; *p* = *p*-value.

**Model num.**	**Exposure**	**Outcome**	**Out-come level**	**Mother OR**	**Mother log-odds**	**Mother log-odds SE**	**Partner OR**	**Partner log-odds**	**Partner log-odds SE**	**Log-odds diff.**	**Diff. log-odds SE**	**Diff. OR**	**Diff. OR LCI**	**Diff. OR UCI**	**Diff. *p***
7a	Dep (cont)	Belief (bin)	Yes	0.976	-0.024	0.036	1.109	0.103	0.056	-0.128	0.067	0.880	0.772	1.003	0.0560
7b	Anx (cont)	Belief (bin)	Yes	0.964	-0.037	0.035	1.094	0.090	0.061	-0.127	0.070	0.881	0.768	1.010	0.0702
8a	Dep (cont)	Identity (bin)	Religious	0.969	-0.031	0.045	1.191	0.175	0.057	-0.206	0.072	0.814	0.706	0.938	0.0044
8b	Anx (cont)	Identity (bin)	Religious	0.912	-0.092	0.043	1.140	0.131	0.059	-0.223	0.073	0.800	0.693	0.924	0.0024
9a	Dep (cont)	Attend (bin)	Regular	0.990	-0.010	0.045	1.122	0.115	0.071	-0.125	0.084	0.882	0.748	1.041	0.1370
9b	Anx (cont)	Attend (bin)	Regular	0.995	-0.005	0.046	1.059	0.057	0.079	-0.062	0.091	0.940	0.786	1.123	0.4941
10a	Dep (bin)	Belief (bin)	Yes	0.950	-0.051	0.105	1.265	0.235	0.222	-0.286	0.246	0.751	0.464	1.215	0.2437
10b	Anx (bin)	Belief (bin)	Yes	0.980	-0.020	0.105	1.253	0.226	0.227	-0.246	0.250	0.782	0.479	1.277	0.3260
11a	Dep (bin)	Identity (bin)	Religious	0.871	-0.138	0.124	1.660	0.507	0.240	-0.645	0.270	0.525	0.309	0.892	0.0171
11b	Anx (bin)	Identity (bin)	Religious	0.833	-0.183	0.121	1.448	0.370	0.218	-0.553	0.250	0.575	0.353	0.939	0.0269
12a	Dep (bin)	Attend (bin)	Regular	1.051	0.050	0.129	1.235	0.211	0.321	-0.161	0.346	0.851	0.432	1.676	0.6409
12b	Anx (bin)	Attend (bin)	Regular	0.988	-0.012	0.129	1.138	0.129	0.325	-0.141	0.350	0.868	0.437	1.723	0.6861

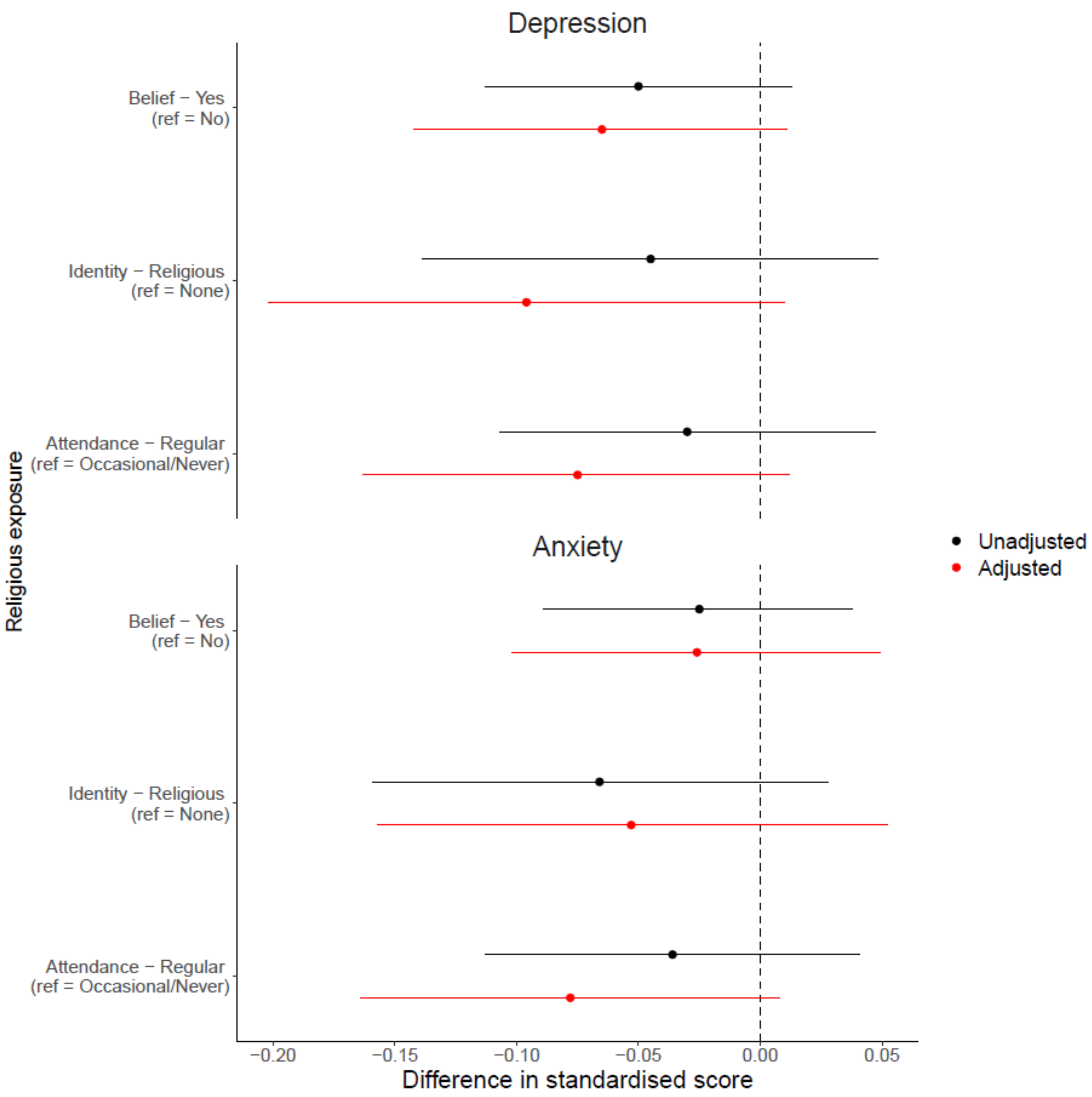

Figure S1: Results of the mothers analyses with binary religious/spiritual belief and behaviour (RSBB) exposures and standardised depression and anxiety scores as outcomes (*n* = 3,856). Results in black are from unadjusted analyses, and those in red from adjusted analyses (adjusting for baseline confounders, RSBB and mental health). The dashed vertical line at '0' indicates a null association. Error bars denote 95% confidence intervals. Full results are in table S8.

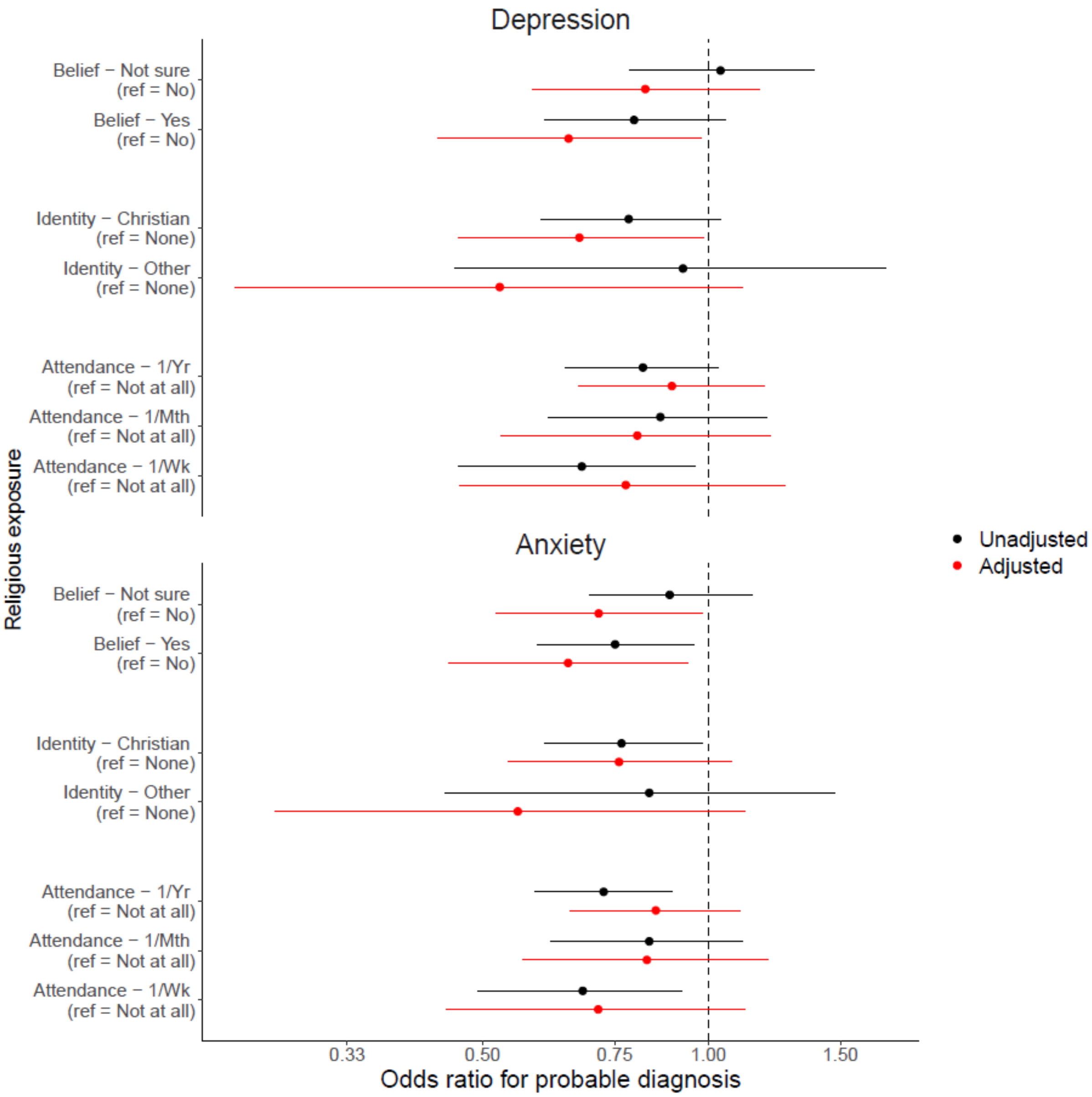

Figure S2: Results of the mothers analyses with categorical religious/spiritual belief and behaviour (RSBB) exposures and binary probable depression and anxiety diagnoses as outcomes, using logistic regression (*n* = 3,856). Results in black are from unadjusted analyses, and those in red from adjusted analyses (adjusting for baseline confounders, RSBB and mental health). The dashed vertical line at '1' indicates a null association. Error bars denote 95% confidence intervals. Taking religious attendance, for instance, relative to the baseline category of 'not at all' attend, in adjusted analyses individuals who attended 'a minimum of once a week' were associated with approximately a 20% decrease in the odds of a probable depression diagnosis (Odds Ratio = 0.776; 95% confidence interval = 0.466 to 1.267). For a plot of the predicted probabilities of the differences in probable depression and anxiety diagnoses from these models, see figure S4. Full results are in table S8.

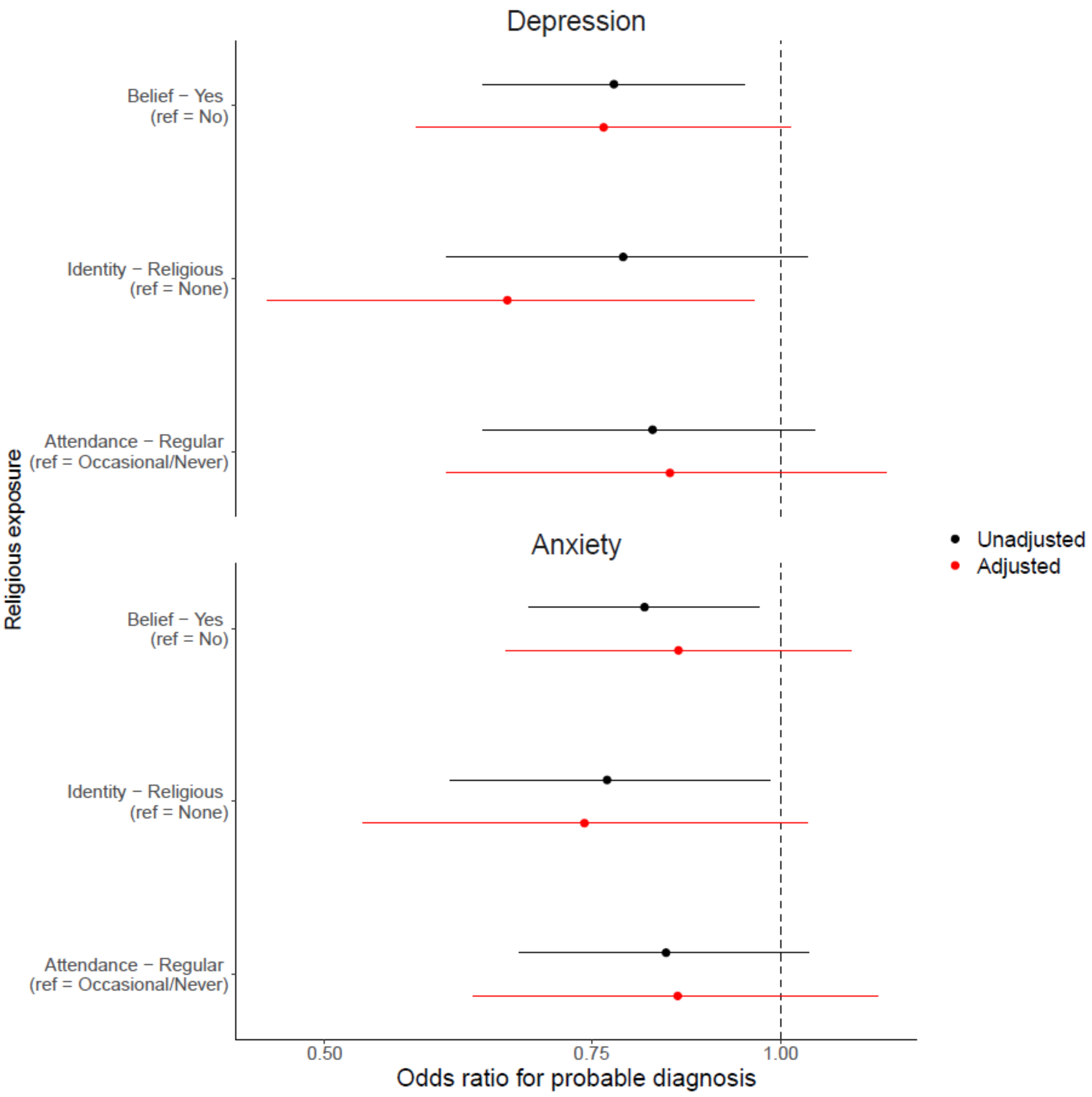

Figure S3: Results of the mothers analyses with binary religious/spiritual belief and behaviour (RSBB) exposures and binary probable depression and anxiety diagnoses as outcomes, using logistic regression (n = 3,856). Results in black are from unadjusted analyses, and those in red from adjusted analyses (adjusting for baseline confounders, RSBB and mental health). The dashed vertical line at ‘1’ indicates a null association. Error bars denote 95% confidence intervals. For a plot of the predicted probabilities of the differences in probable depression and anxiety diagnoses from these models, see figure S5. Full results are in table S8.

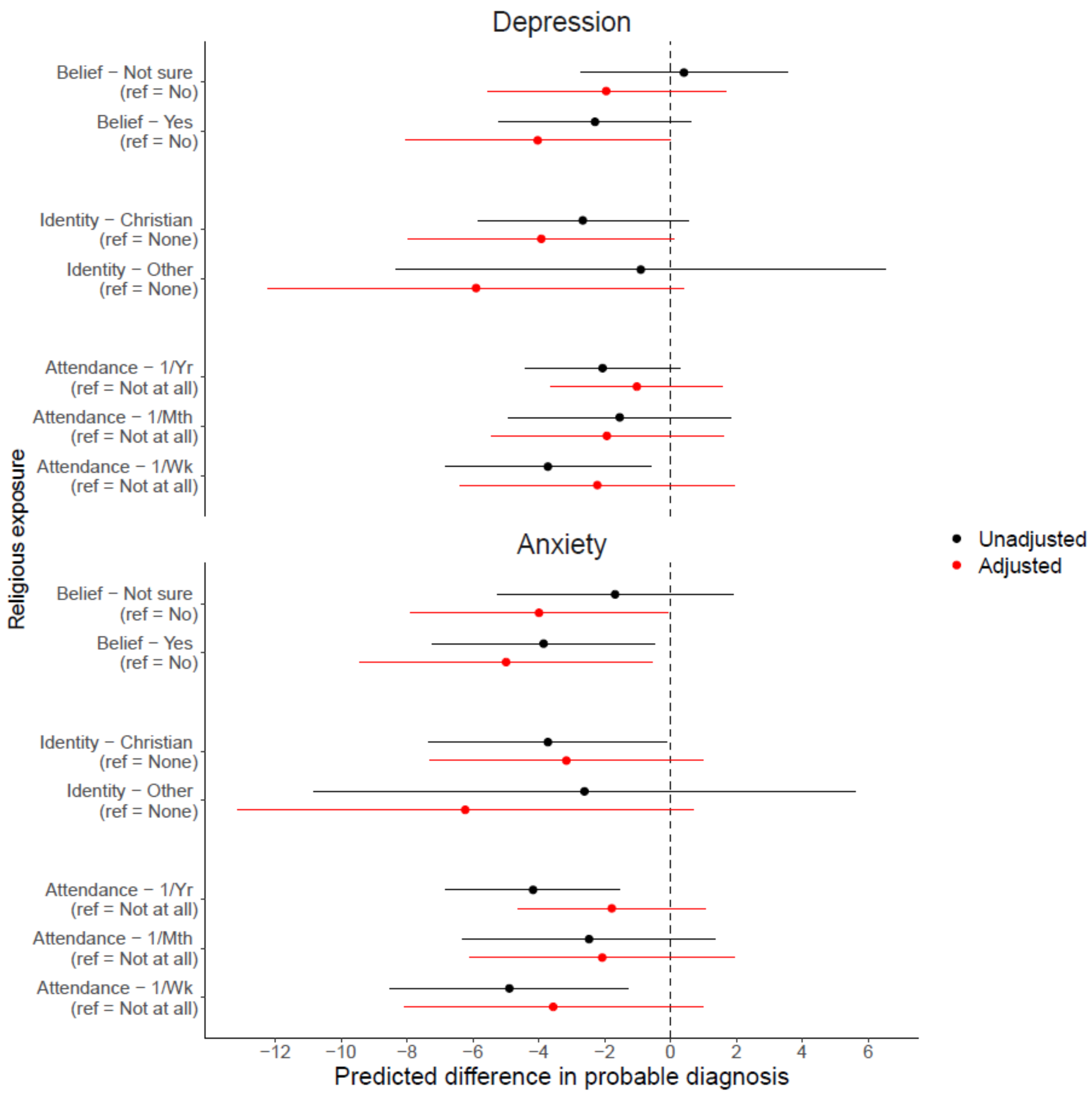

Figure S4: Predicted probabilities of the mothers analyses with categorical religious/spiritual belief and behaviour (RSBB) exposures and binary probable depression and anxiety diagnoses as outcomes, using logistic regression (*n* = 3,856). Results in black are from unadjusted analyses, and those in red from adjusted analyses (adjusting for baseline confounders, RSBB and mental health). The dashed vertical line at ‘0’ indicates no difference in the predicted probability. Error bars denote 95% confidence intervals. These results indicate the difference in the predicted probability of a probable depression or anxiety diagnosis, based on the associated logistic regression model. For instance, taking religious attendance, relative to the baseline category of ‘not at all’ attend, in adjusted analyses individuals who attended ‘a minimum of once a week’ were predicted to be 2.2%-points less likely to be diagnosed with probable depression (95% confidence interval = -6.4 to 1.9). For a plot of the odds ratios associated with these models, see figure S2. Full results are in table S9.

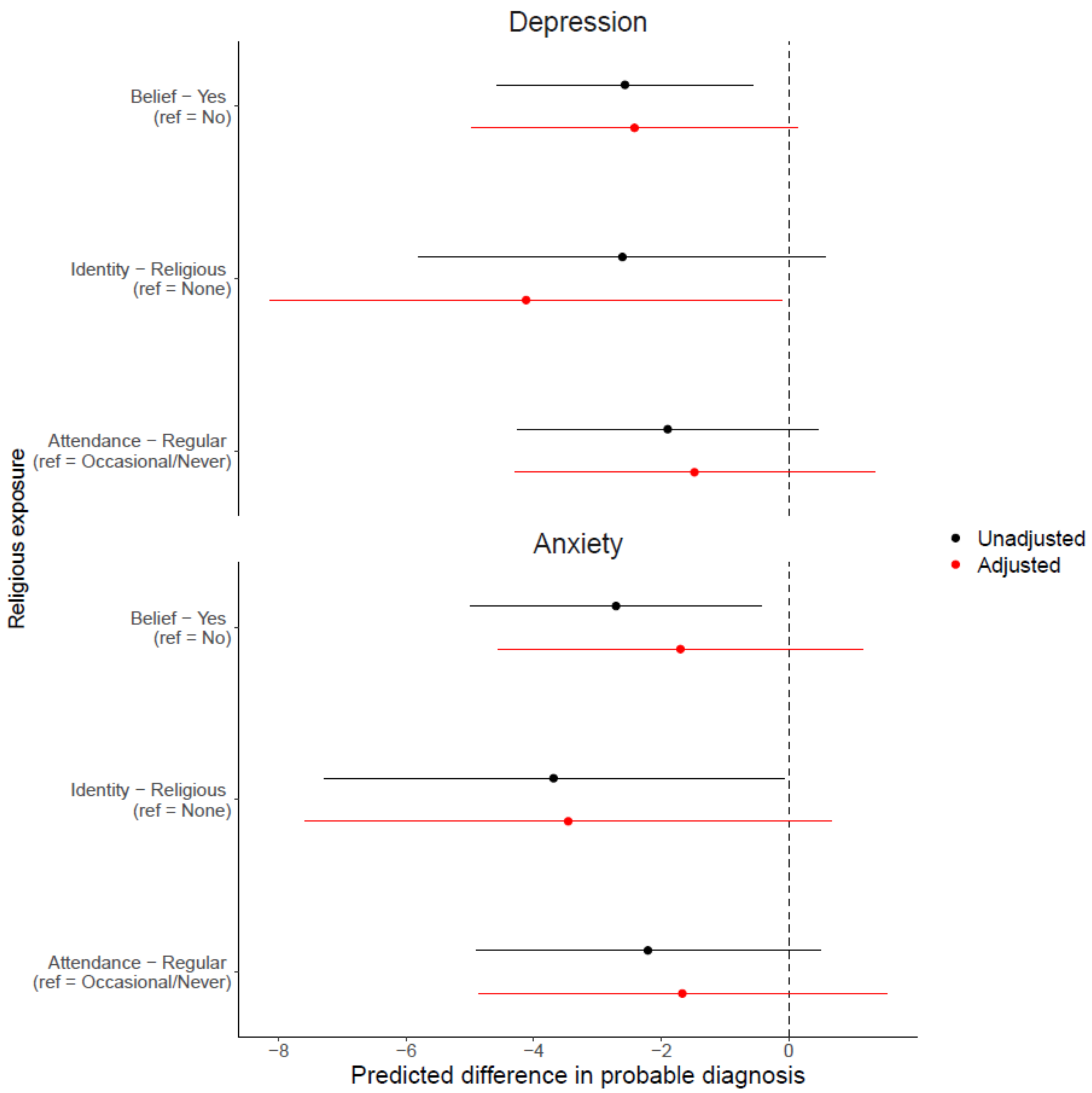

Figure S5: Predicted probabilities of the mothers analyses with binary religious/spiritual belief and behaviour (RSBB) exposures and binary probable depression and anxiety diagnoses as outcomes, using logistic regression (*n* = 3,856). Results in black are from unadjusted analyses, and those in red from adjusted analyses (adjusting for baseline confounders, RSBB and mental health). The dashed vertical line at '0' indicates no difference in the predicted probability. Error bars denote 95% confidence intervals. These results indicate the difference in the predicted probability of a probable depression or anxiety diagnosis, based on the associated logistic regression model. For a plot of the odds ratios associated with these models, see figure S3. Full results are in table S9.

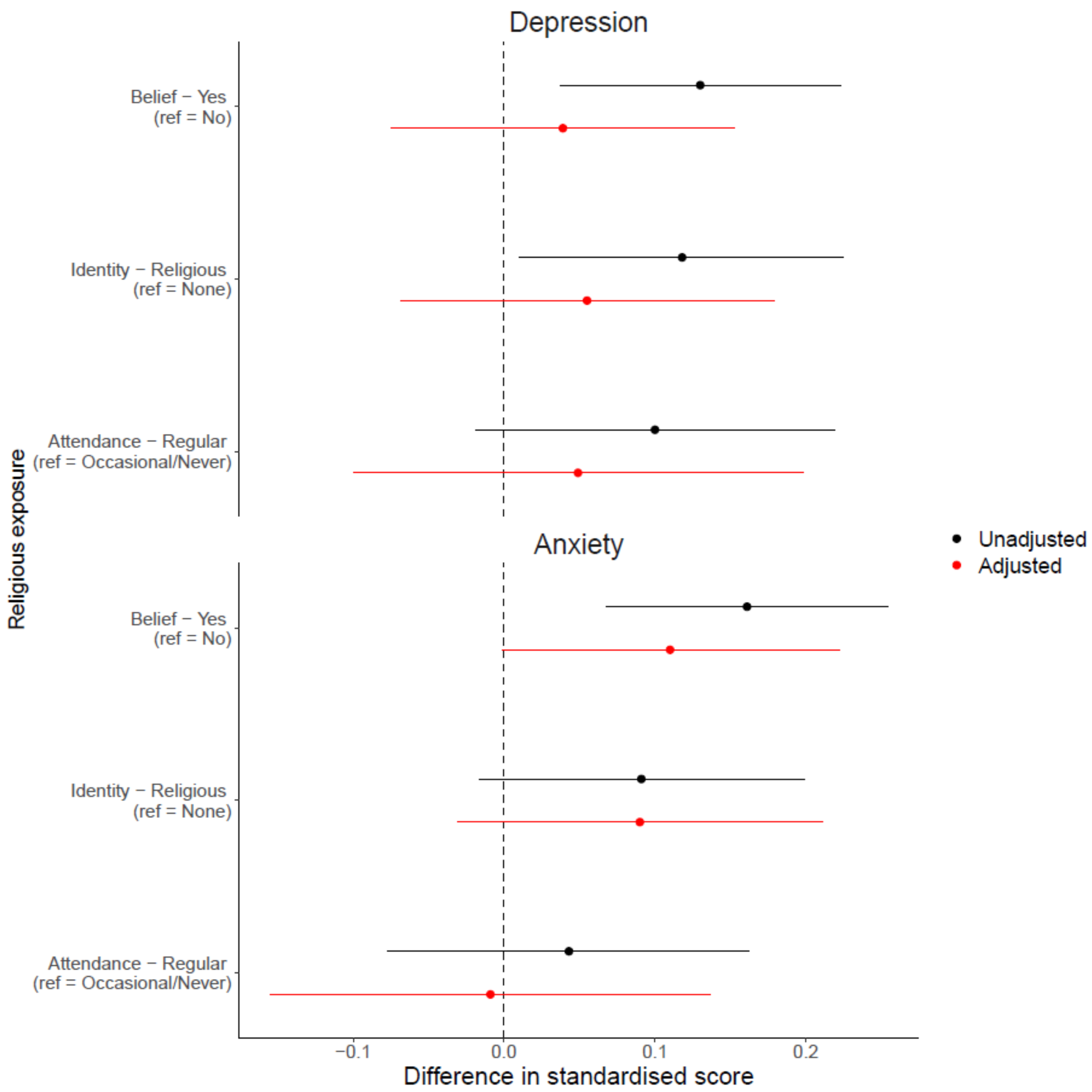

Figure S6: Results of the partners analyses with binary religious/spiritual belief and behaviour (RSBB) exposures and standardised depression and anxiety scores as outcomes (*n* = 1,940). Results in black are from unadjusted analyses, and those in red from adjusted analyses (adjusting for baseline confounders, RSBB and mental health). The dashed vertical line at ‘0’ indicates a null association. Error bars denote 95% confidence intervals. Full results are in table S11.

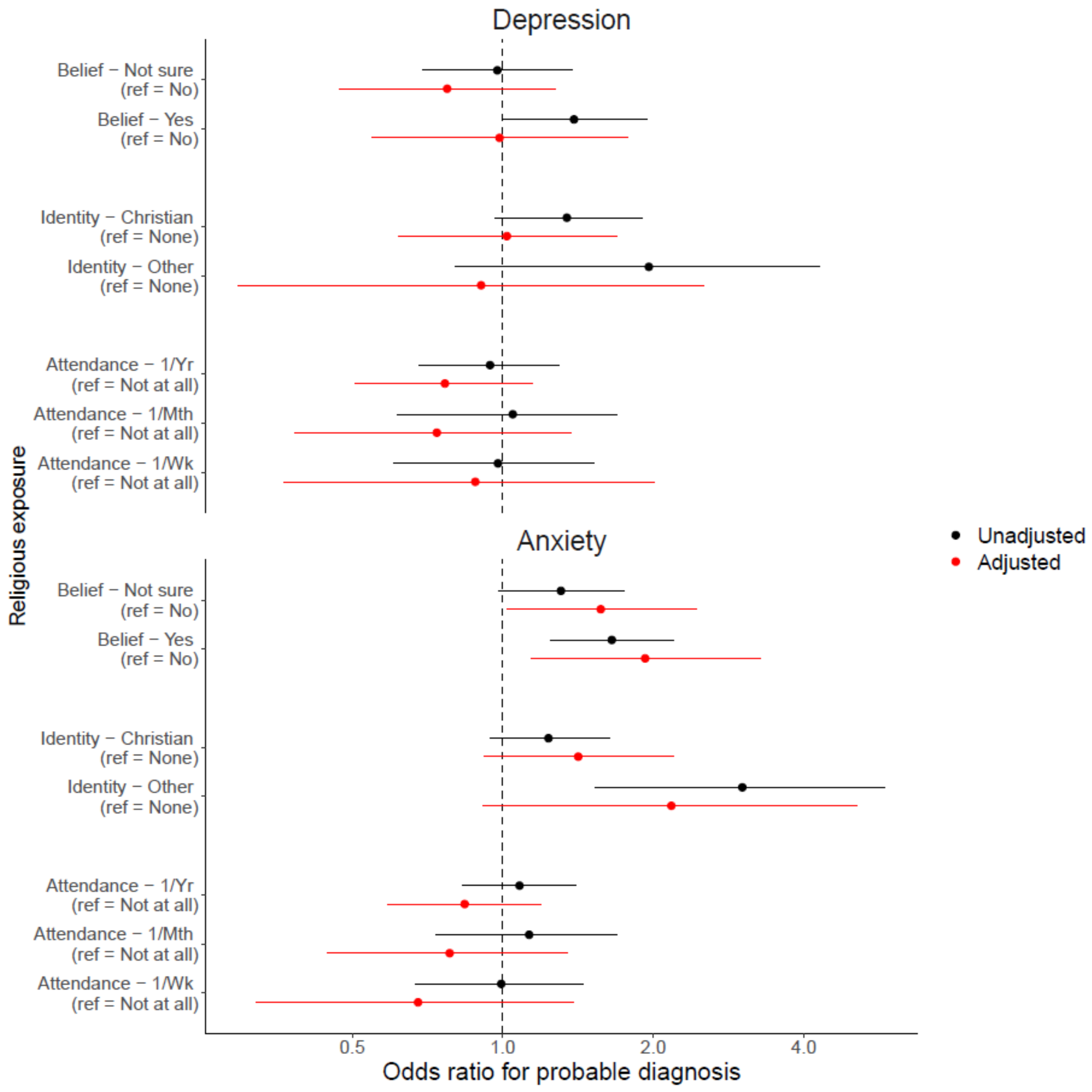

Figure S7: Results of the partners analyses with categorical religious/spiritual belief and behaviour (RSBB) exposures and binary probable depression and anxiety diagnoses as outcomes, using logistic regression (*n* = 1,940). Results in black are from unadjusted analyses, and those in red from adjusted analyses (adjusting for baseline confounders, RSBB and mental health). The dashed vertical line at ‘1’ indicates a null association. Error bars denote 95% confidence intervals. For a plot of the predicted probabilities of the differences in probable depression and anxiety diagnoses from these models, see figure S9. Full results are in table S11.

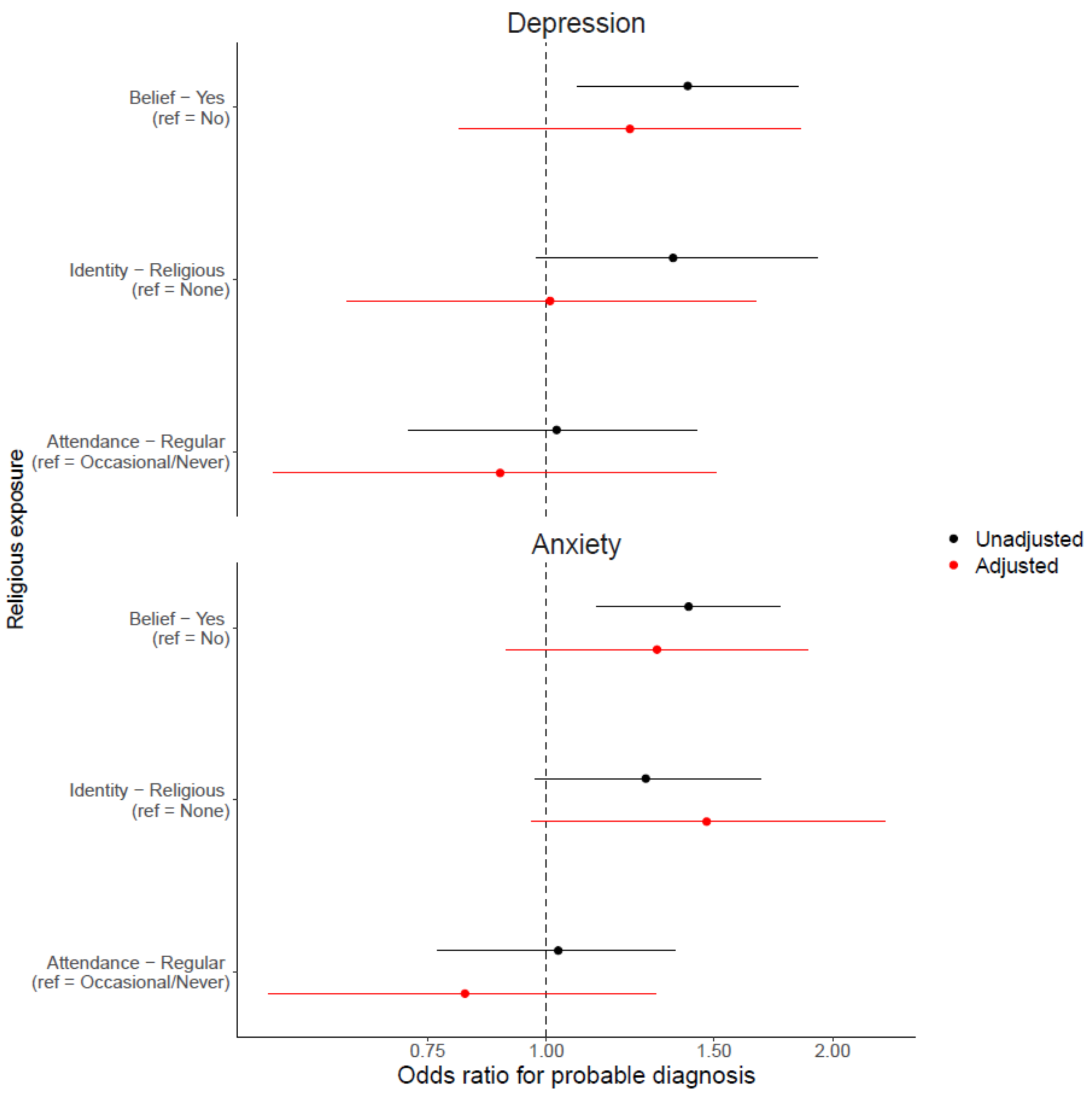

Figure S8: Results of the partners analyses with binary religious/spiritual belief and behaviour (RSBB) exposures and binary probable depression and anxiety diagnoses as outcomes, using logistic regression (*n* = 1,940). Results in black are from unadjusted analyses, and those in red from adjusted analyses (adjusting for baseline confounders, RSBB and mental health). The dashed vertical line at '1' indicates a null association. Error bars denote 95% confidence intervals. For a plot of the predicted probabilities of the differences in probable depression and anxiety diagnoses from these models, see figure S10. Full results are in table S11.

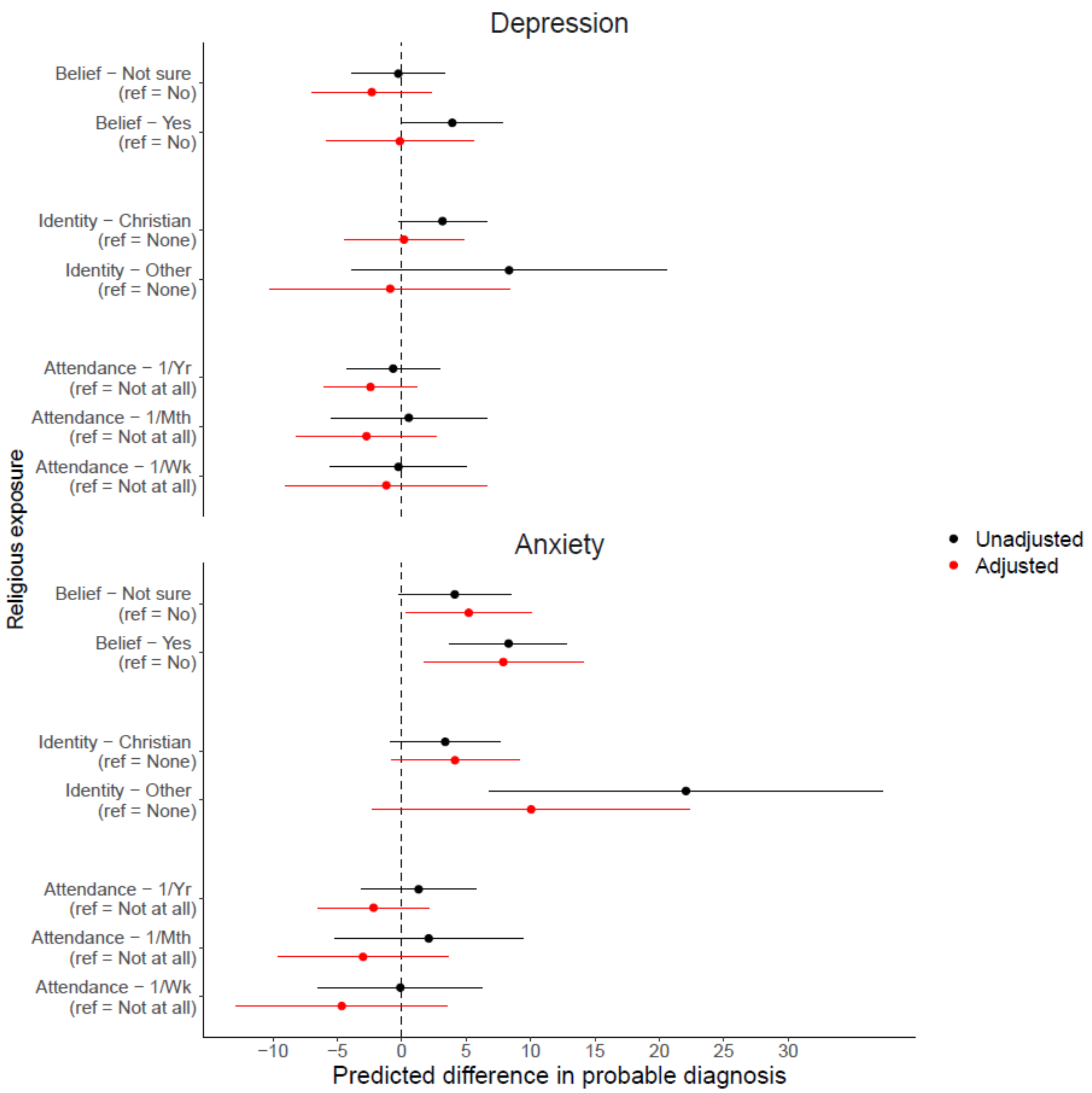

Figure S9: Predicted probabilities of the partners analyses with categorical religious/spiritual belief and behaviour (RSBB) exposures and binary probable depression and anxiety diagnoses as outcomes, using logistic regression (*n* = 1,940). Results in black are from unadjusted analyses, and those in red from adjusted analyses (adjusting for baseline confounders, RSBB and mental health). The dashed vertical line at '0' indicates no difference in the predicted probability. Error bars denote 95% confidence intervals. These results indicate the difference in the predicted probability of a probable depression or anxiety diagnosis, based on the associated logistic regression model. For a plot of the odds ratios associated with these models, see figure S7. Full results are in table S12.

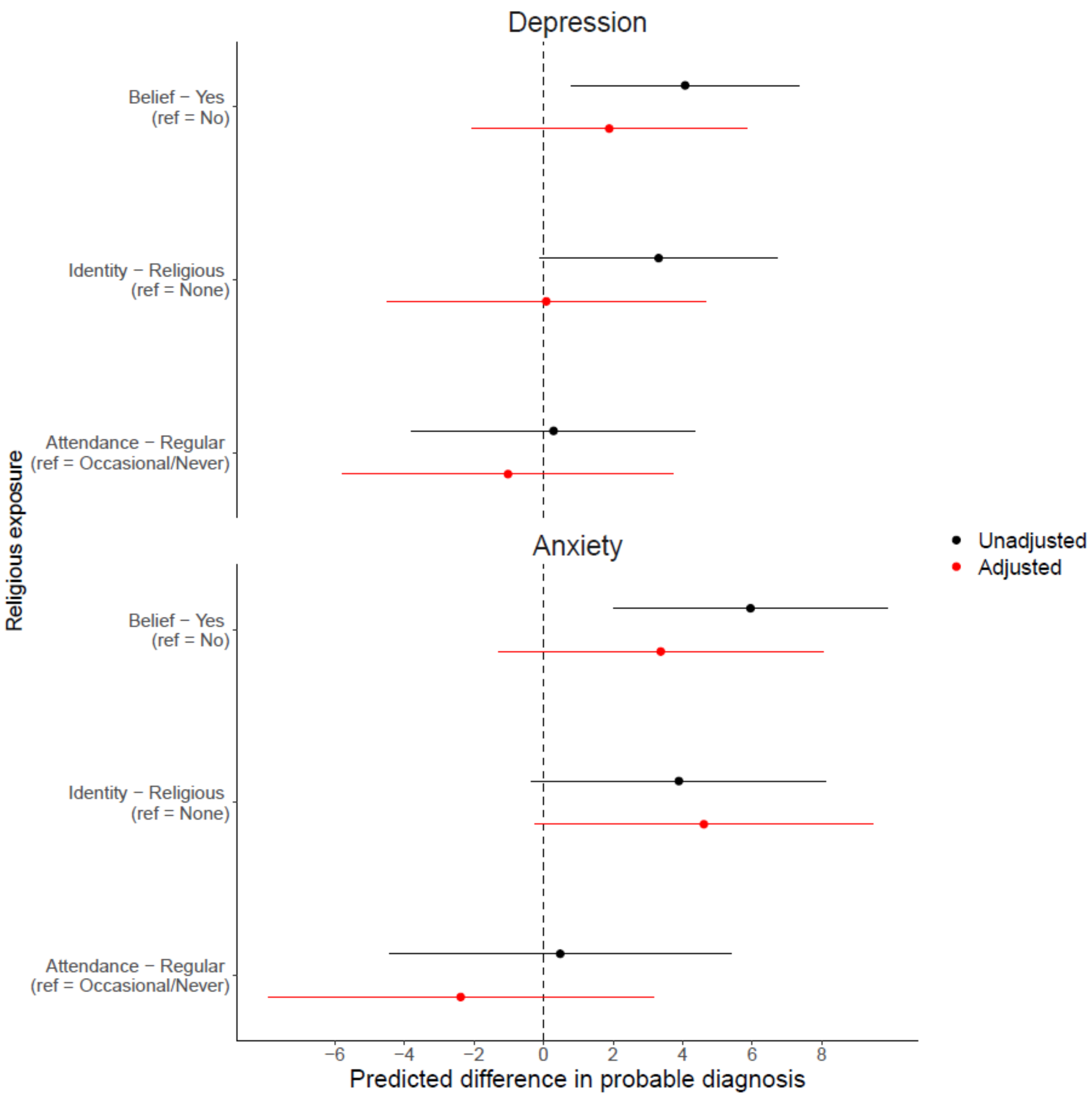

Figure S10: Predicted probabilities of the partners analyses with binary religious/spiritual belief and behaviour (RSBB) exposures and binary probable depression and anxiety diagnoses as outcomes, using logistic regression (*n* = 1,940). Results in black are from unadjusted analyses, and those in red from adjusted analyses (adjusting for baseline confounders, RSBB and mental health). The dashed vertical line at ‘0’ indicates no difference in the predicted probability. Error bars denote 95% confidence intervals. These results indicate the difference in the predicted probability of a probable depression or anxiety diagnosis, based on the associated logistic regression model. For a plot of the odds ratios associated with these models, see figure S8. Full results are in table S12.

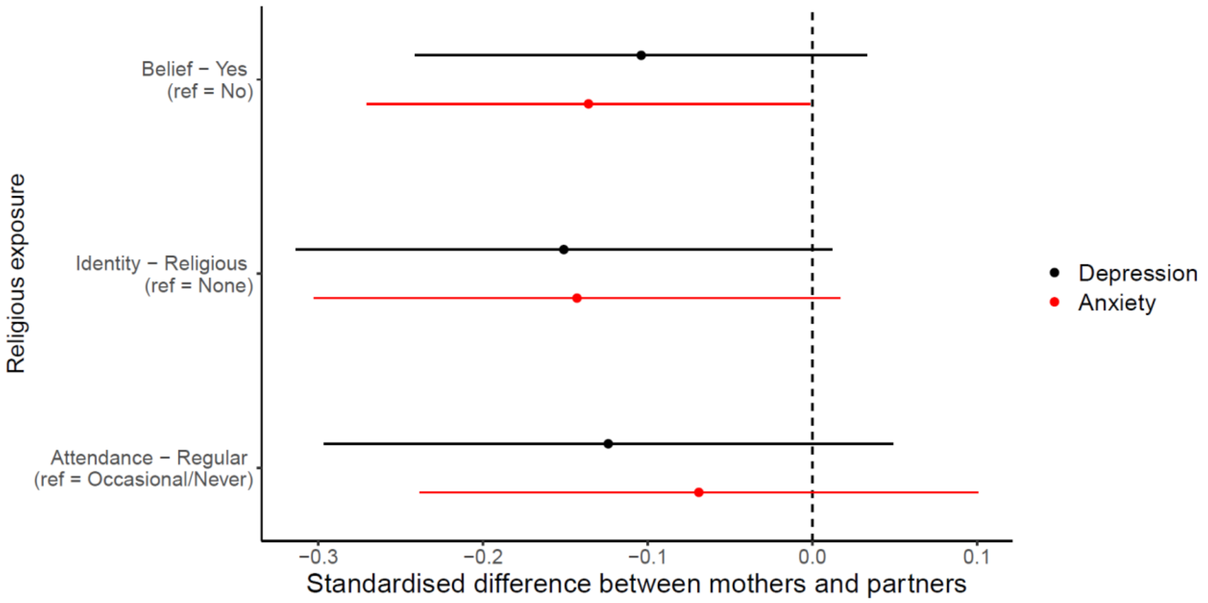

Figure S11: Results of the interaction analyses assessing whether the adjusted mother and partner results differ, with binary religious/spiritual belief and behaviour (RSBB) exposures and standardised depression and anxiety scores as outcomes. Results in black are for the depression outcome, and those in red for anxiety. The dashed vertical line at '0' indicates no difference between mothers and partners, with results below 0 meaning that the estimate was lower in mothers, compared to partners. For instance, for 'Yes' to religious belief and anxiety (relative to answering 'No' or 'Not sure'), the mean difference in mothers was -0.03, while in partners it was 0.11, giving a standardised mean difference here of approximately -0.14. Error bars denote 95% confidence intervals. Full results are in table S13.

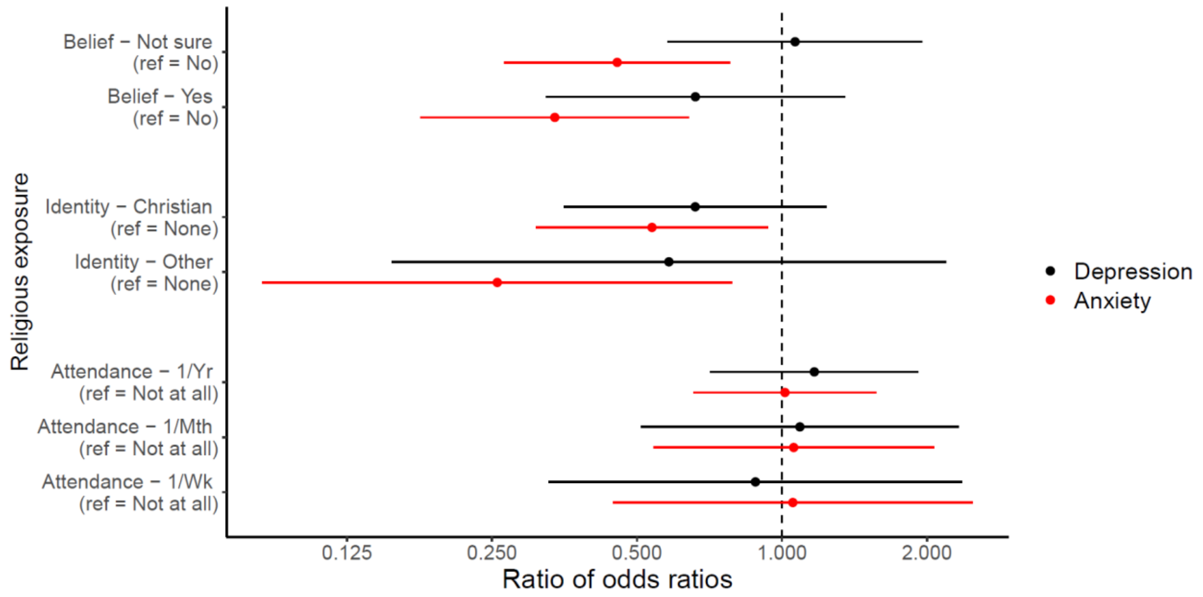

Figure S12: Results of the interaction analyses assessing whether the adjusted mother and partner results differ, with categorical religious/spiritual belief and behaviour (RSBB) exposures and binary probable depression and anxiety diagnoses as outcomes. Results in black are for the depression outcome, and those in red for anxiety. The dashed vertical line at ‘1’ indicates no difference between mothers and partners, with results below 1 meaning that the odds ratio estimate was lower in mothers, compared to partners. For instance, for ‘Yes’ to religious belief and anxiety (relative to answering ‘No’), the odds ratio in mothers was 0.65, while in partners it was 1.93, giving a ratio of these odds ratios here of approximately 0.34. Error bars denote 95% confidence intervals. Full results are in table S14.

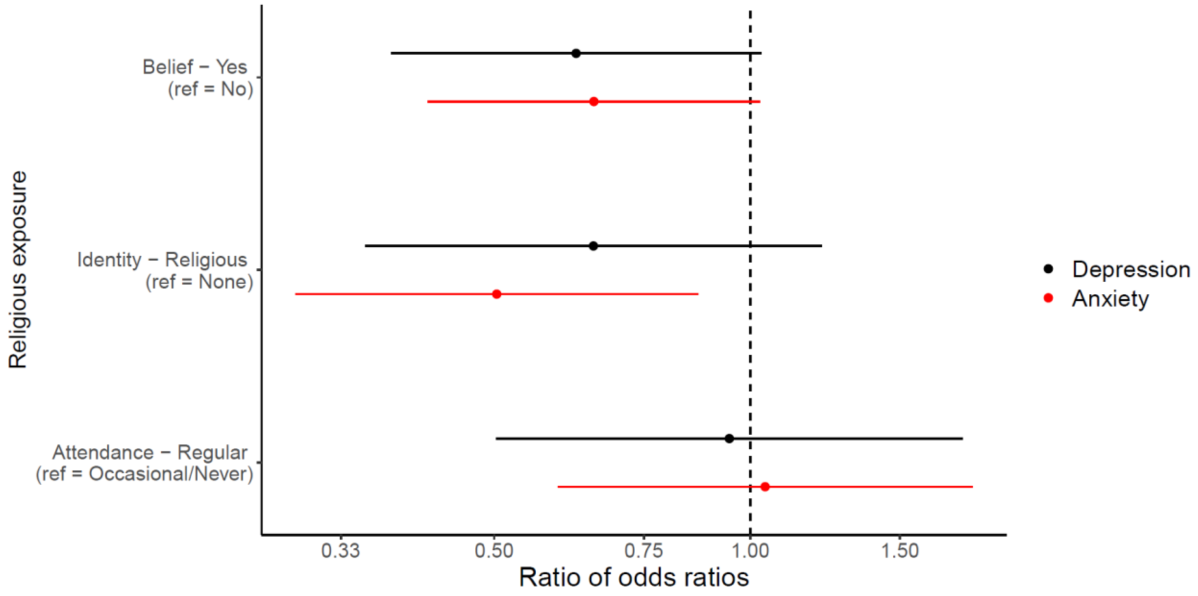

Figure S13: Results of the interaction analyses assessing whether the adjusted mother and partner results differ, with binary religious/spiritual belief and behaviour (RSBB) exposures and binary probable depression and anxiety diagnoses as outcomes. Results in black are for the depression outcome, and those in red for anxiety. The dashed vertical line at '1' indicates no difference between mothers and partners, with results below 1 meaning that the odds ratio estimate was lower in mothers, compared to partners. For instance, for 'Yes' to religious belief and anxiety (relative to answering 'No' or 'Not sure'), the odds ratio in mothers was 0.86, while in partners it was 1.31, giving a ratio of these odds ratios here of approximately 0.65. Error bars denote 95% confidence intervals. Full results are in table S14.

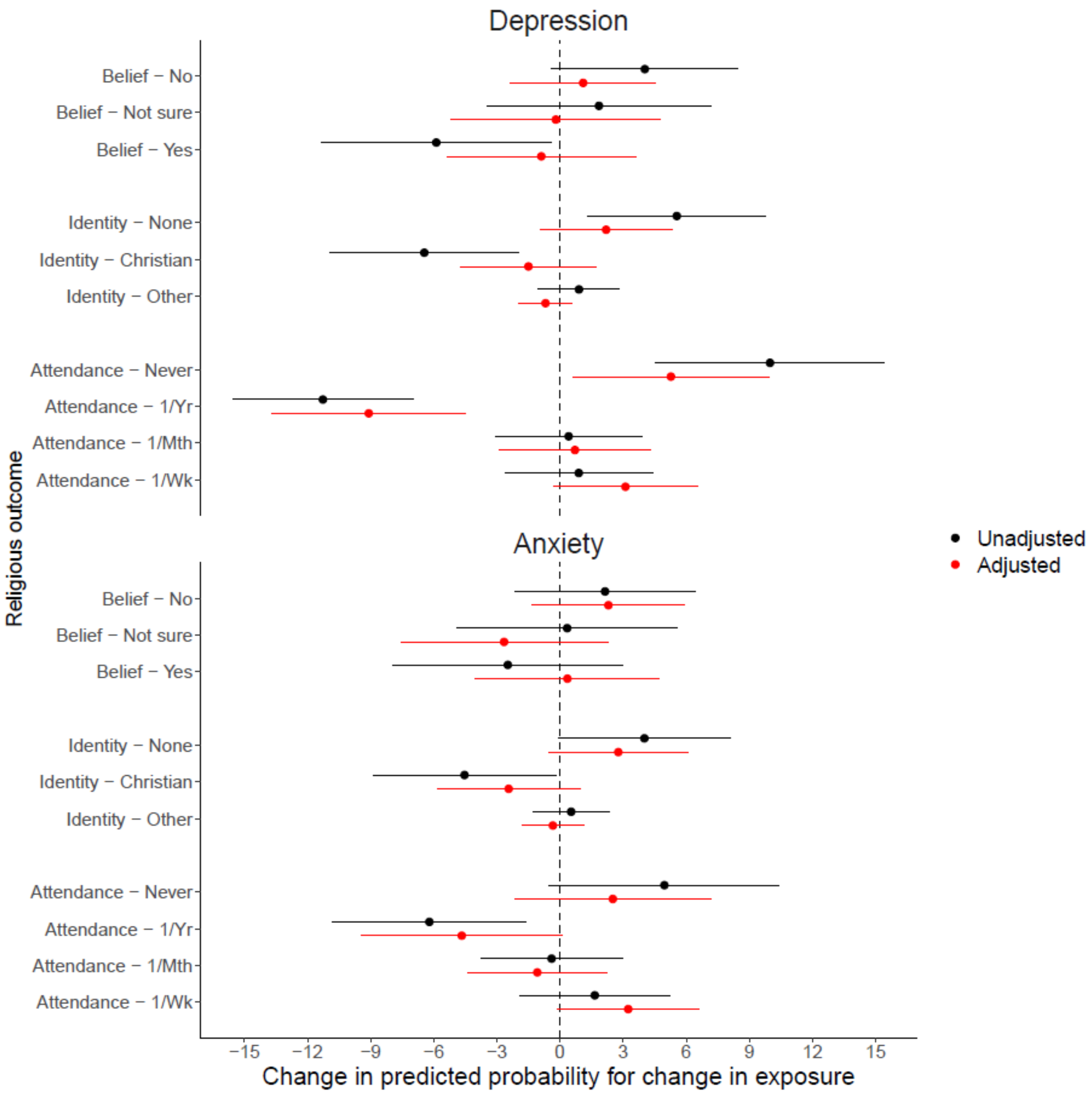

Figure S14: Results of the mothers analyses with binary probable depression and anxiety diagnoses as exposures and categorical religious/spiritual belief and behaviour (RSBB) as outcomes (*n* = 4,025). This plot displays the predicted change in the probability of the RSBB outcome for a change in the mental health exposure from 'no' to 'yes', based on the associated multinomial regression model. Results in black are from unadjusted analyses, and those in red from adjusted analyses (adjusting for baseline confounders, RSBB and mental health). The dashed vertical line at '0' indicates a null association. Error bars denote 95% confidence intervals. Full results of the multinomial models are in table S16, with predicted probabilities in table S17.

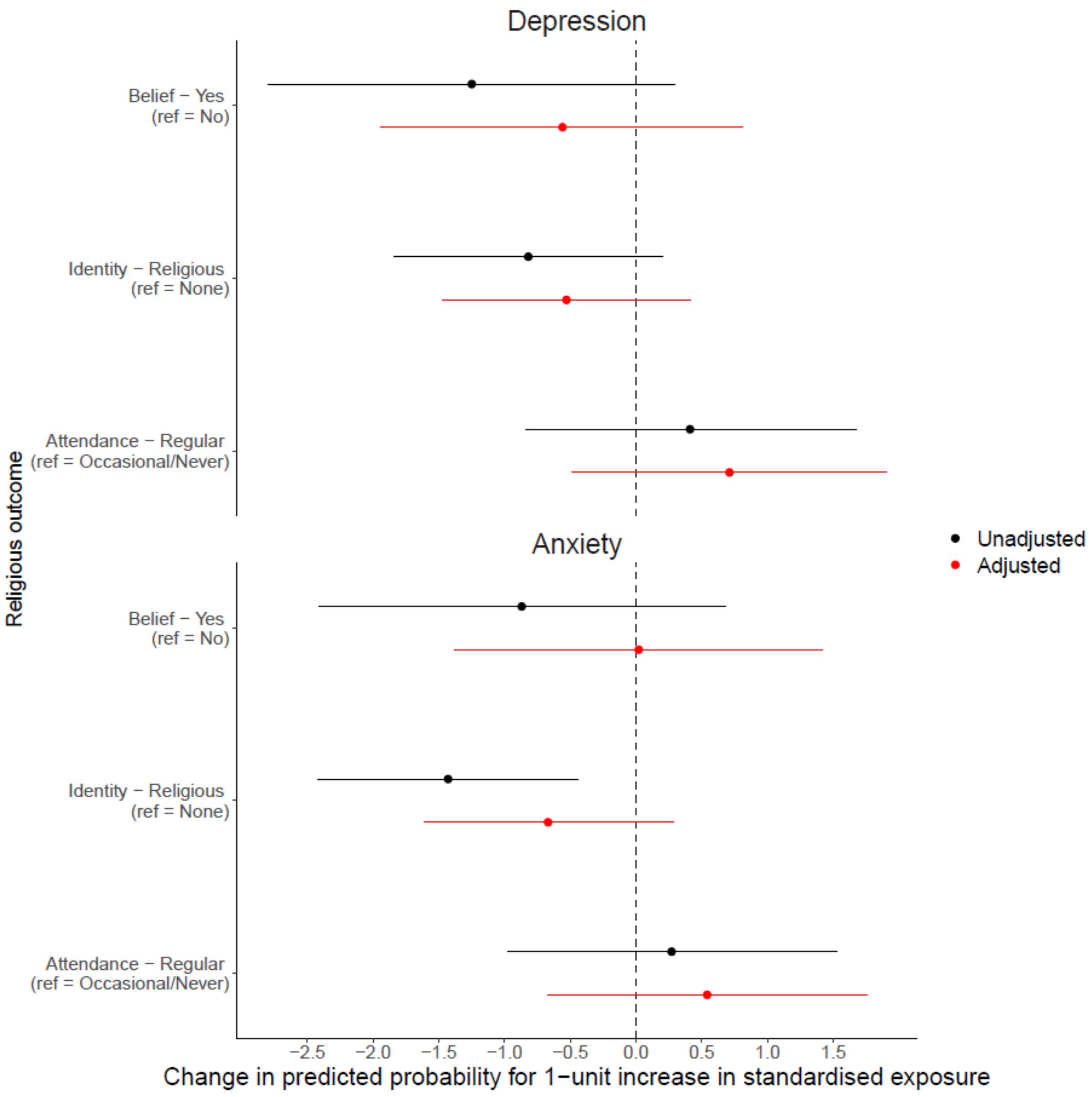

Figure S15: Results of the mothers analyses with standardised depression and anxiety scores as exposures and binary religious/spiritual belief and behaviour (RSBB) as outcomes (*n* = 4,025). This plot displays the predicted change in the probability of the RSBB outcome for a one-standardised-unit increase in the mental health exposure, based on the associated logistic regression model. Results in black are from unadjusted analyses, and those in red from adjusted analyses (adjusting for baseline confounders, RSBB and mental health). The dashed vertical line at ‘0’ indicates a null association. Error bars denote 95% confidence intervals. Full results of the logistic models are in table S16, with predicted probabilities in table S17.

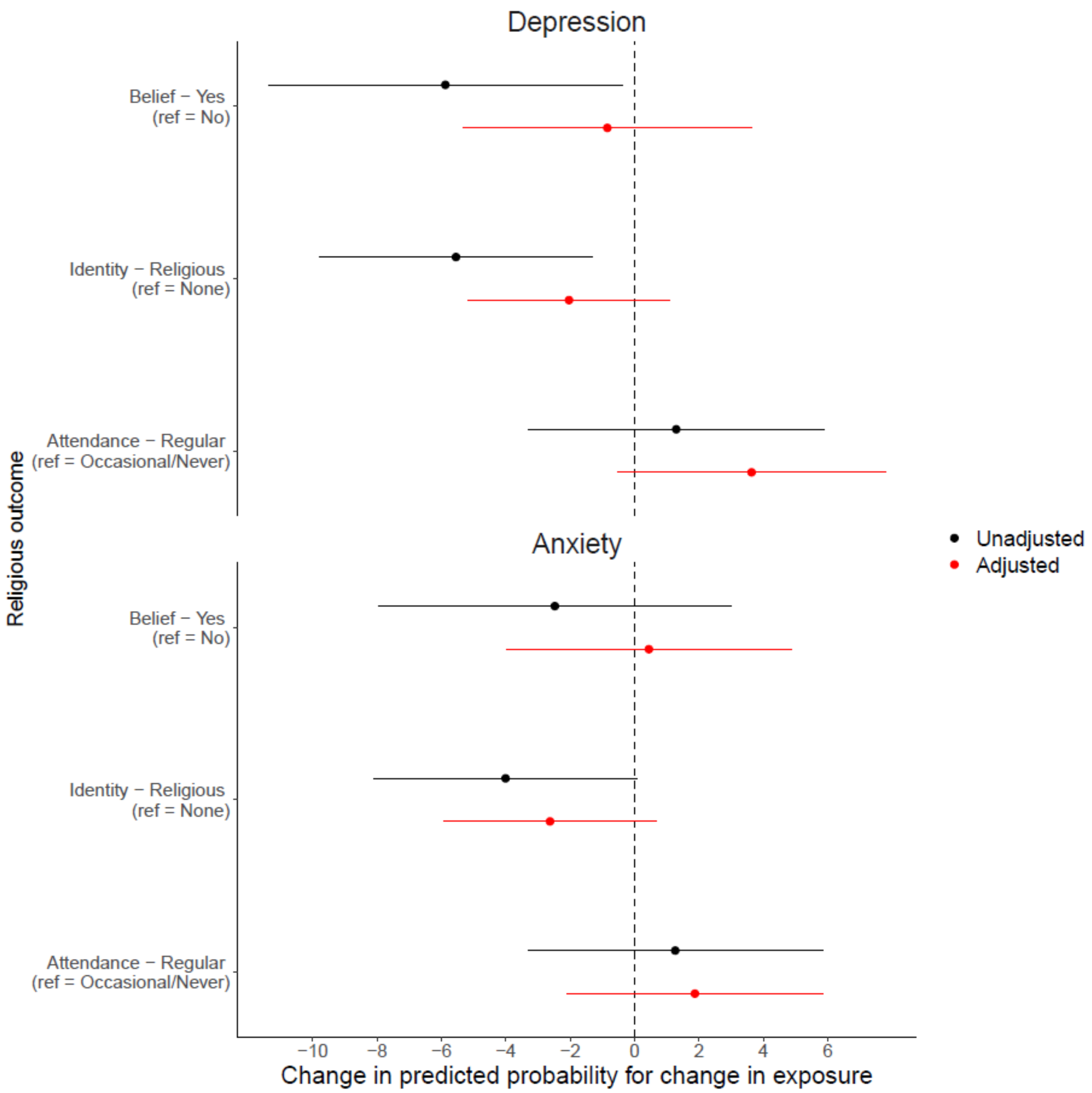

Figure S16: Results of the mothers analyses with binary probable depression and anxiety diagnoses as exposures and binary religious/spiritual belief and behaviour (RSBB) as outcomes (*n* = 4,025). This plot displays the predicted change in the probability of the RSBB outcome for a change in the mental health exposure from 'no' to 'yes', based on the associated logistic regression model. Results in black are from unadjusted analyses, and those in red from adjusted analyses (adjusting for baseline confounders, RSBB and mental health). The dashed vertical line at '0' indicates a null association. Error bars denote 95% confidence intervals. Full results of the logistic models are in table S16, with predicted probabilities in table S17.

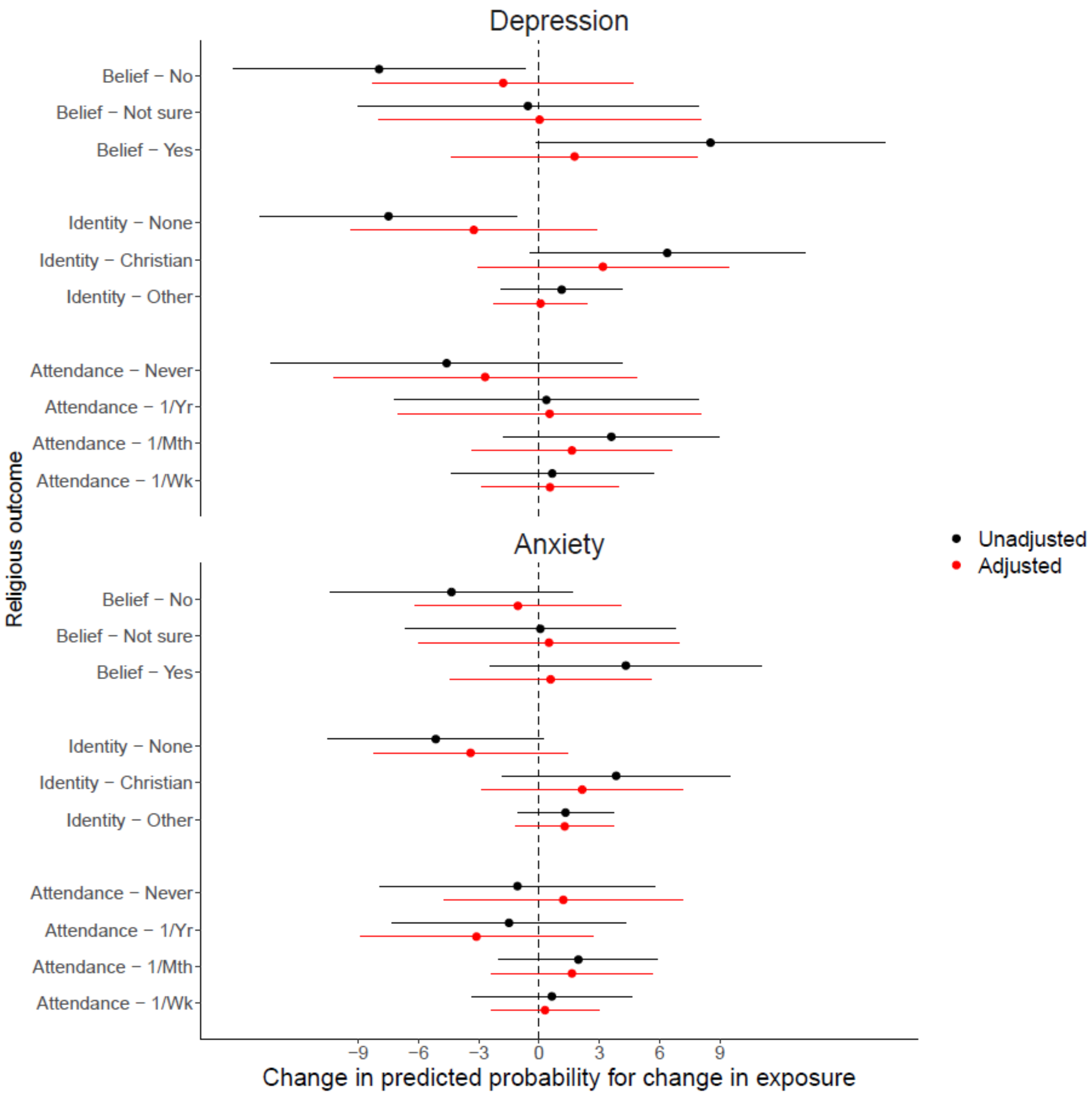

Figure S17: Results of the partners analyses with binary probable depression and anxiety diagnoses as exposures and categorical religious/spiritual belief and behaviour (RSBB) as outcomes (*n* = 2,120). This plot displays the predicted change in the probability of the RSBB outcome for a change in the mental health exposure from ‘no’ to ‘yes’, based on the associated multinomial regression model. Results in black are from unadjusted analyses, and those in red from adjusted analyses (adjusting for baseline confounders, RSBB and mental health). The dashed vertical line at ‘0’ indicates a null association. Error bars denote 95% confidence intervals. Full results of the multinomial models are in table S19, with predicted probabilities in table S20.

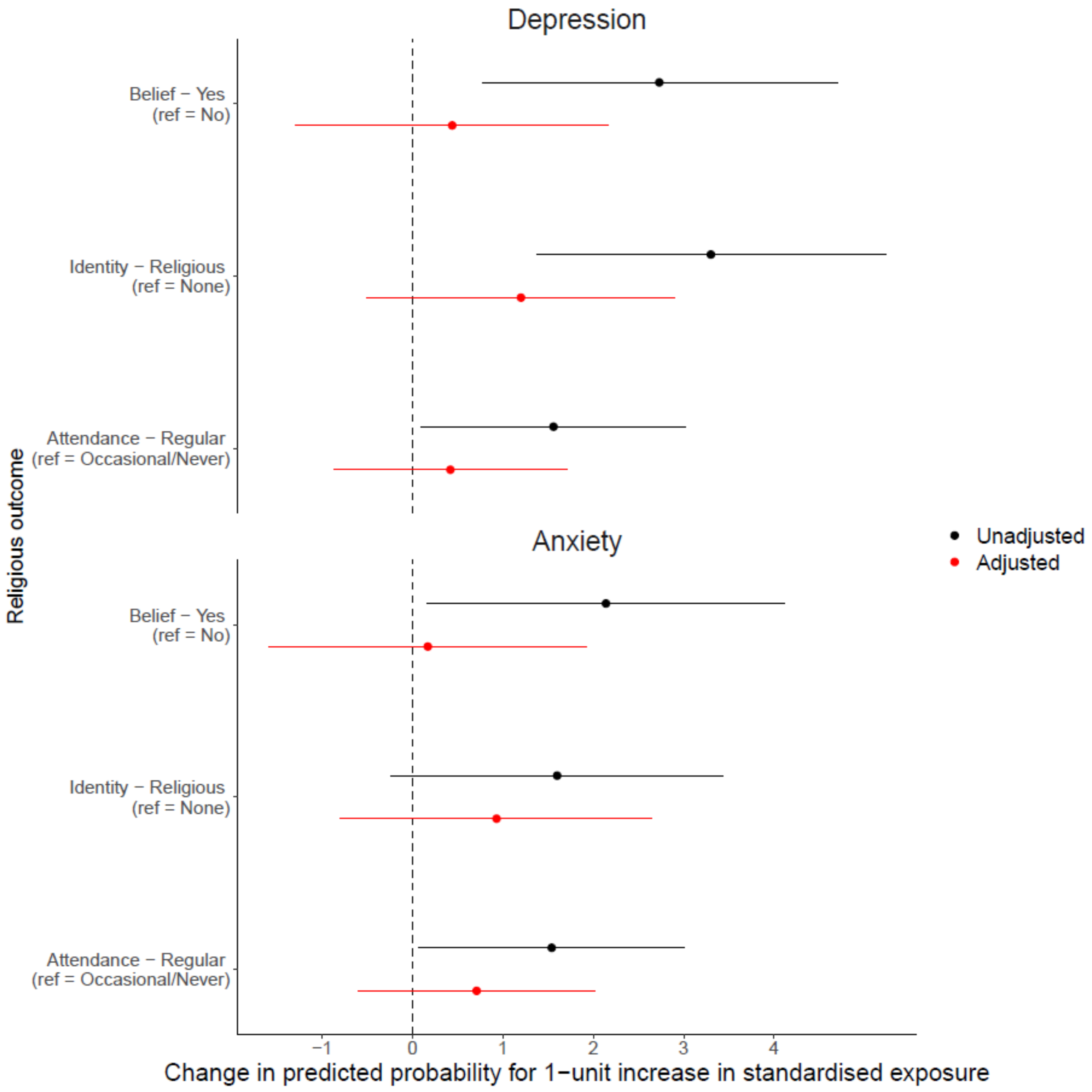

Figure S18: Results of the partners analyses with standardised depression and anxiety scores as exposures and binary religious/spiritual belief and behaviour (RSBB) as outcomes (*n* = 2,120). This plot displays the predicted change in the probability of the RSBB outcome for a one-standardised-unit increase in the mental health exposure, based on the associated logistic regression model. Results in black are from unadjusted analyses, and those in red from adjusted analyses (adjusting for baseline confounders, RSBB and mental health). The dashed vertical line at '0' indicates a null association. Error bars denote 95% confidence intervals. Full results of the logistic models are in table S19, with predicted probabilities in table S20.

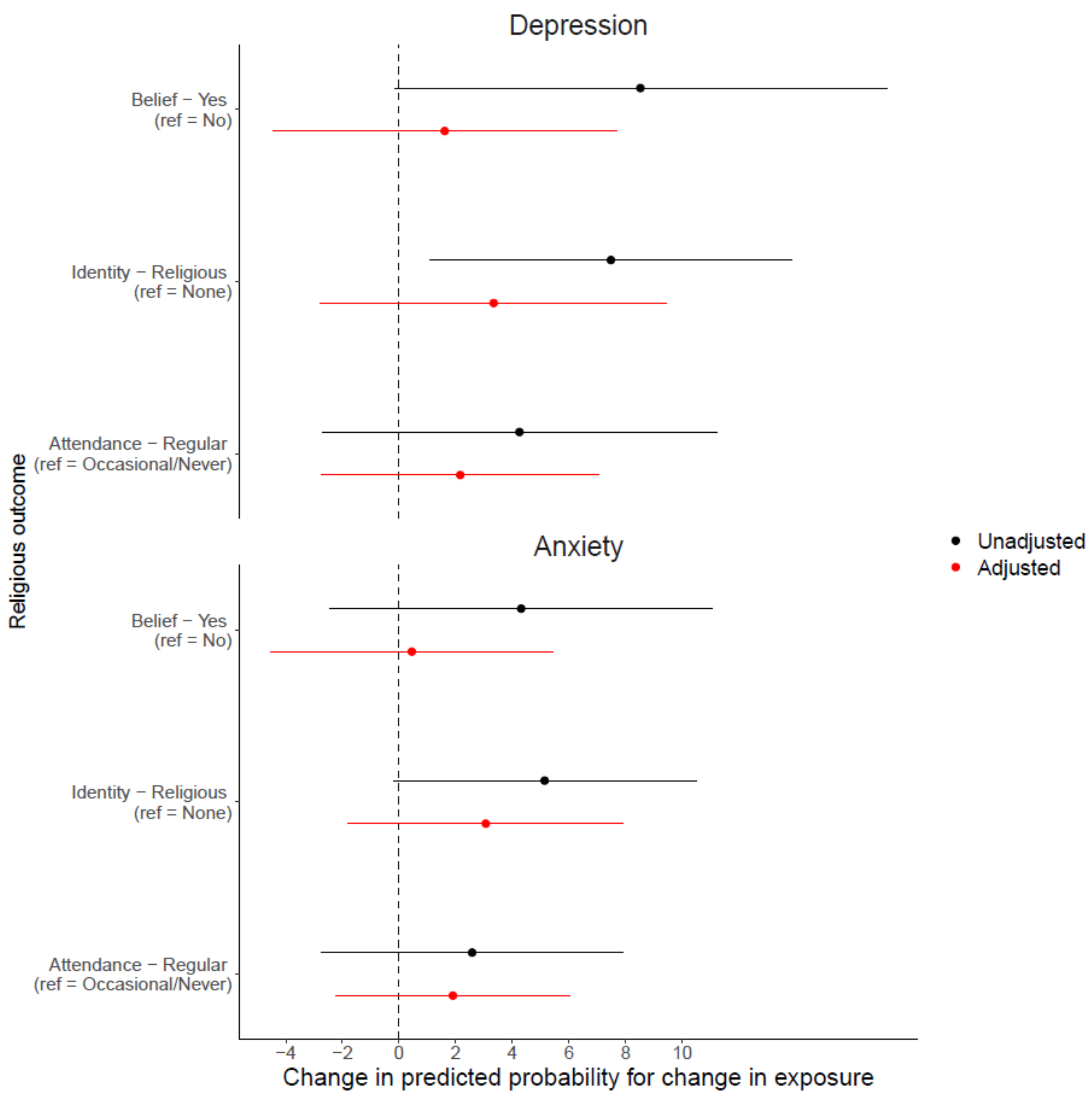

Figure S19: Results of the partners analyses with binary probable depression and anxiety diagnoses as exposures and binary religious/spiritual belief and behaviour (RSBB) as outcomes (*n* = 2,120). This plot displays the predicted change in the probability of the RSBB outcome for a change in the mental health exposure from 'no' to 'yes', based on the associated logistic regression model. Results in black are from unadjusted analyses, and those in red from adjusted analyses (adjusting for baseline confounders, RSBB and mental health). The dashed vertical line at '0' indicates a null association. Error bars denote 95% confidence intervals. Full results of the logistic models are in table S19, with predicted probabilities in table S20.

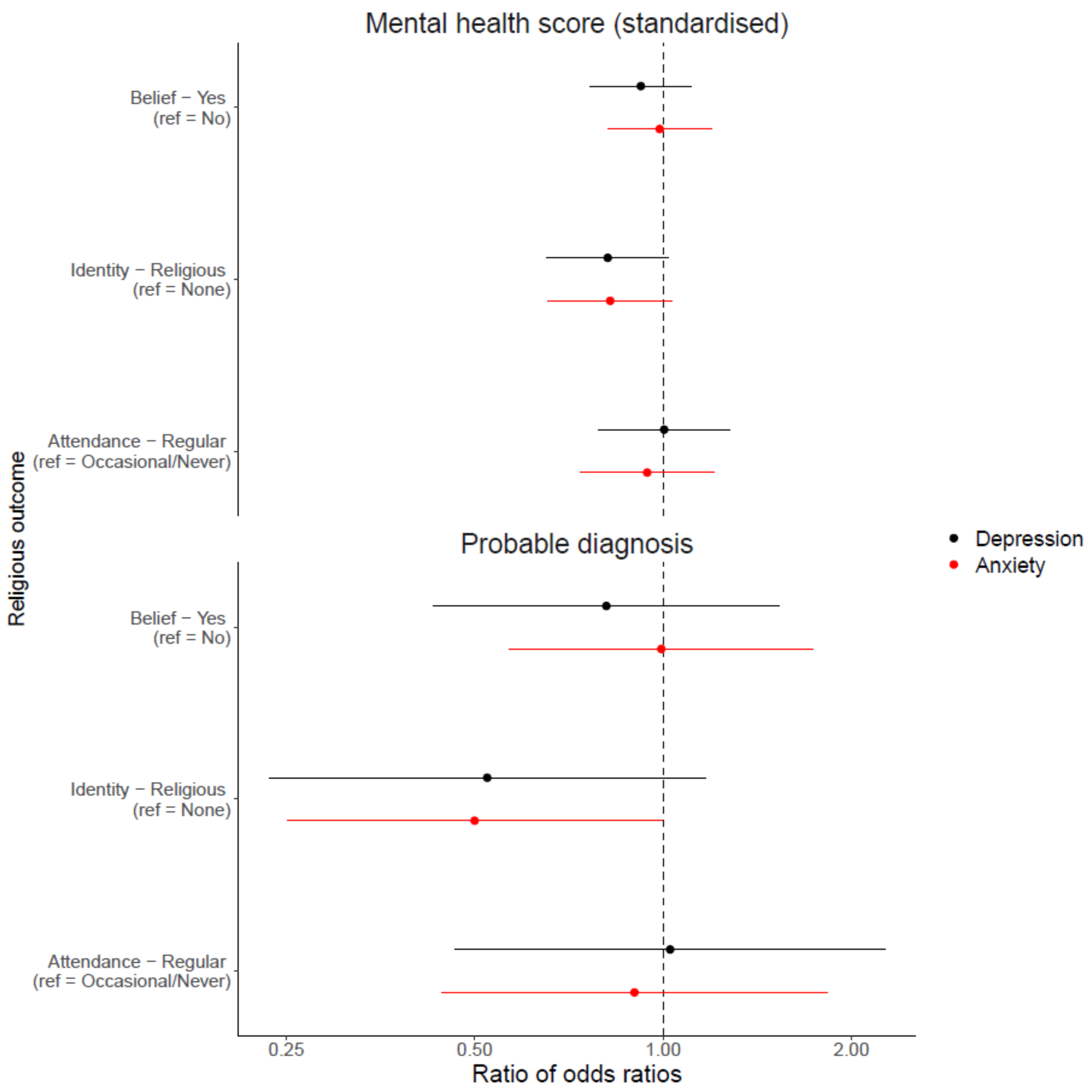

Figure S20: Results of the interaction analyses assessing whether the adjusted mother and partner results differ, with standardised continuous mental health, or binary probable depression and anxiety diagnoses, as exposures and binary religious/spiritual beliefs and behaviours (RSBB) as outcomes. Results in black are for the depression outcome, and those in red for anxiety. The dashed vertical line at '1' indicates no difference between mothers and partners, with results below 1 meaning that the odds ratio (OR) estimate was lower in mothers, compared to partners. For instance, for 'Religious' affiliation (relative to 'None') and continuous depression scores, a one-standardised-unit increase in depression was associated with an OR of 0.92 in mothers, while in partners it was 1.13, giving a ratio of these ORs here of approximately 0.82. Error bars denote 95% confidence intervals. Full results are in table S22.

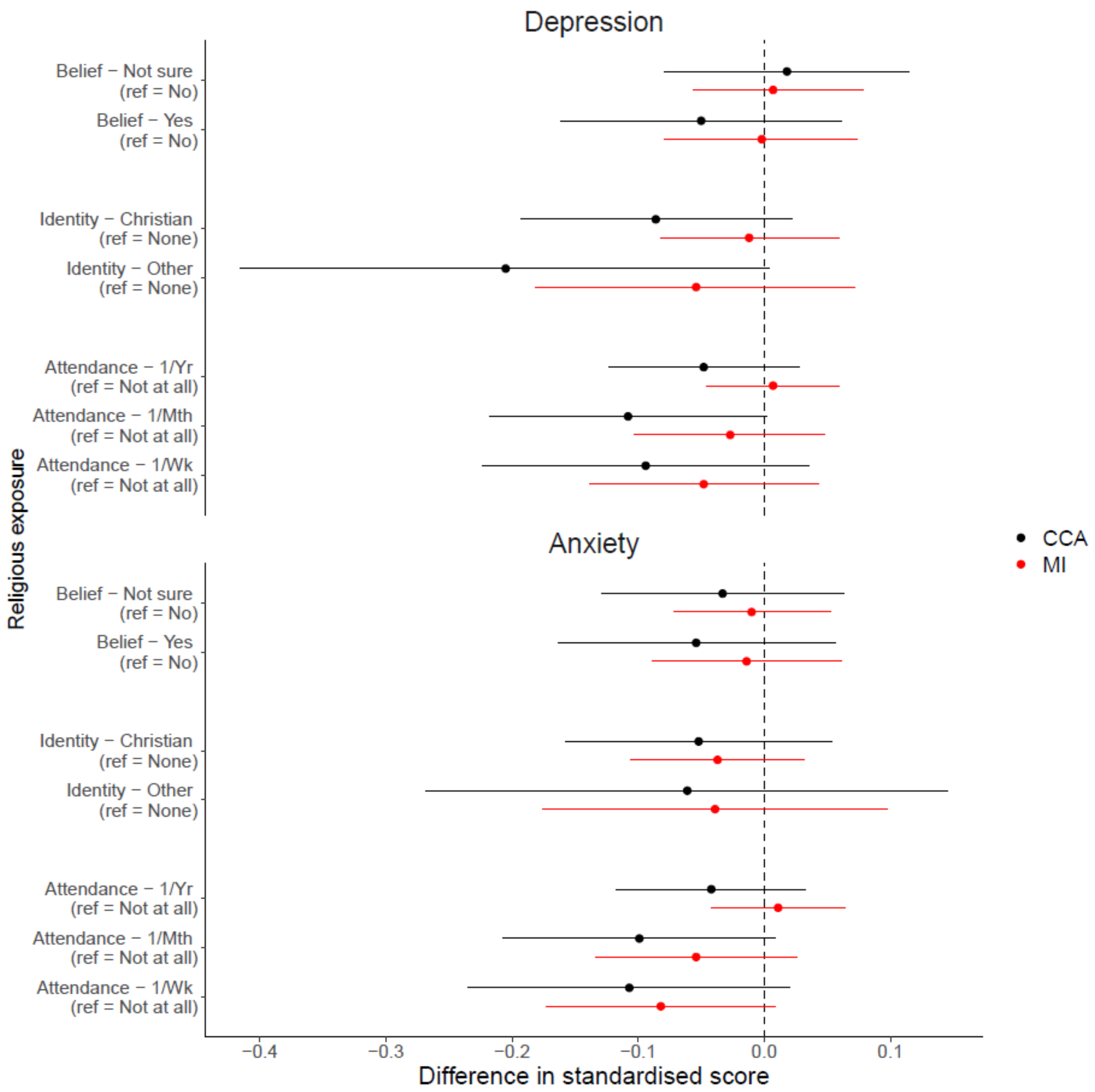

Figure S21: Results of the mothers analyses with categorical religious/spiritual belief and behaviour (RSBB) exposures and standardised depression and anxiety scores as outcomes comparing complete-case analysis (CCA; *n* = 3,856) and multiple imputation (MI; *n* = 13,085) results. Results are for adjusted analyses, with CCA results in black and MI results in red. The dashed vertical line at '0' indicates a null association. Error bars denote 95% confidence intervals. Full results are in Table S8 (for CCA) and Table S23 (for MI).

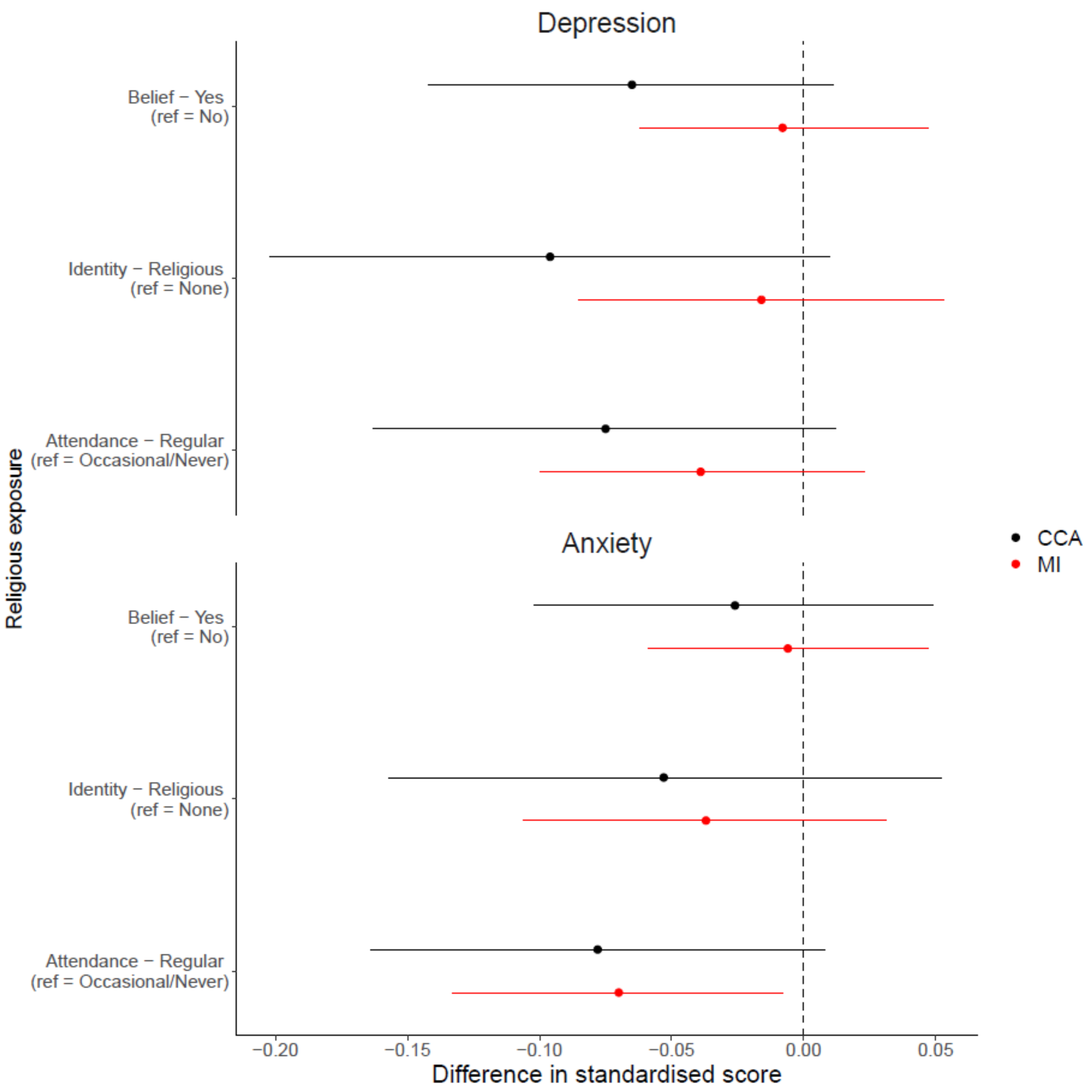

Figure S22: Results of the mothers analyses with binary religious/spiritual belief and behaviour (RSBB) exposures and standardised depression and anxiety scores as outcomes comparing complete-case analysis (CCA; *n* = 3,856) and multiple imputation (MI; *n* = 13,085) results. Results are for adjusted analyses, with CCA results in black and MI results in red. The dashed vertical line at '0' indicates a null association. Error bars denote 95% confidence intervals. Full results are in Table S8 (for CCA) and Table S23 (for MI).

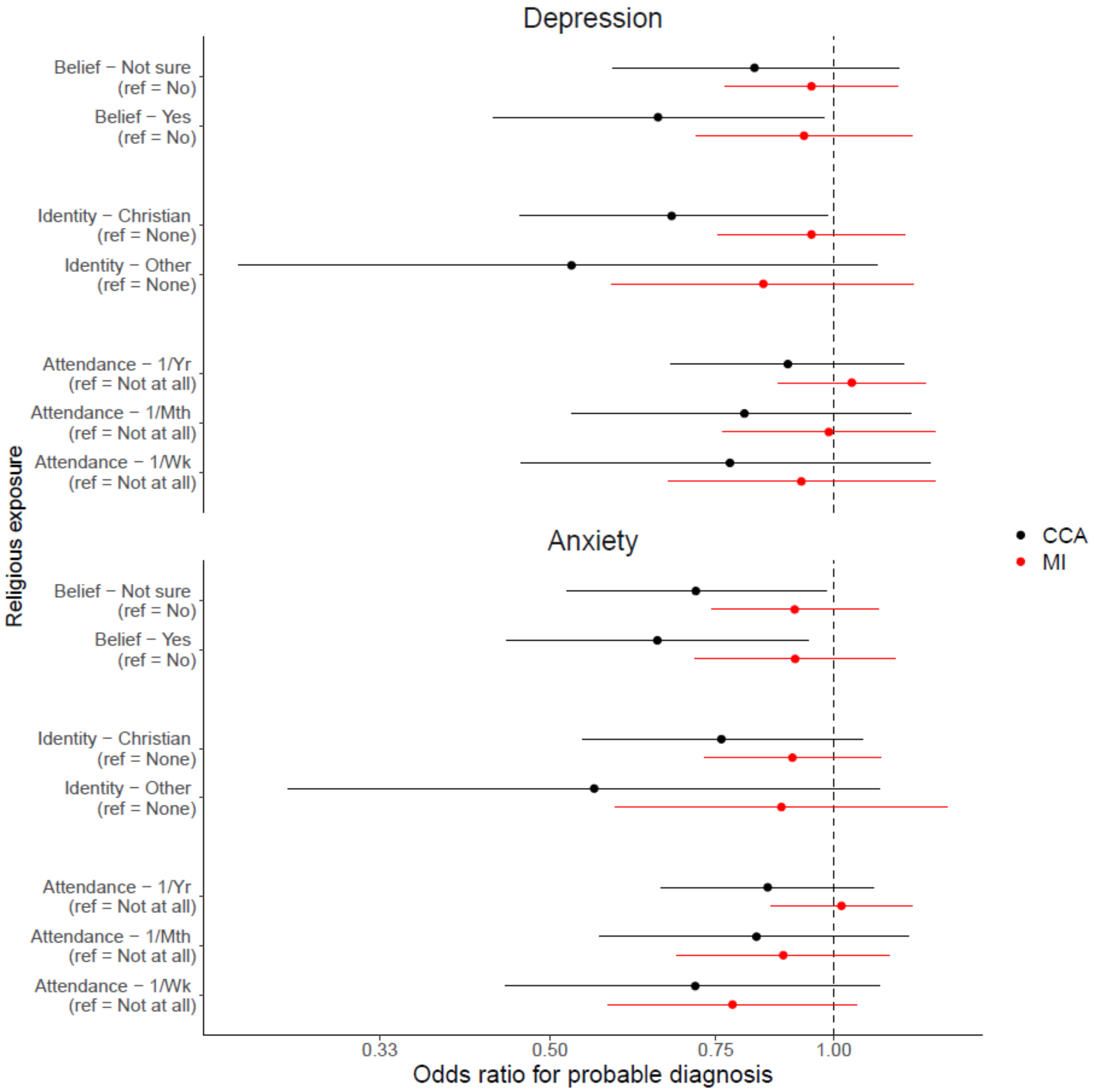

Figure S23: Results of the mothers analyses with categorical religious/spiritual belief and behaviour (RSBB) exposures and binary probable depression and anxiety diagnoses as outcomes, using logistic regression comparing complete-case analysis (CCA; *n* = 3,856) and multiple imputation (MI; *n* = 13,085) results. Results are for adjusted analyses, with CCA results in black and MI results in red. The dashed vertical line at '1' indicates a null association. Error bars denote 95% confidence intervals. Full results are in Table S8 (for CCA) and Table S23 (for MI).

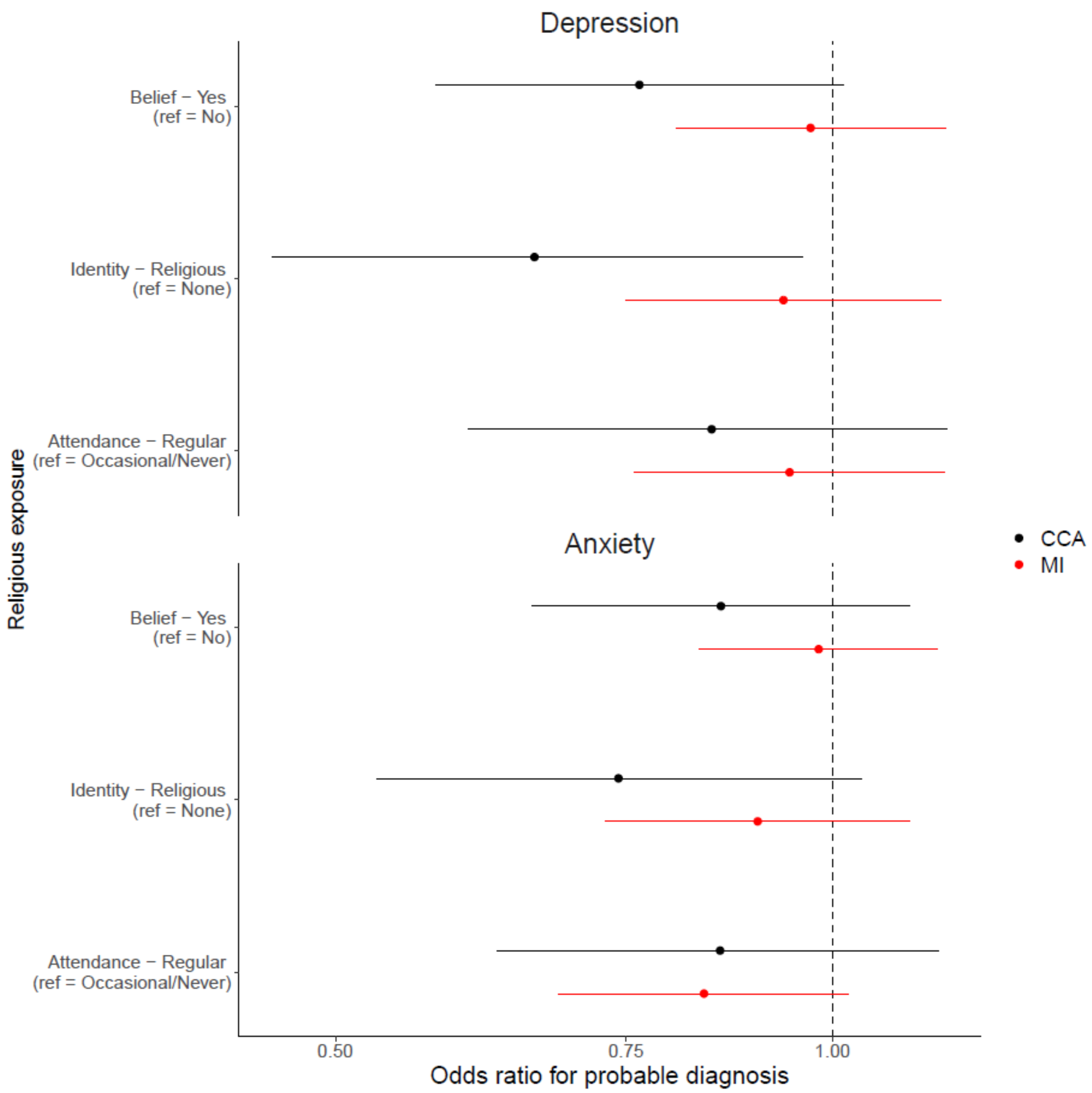

Figure S24: Results of the mothers analyses with binary religious/spiritual belief and behaviour (RSBB) exposures and binary probable depression and anxiety diagnoses as outcomes, using logistic regression comparing complete-case analysis (CCA; *n* = 3,856) and multiple imputation (MI; *n* = 13,085) results. Results are for adjusted analyses, with CCA results in black and MI results in red. The dashed vertical line at '1' indicates a null association. Error bars denote 95% confidence intervals. Full results are in Table S8 (for CCA) and Table S23 (for MI).

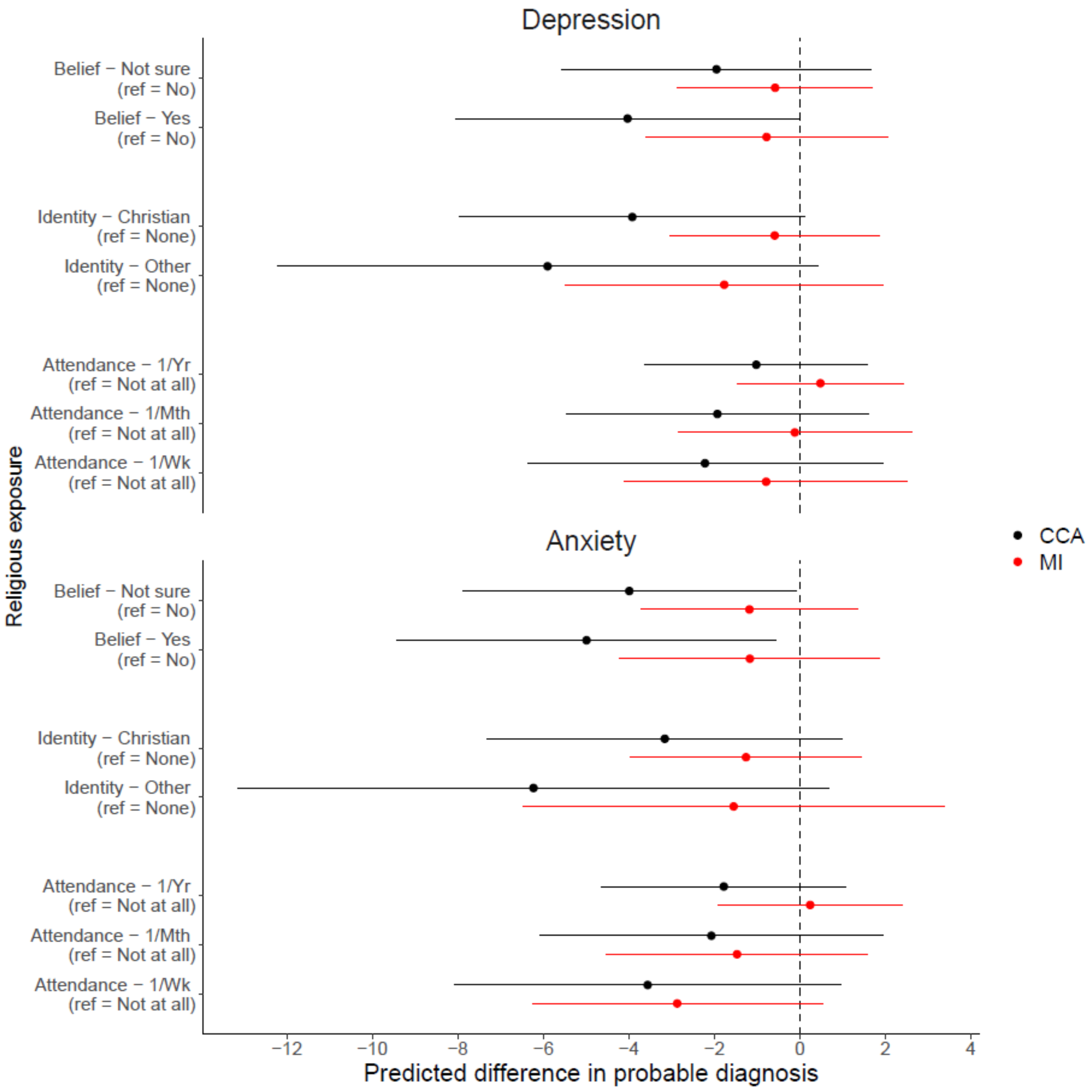

Figure S25: Predicted probabilities of the mothers analyses with categorical religious/spiritual belief and behaviour (RSBB) exposures and binary probable depression and anxiety diagnoses as outcomes, using logistic regression comparing complete-case analysis (CCA; *n* = 3,856) and multiple imputation (MI; *n* = 13,085) results. Results are for adjusted analyses, with CCA results in black and MI results in red. The dashed vertical line at '0' indicates no difference in the predicted probability. Error bars denote 95% confidence intervals. Full results are in Table S9 (for CCA) and Table S24 (for MI).

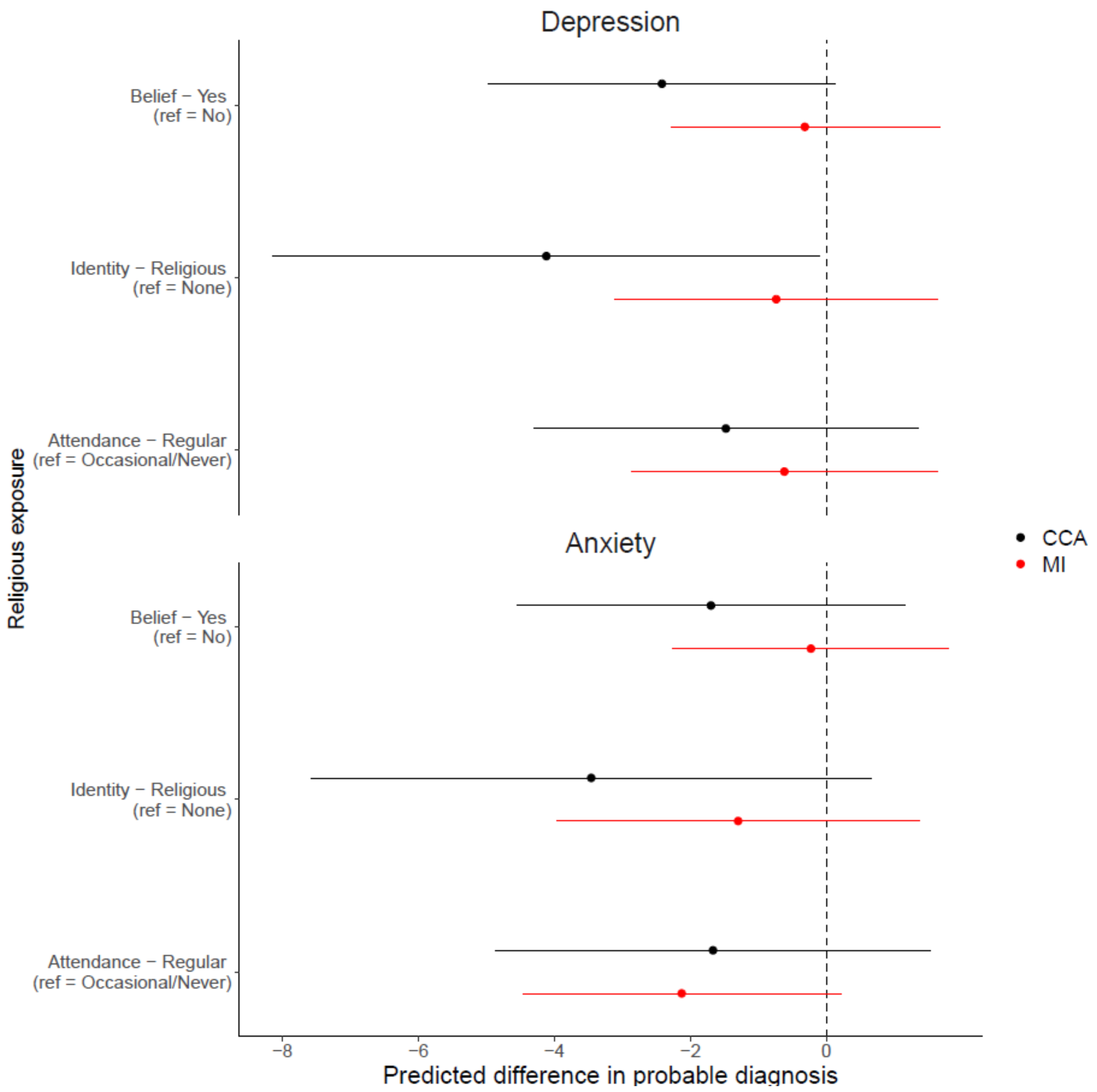

Figure S26: Predicted probabilities of the mothers analyses with binary religious/spiritual belief and behaviour (RSBB) exposures and binary probable depression and anxiety diagnoses as outcomes, using logistic regression comparing complete-case analysis (CCA; *n* = 3,856) and multiple imputation (MI; *n* = 13,085) results. Results are for adjusted analyses, with CCA results in black and MI results in red. The dashed vertical line at '0' indicates no difference in the predicted probability. Error bars denote 95% confidence intervals. Full results are in Table S9 (for CCA) and Table S24 (for MI).

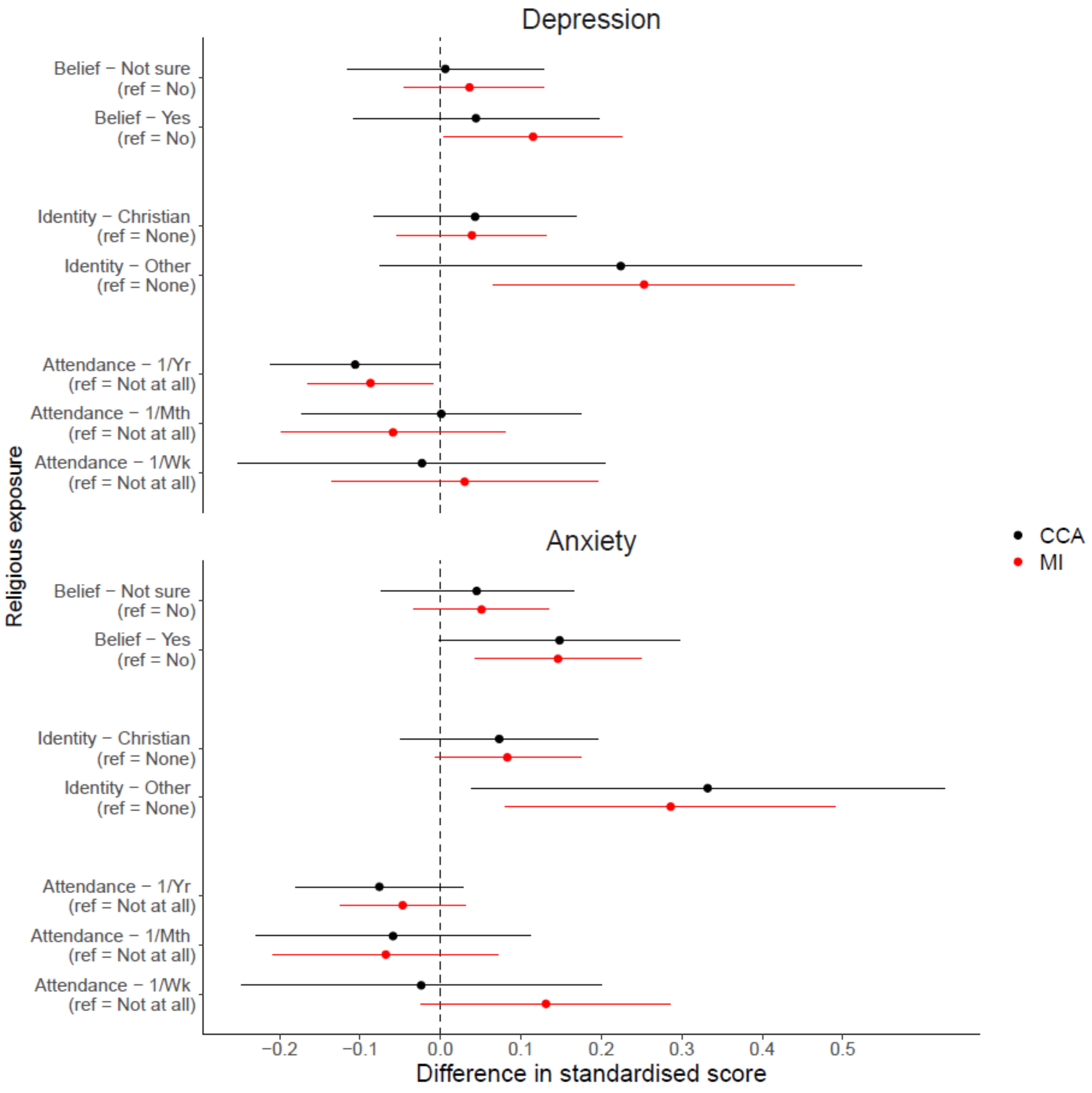

Figure S27: Results of the partners analyses with categorical religious/spiritual belief and behaviour (RSBB) exposures and standardised depression and anxiety scores as outcomes comparing complete-case analysis (CCA; *n* = 1,940) and multiple imputation (MI; *n* = 9,745) results. Results are for adjusted analyses, with CCA results in black and MI results in red. The dashed vertical line at '0' indicates a null association. Error bars denote 95% confidence intervals. Full results are in Table S11 (for CCA) and Table S25 (for MI).

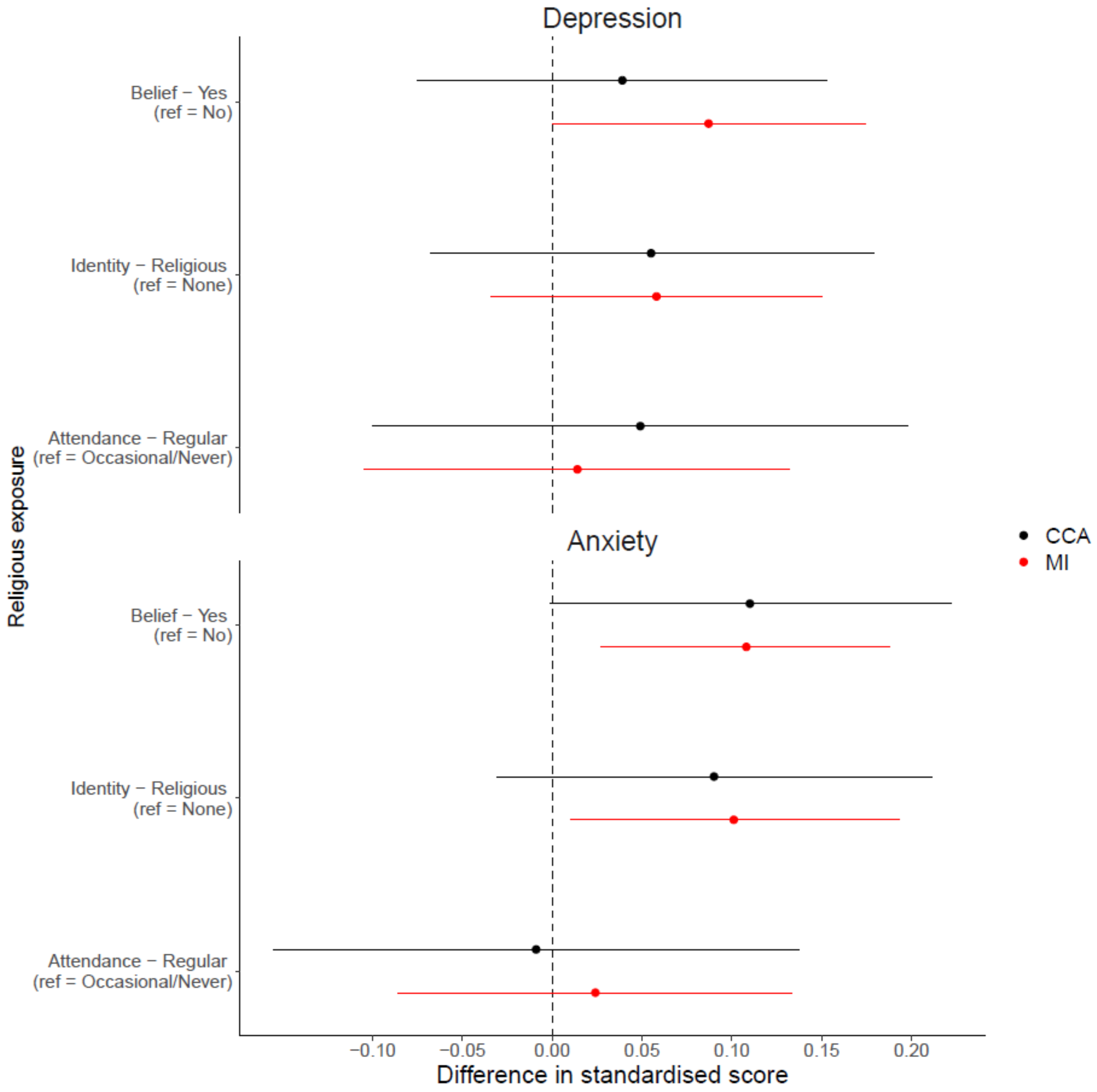

Figure S28: Results of the partners analyses with binary religious/spiritual belief and behaviour (RSBB) exposures and standardised depression and anxiety scores as outcomes comparing complete-case analysis (CCA; *n* = 1,940) and multiple imputation (MI; *n* = 9,745) results. Results are for adjusted analyses, with CCA results in black and MI results in red. The dashed vertical line at ‘0’ indicates a null association. Error bars denote 95% confidence intervals. Full results are in Table S11 (for CCA) and Table S25 (for MI).

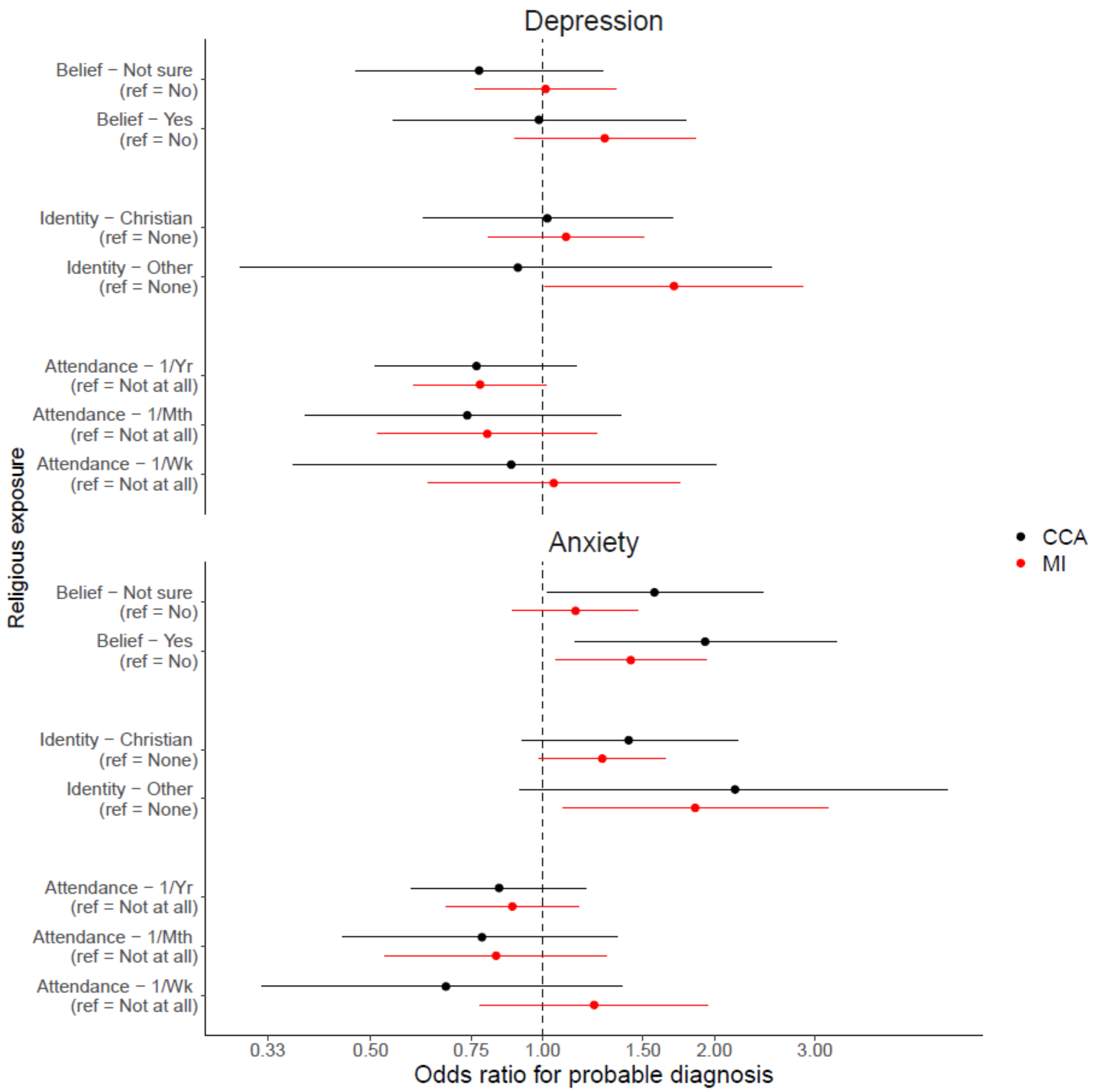

Figure S29: Results of the partners analyses with categorical religious/spiritual belief and behaviour (RSBB) exposures and binary probable depression and anxiety diagnoses as outcomes, using logistic regression comparing complete-case analysis (CCA; *n* = 1,940) and multiple imputation (MI; *n* = 9,745) results. Results are for adjusted analyses, with CCA results in black and MI results in red. The dashed vertical line at '1' indicates a null association. Error bars denote 95% confidence intervals. Full results are in Table S11 (for CCA) and Table S25 (for MI).

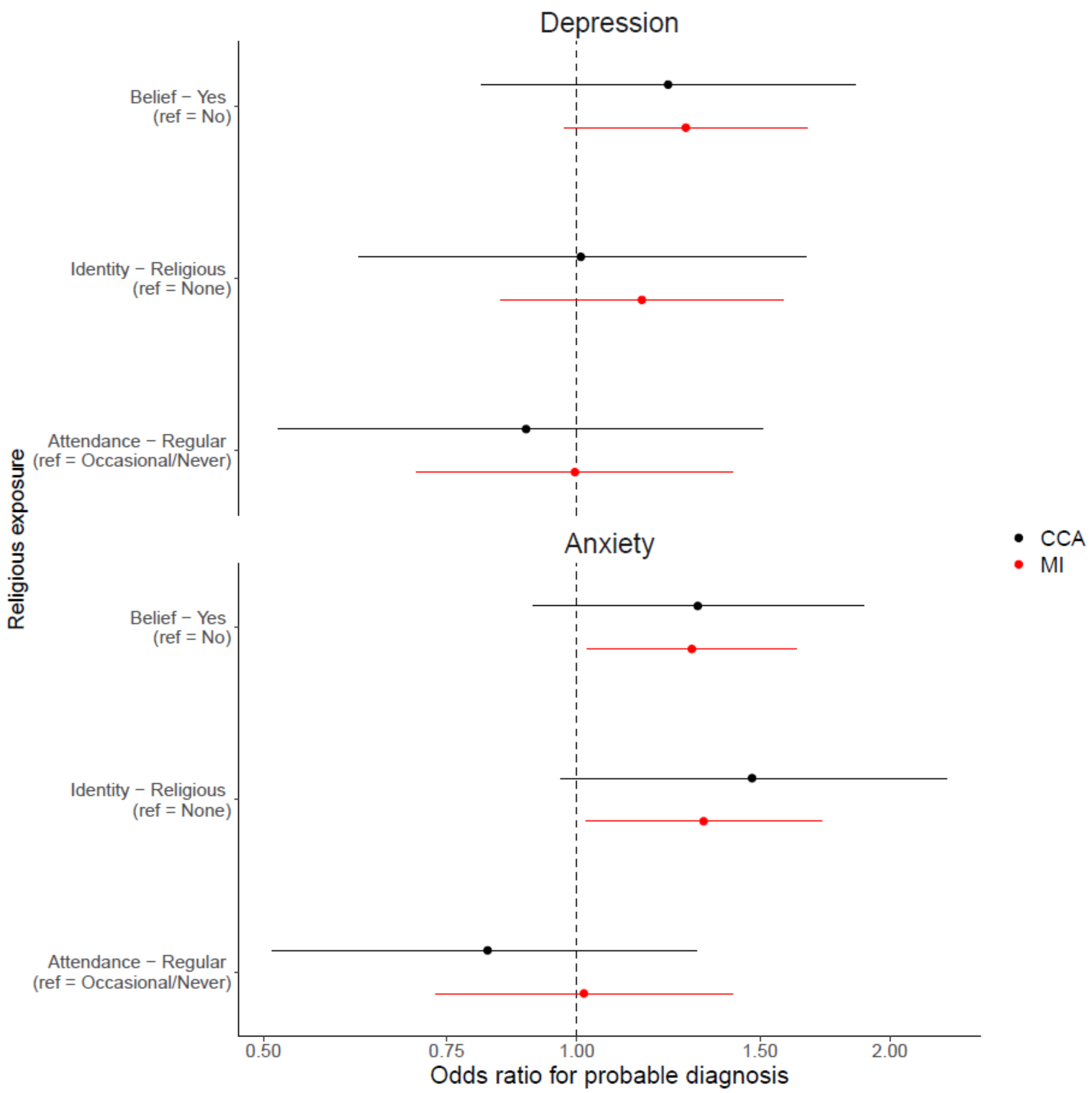

Figure S30: Results of the partners analyses with binary religious/spiritual belief and behaviour (RSBB) exposures and binary probable depression and anxiety diagnoses as outcomes, using logistic regression comparing complete-case analysis (CCA; *n* = 1,940) and multiple imputation (MI; *n* = 9,745) results. Results are for adjusted analyses, with CCA results in black and MI results in red. The dashed vertical line at '1' indicates a null association. Error bars denote 95% confidence intervals. Full results are in Table S11 (for CCA) and Table S25 (for MI).

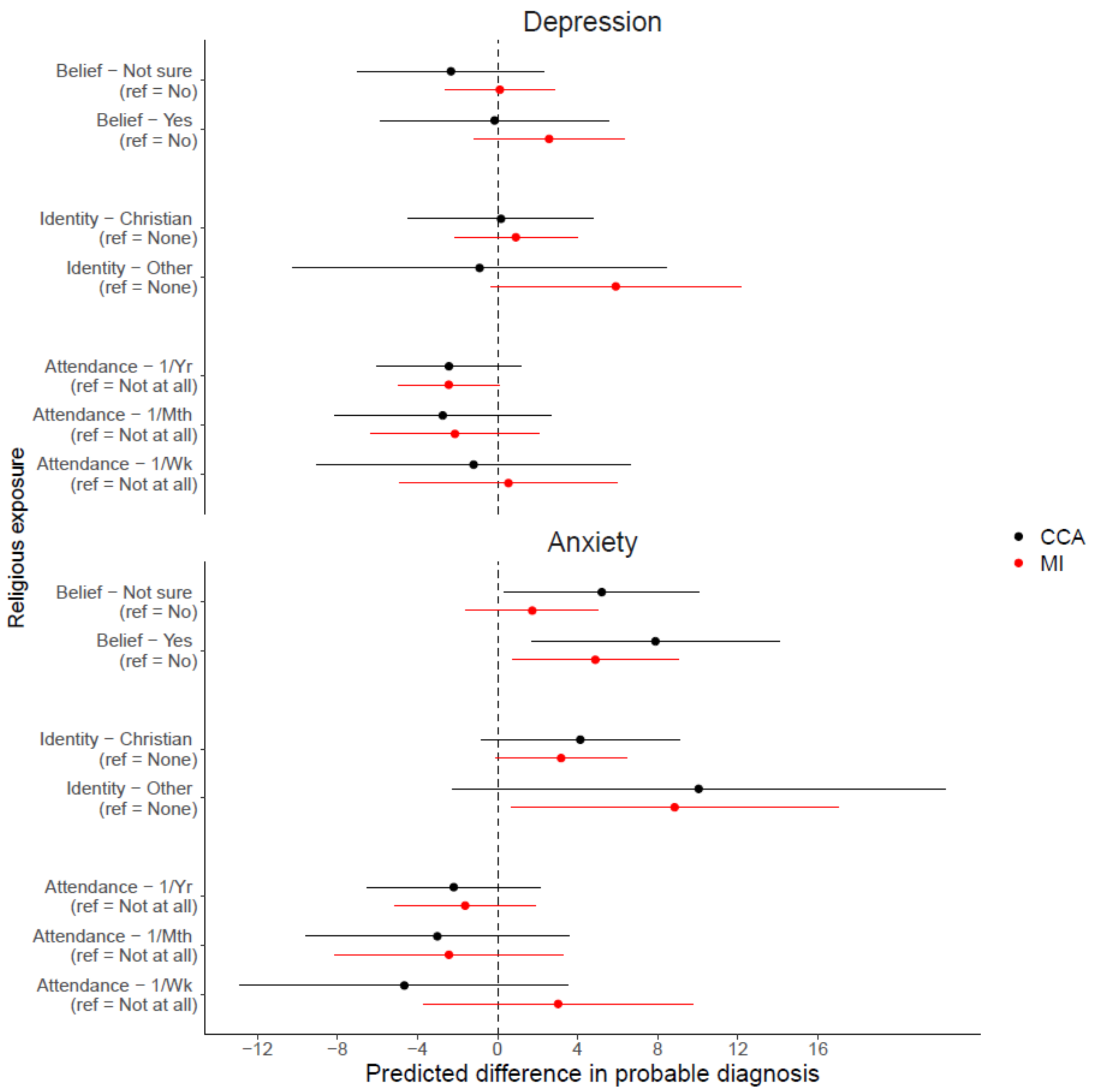

Figure S31: Predicted probabilities of the partners analyses with categorical religious/spiritual belief and behaviour (RSBB) exposures and binary probable depression and anxiety diagnoses as outcomes, using logistic regression comparing complete-case analysis (CCA; *n* = 1,940) and multiple imputation (MI; *n* = 9,745) results. Results are for adjusted analyses, with CCA results in black and MI results in red. The dashed vertical line at '0' indicates no difference in the predicted probability. Error bars denote 95% confidence intervals. Full results are in Table S12 (for CCA) and Table S26 (for MI).

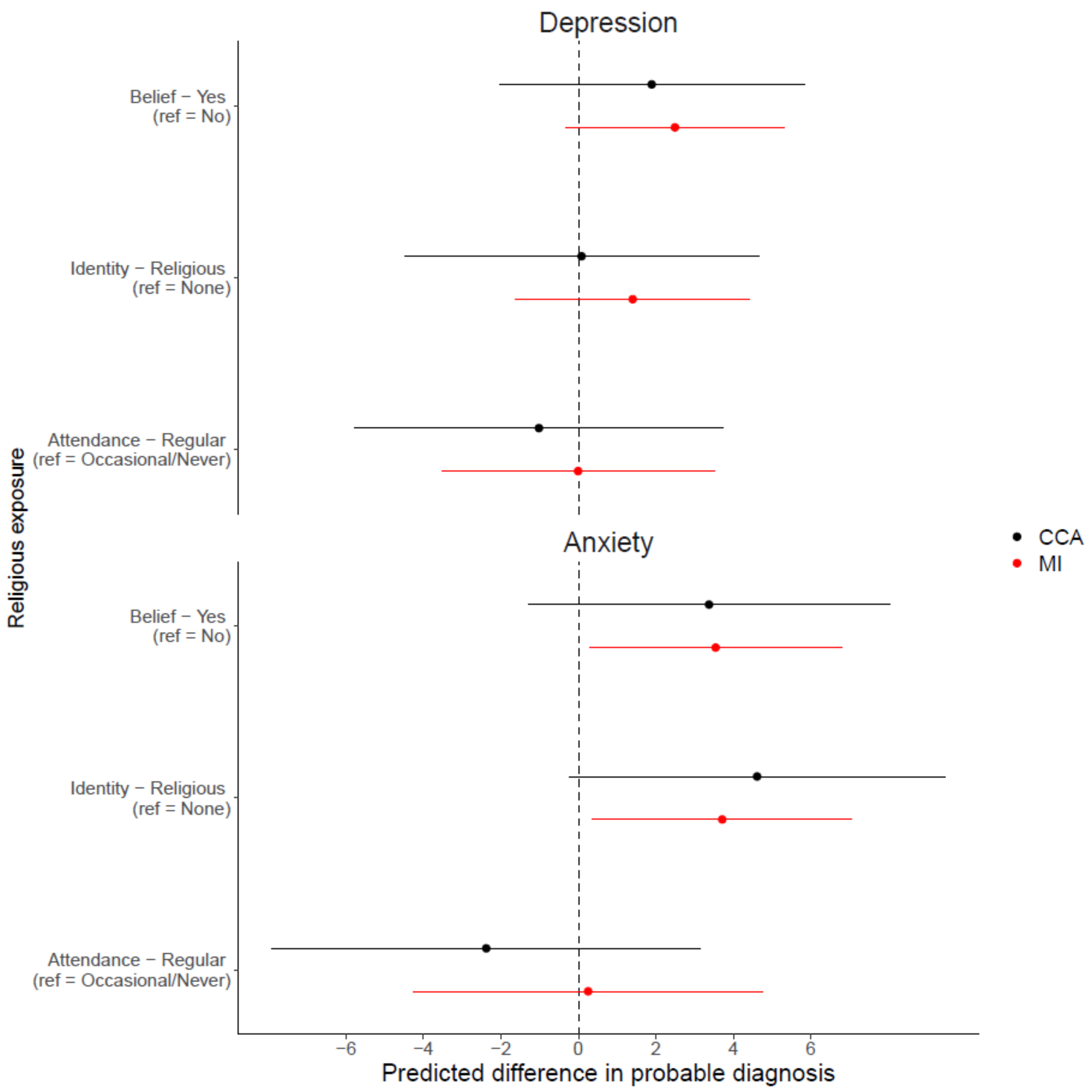

Figure S32: Predicted probabilities of the partners analyses with binary religious/spiritual belief and behaviour (RSBB) exposures and binary probable depression and anxiety diagnoses as outcomes, using logistic regression comparing complete-case analysis (CCA; *n* = 1,940) and multiple imputation (MI; *n* = 9,745) results. Results are for adjusted analyses, with CCA results in black and MI results in red. The dashed vertical line at ‘0’ indicates no difference in the predicted probability. Error bars denote 95% confidence intervals. Full results are in Table S12 (for CCA) and Table S26 (for MI).

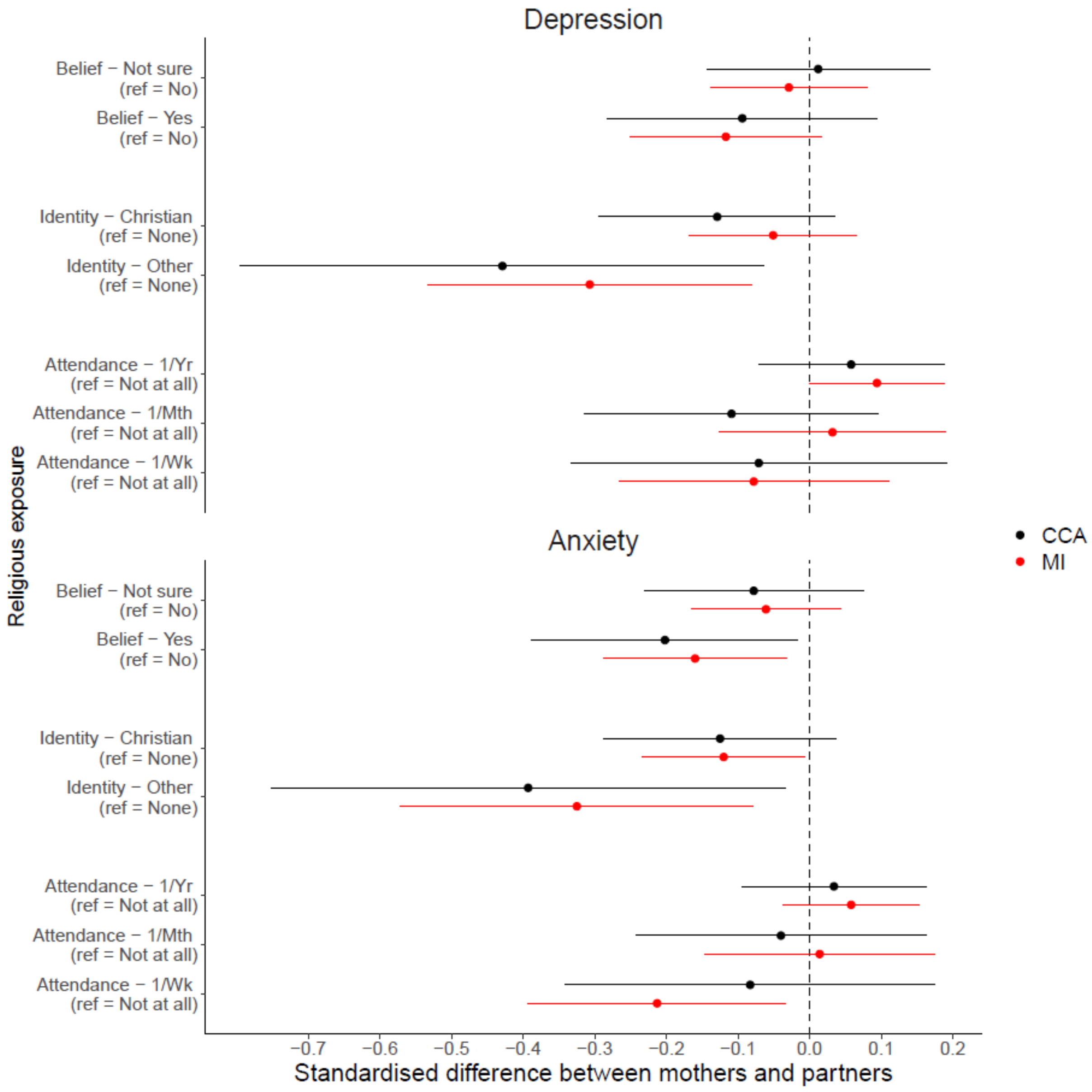

Figure S33: Results of the interaction analyses assessing whether the adjusted mother and partner results differ, with categorical religious/spiritual belief and behaviour (RSBB) exposures and standardised depression and anxiety scores as outcomes comparing complete-case analysis (CCA) and multiple imputation (MI) results. Results in black are for the CCA, and those in red for MI. The dashed vertical line at '0' indicates no difference between mothers and partners, with results below 0 meaning that the estimate was lower in mothers, compared to partners. Error bars denote 95% confidence intervals. Full results are in Table S13 (for CCA) and Table S27 (for MI).

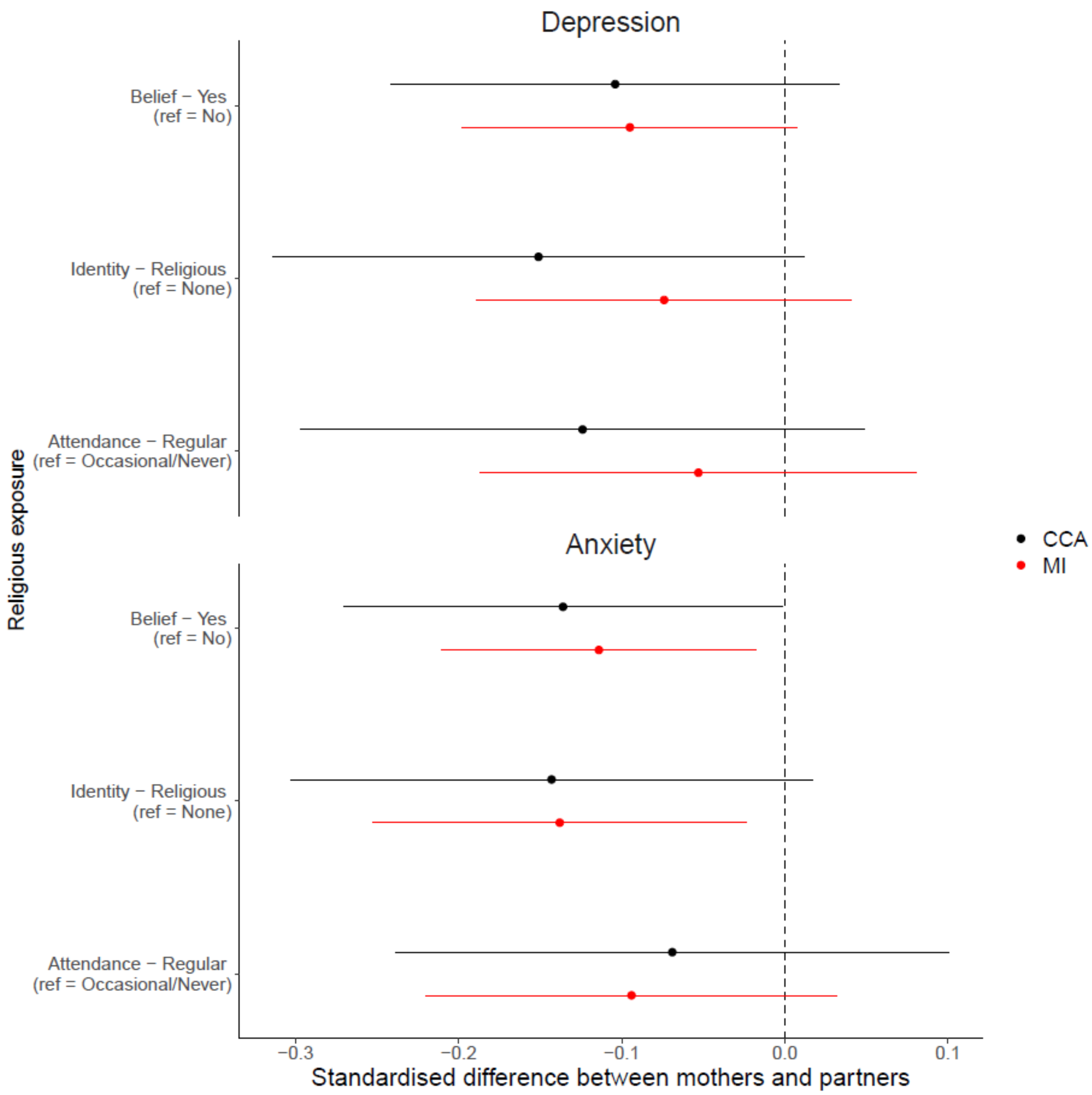

Figure S34: Results of the interaction analyses assessing whether the adjusted mother and partner results differ, with binary religious/spiritual belief and behaviour (RSBB) exposures and standardised depression and anxiety scores as outcomes comparing complete-case analysis (CCA) and multiple imputation (MI) results. Results in black are for the CCA, and those in red for MI. The dashed vertical line at ‘0’ indicates no difference between mothers and partners, with results below 0 meaning that the estimate was lower in mothers, compared to partners. Error bars denote 95% confidence intervals. Full results are in Table S13 (for CCA) and Table S27 (for MI).

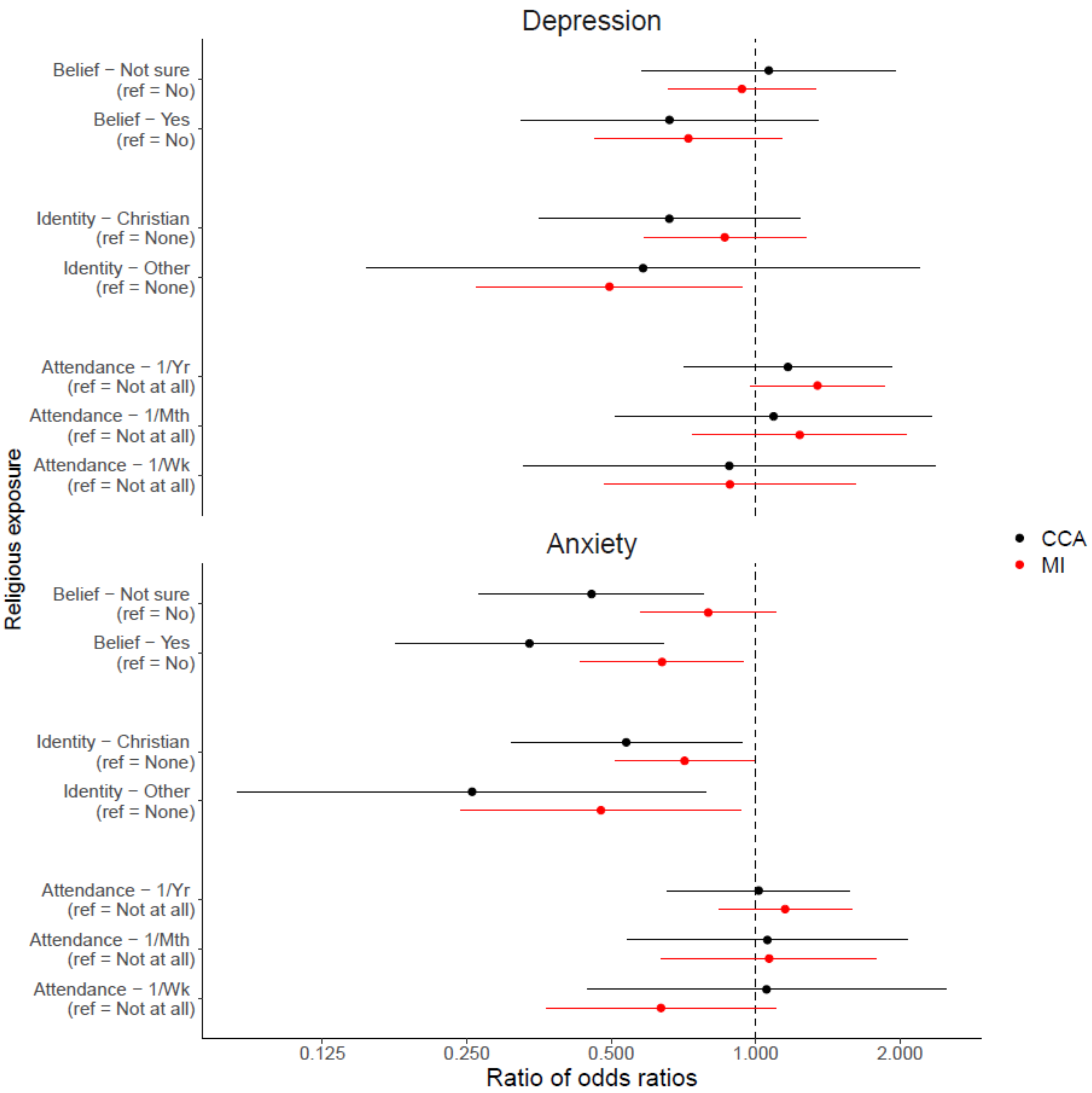

Figure S35: Results of the interaction analyses assessing whether the adjusted mother and partner results differ, with categorical religious/spiritual belief and behaviour (RSBB) exposures and binary probable depression and anxiety diagnoses as outcomes comparing complete-case analysis (CCA) and multiple imputation (MI) results. Results in black are for the CCA, and those in red for MI. The dashed vertical line at '1' indicates no difference between mothers and partners, with results below 1 meaning that the odds ratio estimate was lower in mothers, compared to partners. Error bars denote 95% confidence intervals. Full results are in Table S14 (for CCA) and Table S28 (for MI).

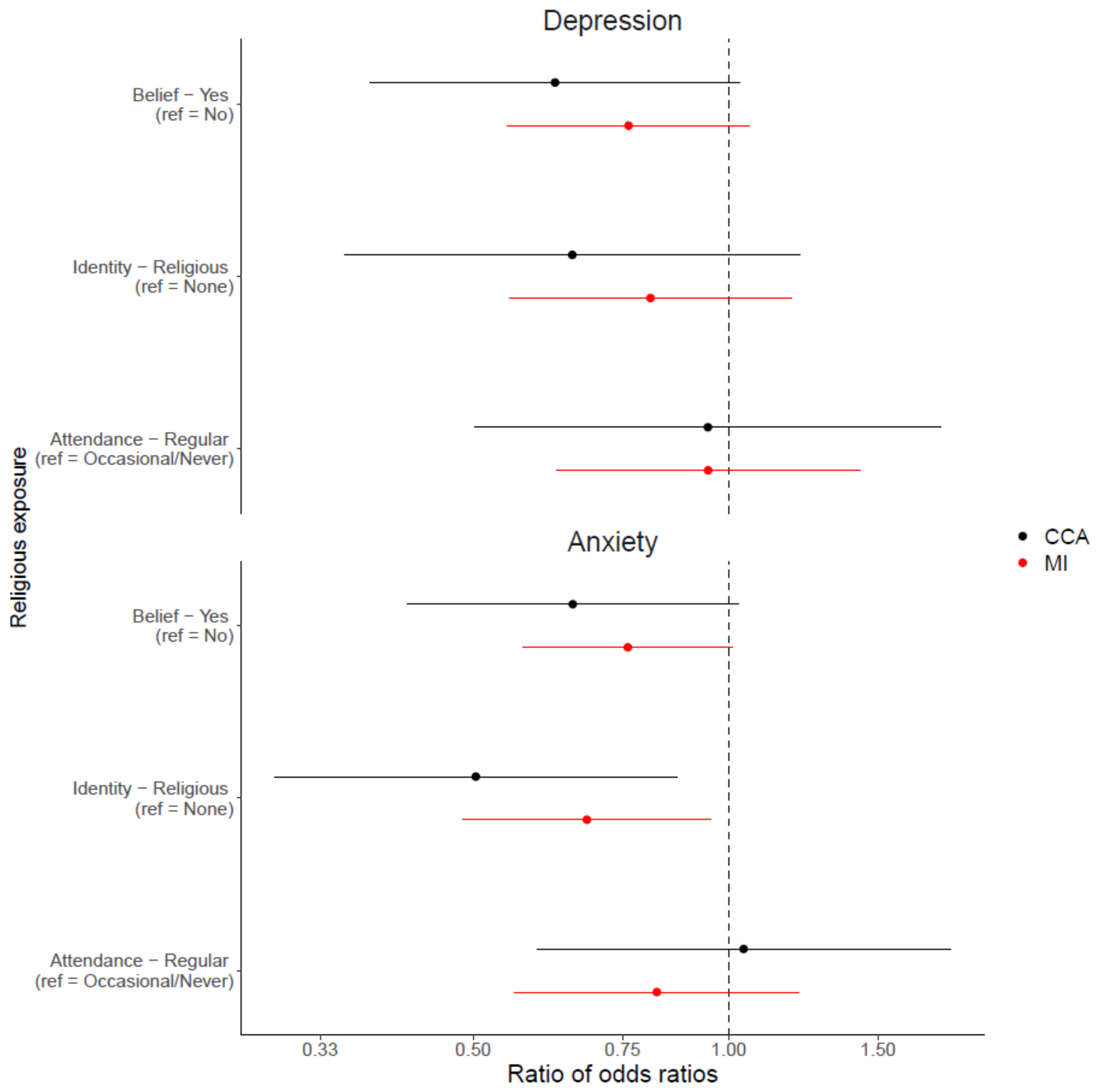

Figure S36: Results of the interaction analyses assessing whether the adjusted mother and partner results differ, with binary religious/spiritual belief and behaviour (RSBB) exposures and binary probable depression and anxiety diagnoses as outcomes comparing complete-case analysis (CCA) and multiple imputation (MI) results. Results in black are for the CCA, and those in red for MI. The dashed vertical line at ‘1’ indicates no difference between mothers and partners, with results below 1 meaning that the odds ratio estimate was lower in mothers, compared to partners. Error bars denote 95% confidence intervals. Full results are in Table S14 (for CCA) and Table S28 (for MI).

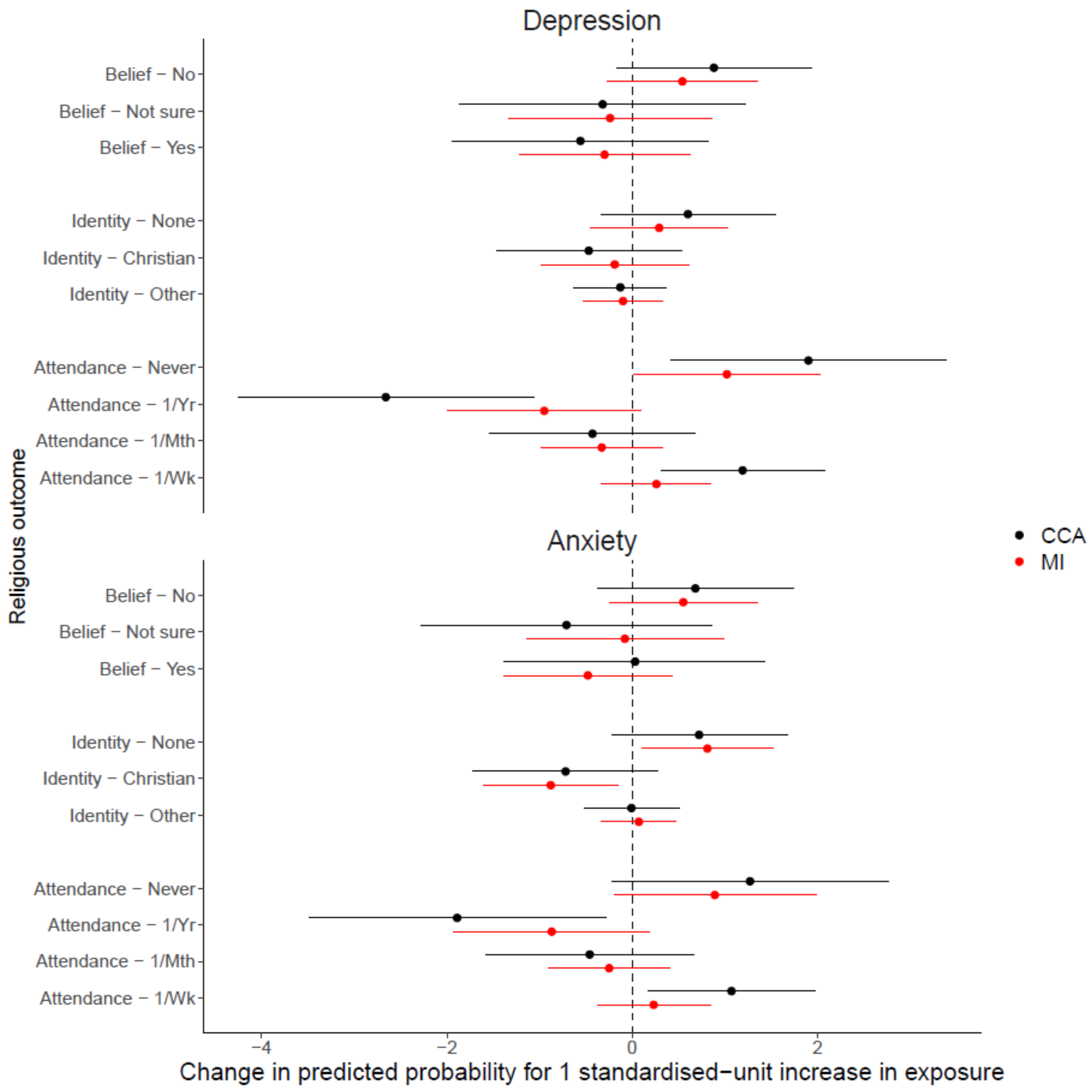

Figure S37: Results of the mothers analyses with standardised depression and anxiety scores as exposures and categorical religious/spiritual belief and behaviour (RSBB) as outcomes comparing complete-case analysis (CCA; *n* = 4,025) and multiple imputation (MI; *n* = 13,150) results. Results are for adjusted analyses, with CCA results in black and MI results in red. This plot displays the predicted change in the probability of the RSBB outcome for a one-standardised-unit increase in the mental health exposure, based on the associated multinomial regression model. The dashed vertical line at '0' indicates a null association. Error bars denote 95% confidence intervals. Full results of the multinomial models are in Tables S16 (for CCA) and S29 (for MI), with predicted probabilities in Tables S17 (for CCA) and S30 (for MI).

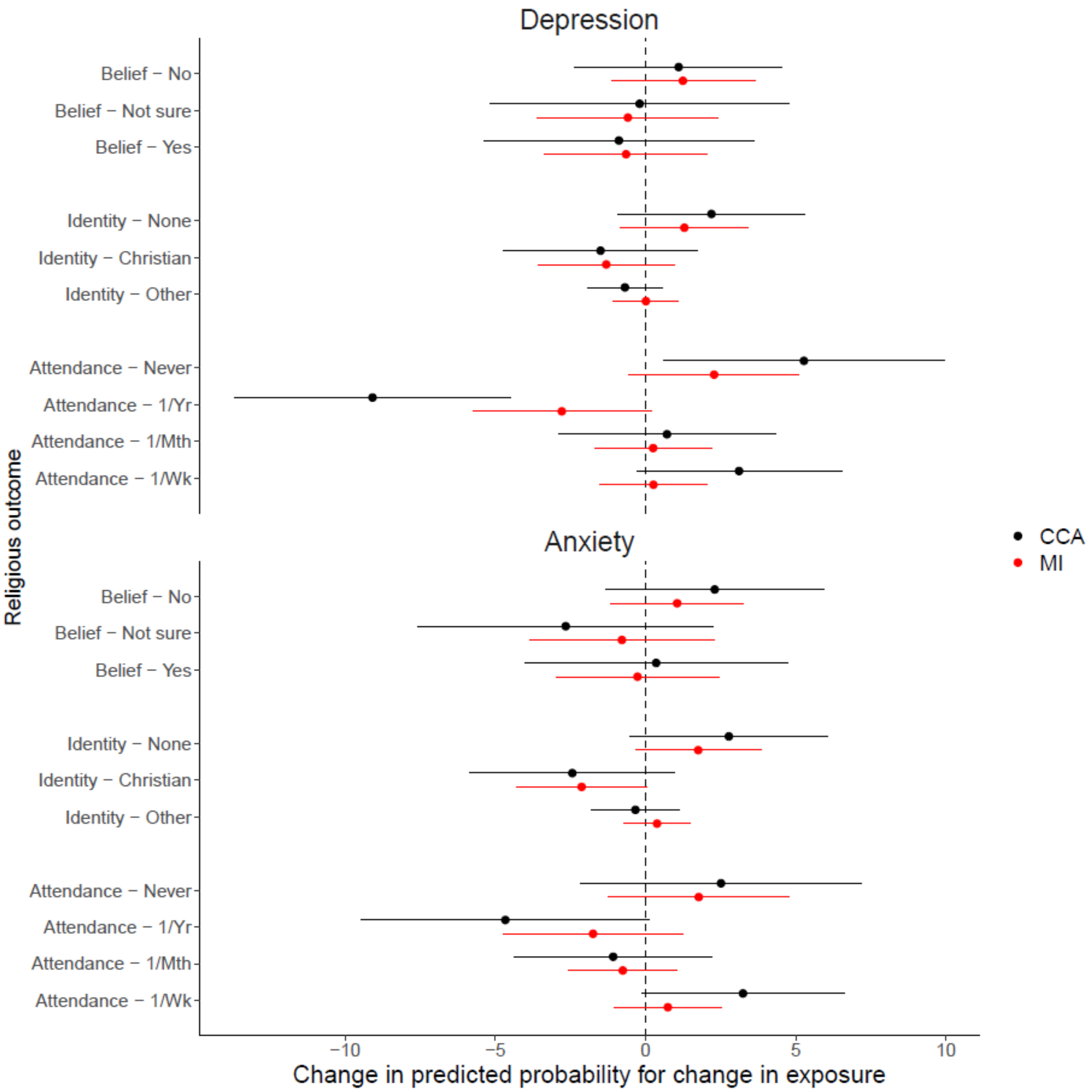

Figure S38: Results of the mothers analyses with binary probable depression and anxiety diagnoses as exposures and categorical religious/spiritual belief and behaviour (RSBB) as outcomes comparing complete-case analysis (CCA; *n* = 4,025) and multiple imputation (MI; *n* = 13,150) results. Results are for adjusted analyses, with CCA results in black and MI results in red. This plot displays the predicted change in the probability of the RSBB outcome for a change in the mental health exposure from 'no' to 'yes', based on the associated multinomial regression model. The dashed vertical line at '0' indicates a null association. Error bars denote 95% confidence intervals. Full results of the multinomial models are in Tables S16 (for CCA) and S29 (for MI), with predicted probabilities in Tables S17 (for CCA) and S30 (for MI).

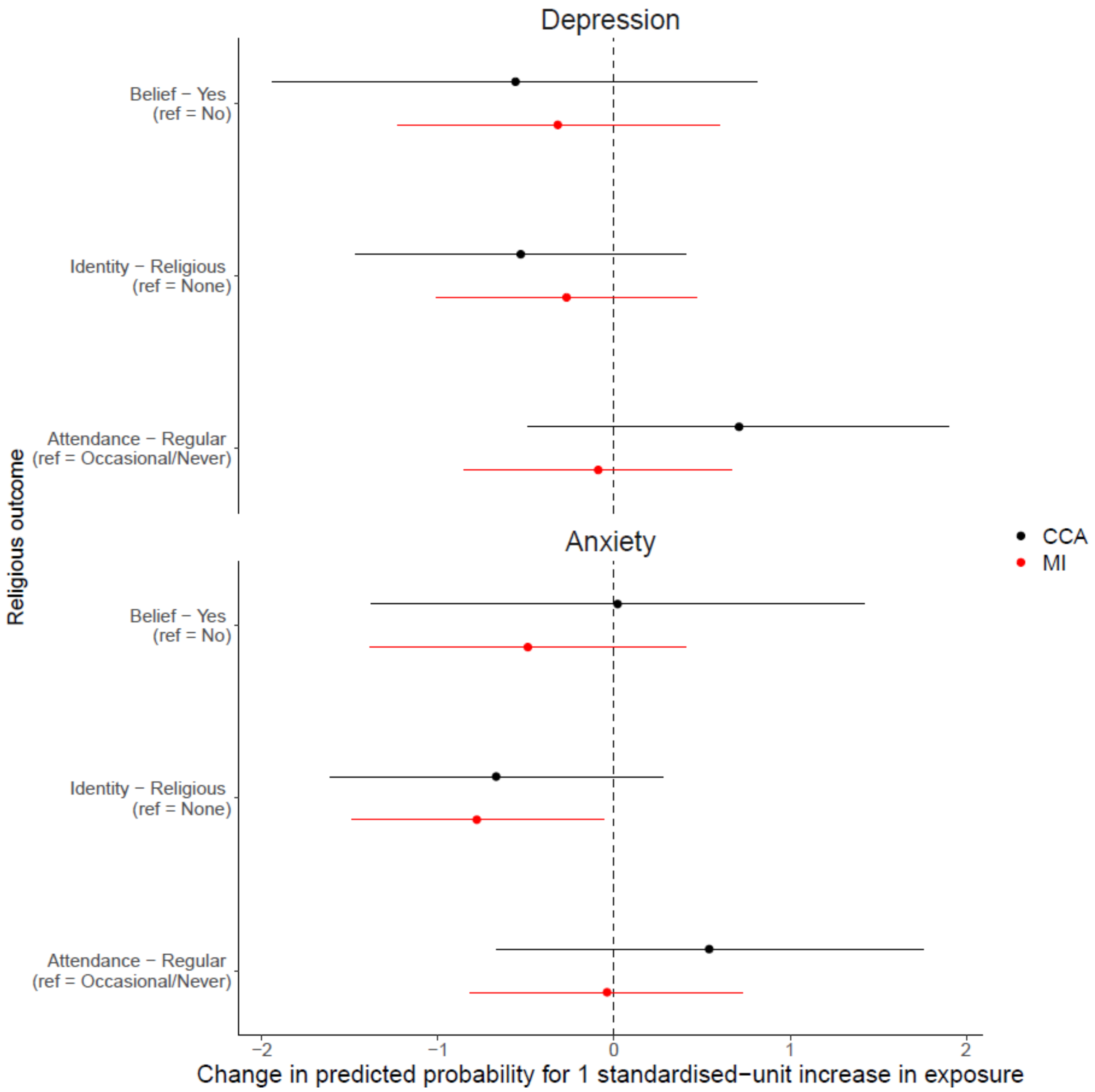

Figure S39: Results of the mothers analyses with standardised depression and anxiety scores as exposures and binary religious/spiritual belief and behaviour (RSBB) as outcomes comparing complete-case analysis (CCA; *n* = 4,025) and multiple imputation (MI; *n* = 13,150) results. Results are for adjusted analyses, with CCA results in black and MI results in red. This plot displays the predicted change in the probability of the RSBB outcome for a one-standardised-unit increase in the mental health exposure, based on the associated multinomial regression model. The dashed vertical line at '0' indicates a null association. Error bars denote 95% confidence intervals. Full results of the logistic models are in Tables S16 (for CCA) and S29 (for MI), with predicted probabilities in Tables S17 (for CCA) and S30 (for MI).

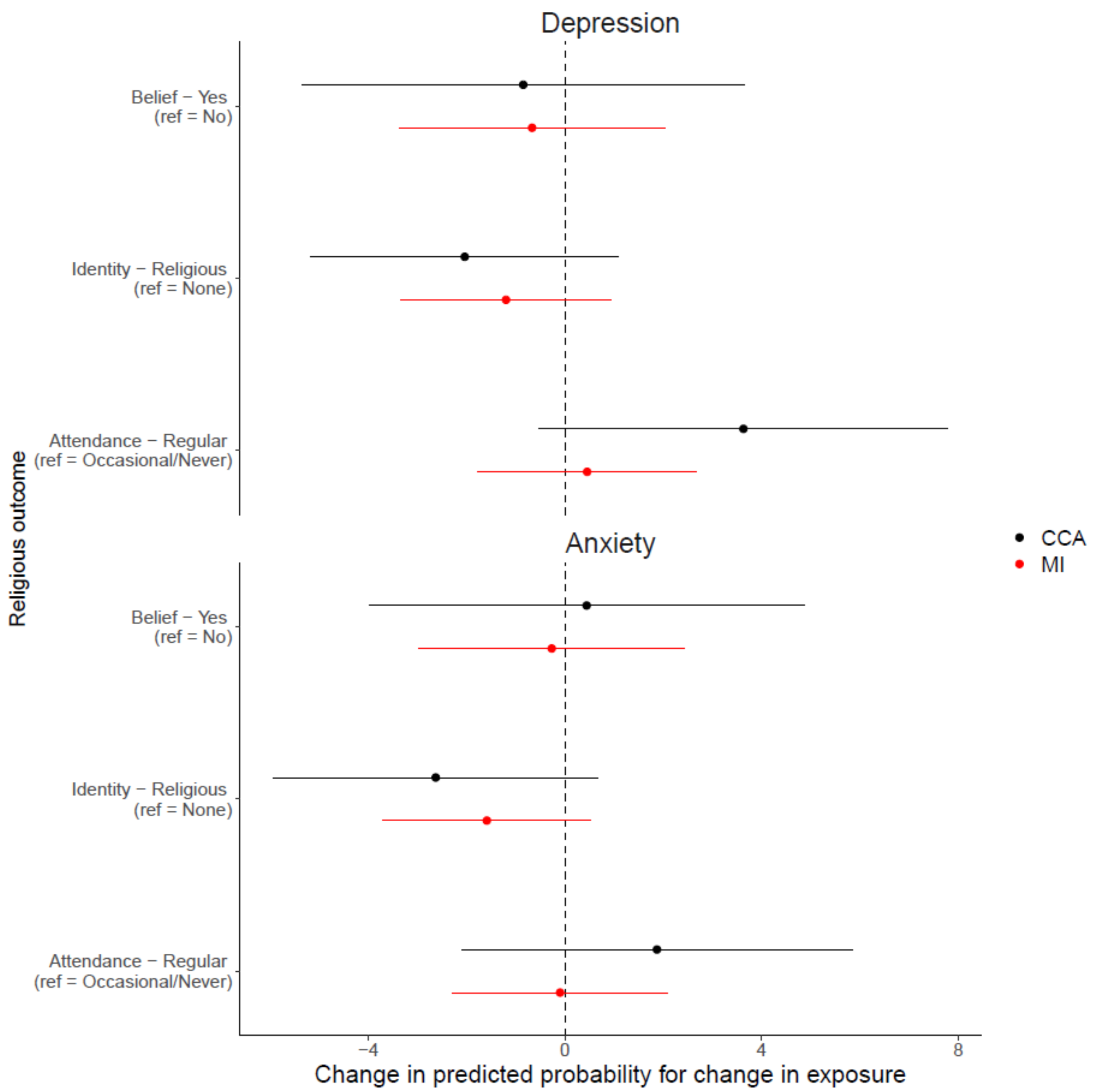

Figure S40: Results of the mothers analyses with binary probable depression and anxiety diagnoses as exposures and binary religious/spiritual belief and behaviour (RSBB) as outcomes comparing complete-case analysis (CCA; n = 4,025) and multiple imputation (MI; n = 13,150) results. Results are for adjusted analyses, with CCA results in black and MI results in red. This plot displays the predicted change in the probability of the RSBB outcome for a change in the mental health exposure from 'no' to 'yes', based on the associated multinomial regression model. The dashed vertical line at '0' indicates a null association. Error bars denote 95% confidence intervals. Full results of the logistic models are in Tables S16 (for CCA) and S29 (for MI), with predicted probabilities in Tables S17 (for CCA) and S30 (for MI).

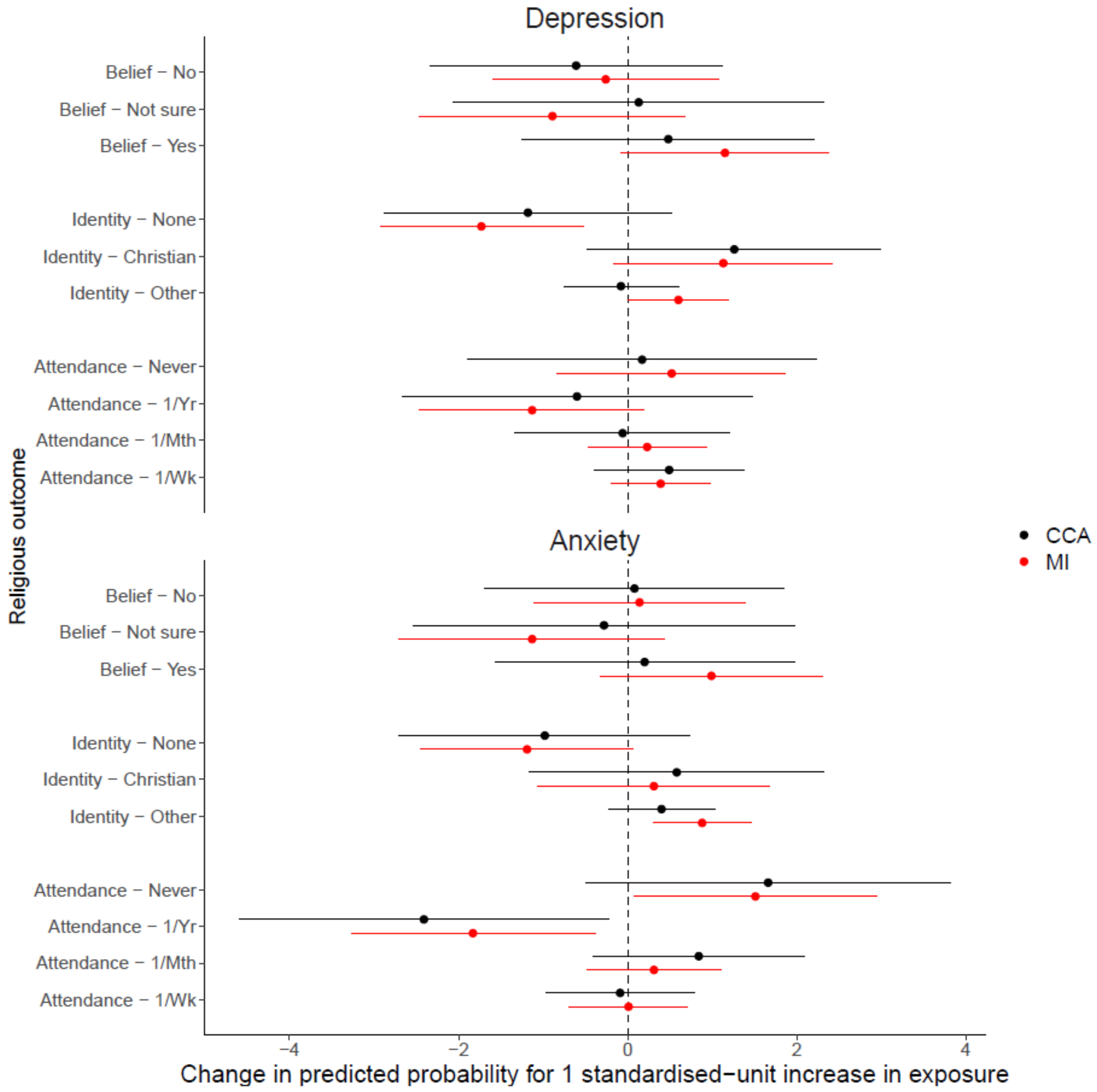

Figure S41: Results of the partners analyses with standardised depression and anxiety scores as exposures and categorical religious/spiritual belief and behaviour (RSBB) as outcomes comparing complete-case analysis (CCA; *n* = 2,120) and multiple imputation (MI; *n* = 9,887) results. Results are for adjusted analyses, with CCA results in black and MI results in red. This plot displays the predicted change in the probability of the RSBB outcome for a one-standardised-unit increase in the mental health exposure, based on the associated multinomial regression model. The dashed vertical line at '0' indicates a null association. Error bars denote 95% confidence intervals. Full results of the multinomial models are in Tables S19 (for CCA) and S31 (for MI), with predicted probabilities in Tables S20 (for CCA) and S32 (for MI).

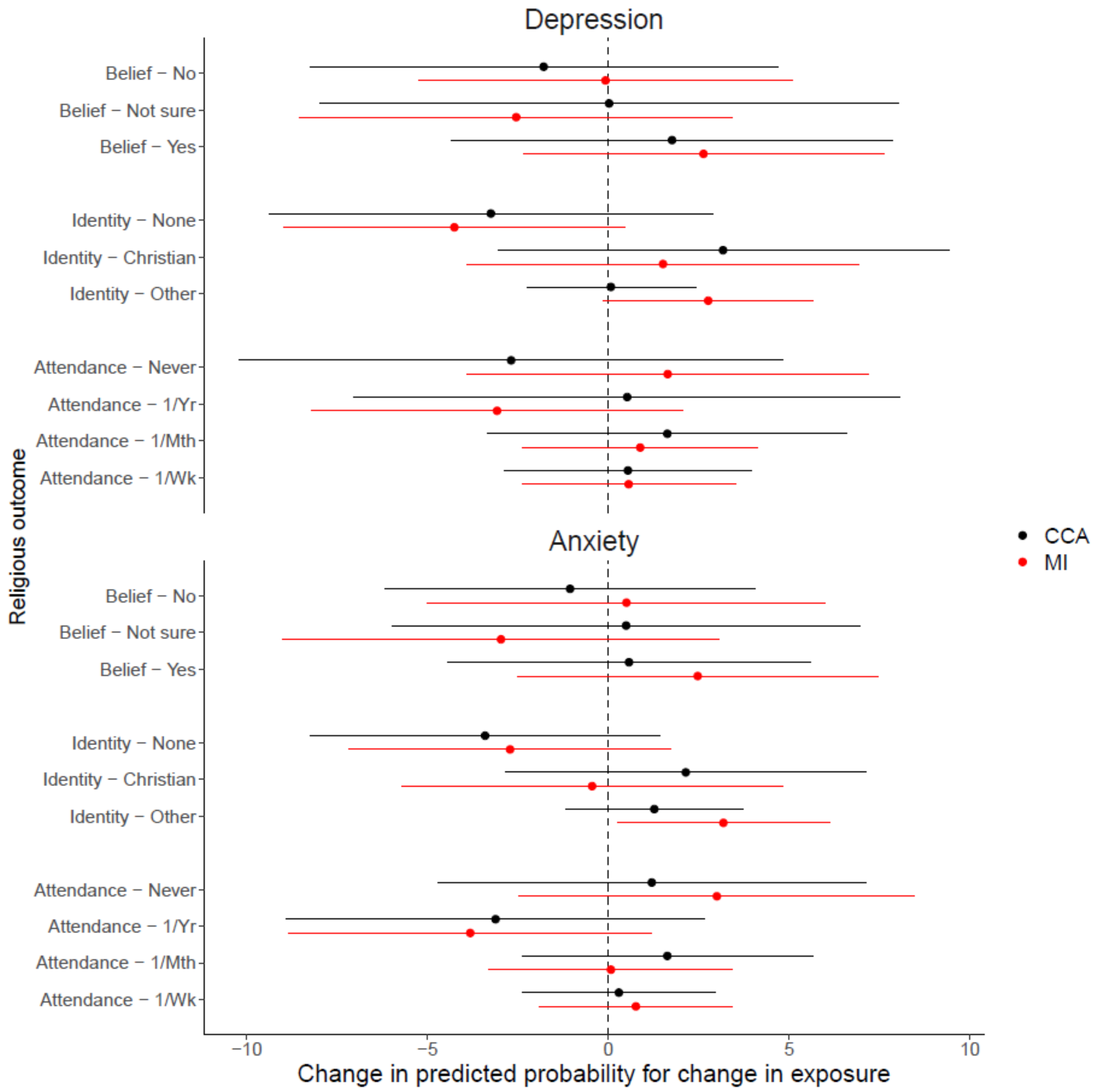

Figure S42: Results of the partners analyses with binary probable depression and anxiety diagnoses as exposures and categorical religious/spiritual belief and behaviour (RSBB) as outcomes comparing complete-case analysis (CCA; *n* = 2,120) and multiple imputation (MI; *n* = 9,887) results. Results are for adjusted analyses, with CCA results in black and MI results in red. This plot displays the predicted change in the probability of the RSBB outcome for a change in the mental health exposure from ‘no’ to ‘yes’, based on the associated multinomial regression model. The dashed vertical line at ‘0’ indicates a null association. Error bars denote 95% confidence intervals. Full results of the multinomial models are in Tables S19 (for CCA) and S31 (for MI), with predicted probabilities in Tables S20 (for CCA) and S32 (for MI).

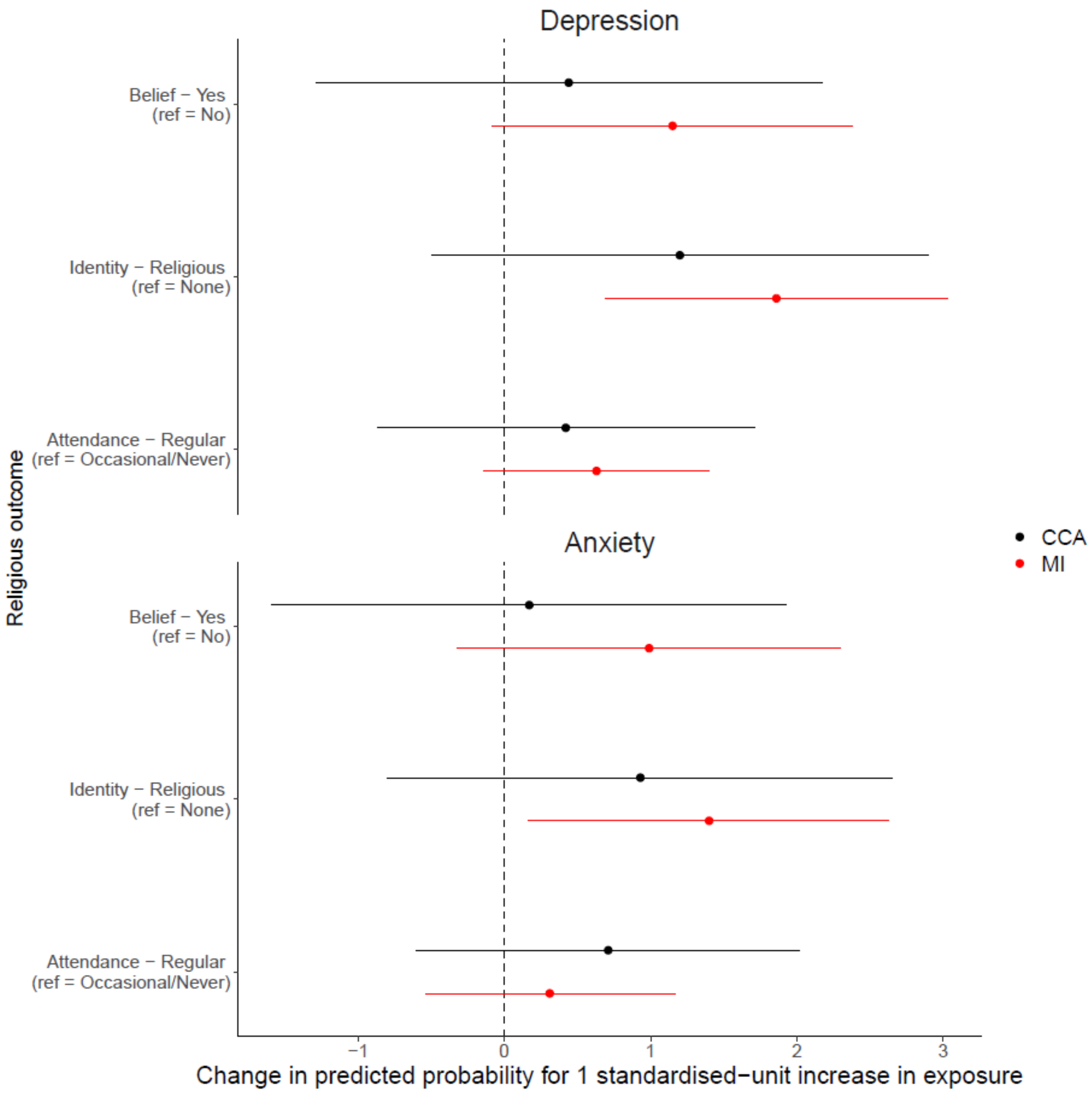

Figure S43: Results of the partners analyses with standardised depression and anxiety scores as exposures and binary religious/spiritual belief and behaviour (RSBB) as outcomes comparing complete-case analysis (CCA; *n* = 2,120) and multiple imputation (MI; *n* = 9,887) results. Results are for adjusted analyses, with CCA results in black and MI results in red. This plot displays the predicted change in the probability of the RSBB outcome for a one-standardised-unit increase in the mental health exposure, based on the associated multinomial regression model. The dashed vertical line at '0' indicates a null association. Error bars denote 95% confidence intervals. Full results of the logistic models are in Tables S19 (for CCA) and S31 (for MI), with predicted probabilities in Tables S20 (for CCA) and S32 (for MI).

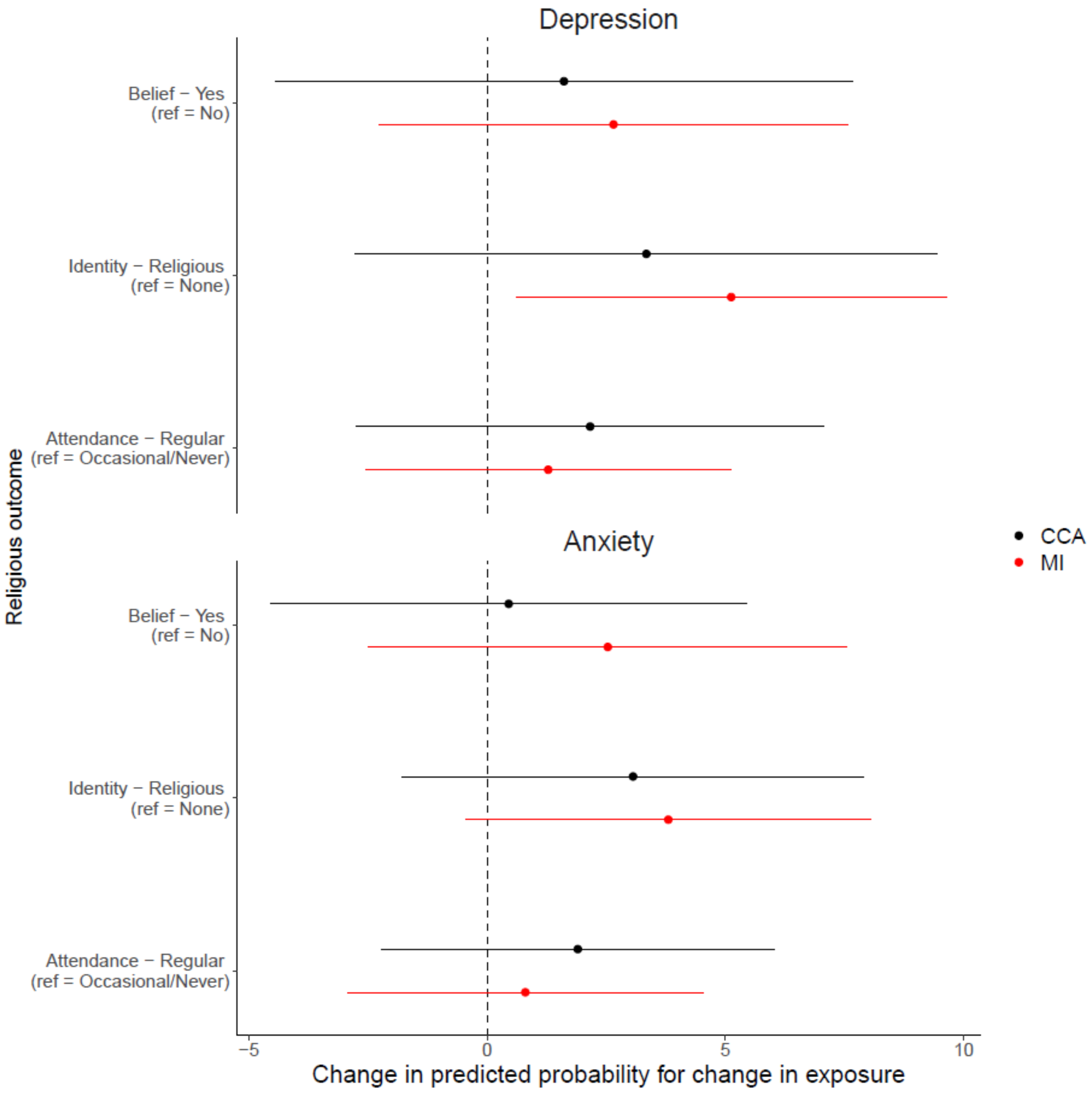

Figure S44: Results of the partners analyses with binary probable depression and anxiety diagnoses as exposures and binary religious/spiritual belief and behaviour (RSBB) as outcomes comparing complete-case analysis (CCA; *n* = 2,120) and multiple imputation (MI; *n* = 9,887) results. Results are for adjusted analyses, with CCA results in black and MI results in red. This plot displays the predicted change in the probability of the RSBB outcome for a change in the mental health exposure from 'no' to 'yes', based on the associated multinomial regression model. The dashed vertical line at '0' indicates a null association. Error bars denote 95% confidence intervals. Full results of the logistic models are in Tables S19 (for CCA) and S31 (for MI), with predicted probabilities in Tables S20 (for CCA) and S32 (for MI).

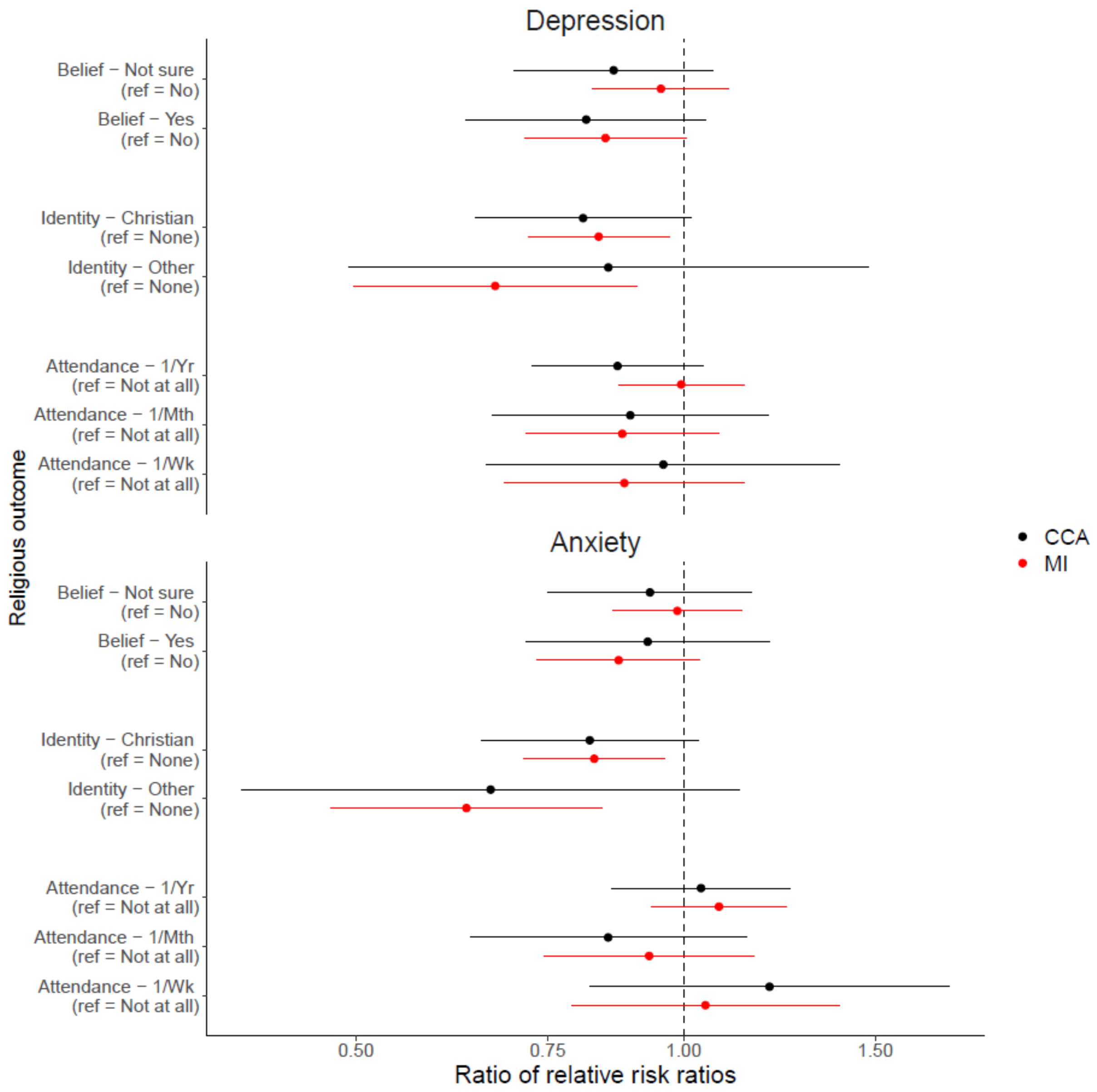

Figure S45: Results of the interaction analyses assessing whether the adjusted mother and partner results differ, with standardised continuous mental health as exposures and categorical religious/spiritual beliefs and behaviours (RSBB) as outcomes comparing complete-case analysis (CCA) and multiple imputation (MI) results. Results in black are for the CCA, and those in red for MI. The dashed vertical line at ‘1’ indicates no difference between mothers and partners, with results below 1 meaning that the relative risk ratio (RRR) estimate was lower in mothers, compared to partners. Error bars denote 95% confidence intervals. Full results are in Table S21 (for CCA) and Table S33 (for MI).

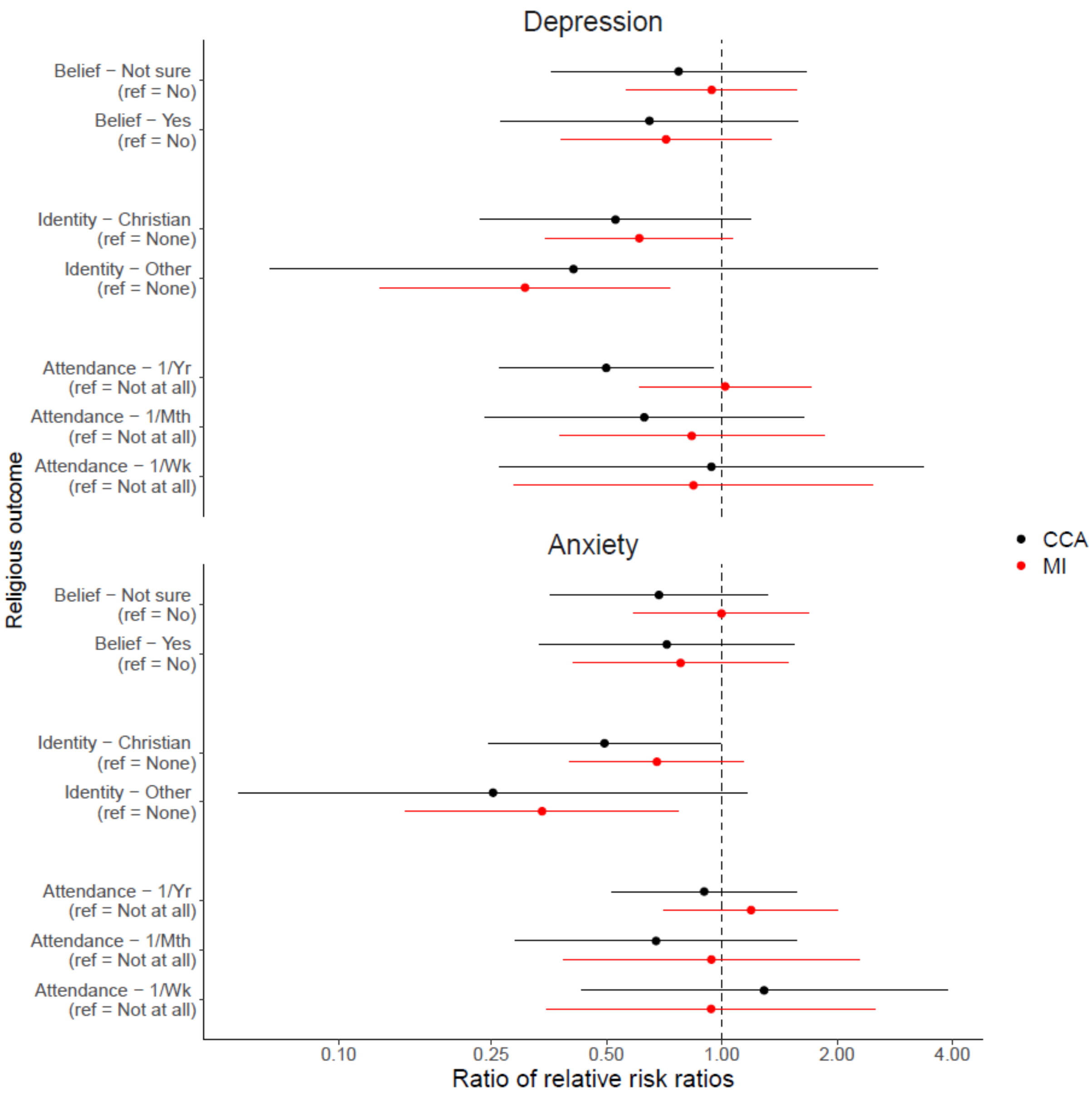

Figure S46: Results of the interaction analyses assessing whether the adjusted mother and partner results differ, with binary probable depression and anxiety diagnoses as exposures and categorical religious/spiritual beliefs and behaviours (RSBB) as outcomes comparing complete-case analysis (CCA) and multiple imputation (MI) results. Results in black are for the CCA, and those in red for MI. The dashed vertical line at '1' indicates no difference between mothers and partners, with results below 1 meaning that the relative risk ratio (RRR) estimate was lower in mothers, compared to partners. Error bars denote 95% confidence intervals. Full results are in Table S21 (for CCA) and Table S33 (for MI).

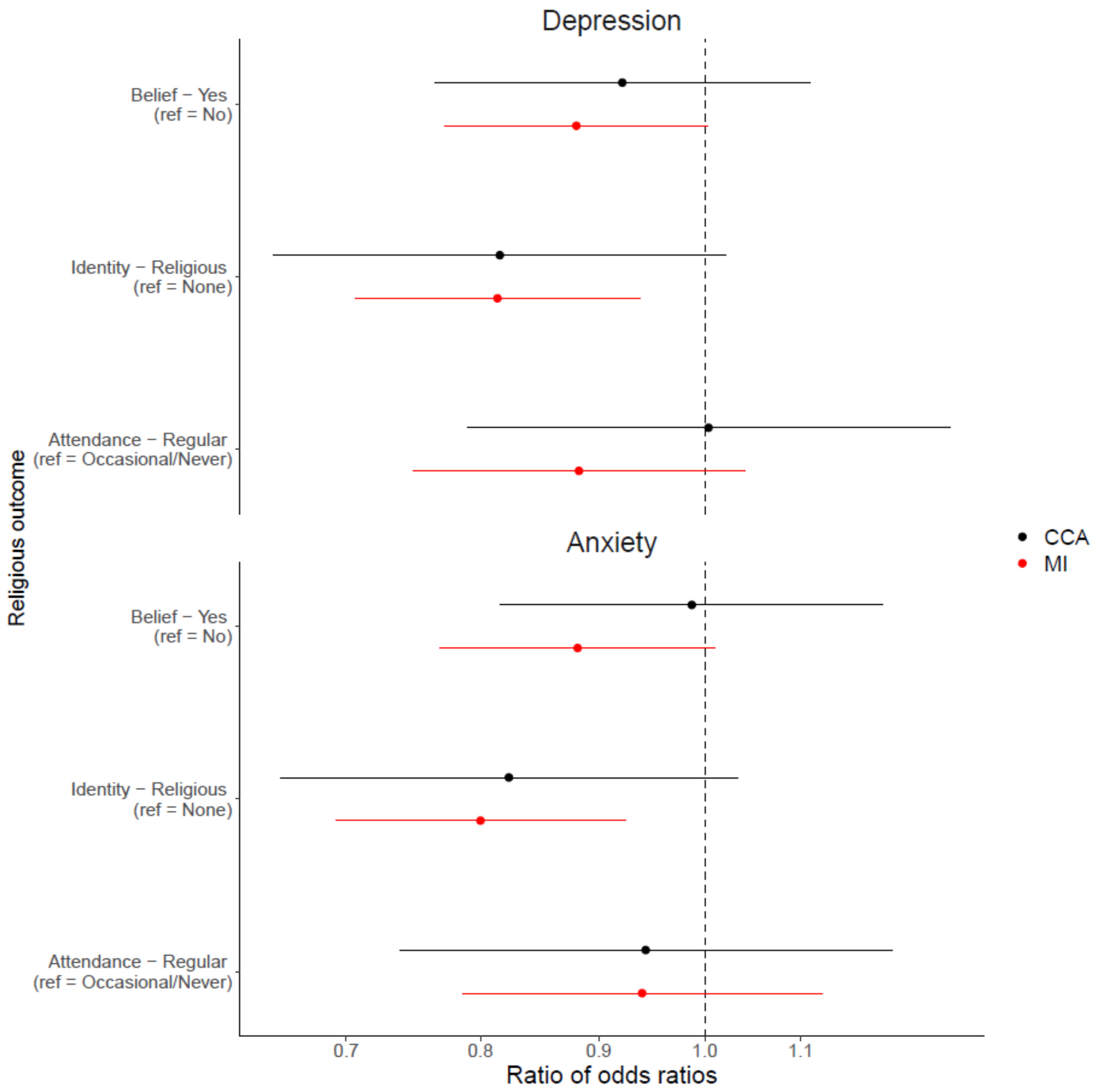

Figure S47: Results of the interaction analyses assessing whether the adjusted mother and partner results differ, with standardised continuous mental health as exposures and binary religious/spiritual beliefs and behaviours (RSBB) as outcomes comparing complete-case analysis (CCA) and multiple imputation (MI) results. Results in black are for the CCA, and those in red for MI. The dashed vertical line at '1' indicates no difference between mothers and partners, with results below 1 meaning that the odds ratio (OR) estimate was lower in mothers, compared to partners. Error bars denote 95% confidence intervals. Full results are in Table S22 (for CCA) and Table S34 (for MI).

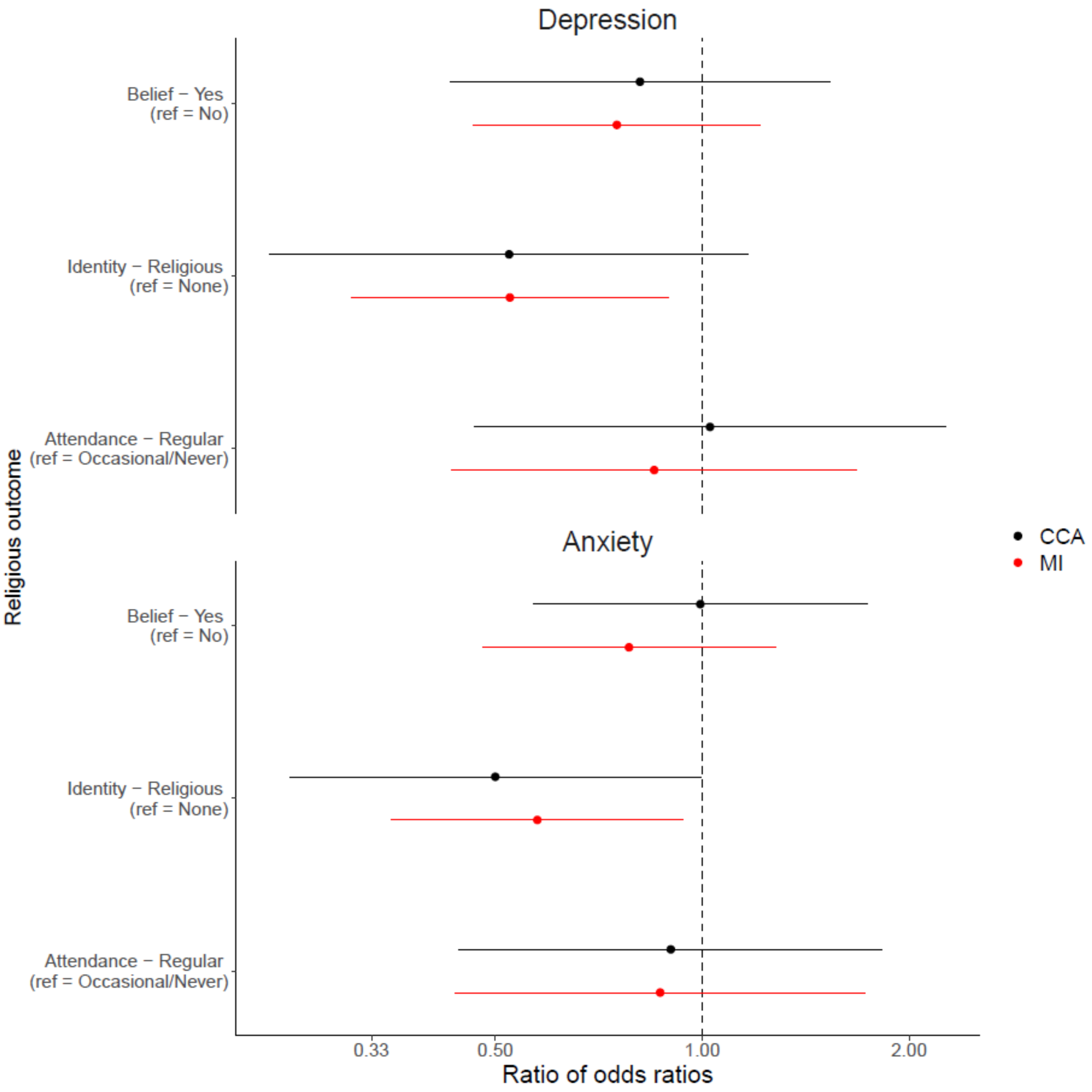

Figure S48: Results of the interaction analyses assessing whether the adjusted mother and partner results differ, with binary probable depression and anxiety diagnoses as exposures and binary religious/spiritual beliefs and behaviours (RSBB) as outcomes comparing complete-case analysis (CCA) and multiple imputation (MI) results. Results in black are for the CCA, and those in red for MI. The dashed vertical line at '1' indicates no difference between mothers and partners, with results below 1 meaning that the odds ratio (OR) estimate was lower in mothers, compared to partners. Error bars denote 95% confidence intervals. Full results are in Table S22 (for CCA) and Table S34 (for MI).

References in Supporting Information

1. van Buuren S. Flexible Imputation of Missing Data. Boca Raton, FL: CRC Press; 2018.
2. Nowicki S, Duke MP. A Locus of Control Scale for Noncollege as Well as College Adults. J Pers Assess. 1974;38: 136–137.
3. Boyce P, Parker G. Development of a scale to measure interpersonal sensitivity. Aust N Z J Psychiatry. 1989;23: 341–351.
