# Supplementary material for: Exploring bidirectional causality between religion and mental health: A longitudinal study using data from the parent generation of a UK birth cohort
Source: PLoS One. 2025 Mar 18;20(3):e0319796. doi: 10.1371/journal.pone.0319796 (PMC11918439; doi:10.1371/journal.pone.0319796)
Supplement: Attachment — Submitted filename: RSBBandParentalMH_ResponseToReviewers_rev1.docx [file pone.0319796.s002.docx]

**Response to Reviewer 1**

The article in question stands out for its well-organized structure and the use of an appropriate academic style, which facilitates the communication of the results and analyses carried out. Each section of the work—from the initial presentation to the data analysis and the subsequent discussion—is articulated clearly and coherently, allowing the reader to easily follow the logical flow of the discourse. The final images, carefully chosen and well integrated into the text, play a crucial role, as they make the results more accessible and immediately understandable, thereby contributing to a better assimilation of the information presented.

However, there are significant improvements that could be made to the introductory part. Currently, the initial overview could benefit from a broader context that situates the work within the existing research framework. Including references to previous studies and similar research would not only enrich the content but also allow for a clearer highlighting of how this specific study fits into the current scientific dialogue. An introduction that outlines the gaps in the previously existing literature and emphasizes the originality of the adopted approach would provide readers with a deeper understanding of the value and importance of the work, making the contribution even more significant. This contextual enrichment would not only serve to attract the reader's interest but also justify the relevance of the research as a whole.

We thank the reviewer for their positive review and helpful suggestions on how to strengthen our paper. We have now updated the introduction accordingly, providing more detail about previous studies in this area, highlighting gaps in previous research, and emphasising how our project aims to build upon this previous work. Note that page and line numbers refer to the unmarked version of the manuscript without tracked changes.

These updates include:

- The importance of exploring these relationships in a range of societies, specifically outside of the US (Pages 3-4, lines 98-109): “For instance, the majority of previous studies – 75%, according to a recent meta-analysis (24) – were based in the US. However, as the US is more religious than other, more secular, countries, such as the majority of Europe (27), these findings may be limited in their generalisability (22). […]. There is therefore a need to conduct studies using longitudinal data in a range of populations to explore how replicable and generalisable these patterns are, and – ultimately – to understand the reasons for any cross-cultural variability.”
- The importance of exploring the relationship between religion and mental health using a range of religiosity measures (Page 4, lines 111-123): “Additionally, much previous work – over half (24) – has focused on religious attendance rather than other aspects of religiosity. […]. Additional research is required to confirm and further investigate these patterns, specifically by exploring the relationship between religiosity and mental health using a range of RSBB variables.”
- The methodological, theoretical and practical importance of understanding bidirectional causation between religion and mental health (Page 4, lines 125-140): “A further open question is the extent to which mental health issues cause RSBB. This has been explored by fewer studies, although existing research has found that depression and anxiety are associated with lower rates of subsequent religious attendance (11,12,28). This is important from a methodological perspective, as if changes in mental health cause subsequent changes in religiosity, then cross-sectional studies investigating religion and mental health are at risk of reverse causality and may be unable to estimate their causal effect of interest without bias (14). From theoretical and practical perspectives, this potential bidirectional causation can feed into theories of how religion and mental health interact, as well as help inform potential interventions to improve mental health. For instance, a common symptom of depression is withdrawal from usual social activities, which may include religious activities (11); conversely, it is also possible that mental health issues could result in an increase in religious coping (33), and hence greater subsequent religiosity, although most longitudinal studies so far have reported a negative relationship between mental health problems and subsequent religiosity. We will therefore also investigate this under-explored topic here to assess whether there is potential bidirectional causation between RSBB and mental health.”
- Including additional literature regarding reasons for potential gender differences in religion and mental health, and justifying why more research in this area is needed (Pages 4-5, lines 142-152): “Finally, some studies have also noted gender-specific associations between religion and mental health (28). In general, there appears to be a stronger protective association between religion and depression among women, with many studies reporting null (15), or even positive/harmful (34), associations among men. Associations between depression and subsequent attendance have also been reported for women, but not men (11). One potential reason for this is gender differences in coping, with women generally more likely to engage in religious coping in times of stress (35), although for an exception finding few gender differences in a largely-secular society see (36). As women are also more likely to use social support as a method of coping (35) – which could include support from co-religionists – this may also play a role in any gender differences. However, as the number of longitudinal studies exploring such gender differences in religion and mental health is small, there is a need for more research in this area.”

**Response to Reviewer 2**

The study benefits from a robust dataset and advanced statistical approaches, such as pre-registered analyses and E-value sensitivity tests. However, the results were largely inconclusive, with small or null associations found between religiosity and mental health. While the study provides valuable insights, several methodological and conceptual issues limit its contributions.

We thank the reviewer for their comments and for seeing merit in our study. We have responded to all comments in turn, with the reviewer’s original comments in black font, and our responses in blue. Note that page and line numbers refer to the unmarked version of the manuscript without tracked changes.

Recommendations for Revision
1. Enhance Measurement Precision:
- Expand religiosity measures to include aspects like private prayer, intrinsic religiosity, and spiritual coping.
- Consider using diagnostic tools or broader measures of mental health to improve outcome validity.

While we agree with the spirit of the reviewer’s suggestion – and would have liked to explore this topic in greater detail – unfortunately given the data available we are unable to act on this suggestion. As the ALSPAC religion and mental health data used here was collected in the early 1990s, we are unable to explore these different aspects of religiosity or mental health suggested by the reviewer because they are simply not available (without use of a time machine!).

Despite this pragmatic limitation, we chose these aspects of religiosity because they “…cover a range of theoretically-relevant religious beliefs and behaviours, including ‘believing’ (religious belief), ‘behaving’ (religious attendance) and ‘belonging’ (religious identity/affiliation) (29). These – or very similar – questions have been widely used in previous religion research, both in ALSPAC (46,47) and more broadly in psychological, epidemiological and sociological disciplines (12,48,49).” (Methods, page 7, lines 231-235). As our chosen measures cover a range of aspects of religiosity, we believe they are valid and appropriate for the aims of our analyses, and in line with many other similar studies on this topic.

Nonetheless, in our original submission we noted this as a limitation briefly in the Methods (page 7, lines 236-238: “We do note, however, that these variables may not cover the full range of religious beliefs, behaviours and experiences; we return to this important point in the discussion”) and in more detail in the Discussion (page 22, lines 914-928): “As highlighted in the Methods section, our measurement of RSBB could be an additional potential limitation. While we explored a number of theoretically-relevant religion variables – religious belief, identity and attendance (29) – it is possible that other aspects of religiosity not explored here may have different associations with mental health, such as positive (or negative) religious coping, non-religious spirituality, intrinsic vs extrinsic religiosity, and private religious behaviours (e.g., prayer (24,67)). In addition, the focus on single questions to measure the traits explored may not fully capture these beliefs/behaviours compared to more detailed and validated multi-item scales, which could contribute to measurement error and bias. However, given our use of secondary ALSPAC data this was unfortunately unavoidable, as is common with many other longitudinal studies on this topic which focus on single-item measures of RSBB (e.g., (12,19,22,28)). A key avenue for future research would therefore be to embed a larger range of well-validated RSBB measures and scales within longitudinal studies to assess whether findings replicate using these more robust measures and further explore similarities and differences between different aspects of religiosity and their relations with mental health.”

Regarding the measurement of mental health, both of our measures of depression and anxiety are psychometrically-validated, with high internal reliability and construct validity, and have been used extensively in epidemiological studies of mental health. As we say in the Methods (pages 7-8, lines 262-266): “Both scales had high internal validity (Cronbach’s alpha > 0.80) and demonstrated construct validity when compared against other validated assessments of depression and anxiety in ALSPAC mothers (Center for Epidemiologic Studies Depression Scale for EPDS, and State and Trait subscales of the Spielberger State-Trait Anxiety Inventory for CCEI-A, respectively (38)).” We therefore believe that our measures of mental health are robust and valid measures of our constructs of interest.

2. Address Missing Data:
- Implement multiple imputation or sensitivity analyses to better address attrition bias.
- Provide a detailed comparison of participants with and without complete data to clarify potential biases.

In our pre-registration document and initial full paper we justified why we did not perform multiple imputation, despite missing data and the risk of selection bias. Briefly, this is because: i) religiosity only has a weak relationship with continued ALSPAC participation; ii) previous work exploring potential selection bias in ALSPAC on religion and mental health found little evidence of bias; iii) the inclusion of a wide range of covariates, including baseline mental health and religiosity, in the analysis models makes the ‘Missing-At-Random’ assumption more plausible; and iv) Beyond the included confounders, there are no additional auxiliary variables to include in the imputation model to predict the exposures and outcomes, and hence remove any potential bias (see pages 21-22, lines 888-908). We therefore believed that our complete-case analyses would be broadly unbiased, and that multiple imputation would not be necessary.

However, as an additional sensitivity analyses we have now performed multiple imputation (page 22, lines 908-912): “Although we believe our complete-case analyses to be largely unbiased, as suggested by a reviewer we performed multiple imputation to impute missing data as an additional sensitivity analysis; results were broadly consistent with the complete-cases results, albeit with somewhat greater precision (for more details, see section S2 of the Supporting Information).”

We have also provided a comparison of participants in the full ALSPAC samples vs those included in the complete-case analyses (page 11, lines 419-423): “Descriptive statistics for all variables, in both the full ALSPAC sample and the complete-case analyses, are in Tables S5 (mothers) and S6 (partners). Compared to the full sample, participants with complete data were older, had higher levels of educational attainment, from less-deprived neighbourhoods, less likely to have an ‘other than White’ ethnicity, slightly more religious, and had marginally lower depression and anxiety scores”.

3. Examine Nonlinear Relationships:
- The findings suggest potential non-linear associations (e.g., mental health influencing both increased and decreased religious attendance). Future analyses should formally test for such patterns.

We are afraid that we are a bit unclear exactly what the reviewer is asking us to do here. The models with mental health as the exposure and religious attendance as the outcome which suggest this non-linear relationship are multinomial models, and hence *are* non-linear models because they do not assume a linear relationship between the different outcome levels.

4. Discuss Limitations More Transparently:
- Acknowledge the limitations of generalizing findings to non-secular populations.

We believe that this has already been adequately addressed in our Discussion. For instance, in our ‘Implications’ sub-section we discuss heterogeneity in results between countries (pages 19-22, lines 790-807): “Although much theoretical work (7,8), and some longitudinal empirical studies (12,23), have suggested that religion may be protective against subsequent mental health problems, we find little convincing evidence for such associations in this UK population. This is consistent with previous work indicating that such associations are highly heterogeneous between studies (22–24), although the reasons for this variation are unclear. Differences in religiosity between countries may be one potential explanation, with rates of religious belief and attendance lower in the UK, and other secularised Western European countries, compared to the US (27), where much previous work on religion and mental health has been conducted. Religion may plausibly have less of an impact on people’s lives in more secular countries, and hence less impact on mental health. For instance, a meta-analysis on the relationship between RSBB and mental health in German-speaking areas found a much smaller protective association compared to studies from the US (67). However, other longitudinal work comparing a range of European countries with different rates of religiosity found similar null associations with subsequent depression (22), arguing against this interpretation. Additional work is needed to understand the reasons for this variation between studies, and this heterogeneity should be considered when thinking about implications for religion/spirituality-based mental health interventions (68), as their efficacy may be highly context-specific.”

We then refer back to this later on when discussing limitations of generalisability (page 22, lines 930-935): “Generalisability may also be a concern. Both religion and mental health have changed considerably since these data were collected in the early 1990s, with religiosity declining (39,78) and increased societal acceptance, awareness and understanding of mental health issues. This makes it difficult to know how generalisable these results are to even the current UK population, let alone other populations with different levels of religious belief, different religions, and different approaches to mental health.”

- Discuss the potential impact of residual confounding and measurement error on null results.

A section on measurement error has now been included in the Discussion (page 21, lines 857-867): “Relatedly, measurement error in either our confounders (i.e., residual confounding), or our exposures or outcomes could potentially result in bias (70). While we again cannot rule this out, our use of a wide range of confounders for each key facet of interest (e.g., baseline depression and anxiety for prior mental health; baseline religious belief, identity and attendance for prior religiosity; a range of proxies for socio-economic position) should minimise somewhat the risk of residual confounding. Similarly, our use of validated mental health scales ought to reduce the risk of measurement error in these variables. While non-differential measurement error can result in bias towards the null, this is not always the case, and different types of measurement error can bias results either towards or away from the null (71); the impact of measurement bias on our results – if any – is therefore difficult to establish with any degree of certainty.”

5. Strengthen Interpretative Framework:
- Delve deeper into the implications of gender-specific differences and why religious belief may show protective associations in women but harmful effects in men.

Additional background for potential gender difference has been added to the Introduction (pages 4-5, lines 142-152): “One potential reason for this is gender differences in coping, with women generally more likely to engage in religious coping in times of stress (35), although see (36). As women are also more likely to use social support as a method of coping (35) – which could include support from co-religionists – this may also play a role in any gender differences. However, as the number of longitudinal studies exploring such gender differences in religion and mental health is small, there is a need for more research in this area.”

We have also expanded upon our gender difference results in the ‘Implications’ sub-section of our Discussion (page 20, lines 828-836): “Finally, we observed – at best – weak evidence for gender differences in these relationships, with a somewhat protective association in mothers and a somewhat harmful association in their partners. However, these effect sizes were relatively small and often consistent with no difference, meaning the evidence for gender differences is suggestive but ultimately inclusive. If there are gender differences, this could perhaps be explained by different coping styles, with women more likely to use religion and social support (including religious social support) as coping mechanisms, compared to men (35). Ultimately, more research is needed to understand first whether these gender differences are robust and replicable, and second what the potential mechanisms explaining this difference are.”

- Explore alternative theoretical frameworks, such as coping theories, that may explain bidirectional relationships more robustly.

Religious coping has been discussed above, in relation to explanations for any potential gender differences. We have also briefly discussed the role of religious coping in relation to bidirectional causality in the Introduction (page 4, lines 131-138): “From theoretical and practical perspectives, this potential bidirectional causation can feed into theories of how religion and mental health interact, as well as help inform potential interventions to improve mental health. For instance, a common symptom of depression is withdrawal from usual social activities, which may include religious activities (11); conversely, it is also possible that mental health issues could result in an increase in religious coping (33), and hence greater subsequent religiosity, although most longitudinal studies so far have reported a negative relationship between mental health problems and subsequent religiosity.”

6. Condense Statistical Presentation:
- While detailed statistical tables are helpful, consider summarizing key findings in the main text to improve readability and focus on meaningful results.

We are afraid that we do not quite understand the reviewer here, as we believe we are already following the reviewer’s suggestion! There are no statistical tables in the main text (only figures summarising the key results; detailed statistical tables are presented in the Supporting Information), and we did try our best to summarise the key findings in the main text, without overwhelming readers with statistical information (indeed, our clear presentation of results was something reviewer 1 picked up on and praised us for!)
